# KM-408, a novel phenoxyalkyl derivative as a potential anticonvulsant and analgesic compound for the treatment of neuropathic pain

**DOI:** 10.1007/s43440-022-00431-7

**Published:** 2022-11-19

**Authors:** Anna Waszkielewicz, Henryk Marona, Katarzyna Pańczyk-Straszak, Barbara Filipek, Anna Rapacz, Kinga Sałat, Monika Kubacka, Agnieszka Cios, Filip Fedak, Maria Walczak, Urszula Hubicka, Anna Kwiecień, Barbara Żuromska-Witek, Przemysław W. Szafrański, Paulina Koczurkiewicz-Adamczyk, Elżbieta Pękala, Katarzyna Przejczowska-Pomierny, Krzysztof Pociecha, Elżbieta Wyska

**Affiliations:** 1 Department of Bioorganic Chemistry, Chair of Organic Chemistry, Faculty of Pharmacy, Jagiellonian University Medical College, Medyczna 9, 30-688 Kraków, Poland; 2 Department of Pharmacodynamics, Chair of Pharmacodynamics, Faculty of Pharmacy, Jagiellonian University Medical College, Medyczna 9, 30-688 Kraków, Poland; 3 Department of Clinical Pharmacy, Faculty of Pharmacy, Jagiellonian University Medical College, Medyczna 9, 30-688 Kraków, Poland; 4 Jagiellonian Centre for Experimental Therapeutics (JCET), Jagiellonian University, Bobrzyńskiego 14, 30-348 Kraków, Poland; 5 Chair and Department of Toxicology, Faculty of Pharmacy, Jagiellonian University Medical College, Medyczna 9, 30-688 Kraków, Poland; 6 Chair of Inorganic and Analytical Chemistry, Faculty of Pharmacy, Jagiellonian University Medical College, Medyczna 9, 30-688 Kraków, Poland; 7 Chair of Organic Chemistry, Faculty of Pharmacy, Jagiellonian University Medical College, Medyczna 9, 30-688 Kraków, Poland; 8 Department of Pharmaceutical Biochemistry, Faculty of Pharmacy, Jagiellonian University Medical College, Medyczna 9, 30-688 Kraków, Poland; 9 Department of Pharmacokinetics and Physical Pharmacy, Faculty of Pharmacy, Jagiellonian University Medical College, Medyczna 9, 30-688 Kraków, Poland

**Keywords:** Aminoalkanols, Synthesis, Degradation studies, Anticonvulsant, Analgesic, Neuropathic pain, Sigma (σ), 5-HT, Safety profile, Pharmacokinetics, Metabolites

## Abstract

**Background:**

Epilepsy frequently coexists with neuropathic pain. Our approach is based on the search for active compounds with multitarget profiles beneficial in terms of potential side effects and on the implementation of screening for potential multidirectional central activity*.*

**Methods:**

Compounds were synthesized by means of chemical synthesis. After antiseizure and neurotoxicity screening in vivo, **KM-408** and its enantiomers were chosen for analgesic activity evaluations. Further safety studies included acute toxicity in mice, the effect on normal electrocardiogram and on blood pressure in rats, whole body plethysmography in rats, and in vitro and biochemical assays. Pharmacokinetics has been studied in rats after *iv* and *po* administration. Metabolism has been studied in vivo in rat serum and urine. Radioligand binding studies were performed as part of the mechanism of action investigation.

**Results:**

Selected results for **KM-408**: K_i_ sigma = 7.2*10^–8^; K_i_ 5-HT_1A_ = 8.0*10^–7^; ED_50_ MES (mice, *ip*) = 13.3 mg/kg; formalin test (I phase, mice, *ip*)—active at 30 mg/kg; SNL (rats, *ip*)—active at 6 mg/kg; STZ-induced pain (mice, *ip*)—active at 1 mg/kg (von Frey) and 10 mg/kg (hot plate); hot plate test (mice, *ip*)—active at 30 mg/kg; ED_50_ capsaicin test (mice, *ip*) = 18.99 mg/kg; tail immersion test (mice)—active at 0.5%; corneal anesthesia (guinea pigs)—active at 0.125%; infiltration anesthesia (guinea pigs)—active at 0.125%.

**Conclusions:**

Within the presented study a novel compound, *R,S*-2-((2-(2-chloro-6-methylphenoxy)ethyl)amino)butan-1-ol hydrochloride (**KM-408**) with dual antiseizure and analgesic activity has been developed for potential use in neuropathic pain treatment.

**Graphical abstract:**

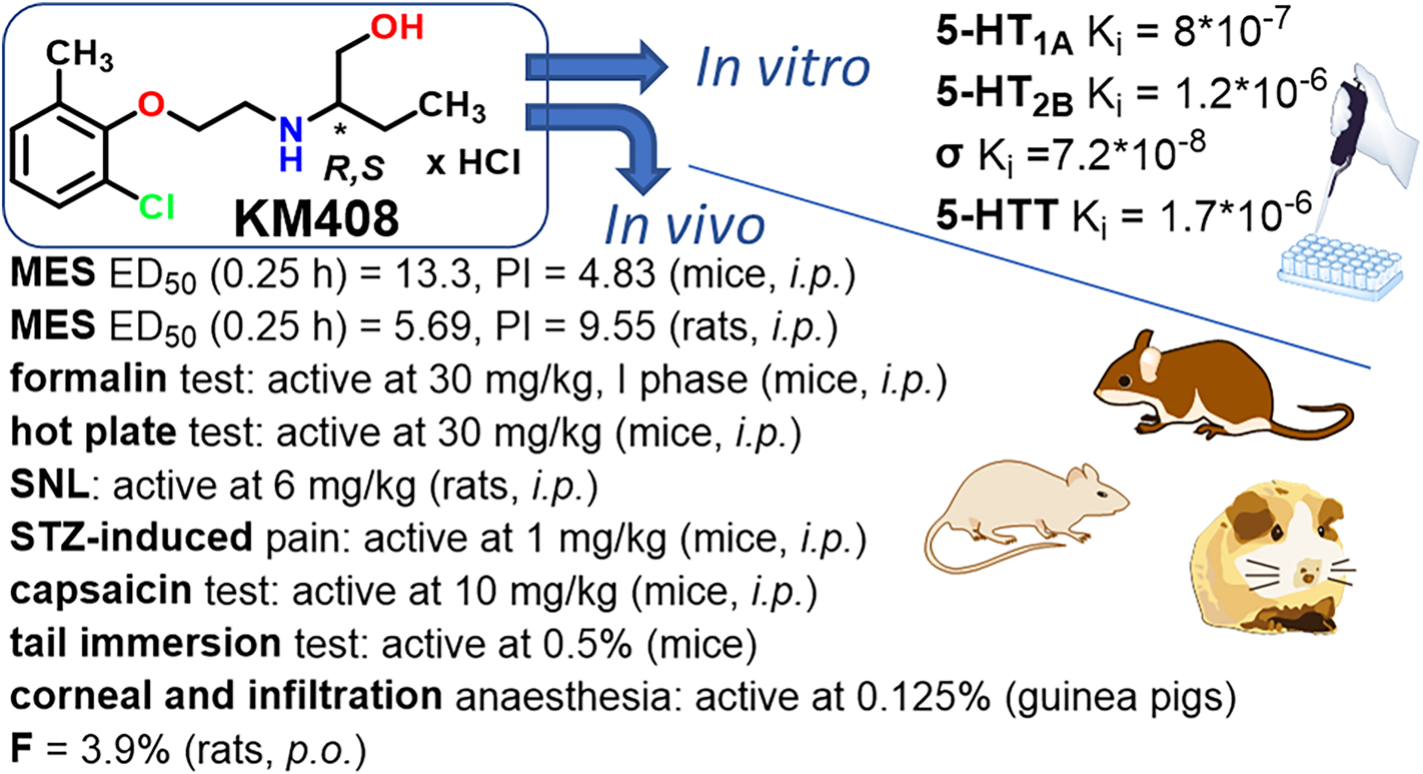

**Supplementary Information:**

The online version contains supplementary material available at 10.1007/s43440-022-00431-7.

## Introduction

Epilepsy and neuropathic pain are nervous system diseases  that considerably influence the quality of life and affect from 1% [1] to 7–10% [2] of the human world population. Therefore, their effective and safe treatment is of paramount importance.

Many antiepileptics have been used as drugs of first choice date back several decades. New drugs often seem promising until they happen to have a too narrow profile and are administered in a multidrug cocktail, enhancing effectiveness. Another problem is the frequent coexistence of epilepsy and other diseases of the nervous system (including neuropathic pain), which forces polypragmasy during therapy [3]. The use of many antiepileptic drugs also carries a risk of side effects, which may prevent dose escalation to a level that ensures satisfactory symptom control [4].

**Fig. 1:**
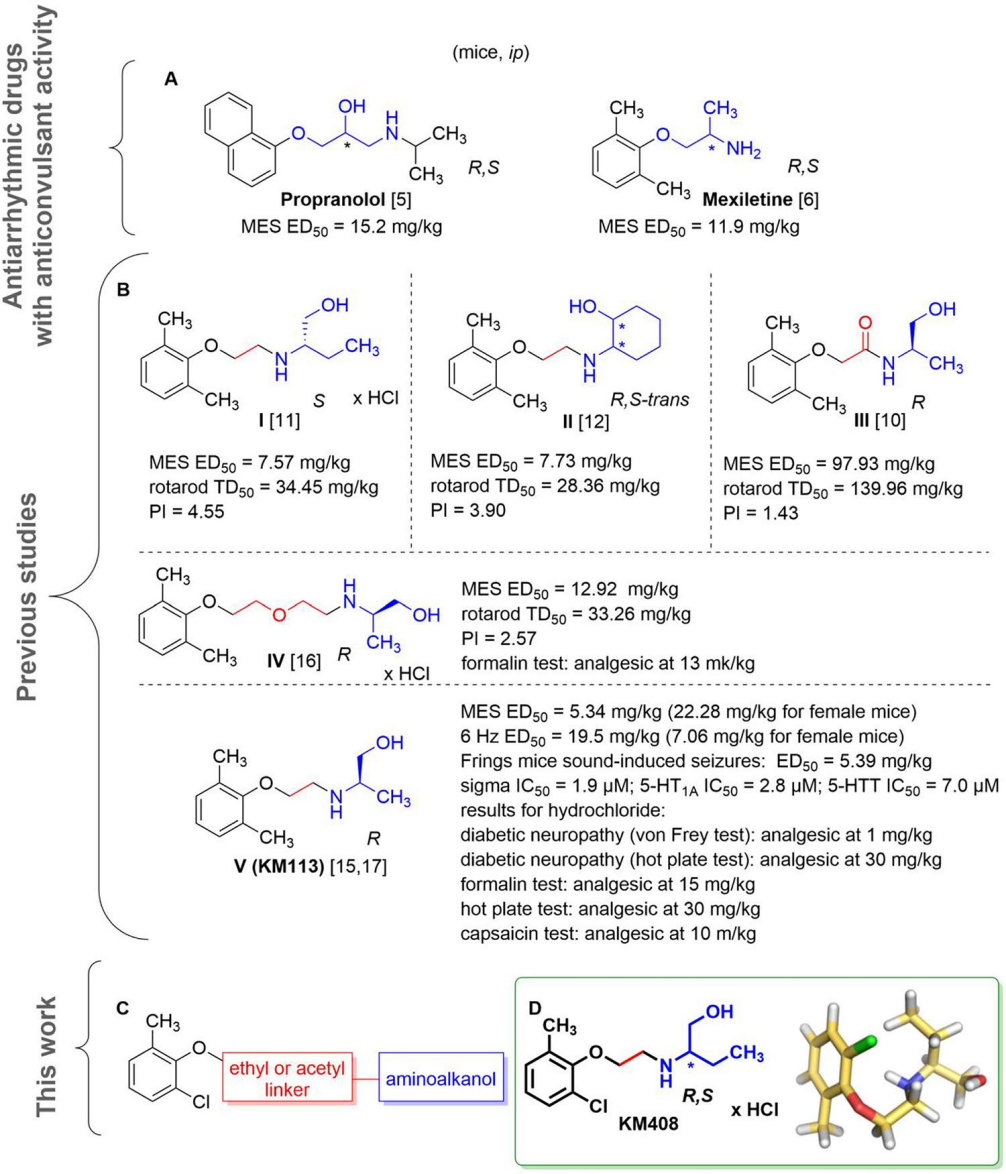
**A** Chemical structures and antiseizure activity of propranolol [5] and mexiletine [6] (antiarrhythmic drugs, the activity of which was a premise for undertaking research within the group of aminoalkanol derivatives); **B** Compounds previously published by our research group, which served as a starting point for the design of title compounds—**I** [11], **II** [12], **III** [10], **IV** [16] and **V** [15, 17]; **C** General structure of the designed compounds; **D** Chemical structure of compound **KM-408** (left) and lowest-ΔH_f_ conformation of its *R* enantiomer (base form) in aqueous solution, found using a two-step MMFF94—PM7/COSMO conformational search (right) [21]; the linkers used are highlighted in red and the aminoalkanol components in blue

The approach of our team is based on the search for active compounds with multitarget profiles beneficial in terms of potential side effects and on the implementation of screening for potential multidirectional central activity in vivo at the early stages of research. Such an approach enables both obtaining compounds with multidirectional activity that is beneficial in the treatment of comorbidities and the detection of the risk of potential side effects in the central nervous system at an early stage of drug development process. Chemically, research is focused on substituted phenoxyalkyl or phenoxyacetyl derivatives of amines, in particular aminoalkanols. Compounds with such a chemical structure may be considered as analogs of propranolol [5] and mexiletine [6]—antiarrhythmic drugs with additional antiseizure activity observed in animal models of seizures (Fig. 1A). These properties result from the mechanisms of action of these drugs targeting both the circulatory system and the central nervous system. The main mode of action of propranolol is through β-adrenergic antagonism, but its antiseizure activity is mediated by sodium channel blockade [5]. In turn, mexiletine is a non-selective sodium channel antagonist, which determines both its antiarrhythmic and antiseizure activities [6]. It is worth mentioning, however, that in the course of our research, we did not observe any significant affinity of the obtained compounds from this chemical group for beta-adrenergic receptors.

The experience we had previously gained ranged from the best compounds elaborated among xylenol derivatives with antiseizure and/or analgesic in neuropathic pain activity, including reference compounds **I**-**V** (Fig. 1B) [7–20]. Replacing the methyl substituent of xylenol with chlorine (Fig. 1C) resulted in a group of compounds with antiseizure activity in vivo, including *R,S*-2-{[2-(2-chloro-6-methylphenoxy)ethyl]amino}butan-1-ol hydrochloride (**KM-408**, Fig. 1D). **KM-408** is subject to intellectual property protection [20] and were chosen for further development as a potential antiseizure and analgesic compound for the treatment of neuropathic pain. Herein, we present **KM-408** properties in the context of other obtained 2-chloro-6-methylphenoxyethyl and 2-chloro-6-methylphenoxyacetyl derivatives of aminoalkanols.

## Materials and methods

### Chemistry

#### Synthesis and physicochemical characterization

*Reagents*. Enantiomeric 2-amino-1-butanol ([α]_546_^20^(*R*) = − 11.25º; [α]_546_^20^(*S*) =  + 11.15º) and *R,S*-1-amino-2-butanol were synthesized via previously published methods [22]. All other reagents were purchased from commercial suppliers (Lancaster, Aldrich) and used without further purification.

*Melting point*. Melting points (M.p.) were determined using a Būchi SMP-20 apparatus (Būchi Labortechnik) and are uncorrected.

*Thin layer chromatography (TLC)*. Kieselgel 60 F_254_ 0.25 mm precoated plates (Merck), UV light spots’ visualization.

*IR spectroscopy*. Recording of infrared (IR) spectra using compressed KBr pellets (1 mg sample: 300 mg KBr) was performed on a Jasco FT/IR 410 spectrometer.

*Nuclear magnetic resonance spectroscopy*. ^1^H NMR spectra were registered at Chair of Organic Chemistry, Faculty of Pharmacy, Jagiellonian University-Medical College (JEOL FT-NMR 500 MHz spectrometer—JNM-ECZR500 RS1 version ECZR and Varian Mercury-VX 300 NMR spectrometer) and Faculty of Chemistry, Jagiellonian University (Bruker AMX 500.13 MHz and 300 MHz spectrometers) with TMS as an internal standard. Chemical shifts were referenced against solvent lock signal. Results are presented in the following format: chemical shift δ (ppm), multiplicity, *J* values in Herts (Hz), number of protons, protons position. The following abbreviations were applied: cyclohex—cyclohexane, s—singlet, d—doublet, t—triplet, bs—broad singlet, m—multiplet, *J*—coupling constant. ^13^C NMR spectrum for compound **4** was registered at the Faculty of Chemistry (Jagiellonian University, Cracow, Poland) using Bruker AVANCE II 500, while for compound **15** at the Faculty of Pharmacy, Jagiellonian University-Medical College (JEOL FT-NMR 500 MHz spectrometer—JNM-ECZR500 RS1 version ECZR).

*Liquid chromatography–mass spectrometry (LCMS)*. The experiments were carried out as previously described [7].

*Optical rotation.* Measurement of optical rotation was carried out for 1% solutions in MeOH or CHCl_3_ using JASCO DIP-1000 (Nippon Bunko, Tokio, Japan) (*λ* = 589 nm) and Polamat A (Carl Zeiss, Jena, Germany) (*λ* = 546 nm).

*Optical purity.* Optical purity of compounds **5** and **6** was measured using an Agilent 1100 HPLC system (Agilent Technologies, Waldbronn, Germany) consisting of a degaser (G1322A), binary gradient pump (G1312A), thermostated autosampler (G1329A) and a DAD detector (G1315B). The experiments were carried out on a Chiralcel OD-RH analytical column (150 × 4.6 mm, 5 μM, Daicel Chemical Industries, Tokyo, Japan) at 20 °C. The autosampler temperature was also set at 20 °C. The mobile phase was an acetonitrile/water mixture (60:40, v/v) with the addition of 0.01% formic acid to improve ionization, at a flow rate of 0.35 mL/min. The volume of the sample applied to the chromatographic column was 50 μL.

The structures of compounds **5** and **6** were confirmed with a use of mass spectrometer with a tandem triple quadrupole mass analyzer API 2000 (Applied Biosystems MDS Sciex, Concord, Ontario, Canada) equipped with an electrospray ion source (ESI). ESI ionization was done in positive mode. Quantitative analysis was performed in the Multiple Reaction Monitoring (MRM) mode. The method was optimized by continuous application of the material to the mass spectrometer using a Harvard infusion pump (10 μL/min). Ion source parameters: ion source temperature (400 °C), voltage at the needle (4500 V), curtain gas (set to 6), collision gas (set to 10), collision energy (37 V). Ion path parameters: de-grouping potential (61 V), focus potential (360 V) input potential (12 V). Data collection and processing was performed with the Applied Biosystems Analyst 1.4 software.

*pK*_*a*_. The assay was performed by potentiometric titration, at 37 °C. The method is based on the additional portions of 0.051 M NaOH solution to the aqueous solution of test compounds, on electromagnetic stirrer with contact thermometer (Heidolph MR Hei-Standard). NaOH solution was added in 500 μL portions using Eppendorf automatic pipette. Waiting a few seconds after each titrant addition allowed the solution temperature to stabilize. After this time pH was recorded with the use of pH-meter CP-551 (Elmetron) for precise determination of pH in laboratory conditions (pH 0–14; pH accuracy ± 0.01) with combined chloride electrode OSH-10-10 (enables measurements in the temperature range 0–60 °C and pH range 1–12). Then, next portion of titrant was added. Titration was completed after 15 mL of NaOH solution was added. Threefold repetitions of titration were applied. p*K*_a_ of the tested compound was calculated on the basis of the pH values obtained.

*2-chloro-6-methyl(phenoxy)ethanol*. Solution of sodium ethanolate was prepared in a 750 mL round-bottomed flask (0.5 mol of sodium dissolved in 200 mL of ethanol), to which 0.5 mol of 2-chloro-6-methylphenol was added. The mixture was heated under reflux and in the boiling point, a solution of 2-bromoethanol was added dropwise for 3 h. Afterward, the mixture was heated for another 2 h and left to cool down. Precipitated white sediment (NaBr) was filtered off, and the filtrate was distilled into an oily residue. 200 mL of water and 10% solution of NaOH were added to the residue. Then extraction with benzene was performed, and the organic phase was additionally washed with 10% NaOH and water, and dried with anhydrous MgSO_4_. After distillation of the solvent, an oily residue was achieved. The crude product was used for further bromination.

*2-chloro-6-methyl(phenoxy)ethyl bromide*. 0.15 mol of 2-chloro-6-methyl(phenoxy)ethanol was put into a 100 mL round-bottomed flask and 0.05 mol of PBr_3_ was slowly added. The mixture was heated for 1.5 h under reflux in a water bath. Then the mixture was put into a flask with ice and neutralized with 15% NaHCO_3_, and afterward, extraction with benzene was performed. After drying the organic phase (anh. MgSO_4_), the solvent was distilled off, and the oily residue was achieved. The crude product was used for further aminolysis.

*General procedure for preparation of 2-chloro-[6-methyl(phenoxy)ethyl]aminoalkanols*. 0.012 mol of 2-chloro-6-methyl(phenoxy)ethyl bromide was put into a 100 mL round-bottomed flask. Then 0.012 mol of appropriate aminoalkanol and an excess of anh. K_2_CO_3_ was added. The mixture was heated in toluene under reflux for ca. 5 h and left to cool down. Afterward silica gel was added, and the mixture was heated again. The gel and precipitated KBr were filtered off and the remaining mixture were distilled into an oily residue. Then 10% HCl and active carbon was added, and the mixture was heated. Afterward, the suspension was filtered off and the filtrate was alkalized with 10% NaOH to precipitate the free basis, which was extracted with benzene. The organic phase was dried (anh. MgSO_4_), and the organic solvent was distilled off until the oily residue, which was crystallized. Some compounds were achieved in the form of hydrochlorides, using a saturated solution of HCl in ethanol or using gas HCl, and afterward performing crystallization from a mixture of ethyl acetate/EtOH (3:1).

*2-chloro-6-methyl(phenoxy)acetic acid*. Solution of 0.3 mol NaOH in 250 mL water was prepared in a 750 mL round-bottomed flask. Then 0.3 mol of 2-chloro-6-methylphenol was added. Separately, 0.3 mol of chloroacetic acid in 300 mL 10% NaHCO_3_ was prepared in a flask, and the mixture was added to the formerly prepared solution of sodium phenolate and heated refluxed for 1 h. Then active carbon was added, the mixture was filtered off from the suspension, and after cooling down the filtrate was acidified with 10% HCl. The precipitated acid, after filtering and drying, was crystallized from the mixture of heptane/toluene (1:1), and afterward white crystal precipitate was achieved.

*2-chloro-6-methyl(phenoxy)acetic acid chloride*. 0.15 mol of 2-chloro-6-methyl(phenoxy)acetic acid was put into a 500 mL round-bottomed flask and 0.75 mol of SOCl_2_ (d = 1.63 g/cm^3^) was added, and the mixture was heated under reflux for ca. 30 min. Afterward, an excess of thionyl chloride was distilled off under reduced pressure, and toluene was added to the remaining liquid acid chloride until 100 mL and the solution of the crude chloride was used for the reactions with an appropriate aminoalkanol.

*General procedure for preparation of 2-chloro-[6-methyl(phenoxy)acetyl]aminoalkanols*. 0.02 mol of appropriate aminoalkanol in 30 mL toluene was put in an Erlenmeyer flask, an excess of K_2_CO_3_ was added and dissolved in 50 mL of water. The mixture was cooled down and put on an electromagnetic stirrer. The mixture was added by small amounts of a solution of 2-chloro-6-methyl(phenoxy)acetic chloride in toluene, and the emulsion was left on the stirrer for ca. 0.5 h and afterward it was heated. After cooling the organic phase was separated and dried with anh. MgSO_4_. Then the solvent was distilled off, and the residue was crystallized into a white precipitate of the appropriate derivative. The synthesis of analogous amino acid derivatives has been described previously in detail [10].

##### ***R,S*-1-((2-(2-chloro-6-methylphenoxy)ethyl)amino)propan-2-ol (1)**

M.p. = 72–73 °C (toluene/heptane (1/1)); *R*_f_ = 0.54 (toluene/acetone (1/1)); ^1^H-NMR: (500 MHz, δ ppm, DMSO-d_6_) 7.24 (d, *J* = 7.753, 1H, Ar-H3), 7.14 (dq, *J* = 7.661, 0.692, 1H, Ar-H5), 6.99 (t, *J* = 7.646, 1H, Ar-H4), 4.45 (d, *J* = 4.296, 1H, OH), 3.88 (t, *J* = 5.585, 2H, Ar–O–CH_2_–), 3.58–3.72 (m, 1H, –CH <), 2.84–2.86 (m, 2H, –CH_2_–NH–CH_2_–), 2.44–2.47 (m, 4H, DMSO-d_6_/–CH_2_–NH–CH_2_–), 2.24 (s, 3H, Ar-CH_3_), 1.82–1.91 (m, 1H, NH), 1.01 (d, *J* = 6.301 Hz, 3H, > CH–CH_3_); C_12_H_18_NO_2_Cl (243.74); [%]: N^calc.^/_analyzed_: ^5.75^/_5.74_; C^calc.^/_analysed_: ^59.14^/_59.08_; H^calc.^/_analysed_: ^7.44^/_7.33_; LCMS [M + H]^+^ *m*/*z* calcd for C_12_H_18_NO_2_Cl 244.110, found 244.270, 100%.

##### ***R,S*-2-((2-(2-chloro-6-methylphenoxy)ethyl)amino)propan-1-ol (2)**

M.p. = 65–66 °C (toluene/heptane (1/1)); *R*_f_ = 0.50 (toluene/acetone (1/1)); ^1^H-NMR: (500.13 MHz, δ ppm, DMSO-d_6_) 7.28 (ddq, *J* = 7.9, *J* = 1.6, *J* = 0.6 1H, H-3); 7.18 (ddq, *J* = 7.5, *J* = 1.6, *J* = 0.8, 1H, H-5); 7.03 (ddq, *J* = 7.9, *J* = 7.5, *J* = 0.4 1H, H-4); 4.52 (dd, *J* = 5.0, *J* = 5.6 1H, OH); 3.97–3.89 (m 2H, ArO-CH_2_); 3.30 (ddd, *J* = 10.3, *J* = 5.1, *J* = 5.1 1H, CHH-OH); 3.23 (ddd, *J* = 10.3, *J* = 6.7, *J* = 5.6 1H, CHH-OH); 2.96–2.85 (m, 2H, CH_2_-NH); 2.71–2.64 (m, 1H, CH); 2.28 (ddd, *J* = 0.6, *J* = 0.8, *J* = 0.4, 3H, Ar-CH_3_); 1.94 (bs, 1H, NH); 0.93 (d, *J* = 6.3, 3H, CH_3_-R); C_12_H_18_NO_2_Cl (243.74) [%]: N^calc.^/_analyzed_: ^5.75^/_5.71_; C^calc.^/_analyzed_: ^59.14^/_59.05_ H^calc.^/_analyzed_: ^7.44^/_7.50_; LCMS [M + H]^+^ *m*/*z* calcd for C_12_H_18_NO_2_Cl 244.110, found 244.137, 100%.

##### ***R,S*-1-((2-(2-chloro-6-methylphenoxy)ethyl)amino)butan-2-ol (3)**

M.p. = 46–48 °C (toluene/heptane (1/1)); *R*_f_ = 0.62 (toluene/acetone (1/1)); ^1^H-NMR: (500.13 MHz, DMSO-d_6_, δ ppm) 7.28 (ddq, *J* = 8.0, *J* = 1.7, *J* = 0.7, 1H, H-3); 7.18 (ddq, *J* = 7.5, *J* = 1.6, *J* = 0.8, 1H, H-5); 7.03 (dd, *J* = 8.0, *J* = 7.5, 1H, H-4); 4.41 (d, *J* = 4.8, 1H, OH); 3.93 (t, *J* = 5.6, 2H, Ar-OCH_2_); 3.46–3.40 (m, 1H, CH); 2.90 (td, *J* = 5.6, *J* = 1.2, 2H, CH_2_N); 2.57 (dd, *J* = 11.7, *J* = 4.1, 1H, N–CHH); 2.48 (dd, *J* = 11.7, *J* = 7.6, 1H, C–HH); 2.28 (dd, *J* = 0.7, *J* = 0.8, 3H, CH_3_–Ar); 1.91 (bs, 1H, NH); 1.47–1.38 (m, 1H, CH_2_Et); 1.36–1.27 (m, 1H, CH_2_Et); 0.87 (bs, 1H, NH), C_13_H_20_NO_2_Cl (257.77), [%]: N^calc.^/_analyzed_: ^5.43^/_5.52_; C^calc.^/_analyzed_: ^60.58^/_60.63_ H^calc.^/_analyzed_: ^7.82^/_7.54_; LCMS [M + H]^+^ m/z calcd for C_13_H_20_NO_2_Cl 258.126, found 258.158, 100%.

##### ***R,S*-2-((2-(2-chloro-6-methylphenoxy)ethyl)amino)butan-1-ol (4)**

M.p. = 68–69 °C (toluene/heptane (1/1)); *R*_f_ = 0.56 (toluene/acetone (1/1)); *R*_f_ = 0.45 (CH_3_OH/ethyl acetate (1/3)); ^1^H-NMR: (500 MHz, DMSO-d_6_, δ ppm) 7.24 (d, *J* = 7.763, 1H, Ar–H3), 7.14 (dq, *J* = 7.661, 0.692, 1H, Ar–H5), 6.99 (t, *J* = 7.733, 1H, Ar–H4), 4.40–4.44 (m, 1H, OH), 3.88 (t, *J* = 5.585, 2H, Ar–O–CH_2_–), 3.33–3.39 (m, 1H, –CHH–OH), 3.24 (dt, *J* = 10.811, 5.620, 1H, –CHH–OH), 2.82–2.92 (m, 2H, –CH_2_–NH–), 2.39–2.44 (m, 1H, > CH–), 2.24 (s, 3H, Ar–CH_3_), 1.80–1.86 (m, 1H, NH), 1.29–1.36 (m, 2H, –CH_2_–CH_3_), 0.81 (t, *J* = 7.446, 3H, –CH_2_–CH_3_); ^13^C-NMR: (125.77 MHz, DMSO-d_6_, δ ppm) 152.85 (C-1); 133.09 (C-6); 129.89 (C-5); 127.68 (C-3); 126.67 (C-2); 124.72 (C-4); 72.82 (C-8); 62.64 (C-10); 60.14 (C-11); 46.57 (C-9); 23.64 (C-12); 16.05 (C-7); 9.92 (C13), C_13_H_20_NO_2_Cl (257.77), [%]: N^calc.^/_analyzed_: ^5.43^/_5.47_; C^calc.^/_analyzed_: ^60.58^/_60.59_; H^calc.^/_analyzed_: ^7.82^/_7.65_; LCMS [M + H]^+^ *m*/*z* calcd for C_13_H_20_NO_2_Cl 258.12, found 258.23, 100%.

##### ***R,S*-2-((2-(2-chloro-6-methylphenoxy)ethyl)amino)butan-1-ol hydrochloride (4a, KM-408)**

M.p. = 109–111 °C; R_f_ = 0.68 (CH_3_OH); *R*_f_ = 0.59 (CH_3_OH/ethyl acetate (1/1)); ^1^H-NMR: (500 MHz, DMSO-d_6_, δ ppm) 9.12 (bs, 1H, NHH^+^), 8.90 (bs, 1H, NHH^+^), 7.29 (dt, *J* = 7.947, 0.752, 1H, Ar-H3), 7.18 (d, *J* = 7.373, 1H, Ar-H5), 7.05 (t, *J* = 7.724, 1H, Ar-H4), 5.37 (t, *J* = 5.155, 1H, OH), 4.18 (t, *J* = 5.728, 2H, Ar–O–CH_2_–), 3.74 (dt, *J* = 12.458, 3.795, 1H, –CHH–OH), 3.59 (dt, *J* = 12.315, 5.155, 1H, –CHH–OH), 3.33–3.41 (m, 2H, –CH_2_–NH–), 3.13 (bs, 1H, > CH–), 2.29 (s, 3H, Ar–CH_3_), 1.58–1.78 (m, 2H, –CH_2_–CH_3_), 0.90 (t, *J* = 7.446, 3H, –CH_2_–CH_3_); C_13_H_21_NO_2_Cl_2_ (294.22), [%]: N^calc.^/_analyzed_: ^4.76^/_4.72_; C^calc.^/_analyzed_: ^53.06^/_52.80_ H^calc.^/_analyzed_: ^7.20^/_7.60_; LCMS [M + H]^+^ *m*/*z* calcd for C_13_H_21_NO_2_Cl_2_ 258.126, found 258.23, 100%, p*K*_a_ = 9.501 (± 0.971).

##### ***R*-(-)-2-((2-(2-chloro-6-methylphenoxy)ethyl)amino)butan-1-ol (5)**

M.p. = 66–68 °C (toluene/heptane (1/1)); *R*_f_ = 0.56 (toluene/acetone (1/1)); *R*_f_ = 0.45 (CH_3_OH/ethyl acetate (1/3)); ^1^H-NMR: (DMSO-d_6_, 500 MHz, δ ppm) 7.24 (dd, *J* = 8.019, *J* = 1.146, 1H, Ar-H3), 7.14 (d, *J* = 7.481, 1H, Ar-H5), 6.96–7.02 (m, 1H, Ar-H4), 4.42 (t, *J* = 5.298, 1H, OH), 3.88 (t, *J* = 5.728, 2H, Ar–O–CH_2_–), 3.36 (dt, *J* = 10.382, *J* = 4.976, 1H, –CHH–OH), 3.20–3.27 (m, 1H, –CHH–OH), 2.81–2.92 (m, 2H, –CH_2_–NH–), 2.38–2.44 (m, 1H, > CH–), 2.24 (s, 3H, Ar–CH_3_), 1.79–1.85 (m, 1H, NH), 1.28–1.38 (m, 2H, –CH_2_–CH_3_), 0.81 (t, *J* = 7.446, 3H, –CH_2_–CH_3_); C_13_H_20_NO_2_Cl (257.77), [%]: N^calc.^/_analyzed_: ^5.43^/_5.40_; C^calc.^/_analyzed_: ^60.58^/_60.26_ H^calc.^/_analyzed_: ^7.82^/_7.77_; (c = 1%, CHCl_3_): [α]_546_^24.1=^- 22.0º, [α]_589_^22.7=^- 20.94º, ee = 100%; LCMS [M + H]^+^ *m*/*z* calcd for C_13_H_20_NO_2_Cl 258.12, found 258.192, 100%.

##### ***R*-(-)-2-((2-(2-chloro-6-methylphenoxy)ethyl)amino)butan-1-ol hydrochloride (5a)**

M.p. = 113–115 °C; *R*_f_ = 0.60 (CH_3_OH/ethyl acetate (1/1)); ^1^H-NMR: (DMSO-d_6_, 500 MHz, δ ppm) 8.71–8.97 (m, 2H, NH_2_^+^), 7.29 (dt, *J* = 8.019, *J* = 0.859, 1H, Ar-H3), 7.19 (dq, *J* = 7.661, *J* = 0.692, 1H, Ar-H5), 7.03–7.08 (m, 1H, Ar-H3), 5.36 (t, *J* = 4.869, 1H, OH), 4.15 (t, *J* = 5.728, 2H, Ar–O–CH_2_–), 3.70–3.78 (m, 1H, CHH–OH), 3.58 (dt, *J* = 12.243, *J* = 5.048, 1H, –CHH–OH), 3.38 (bs, 2H, –CH_2_–NH_2_^+^–), 3.10–3.19 (m, 1H, > CH–), 2.28 (s, 3H, Ar–CH_3_), 1.55–1.79 (m, 2H, –CH_2_–CH_3_), 0.90 (t, *J* = 7.589, 3H, –CH_2_–CH_3_); C_13_H_21_NO_2_Cl_2_ (294.22), [%]: N^calc.^/_analyzed_: ^4.76^/_4.75_; C^calc.^/_analyzed_: ^53.06^/_52.83_ H^calc.^/_analyzed_: ^7.20^/_7.19_; (c = 1%, CH_3_OH): [α]_589_^23.3=^- 1.6º; LCMS [M + H]^+^ *m*/*z* calcd for C_13_H_21_NO_2_Cl_2_ 258.126, found 258.225, 100%.

##### ***S*-( +)-2-((2-(2-chloro-6-methylphenoxy)ethyl)amino)butan-1-ol (6)**

M.p. = 66–68 °C (toluene/heptane (1/1)); *R*_f_ = 0.56 (toluene/acetone (1/1)); *R*_f_ = 0.45 (CH_3_OH/ethyl acetate (1/3)); ^1^H-NMR: (DMSO-d_6_, 500.13 MHz, δ ppm) 7.28 (dd, *J* = 7.9, *J* = 1.7, 1H, H-3(Ar)); 7.18 (dd, *J* = 7.6, *J* = 1.7, 1H, H-5(Ar)); 7.03 (dd, *J* = 7.9, *J* = 7.6, 1H, H-4(Ar)); 4.47 (bs, 1H, OH); 3.93 (t, *J* = 5.7, 2H, Ar–O–CH_2_); 3.41 (dd, *J* = 4.9, *J* = 10.6, 1H, CHH–OH); 3.28 (dd, *J* = 6.5, *J* = 10.6, 1H, CHH–OH); 2.96–2.87 (m, 2H, –CH_2_–N); 2.49–2.42 (m, 1H, NH–CH(C_2_H_5_)–CH_2_OH); 2.28 (s, 3H, CH_3_–Ar); 1.96 (bs, 1H, NH); 1.44–1.31 (m, 2H, –CH_2_–CH_3_); 0.86 (t, *J* = 7.6, 3H, –CH_2_–CH_3_); (c = 1%, CHCl_3_): [α]_546_^24.1=^ + 21.00º; [α]_589_^22.7=^ + 21.04º, ee = 100%; LCMS [M + H]^+^ *m*/*z* calcd for C_13_H_20_NO_2_Cl 258.12, found 258.23, 97.32%.

##### ***S*-( +)-2-((2-(2-chloro-6-methylphenoxy)ethyl)amino)butan-1-ol hydrochloride (6a)**

M.p. = 113–115 °C; *R*_f_ = 0.60 (CH_3_OH/ethyl acetate (1/1)); ^1^H-NMR: (500 MHz, DMSO-d_6_, δ ppm) 9.12 (bs, 1H, NHH^+^), 8.90 (bs, 1H, NHH^+^), 7.26–7.32 (m, 1H, Ar–H3), 7.16–7.21 (m, 1H, Ar–H5), 7.05 (t, *J* = 7.734, 1H, Ar–H4), 5.37 (t, *J* = 5.012, 1H, OH), 4.18 (t, *J* = 5.871 Hz, 2H, Ar–O–CH_2_–), 3.74 (dt, *J* = 12.601, 3.866, 1H, –CHH–OH), 3.59 (dt, *J* = 12.243, 5.048, 1H, –CHH–OH), 3.33–3.41 (m, 2H, –CH_2_–NH–), 3.13 (bs, 1H, > CH–), 2.28 (s, 3H, Ar–CH_3_), 1.58–1.78 (m, 2H, –CH_2_–CH_3_), 0.90 (t, *J* = 7.446, 3H, –CH_2_–CH_3_); C_13_H_21_NO_2_Cl_2_ (294.22), [%]: N^calc.^/_analyzed_: ^4.76^/_4.74_; C^calc.^/_analyzed_: ^53.06^/_52.97_ H^calc.^/_analyzed_: ^7.20^/_7.25_; (c = 1%, CH_3_OH): [α]_589_^23.3=^ + 1.4º; LCMS [M + H]^+^ *m*/*z* calcd for C_13_H_21_NO_2_Cl_2_ 258.12, found 258.23, 100%.

##### **2-((2-(2-chloro-6-methylphenoxy)ethyl)amino)-2-methylpropane-1,3-diol (7)**

M.p. = 83–85 °C (toluene/heptane (1/1)); *R*_f_ = 0.52 (CH_3_OH/ethyl acetate (1/1)); CDCl_3_, 300 MHz, δ ppm) 6.92–7.22 (m, 3H, Ar–H), 4.03 (t, *J* = 5.1, 2H, –O–CH_2_–CH_2_–NH–), 3.55 (s, 4H, (–CH_2_–OH)_2_), 2.99 (t, *J* = 5.1, 2H, –O–CH_2_–CH_2_–NH–), 2.50 (bs, 2H, 2x –OH), 2.32 (s, 3H, Ar–CH_3_), 2.10 (bs, 1H, NH), 1.06 (s, 3H, –C–CH_3_); C_13_H_20_NO_3_Cl (273.76), [%]: N^calc.^/_analyzed_: ^5.12^/_5.12_; C^calc.^/_analyzed_: ^57.04^/_57.28_; H^calc.^/_analyzed_: ^7.36^/_7.46_; LCMS [M + H]^+^ *m*/*z* calcd for C_13_H_20_NO_3_Cl 274.120, found 274.106, 96.552%.

##### ***R,S-trans*-2-((2-(2-chloro-6-methylphenoxy)ethyl)amino)cyclohexan-1-ol (8)**

M.p. = 73–75 °C (toluene/heptane (1/1)); *R*_f_ = 0.48 (CH_3_OH); ^1^H-NMR: (DMSO-d_6_, 500 MHz, δ ppm) 7.24 (dd, *J* = 8.019, 1.146, 1H, Ar–H3), 7.14 (d, *J* = 7.277, 1H, Ar–H5), 6.99 (t, *J* = 7.609, 1H, Ar–H4), 4.57 (d, *J* = 5.155, 1H, OH), 3.84–3.97 (m, 2H, Ar–O–CH_2_–), 3.07 (tt, *J* = 9.343, 4.833, 1H, > CH–OH), 2.75–2.93 (m, 2H, –CH_2_–NH–), 2.30 (bs, 1H, NH), 2.24 (s, 3H, Ar–CH_3_), 2.14–2.23 (m, 1H, > CH–NH-), 1.83–1.91 (m, 1H, cyclohex), 1.69–1.81 (m, 1H, cyclohex), 1.52–1.61 (m, 2H, cyclohex), 1.00–1.25 (m, 3H, cyclohex), 0.80–0.96 (m, 1H, cyclohex); C_15_H_22_NO_2_Cl (283.80), [%]: N^calc.^/_analysed_: ^4.94^/_4.93_; C^calc.^/_analysed_: ^63.48^/_63.38_ H^calc.^/_analysed_: ^7.81^/_7.79_; LCMS [M + H]^+^ *m*/*z* calcd for C_15_H_22_NO_2_Cl 284.141, found 284.140, 100%.

##### ***R,S*-2-((2-(2-chloro-6-methylphenoxy)ethyl)amino)-1-phenylethan-1-oll (9)**

M.p. = 98–100 °C (toluene/heptane (1/1)); *R*_f_ = 0.60 (CH_3_OH); ^1^H-NMR: (CDCl_3_, 300 MHz, δ ppm) 7.42–6.92 (m, 8H, H–Ar); 4.76 (dd, *J* = 3.9, *J* = 8.9, 1H, Ar–CHOH–CH_2_); 4.07–3.98 (m, 2H, –O–CH_2_–CH_2_–NH); 3.17–3.02 (m, 3H, O–CH_2_–CHH–NH, NH–CH_2_–CHOH); 2.80 (dd, *J* = 8.7, *J* = 12.1, 1H, O–CH_2_–CHH–NH); 2.30 (s, 3H, CH_3_–Ar); 1.40 (bs, 1H, OH), C_17_H_20_NO_2_Cl (305.81), [%]: N^calc.^/_analysed_: ^4.58^/_4.50_; C^calc.^/_analysed_: ^66.71^/_66.59_; H^calc.^/_analysed_: ^6.59^/_6.57_, LCMS [M + H]^+^ *m*/*z* calcd for C_17_H_20_NO_2_Cl 306.126, found 306.269, 100%.

##### ***R,S*-2-(2-chloro-6-methylphenoxy)-N-(2-hydroxypropyl)acetamide (10)**

M.p. = 102–104 °C; *R*_f_ = 0.82 (CH_3_OH/ethyl acetate (1/1)); 1H-NMR: (CDCl_3,_ 300 MHz, δ ppm) 7.38 (bs, 1H, NH); 7.26–6.97 (m, 3H, H–Ar); 4.43 (s, 2H, O–CH_2_–CO); 4.08–3.98 (m, 1H, –CH–OH); 3.62–3.54 (m, 1H, NH–CHH); 3.34–3.25 (m, 1H, NH–CHH); 2.51 (bs, 1H, OH); 2.31 (s, 3H, CH_3_–Ar); 1.25 (d, *J* = 6.4, 3H, CH–CH_3_), C_12_H_16_NO_3_Cl (257.72), [%]: N^calc.^/_analyzed_: ^5.43^/_5.12_; C^calc.^/_analyzed_: ^55.93^/_56.19;_ H^calc.^/_analyzed_: ^6.26^/_6.32_.

##### ***R,S*-2-(2-chloro-6-methylphenoxy)-N-(1-hydroxypropan-2-yl)acetamide (11)**

M.p. = 88–90 °C; *R*_f_ = 0.87 (CH_3_OH/ethyl acetate (1/1)); ^1^H-NMR: (DMSO-d_6,_ 500.13 MHz, δ ppm) 7.76 (d, *J* = 8.5, 1H, NH); 7.31 (dd, *J* = 8.0, *J* = 1.8, 1H, H-3); 7.21 (dd, *J* = 7.6, *J* = 1.8, 1H, H-5); 7.07 (dd, *J* = 8.0, *J* = 7.6, 1H, H-4); 4.78 (bs, 1H, OH); 4.35 (d, *J* = 14.0, 1H, CHH–O–Ar); 4.28 (d, *J* = 14.0, 1H, CHH–O–Ar); 4.04–3.82 (m, 1H, CH); 3.48–3.33 (m, 2H, CH_2_–OH); 2.30 (s, 3H, Ar–CH_3_); 1.10 (d, *J* = 6.7, 3H, –CH_3_), C_12_H_16_NO_3_Cl (257.72), [%]: N^calc.^/_analyzed_: ^5.43^/_5.35_; C^calc.^/_analyzed_: ^55.93^/_56.19;_ H^calc.^/_analyzed_: ^6.26^/_6.39_; LCMS [M + H]^+^ m/z calcd for C_12_H_16_NO_3_Cl 258.089, found 258.225, 100%.

##### ***R,S*-2-(2-chloro-6-methylphenoxy)-N-(1-hydroxybutan-2-yl)acetamide (12)**

M.p. = 59–60 °C; *R*_f_ = 0.84 (CH_3_OH/ethyl acetate (1/1)); IR (KBr, cm^−1^) *v*: 3278, 3075, 2966, 2934, 2875, 1655, 1547, 1263, 1047; ^1^H-NMR: (DMSO-d_6,_ 500.13 MHz, δ ppm) 7.67 (d, *J* = 8.7, 1H, NH); 7.31 (dd, *J* = 8.2, *J* = 1.7, 1H, H-3); 7.21 (dd, *J* = 7.6, *J* = 1.7, 1H, H-5); 7.08 (dd, *J* = 8.0, *J* = 7.6, 1H, H-4); 4.74 (t, *J* = 5.5, 1H, OH); 4.38 (d, *J* = 14.1, 1H, O–CH–HC=O); 4.31 (d, *J* = 14.1, 1H, O–CHHC=O); 3.80–3.72 (m, 1H, CH); 3.51–3.32 (m, 2H, CH_2_–OH); 2.30 (s, 3H, Ar–CH_3_); 1.62 (qdd, *J* = 18.9, *J* = 7.4, *J* = 5.2, 1H, CHH(Et)); 1.43 (qdd, *J* = 18.9, *J* = 8.6, *J* = 7.4, 1H, CHH(Et)); 0.87 (t, *J* = 7.4, 3H, –CH_3_), C_13_H_18_NO_3_Cl (271.75), [%]: N^calc.^/_analyzed_: ^5.15^/_5.15_; C^calc.^/_analyzed_: ^57.46^/_57.79;_ H^calc.^/_analyzed_: ^6.68^/_7.25_; LCMS [M + H]^+^ m/z calcd for C_13_H_18_NO_3_Cl 272.105, found 272.312, 100%.

##### ***R,S-*(2-(2-chloro-6-methylphenoxy)acetyl)alanine (13)**

M.p. = 199–201 °C; *R*_f_ = 0.86 (CH_3_OH/ethyl acetate (1/1)); IR (KBr, cm^−1^) *v*: 3364, 2984, 2923, 2750, 2616, 2544, 1859, 1727, 1631, 1421, 1247, 1221; ^1^H-NMR: (CDCl_3,_ 300 MHz, δ ppm) 7.50 (bs, 1H, CO–NH); 7.25–6.98 (m, 3H, H–Ar); 4.80–4.70 (m, 1H, NH–CH–CH_3_); 4.46 (dd, *J* = 14.9, *J* = 20.3, 2H, O–CH_2_–CO); 2.32 (s, 3H, CH_3_–Ar); 1.58 (d, *J* = 7.2, 3H, CH–CH_3_), C_12_H_14_NO_4_Cl (271.70), [%]: N^calc.^/_analyzed_: ^5.16^/_5.12_; C^calc.^/_analyzed_: ^53.04^/_53.17;_ H^calc.^/_analyzed_: ^5.19^/_5.37_; LCMS [M + H]^+^ *m*/*z* calcd for C_12_H_14_NO_4_Cl 272.068, found 272.245, 100%.

##### **R-(+)-(2-(2-chloro-6-methylphenoxy)acetyl)alanine (14)**

M.p. = 186–188 °C; *R*_f_ = 0.53 (CH_3_OH/ethyl acetate (1/1)); ^1^H-NMR: (DMSO-d_6,_ 500 MHz, δ ppm) 12.66 (bs, 1H, COOH), 8.24 (d, *J* = 7.446, 1H, NH), 7.28 (dt, *J* = 7.947, 0.752, 1H, Ar–H3), 7.17 (d, *J* = 7.519, 1H, Ar–H5), 7.04 (t, *J* = 7.745, 1H, Ar–H4), 4.27–4.38 (m, 3H, Ar–O–CH_2_–, > CH–), 2.27 (s, 3H, Ar–CH_3_), 1.31 (d, *J* = 7.160, 3H, > CH–CH_3_); C_12_H_14_NO_4_Cl (271.70), [%]: N^calc.^/_analysed_: ^5.16^/_5.11_; C^calc.^/_analysed_: ^53.04^/_53.23;_ H^calc.^/_analysed_: ^5.19^/_5.43_; (c = 1%, CH_3_OH): [α]_546_^19.5=^ + 9.40°; [α]_589_^19.1=^ + 8.48°; LCMS [M + H]^+^ *m*/*z* calcd for C_12_H_14_NO_4_Cl 272.068, found 272.179, 100%.

##### **S-(-)-(2-(2-chloro-6-methylphenoxy)acetyl)alanine (15)**

M.p. = 186–188 °C; *R*_f_ = 0.53 (CH_3_OH/ethyl acetate (1/1)); ^1^H-NMR: (DMSO-d_6,_ 500.13 MHz, δ ppm) 12.59–12.76 (m, 1H, COOH), 8.17–8.31 (m, 1H, NH), 7.23–7.31 (m, 1H, Ar–H4), 7.13–7.20 (m, 1H, Ar–H3), 7.01–7.06 (m, 1H, Ar–H5), 4.33–4.36 (m, 2H, Ar–O–CH_2_–), 4.32 (s, 1H, –NH–CH <), 2.23–2.30 (m, 3H, Ar–CH_3_), 1.27–1.36 (m, 3H > CH–CH_3_); ^13^C-NMR: (DMSO-d_6,_ 126 MHz, δ ppm) 174.26 (s, HO–C=O), 167.53 (s, –HN–C=O), 152.63 (s, Ar–C–O–), 133.87 (s, Ar–C6), 130.64 (s, Ar–C5), 128.35 (s, Ar–C3), 127.03 (s, Ar–C2), 126.02 (s, Ar–C4), 71.28 (s, –O–CH_2_–), 47.76 (s, > CH–CH_3_), 17.76 (s, > CH–CH_3_), 16.68 (s, Ar–CH_3_); (c = 1%, CH_3_OH): [α]_546_^19.5=^− 9.40°; [α]_589_^19.1=^− 9.10°; LCMS [M + H]^+^ *m*/*z* calcd for C_12_H_14_NO_4_Cl 272.068, found 272.179, 100%.

##### **R,S-2-(2-(2-chloro-6-methylphenoxy)acetamido)butanoic acid (16)**

M.p. = 172–174 °C; *R*_f_ = 0.62 (CH_3_OH/ethyl acetate (1/1)); IR (KBr, cm^−1^) *v*: 3372, 2978, 2925, 2880, 2747, 2548, 1727, 1633, 1241, 1047, 777; ^1^H-NMR: (DMSO-d_6,_ 500.13 MHz, δ ppm) 8.11 (d, *J* = 7.8, 1H, NH); 7.31 (dd, *J* = 7.8, *J* = 1.7, 1H, H-3); 7.21 (dd, *J* = 7.6, *J* = 1.7, 1H, H-5); 7.08 (dd, *J* = 7.8, *J* = 7.6, 1H, H-4); 4.44 (d, *J* = 14.0, 1H, CHH–O–Ar); 4.37 (d, *J* = 14.0, 1H, CHH–O–Ar); 4.32–4.22 (m, 1H, CH); 2.31 (s, 3H, Ar–CH_3_); 1.94–1.64 (m, 2H, –CH_2_–CH_3_); 0.90 (t, *J* = 7.3, 3H, CH_2_–CH_3_), C_13_H_16_NO_4_Cl (285.73), [%]: N^calc.^/_analyzed_: ^4.90^/_4.87_; C^calc.^/_analyzed_: ^54.65^/_54.70;_ H^calc.^/_analyzed_: ^5.64^/_5.69_; LCMS [M + H]^+^ *m*/*z* calcd for C_13_H_16_NO_4_Cl 286.084, found 286.200, 100%.

##### ***Trans*-2-(2-chloro-6-methylphenoxy)-N-(4-hydroxycyclohexyl)acetamide (18)**

M.p. = 134–136 °C; *R*_f_ = 0.50 (toluene/acetone (1/1)); ^1^H-NMR: (300 MHz, CDCl_3_, δ ppm) 7.25–7.20 (m, 1H, Ar–H3); 7.12–7.07 (m, 1H, Ar–H5); 7.03–6.96 (m, 1H, Ar–H4); 6.84 (d, *J* = 6.92, 1H, –CO–NH–); 4.37 (s, 2H, –O–CH_2_–); 3.99–3.81 (m, 1H, –CH–OH); 3.74–3.58 (m, 1H, CH–NH); 2.30 (s, 3H, CH_3_Ar); 2.16–1.97 (m, 4H, cyclohex); 1.54–1.26 (m, 4H, cyclohex); C_15_H_20_O_3_NCl (297.78), LCMS [M + H] + *m*/*z* calcd for C_15_H_20_O_3_NCl 298.12, found 298.18, 95.99%.

#### Forced degradation studies

*HPLC conditions and equipment.* The liquid chromatography system HITACHI High-Technologies Corporation (Tokyo, Japan) equipped with a solvent delivery pump (L-2130), a degasser, an autosampler (L-2200), a photodiode array detector (L-2455) and a column oven (L-2350) was used. The chromatographic analysis of **KM-408** was performed on a Fusion RP-18 column (150 mm × 4.6 mm; particle size 4 µm) from Phenomenex with isocratic elution using a mobile phase composed of ammonium acetate (0.15 M, pH 4.4) and methanol (45:55, v/v) at a flow rate of 1.1 mL min^−1^ at 25 °C. The injection volume was 20 µL. Based on the registered absorption spectrum detection wavelength was selected as 230 nm. The analysis time was 15 min.

*Irradiation conditions.* Irradiation was conducted in a climatic chamber KBF-ICH 240 APT.lineTM (Binder GmbH, Tuttlingen, Germany) at 25 °C and 60% humidity using UVA radiation (320–400 nm) with maximum emission at 365 nm. The intensity of radiation was determined using a radiometer type VLX-3 W, Vilber Lourmat, with a CX-365 sensor, to be each time of 0.25 mW m^−2^. The distance between the samples and the radiation source was 13 cm.

*Degradation in acidic solution.* For acidic degradation, about 100 mg of **KM-408** was accurately weighed, transferred to a volumetric flask of 10 mL capacity and dissolved in 0.5 M hydrochloric acid. 8 mL of the examined solution was transferred to a glass vial and heated in an oven at 70 °C. The sample volume of 0.5 mL was taken and diluted with 0.5 mL of water. The solution was analyzed three times by HPLC method.

*Degradation in basic solution.* For basic degradation, 100 mg of **KM-408** was accurately weighed, transferred to a volumetric flask of 10 mL capacity and dissolved in 0.5 M NaOH. 4 mL of examined solution was transferred to glass vials of 8 mL capacity and heated in an oven at 70 °C. The volume of 0.5 mL was taken and diluted with 0.5 mL of 0.5 M HCl. The solution was analyzed three times by HPLC method.

*Degradation in phosphate buffer at pH* = *7.0*. 100 mg of **KM-408** was weighed, transferred to a volumetric flask of 10 mL capacity and dissolved in phosphate buffer at pH = 7.0. The volume of 8 mL of examined solution was transferred to glass vials of 8 mL capacity and stored in a dark place at 25 °C or heated in an oven at 70 °C. The volume of 0.5 mL of solution was taken and diluted with 0.5 mL of water. The solution was analyzed by HPLC method.

*Degradation in the presence of oxidative agent.* 100 mg of **KM-408** was weighed, transferred to a volumetric flask of 10 mL capacity and filled up to a volume with 1.5% H_2_O_2_. The samples were stored at 25 °C. The volume of 0.5 mL of solution was taken and diluted with 0.5 mL of water. The solution was analyzed three times by HPLC method. 100 mg of **KM-408** was weighed, transferred to a volumetric flask of 10 mL capacity and filled up to a volume with 0.001 M CuSO_4_. The volume of 8 mL of examined solution was transferred to glass vials of 8 mL capacity and heated in an oven at 40 °C. The volume of 0.5 mL of solution was taken and diluted with 0.5 mL of water. The solution was analyzed by HPLC method.

*Degradation in the presence of reducing agent.* 100 mg of **KM-408** was weighed, transferred to a volumetric flask of 10 mL capacity and filled up to a volume with 0.005 M Na_2_S_2_O_3_. 8 mL of the examined solution was transferred to 8 mL glass vials and stored at 25 °C and 40 °C. The volume of 0.5 mL of solution was taken and diluted with 0.5 mL of water. The solution was analyzed three times by HPLC method.

*Photodegradation under UV irradiation.* 25 mg of **KM-408** was weighed, transferred to a volumetric flask of 25 mL capacity and filled up to a volume with methanol. 2 mL of the examined solution was transferred to a quartz Petri-dishes with diameter 4 cm and sealed with parafilm. Dark control samples were prepared in the same way, but they were secured with aluminum foil. The examined samples and the dark control samples were placed in a climatic chamber at 20 °C and humidity 60% and irradiated for 96 h. The volume of 0.5 mL of solution was taken to the vials and the solution was analyzed by HPLC method.

*Kinetic studies under basic conditions*

*Chromatographic conditions.* The liquid chromatography system Merck-Hitachi LaChrom Elite, equipped with a LiChroSpher RP-18 (250 × 4.6 mm; particle size 5 µm) column from Merck was used. Isocratic elution using a mobile phase composed of phosphate buffer pH = 4.36 and acetonitrile (40:60, v/v) at a flow rate of 1 mL min^−1^ at 25 °C and single-wavelength UV detection at 260 nm was applied for the analysis. The injection volume was 20 µL. The analysis time was 18.5 min.

*Kinetic studies. ***KM-408** (0.125 g) was dissolved in dioxane (5 ml) and added to pH = 9.9, ionic strength 0.5 borate buffer (20 mL) heated to 70 °C in a reaction apparatus. After 5 min, first 50 µL sample was taken using a Hamilton syringe. The sample was diluted with 100 µL of pH = 4.36 phosphate buffer and frozen at − 18 °C. The reaction was conducted for 1606 h with samples taken at selected time intervals. After the experiment was finished, the samples have been defrosted and analyzed using the RP-HPLC method described above.

### Pharmacodynamics

#### In vitro pharmacology

*Radioligand binding.* Radioligand binding assays were performed at Eurofins CEREP (France). The results are expressed as a percent of control specific binding (measured specific binding*100/control specific binding) and as a percent inhibition of control specific binding (100-(measured specific binding*100)/control specific binding) obtained in the presence of the test compounds. The IC_50_ values (concentration causing a half-maximal inhibition of control specific binding) and Hill coefficients (nH) were determined by non-linear regression analysis of the competition curves generated with mean replicate values using Hill equation curve fitting (Y = D + [A-D]/[1 + (C/C_50_)^nH^], where Y = specific binding, A = left asymptote of the curve, D = right asymptote of the curve, C = compound concentration, C_50_ = IC_50_, and nH = slope factor). This analysis was performed using software developed at Cerep (Hill software) and validated by comparison with data generated by the commercial software SigmaPlot^®^ 4.0 for Windows^®^ (© 1997 by SPSS Inc.). The inhibition constants (*K*_*i*_) were calculated using the Cheng Prusoff equation (*K*_*i*_ = IC_50_/[1 + *L*/*K*_D_], where *L* = concentration of radioligand in the assay, and *K*_D_ = affinity of the radioligand for the receptor). A scatchard plot is used to determine the Kd.

*Electrophysiology studies.* Patch-clamp studies for compound **4** were performed at National Institutes of Neurological Disorders and Stroke (National Institutes of Health, NIH, Rockville, USA) within Anticonvulsant Screening Program. Affinities of compound **KM-408** toward Nav1.1–1.8 channels were examined by Chantest Corp., Cleveland, USA. The objective was to examine the in vitro effects of 2 test articles on voltage-gated sodium ion channels expressed in mammalian cells: (1) Cloned hNav1.1 sodium channel (SCN1A gene, expressed in CHO cells), (2) Cloned hNav1.2 sodium channel (SCN2A gene, expressed in CHO cells), (3) Cloned hNav1.3 sodium channel (SCN3A gene, expressed in CHO cells), (4) Cloned hNav1.4 sodium channel (SCN4A gene, expressed in CHO cells), (5) Cloned hNav1.5 sodium channel (SCN5A gene, expressed in CHO cells), (6) Cloned hNav1.6 sodium channel (SCN8A gene, expressed in CHO cells), (7) Cloned hNav1.7 sodium channel (SCN9A gene, expressed in CHO cells), (8) Cloned hNav1.8/beta3 sodium channel (SCN10A gene, co-expressed in CHO cells with the beta3 subunit encoded by the SCN3B gene). The study was conducted in accordance with procedures published in peer-reviewed journals and with the standard operating procedures of ChanTest Corporation. Chemicals used in solution preparation were purchased from Sigma-Aldrich (St. Louis, MO) unless otherwise noted and were of ACS reagent grade purity or higher. Stock solutions of test articles and the positive control were prepared in dimethyl sulfoxide (DMSO) and stored frozen, unless otherwise specified. Test article and positive control concentrations were prepared fresh daily by diluting stock solutions into a HEPES-buffered physiological saline (HB-PS) solution (composition in mM): NaCl, 137; KCl, 4.0; CaCl_2_, 1.8; MgCl2, 1; HEPES, 10; Glucose, 10; pH adjusted to 7.4 with NaOH, which is prepared weekly and refrigerated until use. For hNav1.8/beta3 solutions 0.2 μM TTX were added daily. Since previous results have shown that ≤ 0.3% DMSO and 0.05% pluronic F-127 do not affect channels currents, all test and control solutions will contain 0.3% DMSO and 0.05% F-127. Each test article formulation was sonicated (Model 2510/5510, Branson Ultrasonics, Danbury, CT), at ambient room temperature for at least 20 min to facilitate dissolution. The effects of 3 concentrations of the test article were evaluated. Pre-weighted powder of the test articles was provided and tested in the following concentrations: 10, 30, and 100 µM. The test article formulations were loaded in a glass-lined 384-well compound plate and placed in the plate well of IonWorks QuattroTM (Molecular Devices Corporation, Union City CA).

#### In vivo pharmacology

##### Antiseizure/toxicity screening

The assays were carried out within the Epilepsy Therapy Screening Program (ETSP, previously known as the Anticonvulsant Screening Program, ASP), Epilepsy Branch, National Institute of Neurological and Communicative Disorders and Stroke, National Institutes of Health in Rockville, MD, USA [23]. The methodology is available in the program archive [24].

##### Analgesic activity

*Animals.* The sciatic nerve ligation procedure and formalin test were performed in male albino Sprague–Dawley rats of age 28–38 days (275–700 g, obtained from the Raleigh facility of Charles River) and adult male albino CF No 1 mice (18–30 g, obtained from Charles River, Portage, Michigan), respectively, at National Institutes of Neurological Disorders and Stroke (NINDS) within the Antiepileptic Drug Discovery Program. The breeding conditions are available in the program archive [24].

Other tests were performed at the Jagiellonian University-Medical College on adult male CD-1 mice weighing 18–26 g and male Wistar rats weighing 200–250 g obtained from an accredited animal house at the Faculty of Pharmacy, Jagiellonian University Medical College, Krakow, Poland, and male Outbred CV guinea-pigs (250–300 g), purchased from a licensed breeder (Maria Staniszewska, Ilkowice 41, 32–218 Słaboszów, Poland) were used for the in vivo assays. The animals were kept under constant conditions of ambient temperature (at room temperature of (22 ± 2 °C) under a 12:12 h light–dark cycle, with ad libitum access to a standard pellet diet and tap water. All the experiments were performed between 8 a.m. and 3 p.m. For the experiments, the animals were selected in a random way. Trained observers performed all measurements.

Experimental procedures involving animals performed at Jagiellonian University-Medical College were carried out in accordance with EU Directive 2010/63/EU and approved by the I Local Ethics Committee for Experiments on Animals of the Jagiellonian University in Krakow, Poland (approval numbers 12/2011, 23.02.2011; 123/2011, 16.11.2011)*.*

*Behavioral tests*

*Formalin test.* The first screening in doses 15 and 30 mg/kg was performed at the Jagiellonian University-Medical College. Groups of mice (*n* = 8) were treated *ip* with: vehicle (10 ml/kg, control), and with tested compounds (15 mg/kg and/or 30 mg/kg). After 30 min, animals were injected with 20 μL of a 0.5% formalin solution into the plantar surface of the hind paw. The total time spent by animal licking or biting the injected paw was assessed for the following 40 min.

The formalin test for compounds **4**, **KM-408**, **5**, **5a** and **6** in lower doses has been performed within Epilepsy Therapy Screening Program (ETSP, previously known as the Anticonvulsant Screening Program, ASP), Epilepsy Branch, National Institute of Neurological and Communicative Disorders and Stroke, National Institutes of Health in Rockville, MD, USA [23]. The methodology is available in the program archive [24].

*Sciatic nerve ligation.* The test has been performed within Epilepsy Therapy Screening Program (ETSP, previously known as the Anticonvulsant Screening Program, ASP), Epilepsy Branch, National Institute of Neurological and Communicative Disorders and Stroke, National Institutes of Health in Rockville, MD, USA [23]. The methodology is available in the program archive [24].

*Streptozotocin-induced diabetic neuropathy*

*Induction of diabetes.* To induce diabetes the mice were injected with a single *ip* streptozotocin (STZ, Sigma-Aldrich, Germany) dose (200 mg/kg) dissolved in 0.1 N citrate buffer. Blood glucose levels were measured 1 h before and repeatedly 1, 2 and 3 weeks after STZ injection using a blood glucose monitoring system (AccuChek Active, Roche, France). Blood samples (5 µL) for the measurement of glucose concentration were obtained from the mouse tail vein. The animals were defined as diabetic when their blood glucose concentration exceeded 300 mg/dL and only those mice were used as diabetic mice in further tests. The body weight of mice was monitored 1 h before and 21 days after STZ injection.

*Assessment of tactile allodynia in STZ-treated mice—von Frey test.* Mechanical hyperalgesia was tested as previously described in detail [25] using the electronic von Frey device (Bioseb, France). Briefly, in a quiet room, mice were placed in test compartments on an elevated metal mesh grid and allowed to acclimate for 30 min. After a habituation period each mouse was tested 3 times in the plantar region of hind paw, to obtain baseline values. Subsequently, the mice were pre-treated with the tested compound dissolved in saline at doses 1, 10 and 30 mg/kg. Thirty minutes later the animals were tested again and mean values of mechanical withdrawal threshold were obtained for each mouse.

*Assessment of heat hyperalgesia in STZ-treated mice—hot plate test.* Thermal hyperalgesia was assessed using the hot plate apparatus (Bioseb, France). In this test, after the establishment of pre-drug latency to pain reaction for each animal, the mice were treated with the tested compound and 30 min later they were placed into a glass cylinder on a hot plate set at 55 °C and observed for a nocifensive response (hind paw licking or jumping). The cut-off time was established at 30 s [26].

*Acute, thermally induced pain model—the hot plate test.* The hot plate test was performed as previously described [17]. The mice were *ip* pre-treated 30 min before experiment either with the compound, dissolved in saline (at doses 10, 30 and 100 mg/kg) or vehicle and were placed on a hot plate set at 55 °C (Hot plate, Omega 2A, Poland). Latency to nocifensive reaction (hind paw licking or jumping) of mice was recorded. The mouse was removed from the plate immediately upon licking a hind paw/jumping or if no response occurred within 60 s (cut-off).

*Inflammatory acute pain—writhing test.* In this test, mice were placed individually into glass beakers and were allowed to habituate for the next 30 min. Then, each mouse was injected with tested compound, dissolved in saline (at a dose 30 mg/kg) or vehicle, and then placed back into the glass beaker for 30 min. To induce inflammatory pain, 0.9% acetic acid solution (Polskie Odczynniki Chemiczne, Poland) prepared in saline was injected by the intraperitoneal route. Mice were placed in the beakers again and were observed continuously for the next 30 min. Stereotypical writhes (lengthwise constrictions of the torso with a concomitant concave arching of the back) were counted over this period in drug-treated and control mice [27].

*Neurogenic pain—capsaicin test.* In this test compound dissolved in saline was tested at doses 5, 10, and 30 mg/kg. Compound was administered *ip* and 30 min later 1.6 μg of capsaicin (Sigma-Aldrich, Germany) dissolved in 20 μL of a mixture containing 0.9% saline and ethanol (5% of the final volume) was injected intraplantarly in the ventral surface of the right-hind paw of each mouse. The animals were observed individually for 5 min following capsaicin injection. In all experimental groups, the amount of time spent on licking, biting, or lifting the injected paw was recorded with a chronometer and was considered as an indicator of nociception [28].

*Local anesthetic activity*

*The tail immersion test in mice.* The heat method used for evaluating the systemic analgesic activity can also be used with a slight modification to determine whether a compound possesses local anesthetic activity. The method was performed by subcutaneous (*sc*) injection of the tested substance dissolved in saline, at a concentration range 0.06–2%, in a constant volume of 0.2 mL about 1 cm from the root of the mouse tail. Fifteen minutes later, the 3-cm distal part of the tail was immersed into temperature-controlled water (50 ± 0.5 °C). The reaction time (latency until the tail is pulled away) was measured by the means of a chronometer. In this assay the cut-off was 20 s [29].

*Local anesthetic activity in guinea pigs* [30]

*A. Corneal anesthesia.* The studied compounds, dissolved in saline, were instilled to the right conjunctival sac as 0.125%, 0.25%, and 0.5% solutions in a volume of 0.05 mL, and the same volume of 0.9% saline was applied to the left eye. The corneal reflex was examined by irritation of right eye conjunctiva (studied eye) and a left eye conjunctiva (control eye) by horsehair. The strength of local anesthetic activity was determined from the moment of solution instillation to the moment of reflex return. The presence or lack of corneal reflex were assessed. The eye conjunctiva irritation was done 6 times (every 5 s) with the interval of 5 min during the first 30 min [31].

*B. Infiltration anesthesia (intradermal wheal test).* Infiltration anesthesia was tested in guinea pigs by causing an intradermal wheal by injecting the studied compounds, dissolved in saline, at a constant volume of 0.1 mL and a concentration of 0.125%, 0.25%, and 0.5% to the dorsum skin. The backs of male guinea pigs were shaved 24 h prior to the start of the experiment. A painful reaction to a prick of the skin at the centre of the wheal three times and every 5 s with 5 min intervals during the first 30 min of observation was tested. The experiment was continued to achieve a return of a reaction to the prick. The control wheal was done by an intradermal injection of 0.1 mL of 0.9% NaCl [25]. Local anesthesia was determined by calculating the ratio of the number of positive responses (no flinch) to the number of negative responses (flinch).

*Statistical analysis.* The obtained data were statistically estimated using one-way analysis of variance (ANOVA), followed by Dunnett’s or Tukey’s multiple comparison tests or Student’s t-test. Differences between groups were considered as significant if *p* < 0.05.

### Safety pharmacology

#### Effect on cardiovascular system

*Animals.* Male Wistar rats weighing 200–250 g obtained from an accredited animal house at the Faculty of Pharmacy, Jagiellonian University Medical College, Krakow, Poland, were used. The animals were housed in groups of four in controlled environment (ambient temperature 21 ± 2 °C; relative humidity 50–60%; 12-h light/dark cycles (lights on at 8:00). Standard laboratory food (LSM-B) and filtered water were freely available. All the experiments were performed between 8 a.m. and 3 p.m. Procedures involving animals and their care were conducted in accordance with the current European Community and Polish legislation on animal experimentation. Experimental procedures involving animals performed at Jagiellonian University-Medical College were carried out in accordance with EU Directive 2010/63/EU and approved by the I Local Ethics Committee for Experiments on Animals of the Jagiellonian University in Krakow, Poland (approval numbers 12/2011, 23.02.2011; 123/2011, 16.11.2011)*.*

*Influence on blood pressure in rats*. Rats were anaesthetized with thiopental (75 mg/kg *ip*). The right carotid artery was cannulated with polyethylene tube filled with heparin in saline to facilitate pressure measurements using a Datamax apparatus (Columbus Instruments). The studied compound **KM-408** dissolved in saline was administered into the caudal vein after a 15 min stabilization period, in a dose corresponding to that one from pharmacokinetic studies (5 mg/kg).

*The effect on normal electrocardiogram*. In vivo electrocardiographic investigations were carried out on using ASPEL ASCARD B5, (Aspel, Poland) apparatus, standard lead II, and paper speed of 50 mm/s. The ECG was recorded just prior to and also 1, 2, 3, 5, 10, 15, 20, 30, 40, 50, and 60 min following the *iv* or 5, 10, 20, 30, 40, 50, 60, 70, 80, 90 following the *po* administration of the compound. The assessment of the effects of **KM-408** on the ECG intervals and the heart rate was determined at the dose used in the study of its hypotensive activity (5 mg/kg *iv*). In addition, the effects of **KM-408** on the ECG was studied also after oral administration, at the doses of 50 and 300 mg/kg *po.*

#### Respiratory function

*Whole body plethysmography.* The method, which detects the effects of a test substance on airway function, follows that described by Chong et al. [32] and by Hamelmann et al. [18]. Rats were placed individually in a single chamber plethysmograph (Buxco) in which they can move and drink freely. After a habituation period of 150 min, the following parameters were recorded: inspiratory time, expiratory time, peak inspiratory flow, peak expiratory flow, tidal volume, respiratory rate, relaxation time, pause, enhanced pause. All data generated were acquired using specialized EMKA Technologies software (lOX version 1.7.0; ANALYST version 1.49), connected to Buxco plethysmographic equipment. Recordings were taken for 30 min before and for 4 h after administration of the test substance. Effects were reported at time points 0 (represented by the mean value obtained from T-5, T-15 and T-25 min from the end of the 30-min period before administration), and then 10, 15, 20, 30, 60, 90, 120, 180 and 240 min after administration. Each time point represented the mean value of a 2-min block (recorded 1 min before and 1 min after each time point). In cases where the data can be considered invalid, for example if recorded during a period of sniffing thereby causing a brief period of increased respiratory frequency, the retained values were those as close as possible to the theoretical time point. Results were expressed as absolute values and as percent changes from initial mean values (T0). The experiment included 4 groups (control and 3 test groups); 6 rats were studied per group. Each test substance was evaluated at 3 doses (6 mg/kg, 10 mg/kg, and 20 mg/kg), administered *ip*, and compared with a vehicle control group. Intergroup comparison was performed using a two-way analysis of variance (group, time) with repeated measures at each time, followed by a one-way analysis of variance (group) at each time in case of a significant group x time interaction. The analysis was completed by Dunnett’s *t* tests where group effect was significant.

### Toxicology

#### In vivo toxicology

*Animals.* Adult male CD-1 mice weighing 18–26 g and male Wistar rats weighing 200–250 g obtained from an accredited animal house at the Faculty of Pharmacy, Jagiellonian University Medical College, Krakow, Poland, and male Outbred CV guinea-pigs (250–300 g), purchased from a licensed breeder (Ilkowice 41, 32–218 Słaboszów, Poland) were used for the in vivo assays. The animals were kept under constant conditions of ambient temperature (at room temperature of (22 ± 2 °C) under a 12:12 h light–dark cycle, with ad libitum access to standard pellet diet and tap water. All the experiments were performed between 8 a.m. and 3 p.m. For the experiments, the animals were selected in a random way. Trained observers performed all measurements. Experimental procedures involving animals performed at Jagiellonian University-Medical College were carried out in accordance with EU Directive 2010/63/EU and approved by the I Local Ethics Committee for Experiments on Animals of the Jagiellonian University in Krakow, Poland (approval numbers 12/2011, 23.02.2011; 123/2011, 16.11.2011)*.*

*Acute toxicity.* The tested compounds were dissolved in 0.9% saline and administered *po* and injected into the caudal vein (*iv*) of mouse or rats at the constant volume of 10 mL/kg and 1 mL/kg, respectively. Each dose was given to six animals. Observation of the behavior of the animals was carried out for 6 h, and the mortality was determined after 24 h when administered *iv* and after 72 h when administered *po* LD_50_ values were calculated according to the method of Litchfield and Wilcoxon (1949) [33].

*Statistical analysis.* The obtained data were statistically estimated using one-way analysis of variance (ANOVA), followed by Dunnett’s or Tukey’s multiple comparison tests. Differences between groups were considered as significant if *p* < 0.05.

#### Safety biotechnology

##### Cell culture studies

*Cell culture.* An astrocyte cell line (ATCC, CRL-2541) was used in the study. The cells were cultured under standard conditions (37 °C, 5% CO_2_) in DMEM medium supplemented with 10% FBS and antibiotics.

*MTT assay*. Cells were seeded at a density of 1 × 10^4^ cells per well in 96 well plates. Following overnight culture, the cells were then treated with increasing doses of tested compounds and incubated for 24 h. Following cell exposure to each compound for 24 h in 96-well plates, 10 μL MTT reagent (Cayman) was added to each well and after 4 h of incubation (37 °C, 5% CO_2_), the medium was aspirated, and the formazan produced in the cells appeared as dark crystals at the bottom of the wells. Next, Crystal Dissolving Solution (Cayman) was added to each well. Then the optical density (OD) of each well was determined at 570 nm on a plate reader (BIOTEK).

*CV assay*. Cells were seeded at a density of 1 × 10^4^ cells per well in 96-well plates. After 24 h they were treated with increased doses of tested compounds. After 72 h, cells were fixed for 15 min in a solution of formaldehyde (3.7%), washed with PBS and subsequently stained with 500 μL of 0.01% crystal violet solution for 10 min. The dye that stained the cells on the plates was eluted by 500 μL CH_3_OH solution (25% V/V) of citric acid (1.33% m/V) and sodium citrate (1.09% m/V), and the optical density of the extracted dye was read with a spectrophotometer at 540 nm.

##### Mutagenicity assay

Studies were conducted within Epilepsy Therapy Screening Program (ETSP, previously known as the Anticonvulsant Screening Program, ASP), Epilepsy Branch, National Institute of Neurological and Communicative Disorders and Stroke, National Institutes of Health in Rockville, MD, USA [23]. The test was performed according to a modified pre-incubation method [34]. The test compound was dissolved in DMSO and mixed with 20% (final concentration) cytochrome P450-enriched rat liver S9 post-mitochondrial fraction (20% w/v liver homogenate in 0.25 M Sucrose) in a phosphate buffer and added to an overnight bacterial culture. The mixture was divided into two samples and NADPH added to one of the samples to determine if the test compound requires cytochrome P450 bioactivation to be mutagenic. Both reactions (± NADPH) were incubated at 37 °C for 30 min and then added to a top agar solution containing 0.6% agar, 0.05 mM histidine and 0.05 mM biotin and plated on minimal glucose agar plates. Revertant colonies were counted after a 48 h-incubation at 37 °C. Results are presented as percentage of the number of revertant colonies obtained with 0.02 mM benzo[a]pyrene incubated with NADPH. Acridine orange at 0.02 mM was also included as a positive control.

##### Human cytochrome P450 inhibition

The selective reactions utilized for each enzyme were as follows: CYP1A2—phenacetin deethylation [35], CYP2A6—coumarin 7-hydroxylation [36], CYP2B6—7-ethoxy-4-trifluoromethyl coumarin *O*-deethylation [37], CYP2C9—diclofenac 4-hydroxylation [38], CYP2C19—S-mephenytoin 4-hydroxylation [39], CYP2D6—bufuralol 1’-hydroxylation [40], CYP2E1—chlorzoxazone 6-hydroxylation [41], CYP3A4—testosterone 6β-hydroxylation [42]. For inhibitory evaluation, human liver microsomes and the tested compound (added in methanol as a solvent) were incubated under aerobic conditions with cytochrome P450 isozyme-selective substrates and excess NADPH at 37 °C. Incubations were for time periods over which the reaction in the absence of the compound, were determined to be linear, and proportional to the protein concentration. The human liver microsomes used were selected from the bank of those commercially available to be especially rich in the amount of the cytochrome P450 isozyme under investigation. However, it is important to note that the microsomes contained all the other isozymes in addition, and if reaction/substrate was less than specific, the other isozymes may have contributed to a minor extent to the enzyme activity determined. Following incubation, the reaction was terminated, an internal standard added, the metabolic products extracted and separated by reverse phase HPLC. Each assay was performed in duplicate, and the mean value calculated. The HPLC elutions were monitored by UV absorbance for all CYP reactions except those for CYP2A6, CYP2B6 and CYP2D6 where fluorescence monitoring was employed. Quantification was by comparison with authentic metabolites.

### Pharmacokinetics

#### Pharmacokinetic parameters determination

*Pharmacokinetic study design.* Male Wistar rats (250–300 g) bred in-house from progenitors obtained from Charles River Laboratories (Sulzfed, Germany) were used in this study. Animals were fasted overnight prior to drug administration but had free access to water. Two days after cannulation in the jugular vein (SAI Infusion Technologies, USA), **KM-408** (1, 5, and 10 mg/kg) was dissolved in saline and given intravenously (*iv*) by injection into a tail vein (*n* = 4). Moreover, this compound dissolved in the same solvent was administered orally (*po*) by oral gavage at two doses (50 and 100 mg/kg) (*n* = 4). Blood samples were collected from the catheter at various time points up to 5 h. To assess tissue distribution, **KM-408** was administered *iv* at a dose of 5 mg/kg in a separate experiment. The animals were anaesthetized (50/7.5 mg/kg ketamine/xylazine, *ip*) and sacrificed by decapitation at 5, 15, 30, 60, 120, 180, and 300 min after compound administration (*n* = 4 per time point). Blood was collected into heparinized tubes and organs such as liver, brain, lungs, kidneys, and heart were harvested. The blood samples were centrifuged at 4000 rpm for 10 min (EBA 12R, Hettich, Germany). Plasma and organs were stored at − 80 °C until analysis.

Experimental procedures involving animals performed at Jagiellonian University-Medical College were carried out in accordance with EU Directive 2010/63/EU and approved by the I Local Ethics Committee for Experiments on Animals of the Jagiellonian University in Krakow, Poland (approval number 29/2011, 20.04.2011)*.*

*Determination of KM-408 in plasma and tissue homogenates.* Before analysis, tissue samples were homogenized in phosphate-buffered saline, pH 7.4 (1:4, *w*/*v*) with a tissue homogenizer (TH220; Omni International, Inc., Warrenton, VA, USA). Plasma (100 μL) or homogenate samples (500 μL) were mixed with an internal standard (IS) solution (R-( +)-propranolol,  4 μg/mL in methanol). Then, 20 μL of sodium carbonate (0.125 M) was added to each tube and the samples were extracted with 3 mL of diethyl ether by vortexing for 1 min. After centrifugation (3000 rpm, 15 min), the organic phase was transferred to a new tube and evaporated to dryness under a gentle stream nitrogen at 37 °C in a water bath. The dry residue was reconstituted in 100 μL of acetonitrile. Subsequently, 50 μL of 0.2 M borate buffer (pH = 7.0) and 50 μL of 6 mM FMOC-Cl solution in acetonitrile were added. After vortex-mixing for 1 min, the samples were kept for 30 min at 40 °C in a dry heat sterilizer (model MOV-112S, Sanyo, Japan). The reaction was stopped by the addition of 0.1 M solution of glycine in water (30 μL) and vortexing for 1 min. The reaction mixture was transferred directly into the autosampler vials. The chromatographic system (Merck-HITACHI, Japan) consisted of an L-2485 spectrofluorimetric detector operating at the excitation and emission wavelengths of 267 and 316 nm, respectively, an L-2130 pump, an L-2350 column oven, and an L-2200 autosampler. The EZChrom Elite Client/Server version 3.2 (Agilent Technologies Inc.) was used for data acquisition and processing. The analytes were separated on a LiChroCART^®^ RP-18 column (250 × 4.6 mm) with a particle size of 5 μm protected with a guard column (4 × 4 mm) of the same packing material (Merck, Darmstand, Germany). The column temperature was maintained at 50 °C. The mobile phase consisted of a mixture of acetonitrile and water (67:33, v/v), and pumped at a flow rate of 1 mL/min.

In these conditions the retention times of **KM-408** and IS were approximately 13.8 and 16.5 min. The calibration curve, constructed by plotting the ratios of the peak area of **KM-408** to IS versus **KM-408** concentrations was linear in the tested concentration range, i.e., from 10 to 4000 ng/mL for plasma or from 5 to 2000 ng/g for all tissues. No interfering peaks were present in the chromatogram at the retention times of both compounds. The lower limit of quantification was 10 ng/mL in plasma and 5 ng/g in tissue homogenates. The method was accurate and precise as accuracy of quality control samples evaluated both in plasma and tissue homogenates were in the range of 96.28–113.23%, and the intra- and inter-day coefficients of variation (CV%) were less than 9%. The mean extraction recoveries of **KM-408** and IS were 89.15% and 91.33%, respectively in rat plasma and tissue homogenates.

*Pharmacokinetic data analysis.* Plasma and tissue concentration versus time profiles were analyzed by the non-compartmental approach in Phoenix WinNonlin v. 6.3 software (Pharsight Corporation, Mountain View, CA, USA). The peak concentration (*C*_max_) and the time to reach *C*_max_ (*t*_max_) were obtained directly from individual concentration–time profiles. The initial concentration (*C*_0_) after an *iv* dosing was obtained by extrapolation to zero time. The linear trapezoidal rule was applied to calculate the areas under the concentration–time curve (AUC) from the time of dosing to the last measured data point (AUC_0-t_) or infinity (AUC_0-∞_). The terminal slope (*λ*_z_) was estimated by linear regression and the terminal half-life (*t*_0.5λz_) was calculated as ln2/*λ*_z_. The volume of distribution based on the terminal phase (*V*_z_) was calculated as: Dose/(*λ*_z_ ∙ AUC_0-∞_), whereas clearance (CL) was obtained from the following equation: Dose/AUC_0-∞_. The mean residence time (MRT) was defined as: AUMC_0-∞_/AUC_0-∞_, where AUMC is the area under the first moment curve. The absolute bioavailability (*F*_a_) was calculated according to the equation: (AUC_*po*./_Dose_*po*_)/(AUC_*iv*/_Dose_*iv*_). The tissue-to-plasma AUC ratio (*K*_p_) of **KM-408** was calculated by dividing the AUC_tissue_ by the AUC_plasma_.

#### Metabolites’ identification

*Chemicals and reagents.* Chemicals such as HPLC grade acetonitrile and methanol were supplied by Merck (Darmstadt, Germany). Formic acid and ammonium acetate was obtained from Fluka (Buchs, Switzerland). Purified water (18.2 MΩ) was delivered by a Milli-Q water system (Millipore, Billerica, MA, USA).

*Instrumentation.* Qualitative analyses were performed on an Applied Biosystems/MDS Sciex (Concord, Ontario, Canada) API 2000 triple quadrupole mass spectrometer equipped with an electrospray ionization interface. The instrument was coupled to an Agilent 1100 (Agilent Technologies, Waldbronn, Germany) HPLC system. Data acquisition and processing were accomplished using Sciex Analyst 1.4.2 data collection and integration software. A high-resolution LTQ XL Orbitrap Discovery mass spectrometer (Thermo Scientific, Bremen, Germany) equipped with an electrospray ionization probe was used for metabolite identification in the FT/MS and fragmentation mode at a resolution of 30,000. Data were processed using Xcalibur software.

*Preparation of stock, working solutions and dosage.* The stock solution (1.0 mg/mL ± 0.1) was prepared by dissolving an accurately weighed quantity of **KM-408** in methanol. A working solution of KM-408 at concentrations ranging from 20 to 10,000 ng/mL were prepared by the appropriate dilution of the stock solution using the same solvent. Both stock and working solutions of **KM-408** were stored at 4 ℃ until used. The dose of the compound for intragastric administration was prepared by dissolving 20 mg of the substance in water for injection.

*Identification of metabolites.* A group of 4 adult male Wistar rats (13–15 weeks old, 200–220 g) were used in the experiment. The animals were purchased from the Animal House at the Faculty of Pharmacy, Jagiellonian University Medical College, Krakow, Poland. Experimental procedures involving animals performed at Jagiellonian University-Medical College were carried out in accordance with EU Directive 2010/63/EU and approved by the I Local Ethics Committee for Experiments on Animals of the Jagiellonian University in Krakow, Poland (approval number 29/2011, 20.04.2011)*.*

During the habituation period, the groups of 4 rats were kept in a plastic cage at a controlled room temperature (22 ± 2 °C), humidity (55 ± 10%), full-spectrum cold white light (350–400 lx), on 12-h light/12-h dark cycles (the lights came on at 7:00 a.m., and went off at 7:00 p.m.), and had free access to standard laboratory pellets and tap water. KM-408 dissolved in water for injection was administered intragastrically via a probe at a dose of 20 mg/kg. The blood samples were collected in the 24th hour after compound administration under general anesthesia induced by intraperitoneal (*ip*) injection of 50 mg/kg thiopental. The blood samples were taken into the Eppendorf tubes, allowed to clot and then centrifuged at 3000 × *g* for 10 min, and serum was collected. The serum samples were immediately frozen at − 30 °C. Cumulative urine samples were collected at 0 h (predose) and over the 0–2 h, 2–4 h, 4–8 h, 8–12 h, 12–24 h (postdose) course of the study. Urine samples were stored at − 30 °C until used.

*Sample preparation.* The serum and urine sample pretreatment procedure involved acetonitrile precipitation. For this purpose, 200 µL of acetonitrile was added to 100 µL of serum or urine, thereafter vortexed (Vibrax, IKA) during 20 min, and then centrifuged at 3000 × *g* for 15 min at 4 ℃ (Sigma 14-K). The supernatant (200 µL) was then transferred to insert placed in an autosampler vial, and a 10 μL volume of this was injected onto the XBridge C18 (30 mm × 2.1 mm i.d., 3 μm, Waters, Ireland) analytical column.

*HRMS identification of metabolites.* In the first step, the structures of **KM-408** metabolites were previously generated by the software Pallas (Pallas CompuDrug) and MetaSite (Molecular Discovery). In the next step, the metabolites’ structures were compared with the parent molecule based on the premise that metabolites retain the substructures of the parent compound, undergo a similar MS/MS fragmentation pathway and finally generate product ions and neutral losses associated with those substructures [43]. For this reason, the high-resolution fragmentation mass spectra of metabolites were compared with the specific high-resolution fragments of the parent molecule as a template to interpret the likely pattern of metabolites’ structure. The fragmentation pattern and neutral losses e.g*.* + 16 Da for hydroxylation (+ O), + 32 Da for dihydroxylation (+ O_2_), + 14 Da for methylation (+ CH_2_), + 42 Da for acetyl conjugation (+ C_2_H_2_O), + 96 Da for sulfation (+ SO_4_), + 176 Da (+ C_6_H_8_O_6_) and + 193 Da (+ C_6_H_9_O_7_) for hydroxyl *O*-glucuronidation provided evidence of the molecular connectivity of substructures [44].

The metabolites of **KM-408** were identified using a high-resolution mass spectrometer LTQ XL Orbitrap Discovery by sample continuous scanning at a resolution of 30,000, corresponding to a scan time of 200 ms. Orbitrap was calibrated using a mixture of caffeine, MRFA peptide and Ultramark 1621. Conditions of Orbitrap were as follows: spray needle 5 kV; capillary temperature 275 °C; capillary voltage 34.8 V; tube lens voltage 109.7 V; sheath gas (N_2_) 8; auxiliary gas (N_2_) 5. Product ion mass spectra were generated by a collision-induced dissociation of the protonated molecules. Product ion mass spectra were recorded at four different collision energies (10, 20, 30 and 40 eV) using helium (He) as a collision gas. Default automated gain control values for target ions were used for MS and MS/MS analyses. Four decimal monoisotopic masses were used for the mass list and to filter data in Xcalibur software.

## Results

### Chemistry

#### Synthesis

**Scheme 1:**
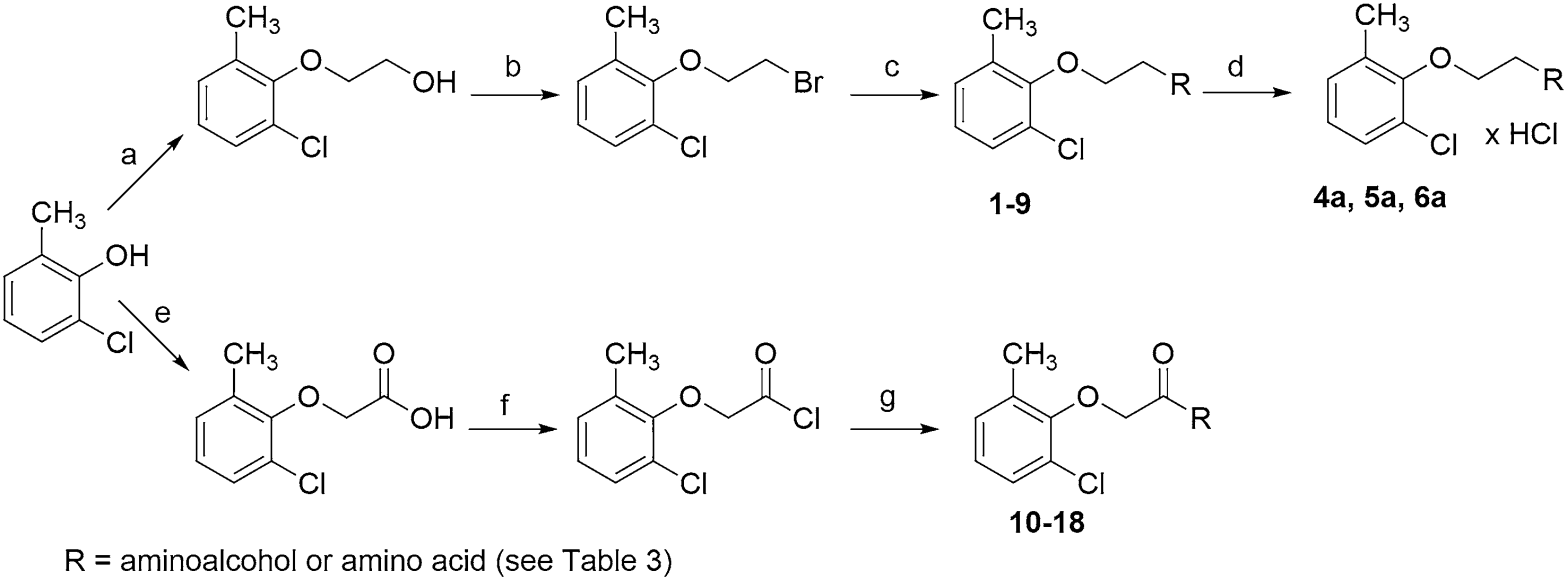
Synthesis of compounds **1**–**18**; **a** 1. C_2_H_5_ONa 2. ClCH_2_CH_2_OH; **b** PBr_3_; **c** aminoalcohol, toluene, K_2_CO_3_; **d** HCl_(g)_; **e** 1. NaOH, 2. ClCH_2_COOH, NaHCO_3_; **f** SOCl_2_; **g** aminoalcohol or amino acid derivative, toluene, H_2_O, K_2_CO_3_

The designed compounds were obtained by chemical synthesis (Scheme 1). The detailed synthetic procedures have been previously decribed in detail for amines [9, 12] and amides [10, 14], including amino acid derivatives [10]. The starting material for the synthesis of all compounds was 2-chloro-6-methylphenol. In the case of the amine derivatives **1**–**9**, 2-chloro-6-methylphenol was converted by the Williamson reaction into 2-(2-chloro-6-methylphenoxy)ethan-1-ol, followed by bromination of the free hydroxyl group to 2-(2-bromoethoxy)-1-chloro-3-methylbenzene. In the case of amide derivatives **10**–**18**, 2-chloro-6-methylphenol was subjected to a Williamson reaction to obtain 2-(2-chloro-6-methylphenoxy)acetic acid, which was then transformed into acid chloride (compounds **10**–**18**). The final step for all derivatives was aminolysis with the use of an appropriate aminoalcohol or amino acid. Amine derivatives **4**–**6** were additionally converted into hydrochlorides by gaseous hydrogen chloride saturation method (compounds **4a**-**6a**).

#### Forced degradation studies

The HPLC–DAD method had been chosen to evaluate the degradation process of **KM-408** under stress conditions according to the ICH requirements [45]. A test procedure was started with the development of the chromatographic method enabling the determination of **KM-408** and its degradation products. Chromatographic separation was performed on a reverse-phase C18 column. Satisfactory results were achieved using a mobile phase composed of ammonium acetate (0.15 M, pH 4.4) and methanol (45:55, v/v), at a flow rate of 1.1 mL min^−1^ at 25 ºC and the analysis time of 15 min. The retention time for **KM-408** under the developed conditions was ~ 3.7 min. Detection of the peak area for **KM-408** and degradation products was carried out at 230 nm that corresponded to the absorption maximum of **KM-408** (Fig. S1).

In the HPLC–UV chromatogram registered for standard solution of **KM-408** at a concentration of 2% w/v the peak of impurity was observed at the retention time 6.2 min. The concentration of the impurity 1.64% was determined using internal normalization method (Fig. S2).

The substance was very stable in 0.5 M HCl at 70 °C. After hydrolysis for 232 h only 1.65% of degradation was observed. After hydrolysis in 0.5 M NaOH at 70 °C only 2.02% of degradation was observed after 232 h. A very similar behavior was observed when degradation was carried out in phosphate buffer pH = 7.0. At lower temperatures at 25 °C after 240 h only 1.52% and at 70 °C after 168 h only 1.96% of degradation was observed.

During the degradation in the presence of H_2_O_2_, the substance was very resistant to the applied conditions, as only 1.84% of degradation products was observed after 168 h. When CuSO_4_ was applied as oxidative agent, 1.65% of degradation was observed after 192 h. In the presence of reducing agent at 40 °C after 168 h 1.50% of degradation was determined and during photodegradation studies conducted for 96 h 1.43% of degradation was observed.

In the stress tests conducted in acidic and basic pH, at 70 °C, **KM-408** proved stable under acidic conditions and some decomposition was observed under basic conditions. Therefore, **KM-408** degradation kinetics under basic conditions has been studied. Borate buffer pH = 9.9 was selected as one of standard buffers at this pH range. Initial attempts showed that under basic conditions, **KM-408** hydrochloride was converted to free base, insoluble in the buffer. For a kinetic study, the homogeneity of the system is critical; therefore, an organic modifier to increase **KM-408** free base solubility under measurement conditions was used. Initially, three organic modifiers were considered: ethanol, dimethyl sulfoxide (DMSO) and 1,4-dioxane. For these modifiers, approximate **KM-408** solubility tests were conducted, using pH 9.9 borate buffer—organic modifier systems, in volume of the solvent corresponding to the planned kinetic experiment. **KM-408** dissolved fully in 20% ethanol and 20% dioxane, whereas for DMSO, full solubility has not been achieved even above 30%. Finally, dioxane was selected due to its higher boiling point, which reduced the risk of evaporation during a prolonged experiment. During the experiment, three degradation products were observed (*t*_R_ = 13 min, *t*_R_ = 15 min and *t*_R_ = 17 min). Kinetic analysis has indicated that degradation of **KM-408** under basic conditions proceeds as a reaction of first order. The degradation rate constant *k*, *t*_0.5_ and *t*_0.1_ are presented in Table 1.

**Table 1:** Summary of **KM-408** degradation kinetics studies under basic conditions: the final results and experimental conditions

Conditions			Rate constant *k* (h^−1^)	Half-life *t*_0.5_ (h)	*t*_0.1_ (h)
Temperature	pH	Modifier			
70.0 ± 0.5 °C	9.90	1,4-dioxane, 20% v/v	0.83·10^–3^	834.9	127.0

### Pharmacodynamics

#### In vitro pharmacology

Compound **4** and its salt form **KM-408** were subjected to high throughput receptor studies performed at Eurofins Cerep, France (binding to receptors/transporters at 100 µM; results available in Supplementary material, Table S1). For compound **KM-408** the screening was followed by IC_50_ and K_i_ determination for targets at which 50% binding was noticed (Table 2 and Supplementary material, Fig. S3). The lowest IC_50_ value has been observed for sigma (*σ*) receptors (8.9*10^–8^ M), serotoninergic 5-HT_1A_ and 5-HT_2B_ receptors (1.3*10^–6^ M and 2.4*10^–6^ M, respectively), as well as 5-HT transporter (3.6*10^–6^ M). The compound is, thus, a non-selective ligand of σ receptors, with a mild affinity for a number of other molecular targets.

**Table 2:** Radioligand binding results as half maximal inhibitory concentration (IC_50_) and inhibition constant (K_i_) for compound **KM-408** (receptors that exhibited > 50% binding at 100 μM)

Receptor^a^	IC_50_ (M)	K_i_ (M)
α_1_ (non-selective)^ant^	1.2*10^–5^	3.3*10^–6^
α_2_ (non-selective)^ant^	2.4*10^–5^	1.0*10^–5^
D_2_S^ant^	1.0*10^–4^	3.4*10^–5^
D_3_^ant^	2.2*10^–5^	4.9*10^–6^
D_4.4_^ant^	6.6*10^–5^	2.5*10^–5^
M_1_^ant^	6.4*10^–5^	5.5*10^–5^
M_4_^ant^	2.4*10^–5^	1.5*10^–5^
M_5_^ant^	3.5*10^–5^	1.8*10^–5^
κ (KOP)^ag^	2.5*10^–5^	1.7*10^–5^
**5-HT**_**1A**_^**ag**^	1.3*10^**–6**^	8.0*10^**–7**^
5-HT_2A_^ant^	5.5*10^–5^	3.0*10^–5^
**5-HT**_**2B**_^**ag**^	2.4*10^**–6**^	1.2*10^**–6**^
5-HT_2C_^ant^	7.7*10^–5^	2.6*10^–5^
5-HT_7_^ag^	1.8*10^–5^	6.5*10^–6^
**σ (non-selective)**^**ag, b**^	8.9*10^**–8**^	7.2*10^**–8**^
Ca^2+^ channel (L, verapamil site) (phenylalkylamine)^ant^	4.3*10^–5^	2.2*10^–5^
Na^+^ channel (site 2)^ant, b, c^	1.5*10^–5^	1.4*10^–5^
Dopamine transporter^ant^	4.3*10^–5^	2.3*10^–5^
**5-HT transporter**^**inh**^	3.6*10^**–6**^	1.7*10^**–6**^

Receptors that exhibited < 50% (including hERG) were omitted

^a^Radioligand used: ag—agonist, ant—antagonist, inh—inhibitor

^b^Radioligand binding studies at 10 µM revealed no selectivity (% inhibition of specific control binding equal to 95.0% and 94.8% for σ1 and σ2, respectively)

^c^As patch-clamp experiments performed for compound **4** on NIE-115 cells indicated potential impact of this compound on sodium currents (Supplementary material, Table S2), affinities of its salt form **KM-408** toward Na_v_1.1–1.8 channels were also determined (Supplementary material, Table S3). No specificity toward any of tested Na_v_ channel types was observed

#### In vivo pharmacology

##### Antiseizure and preliminary toxicity screening

The obtained compounds were screened for antiseizure activity and neurotoxicity in vivo within the Epilepsy Therapy Screening Program (ETSP, previously known as the Anticonvulsant Screening Program, ASP), Epilepsy Branch, National Institute of Neurological and Communicative Disorders and Stroke, National Institutes of Health in Rockville, MD, USA [23]. All compounds were evaluated for antiseizure activity in maximal electroshock seizure (MES) test and in rotarod test for neurotoxicity in mice, *i.p*. In case of promising activity, compounds were proceeded to extended research in other models including studies in various epilepsy models and/or studies in rats *ip*/*po* The summary of results is presented in Table 3.

**Table 3:** Chemical structures of compounds **1**–**18** and a summary of screening studies for antiseizure activity and neurotoxicity

Compd.	R	Antiseizure activity and preliminary toxicity screening (doses expressed in mg/kg)

**1**		MES: active at 30 Rotarod: neurotoxic at 100
**2**		MES: active at 100 Rotarod: neurotoxic at 100
**3**		MES: active at 30 Rotarod: neurotoxic at 100
**4**		MES: ED_50_ (0.25 h) = 21.44 (18.63–25.32), PI = 2.56 female mice, *ip*: TPE = 0.25 h, ED_50_ (0.25 h) = 11.64 (10.55–12.47) mice, *po*: TPE = 0.5 h, ED_50_ = 58.13 (45.58–68.93), PI = 6.12 rats, *ip*: TPE = 0.25 h, ED_50_ (0.25 h) = 7.3 (5.39–9.89), PI < 6.85 rats, *po*: ED_50_ (0.25 h) = 67.69 (43.01–93.82), PI > 3.55; ED_50_ > 60 rats, *ip*: ED_50_ (0.25 h) = 7.3 (5.39–9.89), PI = 4.5 6 Hz: TPE (32 mA) = 0.25 h, ED_50_ (32 mA, 0.25 h) = 32.4 (28.4–36.0) TPE (44 mA) = 0.25 h, ED_50_ (44 mA, 0.25 h) = 29.75 (28.3–31.5) Corneal kindling: TPE = 0.25 h, ED_50_ (0.25 h) = 19.51 (11.86–28.41) Hippocampal kindling: rats, *ip*: avg. seizure score by Racine (0–5) = 5 at 40 (0.25–2.25 h) LTG-resistant amygdala seizures: rats, *ip*: active at 15 (0.25 h) Frings mice sound-induced seizures: ED_50_ (0.25 h) = 6.05 (4.23–8.45) Picrotoxin-induced seizures: ED_50_ (0.25 h) > 70 Bicuculline-induced seizures: ED_50_ (0.25 h) > 70 MTLE mice acute screen evaluation: TPE = 0.25 h, 80.8 ± 16.8% of baseline at 30 (recording period 5–25 min) Pilocarpine-induced status epilepticus prevention: rats, *ip*: no activity at 40 (0.0 h), TD_97_ = 40.53 Rotarod: TD_50_ (0.25 h) = 54.99 (52.95–56.94) female mice, *ip*: TD_50_ (0.25 h) = 40.11 (36.49–42.98) mice, *po*: TD_50_ = 355.98 (292.89–417.09) rats, *ip*: TD_50_ (0.25 h) = 32.85 (29.25–35.51) rats, *p.o*: TD_50_ (0.25 h) > 240
**4a** **KM-408**		MES: TPE = 0.25 h, ED_50_ (0.25 h) = 13.3 (11–15.6), PI = 4.83 rats, *ip*: TPE = 0.25 h, ED_50_ (0.25 h) = 5.69 (3.99–7.25), PI = 9.55 Corneal kindling: avg. seizure score by Racine (0–5) = 2.5 at 30 (0.25 h) 6 Hz: TPE (32 mA) = 0.25 h, ED_50_ (32 mA, 0.25 h) = 29.4 (23.5–41.8) TPE (44 mA) = 0.25 h, ED_50_ (44 mA, 0.25 h) = 35.15 (24.43–43.46) mice, *po*: TPE (44 mA) = 0.25 h, ED_50_ (44 mA, 0.25 h) = 280.66 (191.28–452.66) Hippocampal kindling: rats, *ip*: death at 200 (0.25 h) Rotarod: TD_50_ (0.25 h) = 64.3 (45.1–82.4) rats, *ip*: TD_50_ (0.25 h) = 54.32 (44.12–60.57)
**5**		MES: ED_50_ (0.25 h) = 18.20 (16.60–20.00), PI = 3.29 rats, *ip*: TPE = 0.25 h, MES: ED_50_ (0.25 h) = 4.65 (2.94–6.88), PI > 9.12 rats, *po*: TPE = 0.5 h 6 Hz: TPE = 0.25 h, ED_50_ (32 mA, 0.25 h) = 33.1 (29–37.8) LTG-resistant amygdala seizures: rats, *ip*: active at 30 (0.25 h), seizure score by Racine (0–5) = 3.5 (3/4 rats – toxicity); active at 50 mg/kg (0.25 h, rats, *ip*), seizure score by Racine (0–5) = 2.2 (5/5 rats – toxicity) Hippocampal kindling: rats, *ip*: TPE = 0.75 h seizure score by Racine (0–5) = 4 (Rat1) and 0 (Rat2) at 30 Pilocarpine-induced status epilepticus prevention: rats, *ip*: active at 50 (0.25 h and 0.5 h), at 100 2/2 rats died (0.25 h) Rotarod: TD_50_ (0.25 h) = 59.80 (43.80–74.20) rats, *ip*: TPE = 0.25 h, TD_50_ (0.25 h) > 30 Acute toxicity: rats, *ip*: minimal motor impairment at 50 (0.25 h, 0.5 h)
**5a**		MES: ED_50_ (0.25 h) = 19.78 (16.16–22.33), PI = 2.73 mice, *po*: TPE = 0.25 h, ED_50_ (0.25 h) = 64.12 (52.15–75.37), PI = 5.03 rats, *ip*: TPE = 0.25 h, ED_50_ (0.25 h) = 3.9 (2.1–6.2), PI = 10.15 6 Hz: TPE = 0.5 h, ED_50_ (44 mA) = 52.49 (41.85–67.05) mice, *po*: TPE (44 mA) = 0.25 h, ED_50_ (44 mA, 0.25 h) = 185.04 (126.56–245.59) Corneal kindling: seizure score by Racine (0–5) = 4.3 at 25 (0.25 h) Hippocampal kindling: rats, *ip*: TPE = 0.25 h, seizure score by Racine (0–5) = 4 (Rat1) and 0 (Rat2) at 40 Pilocarpine-induced status epilepticus prevention: rats, *ip*: no activity at 40 Rotarod: TD_50_ (0.25 h) = 54.01 (35.70–64.23) mice, *po*: TPE = 0.25 h, TD_50_ (0.25 h) = 322.33 (257.98–382.69) rats, *ip*: TPE = 0.25 h, TD_50_ (0.25 h) = 39.6 (36.2–78.3)
**6**		MES: ED_50_ (0.25 h) = 20.8 (13.8–29.4), PI = 2.29 rats, *ip*: TPE = 0.5 h, ED_50_ = 10.58 (6.69–15.38), PI = 4.19 rats, *po*: ED_50_ (0.25 h) = 20.8 (13.8–29.4) 6 Hz: TPE = 0.25 h, ED_50_ (32 mA, 0.25 h) = 20.4 (14.9–25.4) Pilocarpine-induced status epilepticus prevention: rats, *ip*: active at 45, at 90—8 deaths in 9 rats Corneal kindling: ED_50_ (0.25 h) = 10.50 (3.67–22.09) Hippocampal kindling: rats, *ip*: ED_50_ (0.25 h) = 15.81 Rotarod: TD_50_ (0.25 h) = 47.7 (32.6–68.3) rats, *ip*: TPE = 0.25 h, TD_50_ (0.25 h) = 44.29 (40.72–47.52)
**6a**		MES: active at 100 Rotarod: neurotoxic at 100
**7**		MES: active at 100 Rotarod: neurotoxic at 100
**8**		MES: active at 30 Rotarod: neurotoxic at 100
**9**		MES: active at 30 Rotarod: neurotoxic at 100

**10**		MES: ED_50_ = 97.93, PI = 1.43 ScPTZ: active at 300 Rotarod: neurotoxic at 300
**11**		MES: active at 300 Rotarod: neurotoxic at 100
**12**		MES: active at 300 (0.5 h, 4 h) ScPTZ: active at 100 Rotarod: neurotoxic at 300
**13**		No activity
**14**		No activity
**15**		No activity
**16**		No activity
**17**		Rotarod: neurotoxic at 300
**18**		MES: active at 30 8 Hz: active at 100 Rotarod: neurotoxic at 100
**Carbamazepine** [46]		MES: ED_50_ = 7.81 (6.32–8.45) 6 Hz: Max. 75% protection at 40 and 80 Frings mice sound-induced seizures: ED_50_ = 11.2 (7.73–16.2) Rotarod: TD_50_ = 45.4 (32.9–54.4)
**Propranolol** [5]		MES: ED_50_ = 15–20
**Allopregnanolone** [47]		MES: TPE = 0.17 h, ED_50_ > 100 ScPTZ: TPE = 0.17 h, ED_50_ = 13.7 (10.1–18.7) 6 Hz: TPE (32 mA) = 0.17 h, ED_50_ (32 mA) = 14.2 (10.3–19.4)

Unless otherwise stated, results 0.5 h after induction of convulsions (antiseizure activity) or administration of the compound (toxicity), mice, *ip*; ED_50_ – dose active in 50% of animals; LTG – lamotrigine; MES – maximal electroshock seizure test; PI – protective index; ScPTZ – subcutaneous pentylenetetrazole seizure test; TD_50_ – dose toxic in 50% of animals; TPE – time to peak effect. Full pharmacological data for compounds **1**–**17** are available on request from the corresponding author or in patent [20] while for compound **18** in Supplementary material (Table S4)

**Fig. 2:**
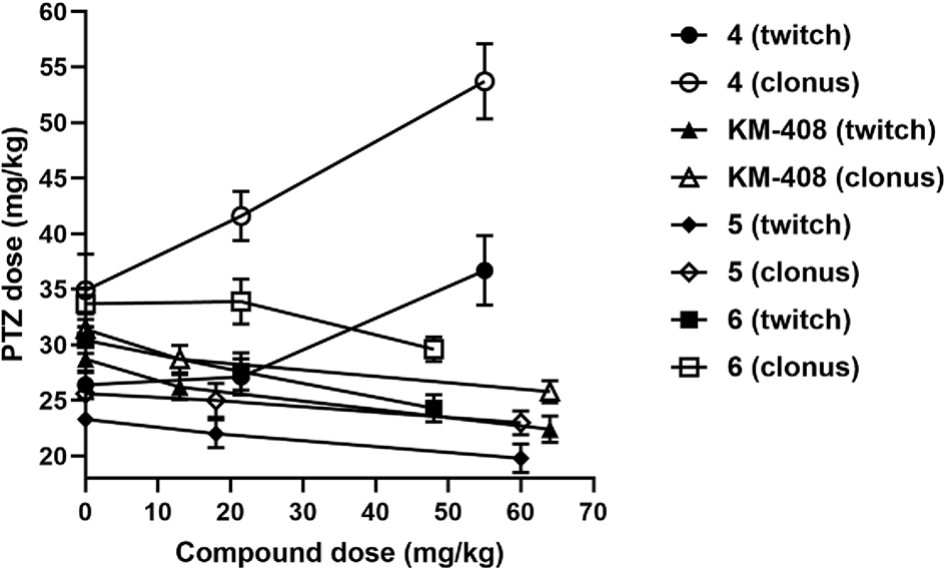
Influence of compounds **4**, **KM-408**, **5** and **6** on seizure threshold [pentylenetetrazole (PTZ)-induced seizures, mice, *ip*]; 0.5% PTZ solution administered *iv* at a constant rate of 0.34 mL/min. Data are expressed as the mean PTZ dose ± SEM for *n* = 10 that induced clonus or twitch in mice exposed to a particular dose of the test compound

Among the tested compounds, the amine derivatives exhibited more favorable pharmacological properties (all tested amines active in MES test). Among the amides, only compounds **10–12** and **18** exhibited a mild antiseizure activity. The most pronounced effect was observed for *R,S*-2N-[(2-chloro-6-methylphenoxy)ethyl]aminobutan-1-ol (**4**), as well as its *R* (**5**) and *S* (**6**) enantiomers (analog of reference compound **I**, Fig. 1B). These three compounds were chosen for extended research as bases or hydrochlorides (compounds **4a—KM-408**, **5a** and **6a**). The results are presented in Table 3. The evaluations covered MES test in mice and rats (after *ip* and *po* administration), several epilepsy models (6 Hz test, lamotrigine-resistant amygdala seizures, sound-induced seizures, picrotoxin-induced seizures, bicuculline-induced seizures, corneal kindling, hippocampal kindling, status epilepticus prevention) and included quantitative studies (ED_50_ and TD_50_ evaluation). They were also evaluated in the *iv* pentylenetetrazole (PTZ) test. The assay allows to determine the effect of drugs on separate components of seizure behavior and has known utility for evaluation of proconvulsant potential of a compound [48]. The observed antiseizure activity does not exclude potential for lowering the seizure threshold by the same compound. As seen in Fig. 2, compound **4** lowered the seizure threshold, however, the effect was not observed for its salt form, namely **KM-408**.

##### Antinociceptive activity evaluation

*Antinociceptive activity in the formalin test (tonic, chemically induced pain model)*

Compounds **KM-408**, **5a** and **6a** were tested for their potential antinociceptive activity in the formalin test (mice, *ip*). Formalin-induced pain behavior is biphasic: the initial acute phase (I phase, 0–5 min, neurogenic pain) is followed by a quiescent period (II phase, 5–15 min), which is then followed by a prolonged tonic response (15–40 min, inflammatory pain). Among tested compounds, **KM-408** and **6a** showed a significant antinociceptive activity at 30 mg/kg in the I phase of pain (Table 4). Due to the beneficial properties of the compound **KM-408** in this test, it has been studied also at lower doses: 21 mg/kg (in base form, compound **4**) and 13.25 mg/kg, together with compounds **5**, **5a** and **6** at 20 mg/kg (Table 5). The most pronounced effect was observed for compound **6** at 20 mg/kg in the II (inflammatory) phase of test (57% of control).

**Table 4:** Antinociceptive activity of compounds **KM-408**, **5a** and **6a** in the formalin-induced pain model (mice, *ip*)

Compd.	Dose (mg/kg)	Time of licking paw ± SEM (I phase) (s)	% of effect (I phase)	Time of licking paw ± SEM (II phase) (s)	% of effect (II phase)
**Control** **(0.9% NaCl)**	–	89.7 ± 5.3	–	30.5 ± 9.6	–
**KM-408**	30.0	60.6 ± 8.0*	32. 4	22.4 ± 6.5	26.6
**5a**	30.0	72.3 ± 8.7	19.4	13.5 ± 5.4	55.7
	15.0	94.3 ± 6.5	+ 5.1	25.5 ± 10.3	16.4
**6a**	30.0	59.7 ± 6.4*	33.4	27.4 ± 7.9	10.2

Data are presented as mean ± SEM for *n* = 8. One-way ANOVA followed by Dunnett’s post hoc test: *p < 0.05, **p < 0.01 (I phase: *F*_4,39_ = 5.573, *p* = 0.0012; II phase: *F*_4,41_ = 0.5338, *p* = 0.7116)

**Table 5:** Antinociceptive activity of compounds **4**, **KM-408**, **5**, **5a** and **6** in formalin-induced pain (mice, *ip*)

Compd.	Dose (mg/kg)	Area under the curve			
		(I phase)	% of control; *t*, df (I phase)	(II phase)	% of control; *t*, df (II phase)
**Control**	–	194.9 ± 31.8	64.0; *t*_14_ = 4.0720	680.9 ± 220.1	72.7, *t*_14_ = 0.6407
**4**	21	124.8 ± 32.7**		569.2 ± 405.0	
**Control**	–	151.8 ± 17.9	75.0; *t*_14_ = 1.4820	591.8 ± 431.5	82.8; *t*_14_ = 0.5623
**KM-408**	13.25	113.7 ± 65.6		489.7 ± 211.3	
**Control**	–	197.2 ± 72.8	78.7; *t*_14_ = 1.3050	470.6 ± 100.1	105.6; *t*_14_ = 0.3579
**5**	20.0	155.1 ± 44.2		496.7 ± 164.7	
**Control**	–	242.3 ± 125.2	76.5; *t*_14_ = 0.8847	749.3 ± 248.8	91.5; *t*_14_ = 0.5440
**5a**	20.0	185.3 ± 115.8		685.6 ± 184.6	
**Control**	–	195.0 ± 93.0	69.3; *t*_14_ = 1.5800	677.0 ± 180.2	57.2; *t*_14_ = 3.6330
**6**	20.0	135.3 ± 36.4		387.0 ± 110.1**	

Data are presented as mean ± SEM for *n* = 8. Student’s *t*-test: **p < 0.01

*Analgesic activity in the sciatic nerve ligation (SNL) model (chronic, neuropathic pain model)*

**Fig. 3:**
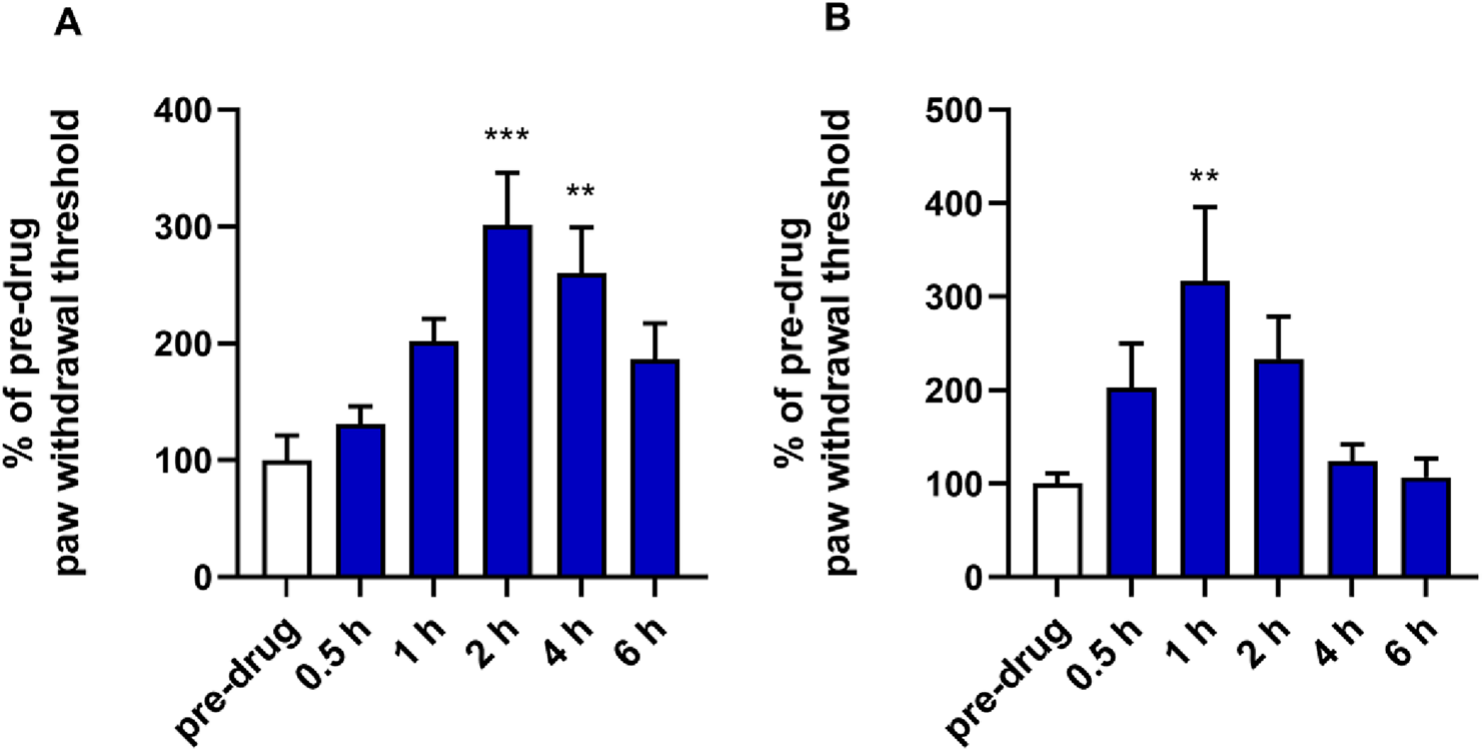
Activity in sciatic nerve ligation (SNL) model (von Frey test) for **A** **KM-408** (rats, *ip*, 6 mg/kg) and **B** **5a** (rats, *ip*, 4 mg/kg). Data are presented as mean ± SEM for *n* = 7–8; pre-drug (white bar) and post-drug at various time points of testing (blue bars). One-way ANOVA (**A** *F*_5,40_ = 6.159, *p* = 0.0003; **B** *F*_5,39_ = 3.735, *p* = 0.0074) followed by Dunnett’s post hoc test: **p < 0.01, ***p < 0.001 (compared to the pre-drug value)

The analgesic activity (von Frey test) of **KM-408** in the sciatic nerve ligation model (SNL) was observed at 6.0 mg/kg (rats, *ip*), i.e., at the dose close to the ED_50_ obtained for this compound in the MES test (5.69 mg/kg; rats, *ip*; Table 3). As it is seen in Fig. 3A (and Table S5 in Supplementary material), in animals treated with **KM-408,** the mechanical nociceptive threshold is increased significantly 2 h (TPE) and 4 h after administration. Similar observations can be made for compound **5a** (TPE = 1 h, dose tested = 4 mg/kg, Fig. 3B and Table S5 in Supplementary material). Taken together, in this model of neuropathic pain active doses of compounds **KM-408** and **5a** were almost two–threefold lower as minimal effective dose (MED) of Oxcarbazepine (SNL MED = 10 mg/kg, rats, *ip*) and ED_50_ value of Gabapentin (SNL ED_50_ = 32 mg/kg, rats, *ip*) [49].

*Streptozotocin-induced diabetic neuropathy (chronic, neuropathic pain model)*

Approx. 70% of mice developed diabetes (the blood glucose level > 300 mg/dl) and these animals were used in subsequent pain tests: von Frey test (assessment of the effect of **KM-408** on tactile allodynia) and hot plate test (assessment of the influence of the test compound on heat hyperalgesia). Mean body weight of mice before streptozotocin (STZ) administration was 20.9 ± 0.2 g, whereas 21 days later, the mean body weight in diabetic mice was 26.8 ± 0.4 g, whereas in the control group (normoglycemic mice) the mean body weight was 30.9 ± 0.8 g.

In the von Frey test in nondiabetic control mice, mean pain sensitivity threshold for mechanical stimulation was 3.08 ± 0.2 g, whereas in STZ-treated, diabetic mice pain sensitivity significantly increased, which resulted in a reduction of mechanical nociceptive threshold to 1.77 ± 0.1 g. In this model, compound **KM-408** at doses 1, 10 and 30 mg/kg significantly elevated the pain threshold in STZ-treated mice by 132% (*p* < 0.001), 125% (*p* < 0.001), and 164% (*p* < 0.001), respectively.

**Fig. 4:**
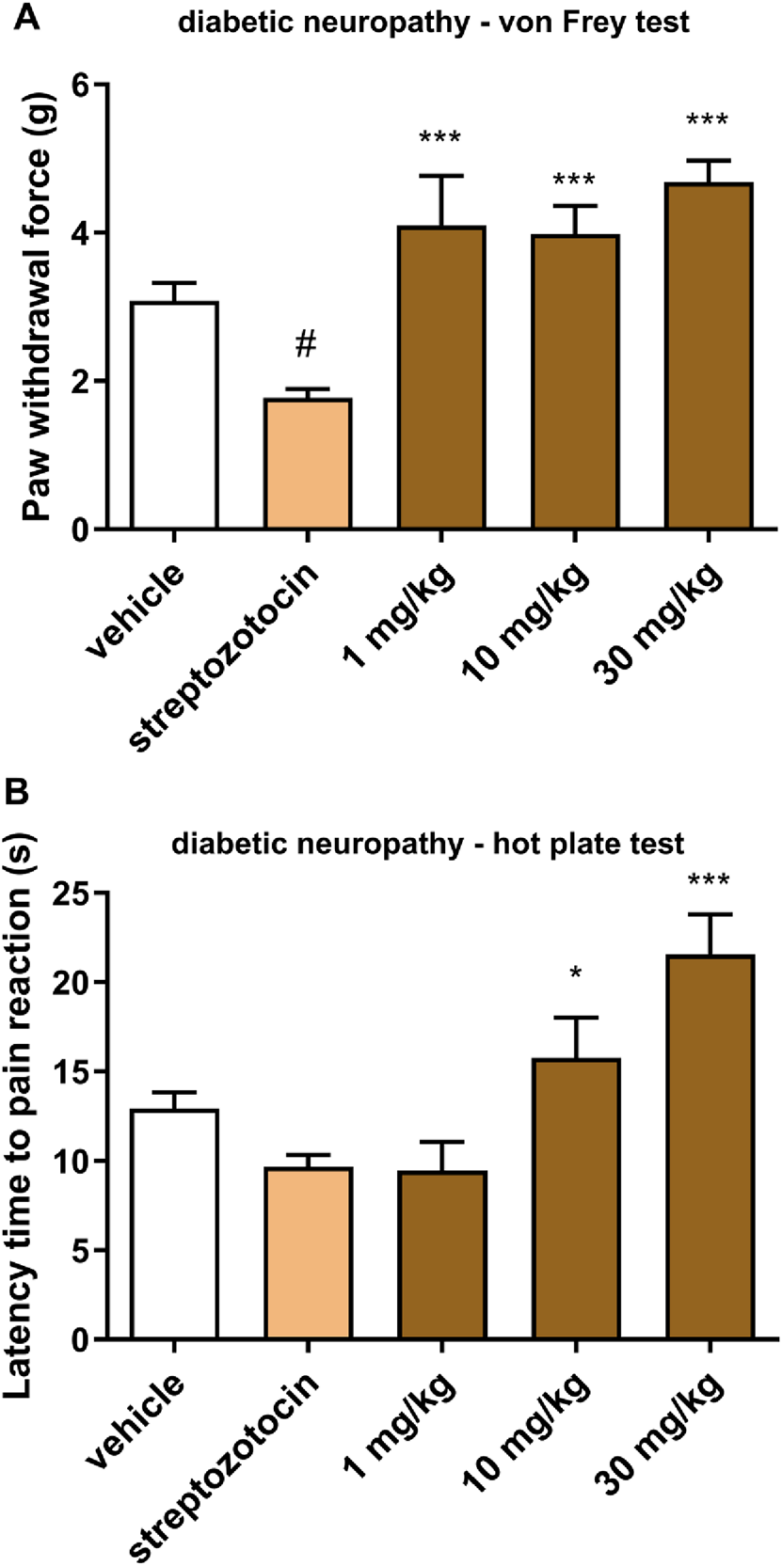
Effects of compound **KM-408** on tactile allodynia measured using **A** von Frey test, and **B** heat hyperalgesia assessed in the hot plate test in the mouse diabetic neuropathic pain model induced by streptozotocin (STZ). Data are presented as mean ± SEM for *n* = 10; vehicle (white bar), streptozotocin (beige bar) and **KM-408** + streptozotocin at various time points of testing (brown bars). One-way ANOVA (von Frey test: *F*_4,63_ = 15.7, *p* < 0.0001; hot plate test: *F*_4,63_ = 12.13, *p* < 0.0001) followed by Tukey’s post hoc test: ^#^*p* < 0.05 (compared to the normoglycemic control); *p < 0.05, ***p < 0.001 (compared to the STZ-treated group)

In the hot plate test in nondiabetic control mice (normoglycemic control group), the baseline latency to pain reaction was 12.93 ± 0.9 s, whereas in STZ-treated mice the obtained value was 9.68 ± 0.7 s. Compound **KM-408** at doses of 10 and 30 mg/kg was able to prolong significantly the latency to pain reaction to 15.8 ± 2.2 s (by 63%, *p* < 0.001 vs. diabetic control) and to 21.6 ± 2.2 s (by 123%, *p* < 0.05 vs. diabetic control), respectively. The dose 1 mg/kg of **KM-408** was not effective in this assay. These results are presented in Fig. 4.

*Antinociceptive activity in the hot plate test (acute, thermally induced pain model)*

**Fig. 5:**
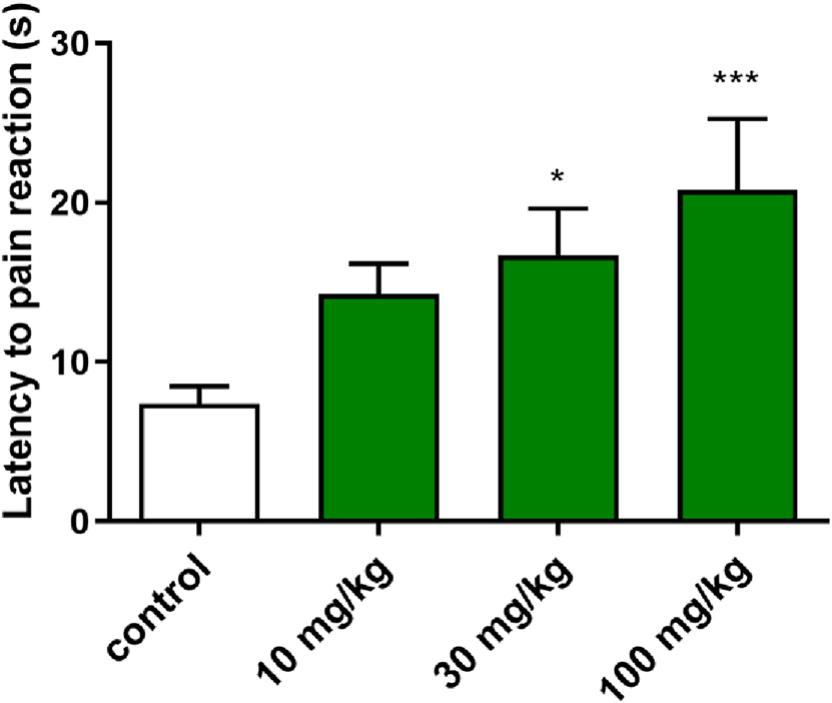
Antinociceptive activity of compound **KM-408** in the hot plate test in mice. Data are presented as mean ± SEM for *n* = 8–10; control (white bar) and **KM-408** at various doses (green bars). One-way ANOVA (*F*_3,46_ = 7.714, *p* = 0.0003) followed by Dunnett’s post hoc test: *p < 0.05, ***p < 0.001 (compared to the vehicle-treated group)

Since acute pain often requires the use of higher doses of analgesics than the chronic form of pain, **KM-408** administered *ip* at doses 30 and 100 mg/kg was also tested for its ability to relieve acute, thermally induced pain. The test compound demonstrated a statistically significant analgesic activity, as it effectively prolonged the nocifensive response by 123% (*p* < 0.05 vs. control) and 177% (*p* < 0.001 vs. control), at doses 30 and 100 mg/kg, respectively. At a lower dose (10 mg/kg) no significant effect on the thermal pain threshold was observed. The results obtained are presented in Fig. 5.

*Antinociceptive activity in the writhing test*

**Fig. 6:**
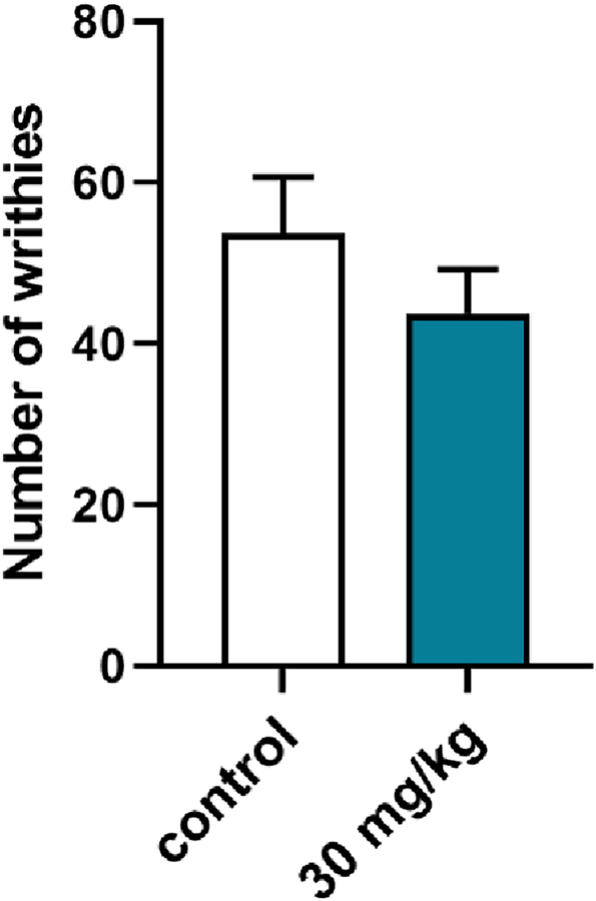
Antinociceptive activity of compound **KM-408** in the writhing test in mice. Data are presented as mean ± SEM for *n* = 9–10; control (white bar) and **KM-408** at 30 mg/kg (blue bars). *t*-test (ns, *t* = 1405)

In this test, which is a mouse model of visceral pain, compound **KM-408** tested at a dose 30 mg/kg (the lowest active dose in the hot plate test, Fig. 5) was not significantly effective in reducing acetic acid-induced writhing behavior. The results are presented in Fig. 6.

*Antinociceptive activity in the capsaicin test (neurogenic pain model)*

**Fig. 7:**
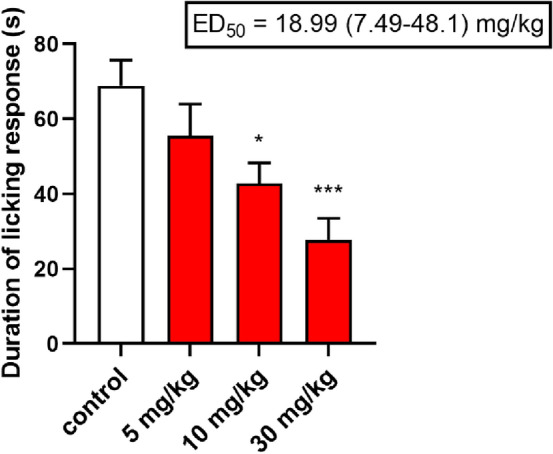
Antinociceptive activity of compound **KM-408** (*ip*) in the capsaicin test in mice. Data are presented as mean ± SEM for *n* = 8; control (white bar) and **KM-408** at various doses (red bars). One-way ANOVA (*F*_3,28_ = 6.757, *p* = 0.0014) followed by Tukey’s post hoc test: *p < 0.05, ***p < 0.001 (compared to the vehicle-treated group)

In this test, compound **KM-408** at doses 10 and 30 mg/kg significantly reduced the licking response of capsaicin-treated mice from 68.9 ± 6.8 s (control group) to 42.7 ± 5.6 s (by 38%, *p* < 0.05) and to 27.7 ± 5.7 s (by 60%, *p* < 0.001), respectively. At a lower dose (5 mg/kg) it diminished nociceptive response by 19% (vs. control group), but this difference did not reach statistical significance. These results are presented in Fig. 7.

*Local anesthetic activity in tail immersion test*

The effect of compounds **KM-408**, **5a** and **6a** on local anesthesia was examined using the tail immersion test. At concentrations of 0.5% and 2% the compound **KM-408** significantly prolonged the animals’ reaction time to a heat stimulus by 166% (*p* < 0.001) and 323% (*p* < 0.0001), respectively. Compounds **5a** and **6a** showed statistically significant local anesthetic effect at concentrations 0.125%, 0.5%, and 2.0%. The local anesthetic activity of all tested compounds was higher than that of the reference compound—phenytoin. These results obtained are presented in Table 6.

**Table 6:** Local anesthetic activity of compounds **KM-408**, **5a** and **6a** in the tail immersion test in mice

Compd.	Concentration (%)	Reaction time to painful stimulus (s)	Prolongation of the time reaction (%)
**Vehicle**	0.9% NaCl	3.5 ± 0.3	–
**KM-408**	0.062	4.4 ± 1.0	25.7
	0.125	5.0 ± 1.0	42.9
	0.25	7.4 ± 1.0	111.4
	0.5	9.3 ± 0.9***	165.7
	2.0	14.8 ± 1.9****	322.9
**5a**	0.015	3.8 ± 0.5	8.6
	0.031	4.9 ± 0.9	40.0
	0.094	5.5 ± 1.6	57.1
	0.125	13.9 ± 1.8****	297.1
	0.5	18.7 ± 1.3****	434.3
	2.0	17.2 ± 1.9****	391.4
**6a**	0.015	3.7 ± 0.6	5.7
	0.031	5.6 ± 0.9	60.0
	0.094	6.4 ± 0.8	82.9
	0.125	15.7 ± 1.8****	348.6
	0.5	19.5 ± 0.5****	457.1
	2.0	18.8 ± 1.9****	437.1
**Vehicle**	0.9% NaCl	4.83 ± 0.60	–
**Phenytoin** [29]	1.0	5.72 ± 0.63	18
	2.0	8.90 ± 0.80***	84
**Vehicle**	0.5% Methylcellulose	7.26 ± 2	–
**Mepivacaine **[50]	1.0	14.13 ± 2.67	94.63
	2.0	15.73 ± 2.09**	116.67

Data are presented as mean ± SEM for *n* = 8–10. One-way ANOVA followed by Dunnett’s post hoc test: **p < 0.02, ***p < 0.001, ****p < 0.0001 (compared to the vehicle-treated group)

*Corneal anesthesia and infiltration anesthesia in guinea pigs*

Experiments performed in guinea pigs showed a potent local anesthetic activity of compounds **KM-408**, **5a** and **6a**. In the model of corneal anesthesia, the inhibition of pain reaction was the most potent for.

**KM-408**, however for all tested compounds used at concentration of 0.5% the effect was stronger than for reference compound—lidocaine (also 0.5%).

In the model of infiltration anesthesia, the significant local anesthetic activity was observed for all tested compounds. The most pronounced local anesthetic effect was shown for **KM-408**—its local anesthetic activity lasted up to 2 h after the compound’s administration, and at this time point of testing was twofold higher than that of lidocaine. These results are presented in Tables 7 and 8.

**Table 7:** Local anesthetic activity of the investigated compounds in corneal anesthesia model in guinea pigs

Compd.	Concentration (%)	Inhibition of pain reaction (%)			
		5 min	30 min	60 min	120 min
**KM-408**	0.125	55.5***	0	0	0
	0.25	80.5***	0	0	0
	0.5	94.4***	8.3	0	0
**5a**	0.125	46.67*	0	0	0
	0.25	36.11*	0	0	0
	0.5	80.55***	0	0	0
**6a**	0.125	22.22*	0	0	0
	0.25	63.89***	0	0	0
	0.5	75***	0	0	0
**Lidocaine** [17]	0.5	61.1***	0	0	0

Data are presented as mean ± SEM for *n* = 6. *t*-test: *p < 0.05,***p < 0.001

**Table 8:** Local anesthetic activity of the investigated compounds in infiltration anesthesia model in guinea pigs

Compd.	Concentration (%)	Inhibition of pain reaction (%)					
		5 min	30 min	60 min	120 min	240 min	24 h
**KM-408**	0.125	100***	33.3*	38.9*	33.3*	11.1	0
	0.25	100***	81.2***	64.7**	70.6***	33.3*	0
	0.5	100***	93.3***	81.2***	66.7***	20.0	0
**5a**	0.125	83.33***	44.44**	22.22	11.11	5.55	0
	0.25	100***	55.55**	33.33*	22.22	16.67	0
	0.5	100***	72.22***	58.82**	52.94**	27.78	0
**6a**	0.125	83.33***	33.33*	33.33*	22.22	11.11	0
	0.25	94.44***	27.78	38.89*	27.78	5.55	0
	0.5	100***	93.75***	43.75*	25	11.11	0
**Lidocaine** [17]	0.5	93.7***	50.0*	44.4*	33.3*	22.2	0

Data are presented as mean ± SEM for *n* = 6. *t*-test: *p < 0.05, **p < 0.01, ***p < 0.001

### Safety pharmacology

#### Effect on cardiovascular system

##### The effect on the blood pressure

**Fig. 8:**
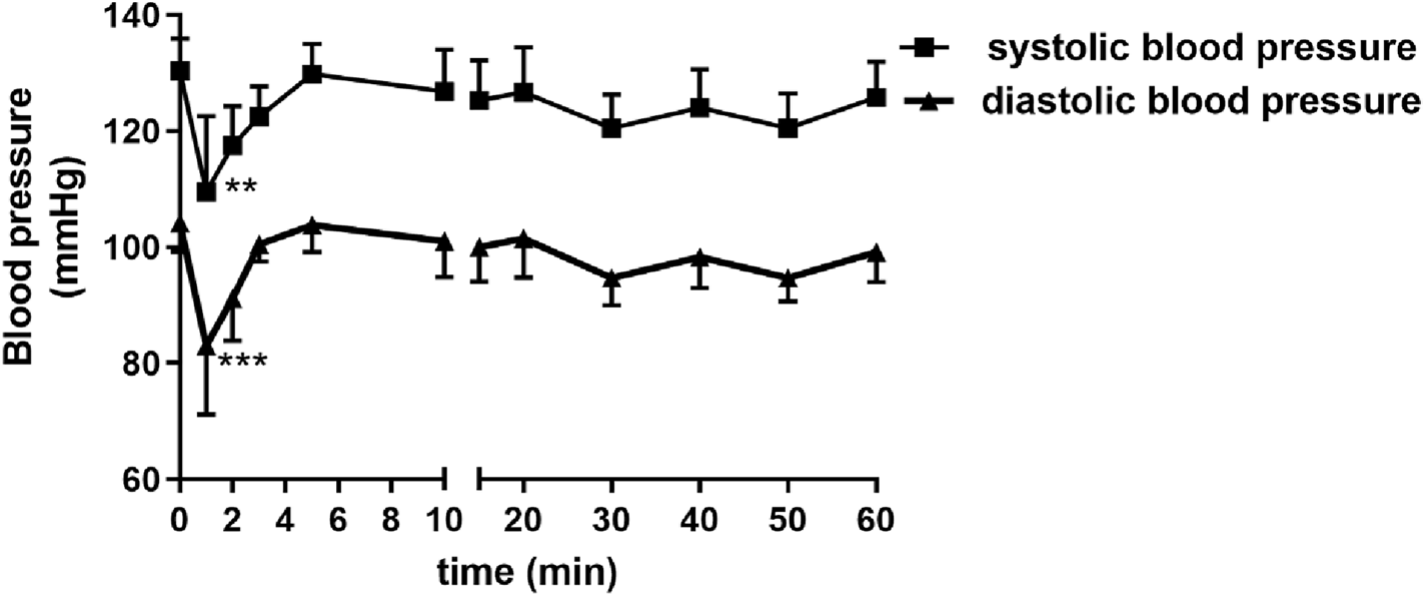
The effect on blood pressure of **KM-408** (5 mg/kg *iv*) in normotensive anaesthetized rats. Data are presented as mean ± SEM for *n* = 6. One-way repeated measures ANOVA (systolic blood pressure: *F*_11,55_ = 2.564, *p* = 0.0107, diastolic blood pressure: *F*_11,55_ = 3.17, *p* = 0.0022) followed by Dunnett’s post hoc test: **p < 0.01, ***p < 0.001

Compound **KM-408** at the dose of 5 mg/kg *iv* produced strong but short-lasting reduction of blood pressure immediately after administration (Fig. 8). It lowered systolic (*F*_11,55_ = 2.564, *p* = 0.0107) and diastolic (*F*_11,55_ = 3.17, *p* = 0.0022) blood pressure by approximately 21 mmHg. Later this effect disappeared, and blood pressure returned to the baseline.

##### The effect on normal electrocardiogram

In vivo ECG study showed that **KM-408** (5 mg/kg *iv*) did not influence QRS (*F*_10,40_ = 0.905, *p* = 0.538) and QT (*F*_10,40_ = 1.588, *p* = 0.146) intervals (Table 9). However, **KM-408** markedly decreased the heart rhythm (*F*_10,40_ = 3.864, *p* = 0.0010) by approx. 12.4–8.8%. The negative chronotropic effect started immediately after administration and lasted until the end of the observation period. The observed bradycardia was accompanied with PQ interval prolongation (*F*_10,40_ = 2.694, *p* = 0.0127) in the 1st minute.

**Table 9:** Effect of compound **KM-408** on the heart rate and ECG intervals in anaesthetized rat (thiopental, 75 mg/kg *ip*) after an *iv* and *po* administration

*iv*												
Dose (mg/kg)	Parameter	Time of observation (min)										
		0	1	3	5	10	15	20	30	40	50	60
**5**	Beats /min	325.6 ± 11.1	290.9 ± 17.1***	285.3 ± 16.2***	291.3 ± 15.7***	294.1 ± 14.7**	292.8 ± 12.4***	294.3 ± 13.2**	291.4 ± 13.3***	290.4 ± 15.8***	292.7 ± 13.7***	296.8 ± 15.5**
	PQ (ms)	50.5 ± 2.6	59.5 ± 5.3***	54.8 ± 2.9	54.4 ± 2.3	54.0 ± 1.9	54.4 ± 1.9	54.4 ± 2.0	54.8 ± 1.7	53.6 ± 2.2	53.6 ± 2.6	53.6 ± 1.5
	QRS (ms)	20.0 ± 2.2	20.8 ± 2.0	21.2 ± 2.3	19.6 ± 1.8	20.6 ± 2.1	20.4 ± 2.2	20.6 ± 2.2	20.4 ± 2.0	20.4 ± 2.6	20.0 ± 2.2	20.0 ± 2.2
	QT (ms)	56.8 ± 3.8	58.0 ± 3.4	60.0 ± 2.8	56.8 ± 3.9	56.2 ± 4.2	55.2 ± 4.1	56.0 ± 4.0	57.2 ± 3.7	57.0 ± 3.7	58.8 ± 3.4	56.0 ± 4.0

*po*												
Dose (mg/kg)	Parameter	Time of observation (min)										
		0	5	10	20	30	40	50	60	70	80	90
**50**	Beats /min	349.9 ± 15.0	324.8 ± 13.2	320.4 ± 15.1	323.4 ± 14.5	327.3 ± 6.0	327.6 ± 8.3	322.5 ± 6.9	335.2 ± 17.2	308.3 ± 8.6	312.9 ± 11.5	313.0 ± 17.6
	PQ (ms)	55.6 ± 1.8	59.2 ± 1.9	59.6 ± 2.1	59.0 ± 1.0	57.4 ± 1.2	59.2 ± 1.5	58.4 ± 1.0	58.4 ± 1.6	59.6 ± 1.0	59.6 ± 0.4	59.6 ± 1.3
	QRS (ms)	15.2 ± 0.8	16.4 ± 0.7	16.0 ± 0.6	16.4 ± 0.7	16.0 ± 0.6	16.4 ± 0.5	16.0 ± 0.6	17.0 ± 1.1	16.4 ± 1.0	17.2 ± 1.3	16.4 ± 1.0
	QT (ms)	55.2 ± 3.9	60.0 ± 0.0	59.2 ± 0.8	58.0 ± 2.0	58.4 ± 1.0	59.2 ± 0.8	58.8 ± 1.2	58.8 ± 1.2	61.2 ± 4.2	60.0 ± 0.0	60.0 ± 0.0
**300**	Beats /min	391.8 ± 25.7	376.1 ± 17.0	362.1 ± 19.7	346.2 ± 20.6*	341.0 ± 21.8*	329.4 ± 19.8**	322.1 ± 20.2***	323.0 ± 20.6***	331.7 ± 33.7**	328.3 ± 22.3**	328.8 ± 20.3**
	PQ (ms)	51.6 ± 3.6	51.2 ± 3.4	54.8 ± 2.1	55.2 ± 2.2	55.2 ± 2.2	55.2 ± 2.1	55.8 ± 2.8	56.0 ± 2.8	55.4 ± 2.3	56.0 ± 2.4	56.0 ± 2.4
	QRS (ms)	12.80 ± 0.8	13.6 ± 0.4	13.6 ± 0.4	14.2 ± 0.2	14.0 ± 0.0	14.4 ± 0.4	14.2 ± 0.2	14.2 ± 0.2	13.4 ± 0.9	13.0 ± 0.9	14.0 ± 0.0
	QT (ms)	58.0 ± 2.0	57.2 ± 2.0	55.6 ± 2.0	58.0 ± 2.0	58.0 ± 2.0	59.2 ± 0.8	60.0 ± 0.0	59.2 ± 0.8	60.0 ± 0.0	60.0 ± 0.0	59.6 ± 0.4

Data are presented as mean ± SEM for *n* = 5. One-way repeated measures ANOVA test followed by Dunnett’s post hoc test: *p < 0.05, **p < 0.01, ***p < 0.001 (compared to the baseline values)

**KM-408** at a dose of 50 mg/kg *po* did not influence heart rate (*F*_10,40_ = 0.8087, *p* = 0.621) or ECG intervals [PQ: (*F*_10,40_ = 0.7531, *p* = 0.6715), QRS: (*F*_10,40_ = 0,9562, *p* = 0.4950), QT: (*F*_10,40_ = 0.6443, *p* = 0.7671)]. However, at a dose of 300 mg/kg *po*, **KM-408**, significantly decreased the heart rhythm (*F*_10,40_ = 4.367, *p* = 0.0004) and induced bradycardia starting from 20 min after administration, by approx. 11.6–17.8% with no influence on other electrocardiogram (ECG) parameters [PQ: (*F*_10,40_ = 2.223, *p* = 0.0361), QRS: (*F*_10,40_ = 1.296, *p* = 0.2656), QT: (*F*_10,40_ = 1.473, *p* = 0.1854)].

#### Respiratory function

Due to the high concentrations of **KM-408** observed in rat lungs (PK analysis), the respiratory function was analyzed with regards for possible adverse effects in the respiratory system. The effects of compound **KM-408** on airway function was evaluated with a use of whole body plethysmography (male Wistar rats, *ip*, doses 6, 10 and 20 mg/kg) at Porsolt, France [18, 32]. During the experiment, the following parameters were recorded: inspiratory time, expiratory time, peak inspiratory flow (the maximum box pressure signal occurring during one breath in a negative direction), peak expiratory flow (the maximum box pressure signal occurring during one breath in a positive direction), tidal volume (the integral of inspiratory time), respiratory rate (breaths/min), relaxation time (the time of pressure decay to 30% of the total expiratory pressure signal), pause [(expiratory time – relaxation time)/relaxation time] and enhanced pause (pause × peak expiratory flow/peak inspiratory flow). No significant effects of compound **KM-408** on the tested parameters were observed over the 4-h test period, as compared with vehicle control group. The detailed results are available in Supplementary material (Figs. S4–S12).

### Toxicology

#### In vivo toxicology

The acute toxicity of tested compound **KM-408** was examined in mice and rats after *po* as well as *iv* administration according to Litchfield and Wilcoxon [33]. The LD_50_ values are given in Table 10.

**Table 10:** Acute toxicity of compound **KM-408** in mice and rats after *iv* and *po* administration

Compd.	LD_50_ value (mg/kg)			
	*iv*		*po*	
	Mice	Rats	Mice	Rats
**KM-408**	19.82 (13.35–29.43)	22.55 (18.62–27.29)	246.19 (177.85–340.80)	669.91 (584.81–767.39)

Each value was obtained from three experimental groups; each group consisted of six animals; the median lethal dose (LD_50_) values and their confidence limits were calculated according to the log-probit method of Litchfield and Wilcoxon (1949) [33]

#### Safety biotechnology/biochemistry

##### Cell culture studies

**Fig. 9:**
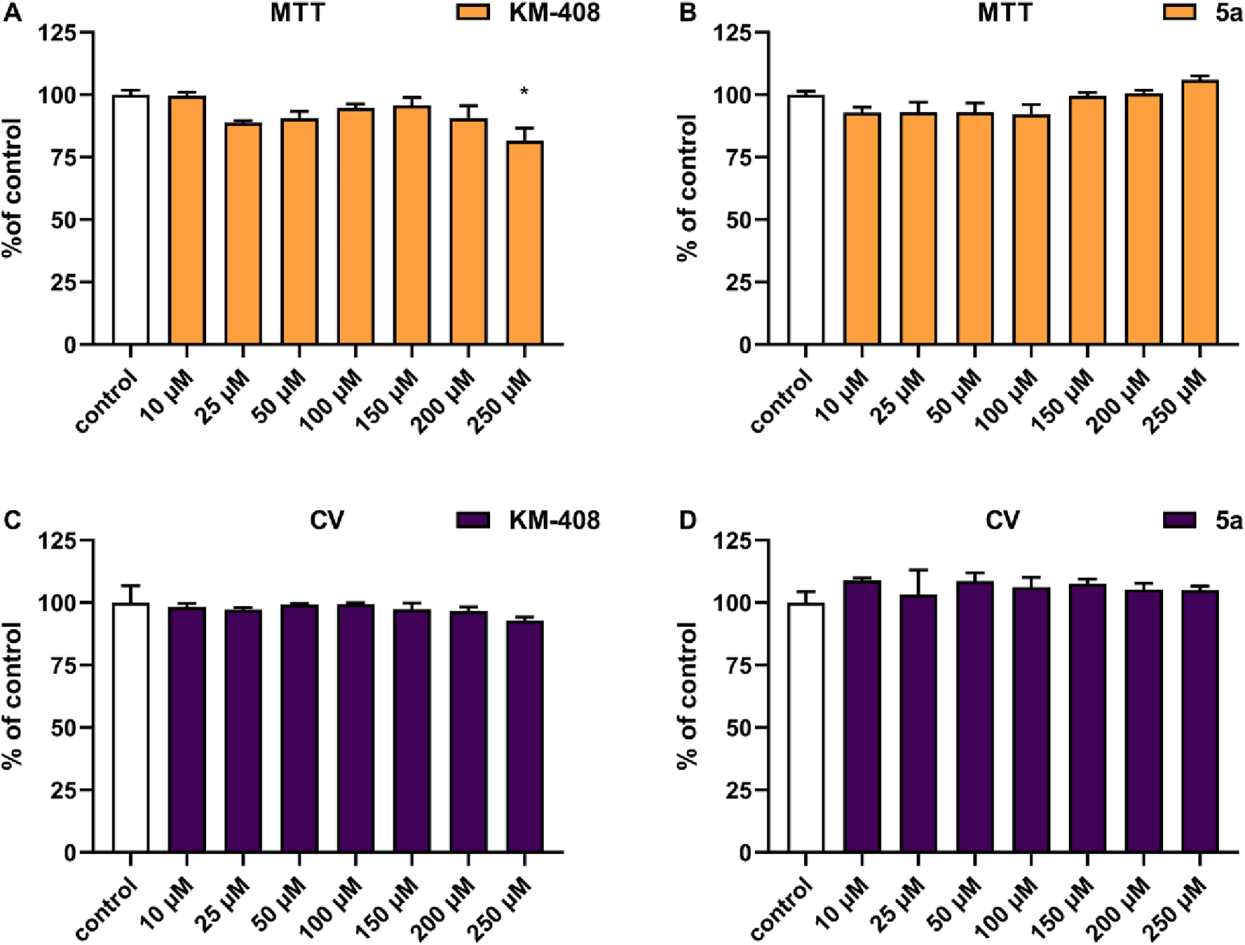
Results of cell culture studies (murine astrocytes) performed for compounds **KM-408** and **5a**: **A** and **B** metabolic impairment evaluation [3-(4,5-dimethylthiazol-2-yl)-2,5 diphenyl tetrazolium bromide (MTT) test], **C** and **D** impact on proliferation evaluation (crystal violet assay). Data are presented as mean ± SEM for *n* = 4; control (white bar) and tested compounds at various doses (colored bars). One-way ANOVA (**A** *F*_7,24_ = 3.935, *p* = 0.0054; **B** *F*_7,24_ = 3.576, *p* = 0.0089; **C** *F*_7,24_ = 0.6796, *p* = 0.6877; **D** *F*_7,24_ = 0.4571, *p* = 0.8555) followed by Dunnett’s post hoc test: *p < 0.05 (compared to the control group)

Compounds **KM-408** and **5a** were selected for studies on cytotoxicity (metabolic impairment evaluation, MTT test) and impact on proliferation (crystal violet assay) of murine astrocytes (Fig. 9). The analyzed compounds did not exhibit cytotoxic activity and a notable impact on proliferation. Only compound **KM-408** exerted some cytotoxicity, decreased cell viability to 81% when compared to control in the highest concentration (250 µM).

##### Mutagenicity assay

Compound **4** was tested for potential mutagenicity with a use of Ames test based on *Salmonella enterica* sv. Typhimurium TA 98 strain (a modified pre-incubation method). The analysis of the results involves counting the number of revertant bacterial colonies in the presence or absence of the chemical compound being tested. The inclusion of S9 fraction from cytochrome P450-enriched rat liver homogenates mimics the in vivo activity of metabolic enzymes in activating some pro-mutagens to mutagens. At tested concentrations 0.1–0.5 mM the compound was non-mutagenic when compared to DMSO solvent control (Table 11).

**Table 11:** Results of Ames test performed for compound **4**

Compd	Concentration (mM)	% of benzo[a]pyrene (+NADPH) control			
		+ NADPH		− NADPH	
		Sample 1	Sample 2	Sample 1	Sample 2
**None**	0	6.2	7.3		
**DMSO**	0.04	5.8	6.3		
**4**	0.10	7.8	7.8	5.5	7.4
	0.20	5.2	7.4	5.4	5.3
	0.50	5.2	7	4.9	6.7
**Benzo[a]pyrene**	0.02	100	100	6.5	6
**Acridine orange**	0.02	117	128	17.9	21.1

##### Human cytochrome P450 inhibition

Compound **4** inhibition of isozyme selective reactions revealed that there was a substantial inhibition of CYP1A2, CYP2A6, CYP2C19 and CYP2D6 (Table 12). Inhibition of these enzymes fits to the competitive or mixed mechanism, with K_i_ values of 23, 371, 54 and 1.4 µM, respectively.

**Table 12:** Human cytochrome P450 inhibition by compound **4**

Compd.	Isozyme	K_i_ (µM)	Inhibition type	% inhibition of 500 µM concentration
**4**	CYP1A2	23.0	Mixed/competitive	65 (substrate at 2 µM)
	CYP2A6	371.0	Mixed/competitive	44 (substrate at 10 µM)
	CYP2B6		Weak inhibition	69 (substrate at 25 µM)
	CYP2C9		Slight inhibition	19 (substrate at 400 µM)
	CYP2C19	54.0	Mixed/competitive	80 (substrate at 150.0 µM)
	CYP2D6	1.4	Mixed/competitive	100 (substrate at 100.0 µM)
	CYP2E1		Slight inhibition	17 (substrate at 500.0 µM)
	CYP3A4		Weak inhibition	52 (substrate at 200.0 µM)

### Pharmacokinetics

#### Determination of pharmacokinetic parameters

**Fig. 10:**
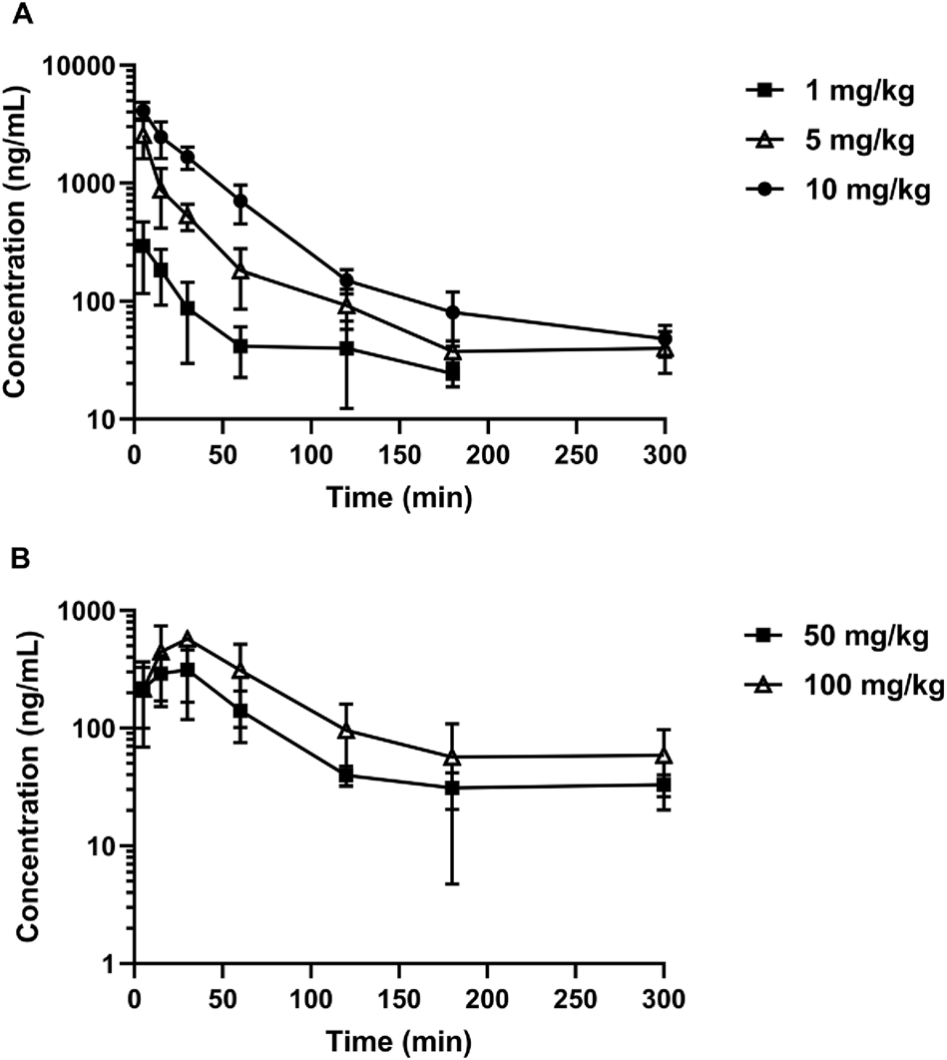
Plasma concentrations versus time profiles of **KM-408** following **A** intravenous (*iv*) and **B** oral (*po*) administration of different doses to rats (*n* = 4)

**KM-408** plasma concentrations following administration of this compound intravenously (3 doses) and orally (2 doses) as a function of time are presented in Fig. 10.

Pharmacokinetic parameters of **KM-408** assessed by the non-compartmental approach as listed in Table 13.

**Table 13:** Pharmacokinetic parameters of **KM-408** in plasma following *iv* and *po* administration of this compound to rats assessed using non-compartmental analysis

*iv*			
Parameter	Dose (mg/kg)		
	**1**	**5**	**10**
*C*_0_ (ng/mL)	370.86	4361.21	5329.03
*λ*_z_ (min^−1^)	0.032	0.018	0.027
*t*_0.5λz_ (min)	21.80	37.94	26.02
*V*_z_ (L/kg)	2.53	3.77	2.30
CL (L/min/kg)	0.08	0.07	0.06
AUC_0-t_ (ng⋅min/mL)	12,397.9	72,379.5	163,345.1
MRT (min)	51.71	43.54	42.29

*po*		
Parameter	Dose (mg/kg)	
	**50**	**100**
*t*_max_ (min)	30	30
*C*_max_ (ng/mL)	313.39	573.26
*λ*_z_ (min^−1^)	0.023	0.020
*t*_0.5λz_ (min)	30.59	34.98
*V*_z_/F (L/kg)	80.35	103.95
CL/F (L/min/kg)	1.95	2.06
AUC_0-t_ (ng⋅min/mL)	25,822.82	48,412.86
MRT (min)	79.13	82.97
*F* (%)	3.9	3.6

To evaluate tissue penetration of **KM-408**, its concentrations in several tissues were measured after *iv* administration of the dose of 5 mg/kg and, based on these data, the non-compartmental analysis was carried out. The results of this analysis are presented in Table 14.

**Table 14:** Pharmacokinetic parameters of **KM-408** in tissues following *iv* administration of this compound at a dose of 5 mg/kg to rats assessed using non-compartmental approach

Parameter	5 mg/kg *iv*				
	Liver	Brain	Lungs	Kidneys	Heart
*t*_max_ (min)	5	5	5	5	15
*C*_max_ (ng/g)	161.09	1771.16	4346.92	1945.11	494.26
*λ*_z_ (min^−1^)	0.017	0.024	0.023	0.043	0.017
*t*_0.5λz_ (min)	41.99	28.56	30.84	16.23	41.30
AUC_0-t_ (ng⋅min/g)	10,224.29	60,126.61	127,399.7	44,786.78	27,685.10
MRT (min)	112.52	37.87	46.94	25.26	58.46
*K*_p_	0.14	0.83	1.76	0.62	0.38

**Fig. 11:**
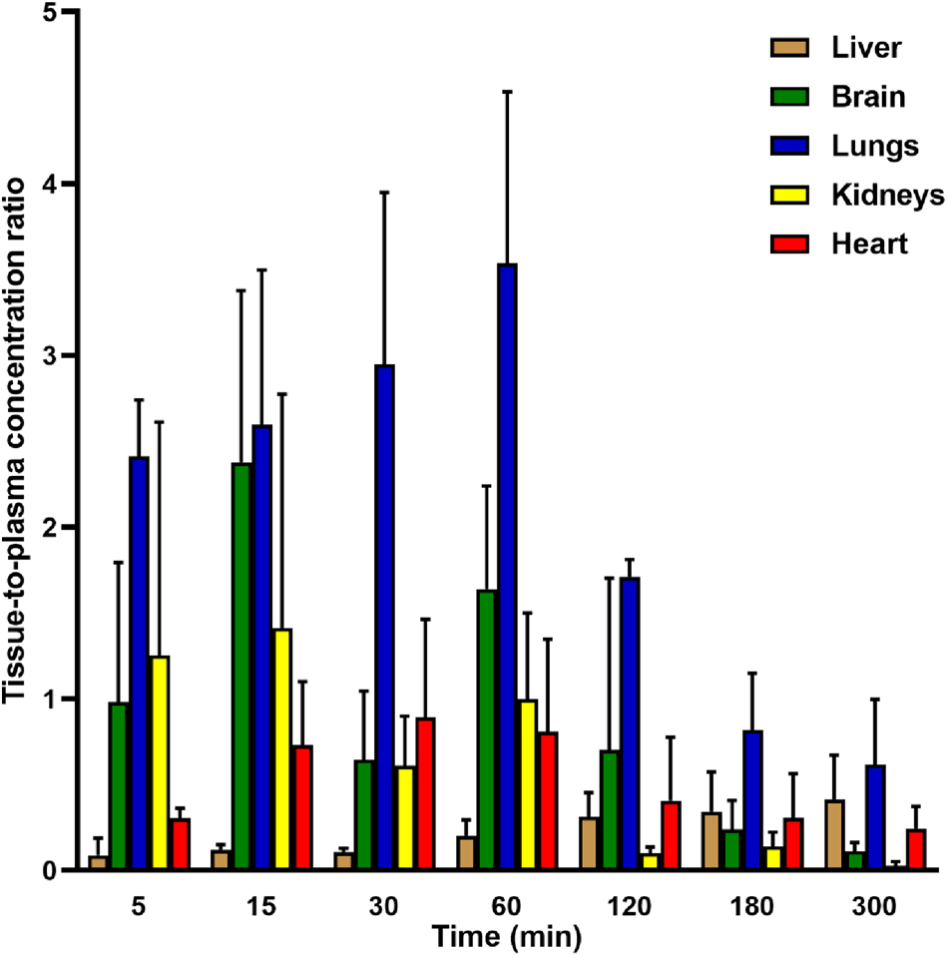
Tissue-to-plasma concentration ratios of **KM-408** following *iv* administration of a dose of 5 mg/kg to rats (*n* = 4)

The values of tissue-to-plasma concentration ratios at each observation time point are presented in Fig. 11.

#### Metabolites’ identification

In the initial step, the identification of **KM-408** metabolites was performed under in silico conditions using Pallas (Pallas CompuDrug) and MetaSite software (Molecular Discovery), then in vivo in rat serum and urine.

The product ion mass spectra of protonated **KM-408** (*m/z* 258.1183) recorded at 40 eV collision energy is shown in Fig. S13 together with proposed structures of the monitored product ions generated by the software ChemBioDraw (PerkinElmer). The differences between the theoretically calculated and experimentally determined masses for **KM-408** product ions were very low, less than 32.7 mDa allowed us to define the chemical structure of the metabolites (Table 15).

**Table 15:** Comparison of calculated and found monoisotopic masses for **KM-408** product ions

Elemental composition	Calculated mass *m*/*z*	Found mass *m*/*z*	Deviation (mDa)
C_7_H_17_N C_9_H_10_ClO C_13_H_20_ClNO	116.1395 169.0520 240.1250	116.1068 169.0416 240.1153	32.7 10.4 9.7

Metabolites were initially extracted from the total ion chromatogram using *m/z* values. To determine the accurate metabolite, signal a mass threshold of 10 ppm was applied. In the next step, the high-resolution fragmentation mass spectra of metabolites were compared with the accurate product ions of **KM-408** [51] using the automated MS/MS comparison tool of ACD/MS Processor [52] and LightSight™ Software [53]. The software screens the ion chromatograms of the expected metabolites according to the predicted gains and losses of metabolite molecular masses compared to the molecular mass of the parent compound. For this reason, exact neutral mass loss and product ion mass filters were used to interrogate data.

**Fig. 12:**
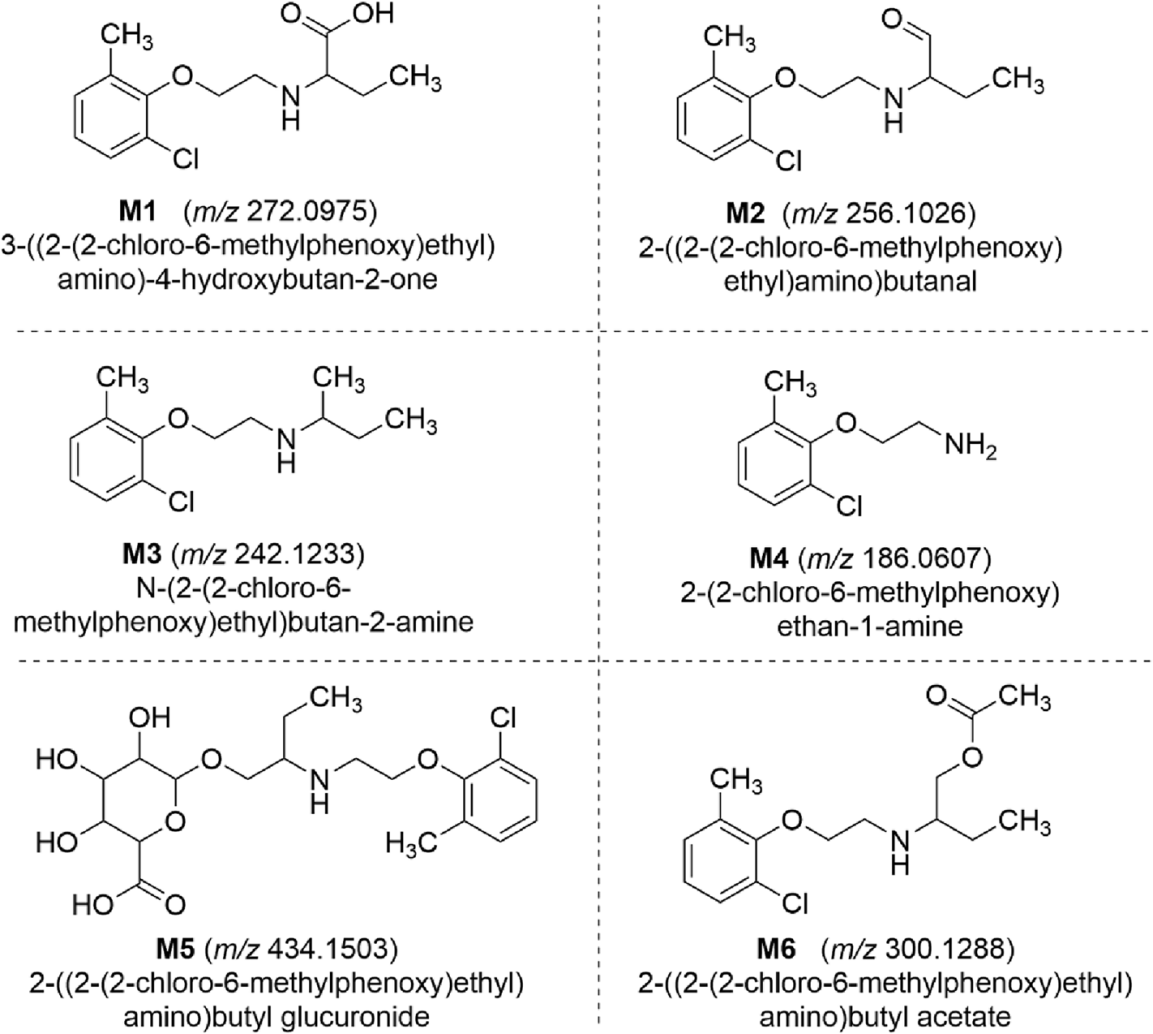
Structures of likely **KM-408** metabolites

Six metabolites of **KM-408** were identified in extracted samples of rat serum and urine, confirming that **KM-408** underwent both phase I and phase II metabolism. As a phase I metabolism, we observed products of oxidation in the side chain (M1, *m/z* 272.0975), side chain oxidation and dehydroxylation (M2, *m/z* 256.1026), dehydroxylation (M3, *m/z* 242.1233), and side chain hydrolysis at nitrogen (M4, *m/z* 186.0607). As the major metabolic pathways of phase II biotransformation, we observed the products of *O*-glucuronidation (M5, *m/z* 449.1738) with characteristic fragments of *m/z* 176.0606 and *m/z* 192.0556 and acetylation (M6, *m/z* 300.1288) with fragments *m/z* 159.1181 (Fig. 12).

## Discussion

Within the course of our research on phenoxyalkylaminoalkanols so far, we have achieved the most promising compounds in the group of 2,6-dimethyl derivatives. The replacement of one methyl substituent with chlorine resulted in a series of antiseizure compounds, of which *R,S*-2*N*-[(2-chloro-6-methylphenoxy)ethyl]amino-1-butanol (**4**), its *R* (**5**) and *S* (**6**) enantiomers, as well as their hydrochlorides (**KM-408**, **5a** and **6**, respectively), were selected for the extended research due to the observed pronounced activity in MES in mice after *ip* administration (**KM-408** MES ED_50_ = 13.3 mg/kg, PI = 4.83).

**KM-408**, its base **4** and enantiomers **5** and **6,** and their hydrochlorides (**5a** and **6a**, respectively) were extensively evaluated for their antiseizure activity, in multiple in vivo tests. The MES test was performed at NINDS as a preliminary screening test for antiseizure activity in the model of *grand-mal* epilepsy, and then each compound was advanced to further studies to assess its activity in other seizure models. Most of the tests were performed starting with the dose equal to ED_50_ obtained in the MES test for the TPE determination.

Kindling models evaluate compounds for activity in seizures which are generated by recurrent kindling of limbic system structures of the brain such as the hippocampus or amygdala. These structures are linked with emotions, therefore, the inhibition of seizures generated there implies inhibition of psychomotor seizures. Therefore, corneal kindling ED_50_ is close to MES ED_50_ due to stimulation of the same organ—the cornea. However, when the organ is not treated with direct proconvulsant stimulus, but with a stimulus—if repeated, increases susceptibility to seizures, it takes a higher dose of the compound to protect the animal. Such a phenomenon is observed when analyzing the results of all kindling in the evaluation of all the compounds, except for corneal kindling of **6** with ED_50_ = 10.50 mg/kg or hippocampal kindling ED_50_ = 15.81 mg/kg compared to MES ED_50_ = 20.8 mg/kg.

The 6 Hz model test was carried out according to the protocol originally described by Brown et al*.* [54] and more recently by Barton et al*.* [55] and Kamiński et al*.* [47] and is a model of pharmacoresistant epilepsy [56]. ED_50_s varied due to the intensity of the current used ranging from 32 to 44 mA. This model was introduced to the NINDS program for the differentiation of profiles of antiseizure.

Frings mice model represents susceptibility to sound-induced seizures and ED_50_ in this model 6.05 mg/kg and it is lower than that for the MES (21.44 mg/kg), probably due to the difference of severity of the stimulus.

On the basis of animal studies’ results, it can be concluded that compound **4** or its hydrochloride **KM-408** might prevent a wide range of epilepsy seizures (including e.g., generalized tonic–clonic seizures—MES test, focal seizures—6 Hz test or drug-resistant seizures—LTG-resistant seizures model).

An important element of the research was the *iv* PTZ test, which allowed the assessment of the effect of tested compounds on the seizure threshold. Among antiseizure, mexiletine is known to have internal pro-convulsant properties (with the simultaneous activity of this compound in the MES test, cited in Fig. 1) [57]. Due to this fact, intravenous PTZ infusion is performed as a test for a compound administered before PTZ order to examine whether the compound causes or prevents seizures at higher doses of PTZ. Normally, PTZ induces seizures and the lowering of seizure-causing dose of PTZ means proconvulsant properties, and increasing of seizure-causing dose of PTZ means antiseizure properties (lack of similarity to mexiletine properties). Of all compounds tested, only compound **4** showed activity in lowering the seizure threshold in this test. This effect was not shown either by the individual enantiomers of compound **4**—compounds **5** and **6**, or—surprisingly—by the hydrochloride of compound **4** (**KM408**). The obtained results indicate that in the course of further development of compound **4** toward the treatment of epileptic patients, the influence of this compound on the seizure threshold should be carefully examined. An alternative may be the use of its enantiomers in therapy. The lowering of the seizure threshold by a compound, however, is not a factor in excluding the racemate from the treatment of other diseases, as numerous drugs available on the market have a documented potential to induce seizures [58, 59], including overdosed or inappropriately used antiepileptics (e.g., valproic acid or carbamazepine) [60, 61].

The analgesic activity of the previously obtained compounds (e.g., reference compounds **IV** and **V**, Fig. 1B), was the starting point for further studies of selected compounds in various models of pain. First, the formalin test was performed to evaluate the potential analgesic activity of test compounds, i.e., their effect on neurogenic and inflammatory pain due to formalin administration. Then, the analgesic activity of **KM-408** was assessed in two rodent models of chronic, neuropathic pain, namely in SNL model in rats and in the diabetic neuropathic pain model in mice. Interestingly, for **KM-408** the TPE in the SNL model was 2 h, while its TPE in antiseizure activity tests (MES, LTG-resistant seizures, 6-Hz test etc.) was 0.25 h. It is also noteworthy that *R* enantiomer **5a** exhibited activity at 4 mg/kg in this model in the von Frey test, with TPE = 1 h. In the mouse diabetic neuropathic pain model, **KM-408** showed a statistically significant analgesic activity at 1 mg/kg in the von Frey test and at 10 mg/kg also in the hot plate test. Taken together, this demonstrates that **KM-408** is able to attenuate tactile allodynia and (at higher dose ranges) also heat hyperalgesia.

Test compounds, in particular, **KM-408** and its enantiomers were also evaluated in other pain tests and pain models. This part of the in vivo research confirmed potential antinociceptive properties of the test compounds in the capsaicin test, formalin test, hot plate test as well as hot plate test after streptozotocin-induced diabetic neuropathy at doses 10–30 mg/kg. In addition to this, in models of local anesthesia, **KM-408** showed local anesthetic properties in mice (concentrations 0.5% and 2%) and guinea pigs (concentrations 0.125% and higher).

Selected tests were performed for both **KM-408** and its enantiomers. This makes it possible to discuss the influence of configuration on analgesic activity. In the case of formalin test (first screening, Table 3), enantiomer *S* (**6a**) showed comparable activity with the racemic mixture (**KM-408**), exhibiting activity in the acute phase of pain (*ca.* 30% of effect). The results for enantiomer *R* (**5a**) were not statistically significant. Extended research at lower doses (Table 4) confirmed the advantageous properties of enantiomer *S*. Additionally, it is worth noting that in general the tested compounds were more active in I (acute) phase of the formalin test. The only exception was compound **6** at 20 mg/kg, showing *ca.* 57% control in II phase of this test. In the tail immersion test in mice (Table 5) the most advantageous properties were shown for enantiomer *S* (**6a**), prolonging the time reaction by 348.6% at 0.125% concentration. Despite the significant activity at this concentration of the *R* enantiomer (**5a**) as well, the administration of the racemic mixture resulted in an increase in the active concentration to 0.5%. In models of local anesthesia in guinea pigs all tested compounds were active at 0.125% concentration (Tables 6, 7). However, the use of racemic mixture **KM-408** caused the most pronounced and prolonged effect (in the case of infiltration anesthesia, statistically significant analgesia was observed up to 240 min after administration).

Considering that in neuropathic pain treatment antiseizure, antidepressants, and local anesthetics are widely used as effective analgesics, local anesthetic properties of **KM-408** seem to be important in terms of the potential use of this compound in this clinical condition [49].

Considered the ratio between ED_50_s achieved for *po* and *ip* administration, we assumed that the relative bioavailability of **KM-408** could range from *ca.* 37% in mice to *ca.* 11% in rats. Having regard to the inaccuracy of the calculations due to the omission of the permeability through the peritoneum, we were waiting for proper *po*/*iv* comparison. In turn, the pharmacokinetic analysis revealed that the absolute bioavailability of this compound was dose-independent and ranged from 3.6 to 3.9% (Table 13) indicating an extensive first-past metabolism both in the gastrointestinal tract and the liver. These results were surprising, however, using all the PK data—were self-explanatory. Moreover, we noticed that the confidence intervals in the same tests but with various sex of animals do not cover: in MES CI for male mice is (18.63–25.32) and for female mice is (10.55–12.47). Not covering the CIs means that there is a significant difference in the mode of action. Such differences in sex for neurological activity could be explained by the antiseizure activity of the female sex hormone allopregnanolone known in MES [47]. The same phenomenon was observed for lead compound **V** (KM113) [15].

The study of the mechanism of activity included a wide panel of receptor studies (Table 2, Table S1, and Fig. S3 in Supplementary material), which revealed IC_50_ equal to 8.9*10^–8^ M for the σ receptors and IC_50_ values of the order of 10^–6^ for the 5-HT_1A_ and 5-HT_2B_ receptors as well as 5-HT transporter. It should be mentioned that the receptor studies also included voltage-gated Na^+^ channels, which are a model molecular target for both antiepileptic and analgesic drugs. The obtained IC_50_ value for **KM-408** (1.5*10^–5^ M) was, however, relatively low compared to the molecular targets mentioned above.

σ1 receptors are connotated with neuropathic pain pathophysiology—their expression may be altered in various ways under the influence of pathological pain mechanisms. For example, it was found to increase in the spinal cord in the early phase of neuropathic pain induced by loose ligation of the sciatic nerve in rats [62] and decrease in cytostatic drug-induced neuropathic pain models (oxaliplatin, paclitaxel) [63]. Research results suggest the potential use of σ1 antagonists in the treatment of various types of neuropathic pain [64–68]. Of particular note is E-58862, a selective antagonist with analgesic activity in numerous models of neuropathic pain, which has become the subject of clinical trials [69, 70]. There is also a known agonist of the σ2 receptor (UKH-1114, a benzomorphan derivative with high selectivity for σ2 receptors), active in a spared nerve injury model (SNI) in mice [71].

The mechanism of the antiseizure and analgesic activity of **KM-408** can be both common and disjoint. Radioreceptor studies in conjunction with the analysis of the literature indicate a high probability of the participation of σ receptors in the analgesic in neuropathic pain activity, however, the involvement of other molecular targets, e.g., serotonin receptors, voltage-gated Na^+^ channels blocking properties or adrenoceptors (particularly in case of high doses tested) cannot be ruled out. To complete the discussion on the mechanism of action, the results of receptor screening should be confronted with the data obtained from pharmacokinetic studies. **KM-408** concentration in rat brain 2 h after *iv* administration (TPE for **KM-408** in the SNL model in rats, *ip*) was 64.86 ng/g. Assuming the tissue density of 1 g/mL, this value can be approximated as 2.2*10^–7^ M and compared with IC_50_ values obtained in receptor studies. Apart from the σ receptor IC_50_ value (8.9*10^–8^ M), IC_50_ values obtained for all other targets significantly exceed the compound concentration in the brain. Considering the approximate calculation and a different route of administration, the participation of σ receptors in the mechanisms of **KM-408** analgesic activity still seems to be the most probable.

The potential mechanism of local anesthetic activity should be considered separately. Due to the high concentration of test compounds at the site of administration, the observed affinity for sodium channels for **KM-408** (Table 6 and Table S3 in Supplementary material) and the results of patch-clamp studies for its basic form (compound **4**, Table S2 in Supplementary information), the most likely mechanism of action is modulation of voltage-gated Na^+^ (Na_v_) channels.

In the case of antiseizure activity, the likely mechanism of action may be the aggregate effect of **KM-408** on selected molecular targets, including e.g., serotonin receptors, serotonin transporter or voltage-gated Na^+^ channels blocking properties in higher doses. The literature review confirms the role of the serotonergic system in the pathophysiology and therapy of both neuropathic pain and epilepsy (the issue was discussed earlier [72]), while the Na^+^ channel is a common molecular target for antiseizure. The possible interaction of **KM-408** with molecular targets that were not covered by the receptor research panel should also be considered.

The safety pharmacology panel performed for **KM-408** covered the evaluation of potential effects on respiratory (no significant effects of **KM-408** on the tested parameters) and cardiovascular systems. An intravenous administration of **KM-408** resulted in a transient decrease in blood pressure and in significant and long-lasting bradycardia, accompanied with PQ interval prolongation immediately after administration. After oral administration at a high but not a low dose, **KM-408** caused also marked bradycardia. The observed effect on the heart rate and ECG record may be explained with voltage-gated Na^+^ channels blocking properties (observed for compound **4**, the base form of **KM-408**) as voltage-gated sodium channel blockers exert a potent bradycardic effect in the rat [73] and may prolong atrio-ventricular conduction time, what manifests in PQ interval prolongation in ECG record. Among all voltage-gated sodium channels, Na_v_1.5 isoform is highly expressed in all types of cardiac myocytes, including the sinus node, the conduction system, atrial and ventricular myocytes [74]. However, it is known that also Na_v_1.1, Na_v_1.6, and Na_v_1.7 isoforms are all present in rat sinoatrial node, and contribute more to its activity [75]. The results from the in vitro studies on cloned Na_v_ channels showed that **KM-408** potently inhibited Na_v_1.1–1.8 currents, with selectivity for blockade of the inactivated state. The ability of **KM-408** to block Na_v_ channels is probably responsible for the observed bradycardia. The above remarks can be supported by analysis of the results of pharmacokinetic studies. Both decreases in blood pressure and bradycardia were observed immediately after administration of **KM-408** to rats (5 mg/kg, *iv*). **KM-408** plasma concentration under the same conditions and time (1.48*10^–5^ M, Table 12) did not exceed but was comparable to the **KM-408** IC_50_ value obtained for Na_v_ channels (1.5*10^–5^ M). Considering the inaccuracy of receptor studies, the participation of Na_v_ channels in the mechanism of the observed hypotension and bradycardia is the most probable.

Moreover, **KM-408**, similarly to mexiletine [76], did not prolong the QRS complex as well as the QT interval, which suggests that it did not influence ventricular depolarization and repolarization at tested doses.

It is also worth noting that in the case of the hERG receptor, **KM-408** showed 26.1% of control specific binding, which bodes well for the cardiovascular safety of tested compounds (50% is considered significant). Similar results were obtained also for compounds **5a** and **6a** (23.9% and 20.9% of control specific binding, respectively, Supplementary material, Table S1) [77]. On the other hand, **KM-408** showed affinity to 5-HT_2B_ receptors. Agonists of this receptor (e.g., some ergot alkaloids [78]) are at risk of aortic valve insufficiency, thickenings, and valve flexibility disorders via extensive fibrosis [79].

Toxicity studies revealed the safety of **KM-408**. LD_50_ values significantly exceed the doses of antiseizure and analgesic activities. In vitro tests showed no significant cytotoxic activity (MTT test) or influence on the proliferation (crystal violet test) of astrocytes, as well as no mutagenic activity (Ames test, Table 11). However, **KM-408** may be at risk of drug–drug interactions due to the observed inhibitory activity of selected cytochrome P450 isoenzymes, including in particular CYP1A2, CYP2A6, CYP2C19, and CYP2D6 (Table 12).

As shown in Table 13, after *iv* dosing the values of *C*_max_ and AUC increased in a disproportionate manner with increasing dose, indicating non-linear pharmacokinetic behavior of the studied compound. Both terminal half-life and clearance were different following the administration of doses 1, 5, and 10 mg/kg confirming this observation. The total clearance decreased with increasing doses suggesting saturation of the elimination process of **KM-408** from the rat body. The volume of distribution significantly exceeded rat body water indicating an extensive tissue distribution of **KM-408**. The peak concentration and AUC following oral administration of the higher dose (100 mg/kg) were almost twofold higher than the values of these parameters observed after the dose of 50 mg/kg. Thus, the processes involved in the absorption, distribution, and elimination of the studied compound after oral administration do not seem saturated. This may be connected with the fact that, in comparison with *iv* dosing, concentrations of **KM-408** attained in plasma are relatively low, probably due to the first-pass metabolism in the gastrointestinal tract and the liver. The elimination half-lives following both oral doses are quite close to those assessed following *iv* administration. This may indicate that absorption from the gastrointestinal tract is fast and complete. The absolute bioavailability (*F*) calculated based on the AUC_0-t_ for the two lower *iv* doses is low and dose-independent. As a result, oral clearance (CL/*F*) and volume of distribution (*V*_z_/*F*) are much higher than those estimated following *iv* dosing.

From Table 14, **KM-408** attained relatively high concentrations in all studied tissues at the first observation time point, i.e., 5 min., with the exception of the liver, where the peak concentration was more than 10 times lower than *C*_max_ observed in the brain, lungs, and kidneys. These may indicate that **KM-408** is extensively metabolized in this organ. The concentrations of the compound under investigation in the heart tissue were also much lower than in the tissues mentioned above and, in addition, the peak concentration in the rat heart was reached later than in other tissues (*t*_max_ = 15 min). The calculated tissue-to-plasma AUC ratios (*K*_p_) followed the same pattern as the values of *C*_max_ reaching the highest value for lung tissue. The elimination rates of **KM-408** from all tissues was were similar to those observed in plasma. The only exception was kidneys, where the terminal half-life was two times shorter than those in plasma and other tissues tested. Similarly, MRT was the shortest in this organ and the highest values of this parameter were observed in the liver.

As expected, tissue-to-plasma concentration ratios (Fig. 11) were very low for the liver and the heart, and the highest values were noted for the lungs. Interestingly, these ratios decreased with time in all investigated tissues, except the liver, where they increased with time, reaching the highest value in the last measured point, i.e., at 300 min. This may support the previous suggestion that, following *iv* administration, the metabolism of **KM-408** may be saturated with higher doses.

For **KM-408**, six major metabolites of phase I and phase II were identified. Side-chain oxidation, dehydroxylation, *N*-dealkylation, *O*-glucuronidation, and acetylation processes followed by conjugation reactions with glucuronic acid and acetic acid, respectively seem to be the main biotransformation pathway for **KM-408**, possibly contributing to the low bioavailability following oral dosing of this compound as a result of significant presystemic metabolism.

The stability studies of drug products and drug substances include long-term studies (12 months) and accelerated stability studies (6 months). Forced degradation is a process that involves degradation at conditions more severe than accelerated conditions and, thus, generates degradation products that can be studied to determine the stability of the molecule which helps in the development of formulation and package [80]. In forced degradation studies of drugs, besides hydrolysis in acidic and basic solutions and verification of photostability, evaluation of the influence of oxidizing and reducing agents has been recommended [45]. Within the performed forced degradation studies **KM-408** showed stability in most applied conditions. Basic conditions at 70 °C caused the compound’s decomposition with a degradation rate constant equal to 0.83·10^–3^ h^−1^ and a half-life equal to 834.9 h.

In conclusion, a number of 2-chloro-6-methylethyl- or 2-chloro-6-methylacetyl derivatives with antiseizure activity were obtained. The extended research conducted for selected compounds made it possible to select **KM-408** (*R,S*-2*N*-[(2-chloro-6-methylphenoxy)ethyl]amino-1-butanol hydrochloride) as a potential antiseizure and analgesic compound for the treatment of neuropathic pain. The most probable mechanism of action for **KM-408** is the modulation of σ receptors’ activity, however, its influence on 5-HT_1A_, and 5-HT_2B_ receptors, and the 5-HT transporter should be also noted. The compound shows a promising safety profile in vitro and in vivo. A challenge in the context of its further development may be the surprisingly low bioavailability after oral administration and the risk of interactions due to inhibition of the activity of selected cytochrome P450 enzymes.

## Supplementary Information

Below is the link to the electronic supplementary material.

Supplementary file1 (DOCX 12181 KB)

Supplementary file2 (PDF 11580 KB)

Supplementary file3 (PDF 5755 KB)

Supplementary file4 (PDF 5760 KB)

Supplementary file5 (PDF 4814 KB)

Supplementary file6 (PDF 5916 KB)

Supplementary file7 (PDF 534 KB)

Supplementary file8 (DOCX 2424 KB)

## Data Availability

Data generated/analysed during this study are included in this published article (and its supplementary information files) or are available from the corresponding author on reasonable request.

## Abbreviations

*λ*_z_: Terminal slope
AUC_0-t_: Area under the concentration–time curve (AUC) from the time of dosing to the last measured data point
AUC_0-∞_: Area under the concentration–time curve (AUC) from the time of dosing to infinity
*C*_0_: Initial concentration
CL: Clearance
*C*_max_: Peak concentration
ECG: Electrocardiogram
ED_50_: Median effective dose
ETSP: Epilepsy Therapy Screening Program
*F*_a_: Absolute bioavailability
IC_50_: Half maximal inhibitory concentration
K_i_: Inhibition constant
K_p_: Tissue-to plasma AUC ratio
LD_50_: Median lethal dose
LTG: Lamotrigine
MES: Maximal electroshock seizure
M.p.: Melting point
MRT: Mean residence time
MTT: 3-(4,5-Dimethylthiazol-2-yl)-2,5 diphenyl tetrazolium bromide
NIH: National Institutes of Health
NINDS: National Institutes of Neurological Disorders and Stroke
PI: Protective index
PTZ: Pentylenetetrazole
SNI: Spared nerve injury
SNL: Sciatic nerve ligation
STZ: Streptozotocin
*t*_0.5λz_: Terminal half-life
TD_50_: Median toxic dose
*t*_max_: Time to reach C_max_
TPE: Time to peak effect
*V*_z_: Volume of distribution based on the terminal phase
