# Supplementary material for: KM-408, a novel phenoxyalkyl derivative as a potential anticonvulsant and analgesic compound for the treatment of neuropathic pain
Source: Pharmacol Rep. 2022 Nov 19;75(1):128–65. doi: 10.1007/s43440-022-00431-7 (PMC9889419; doi:10.1007/s43440-022-00431-7)

**Compound 1**

LCMS


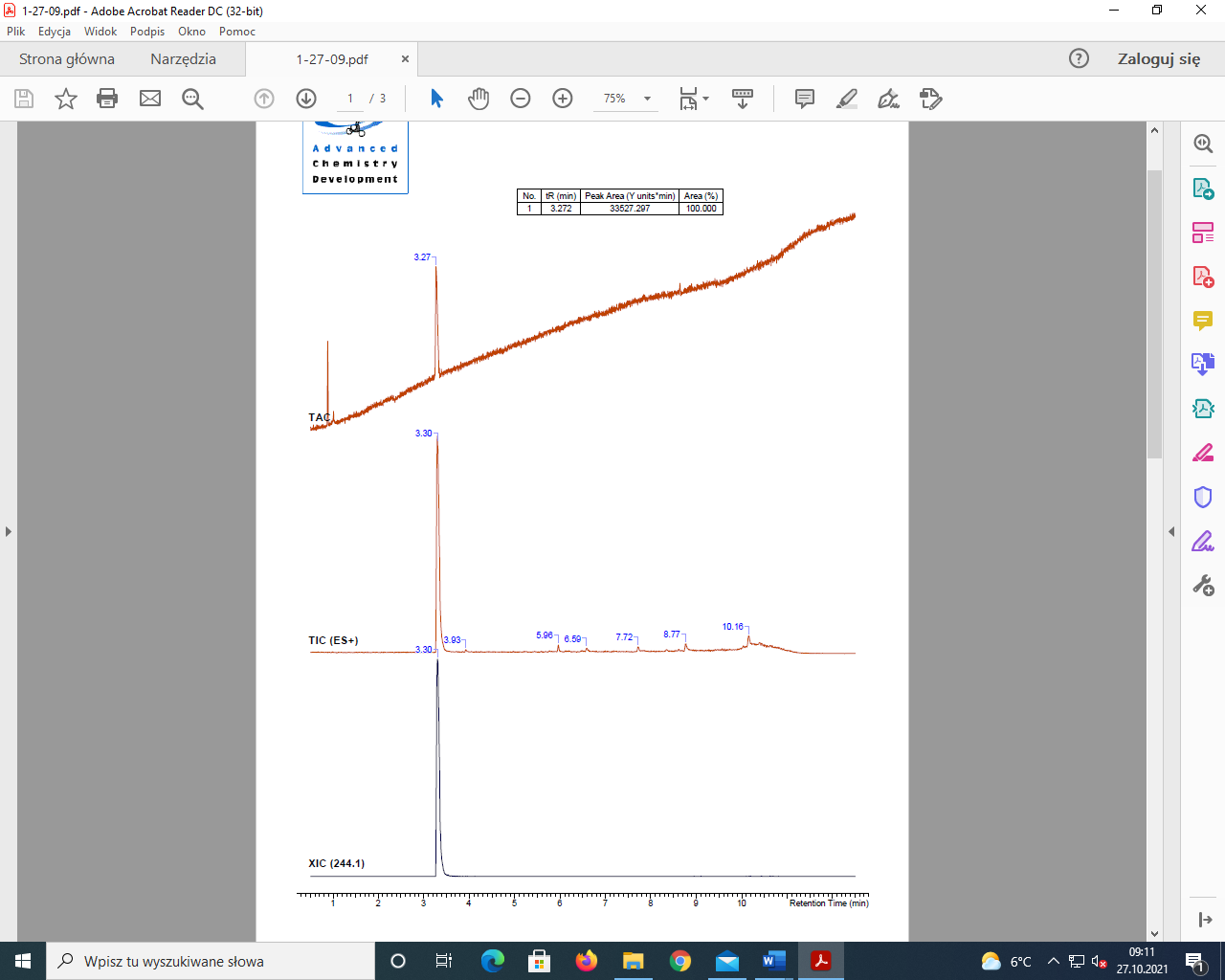


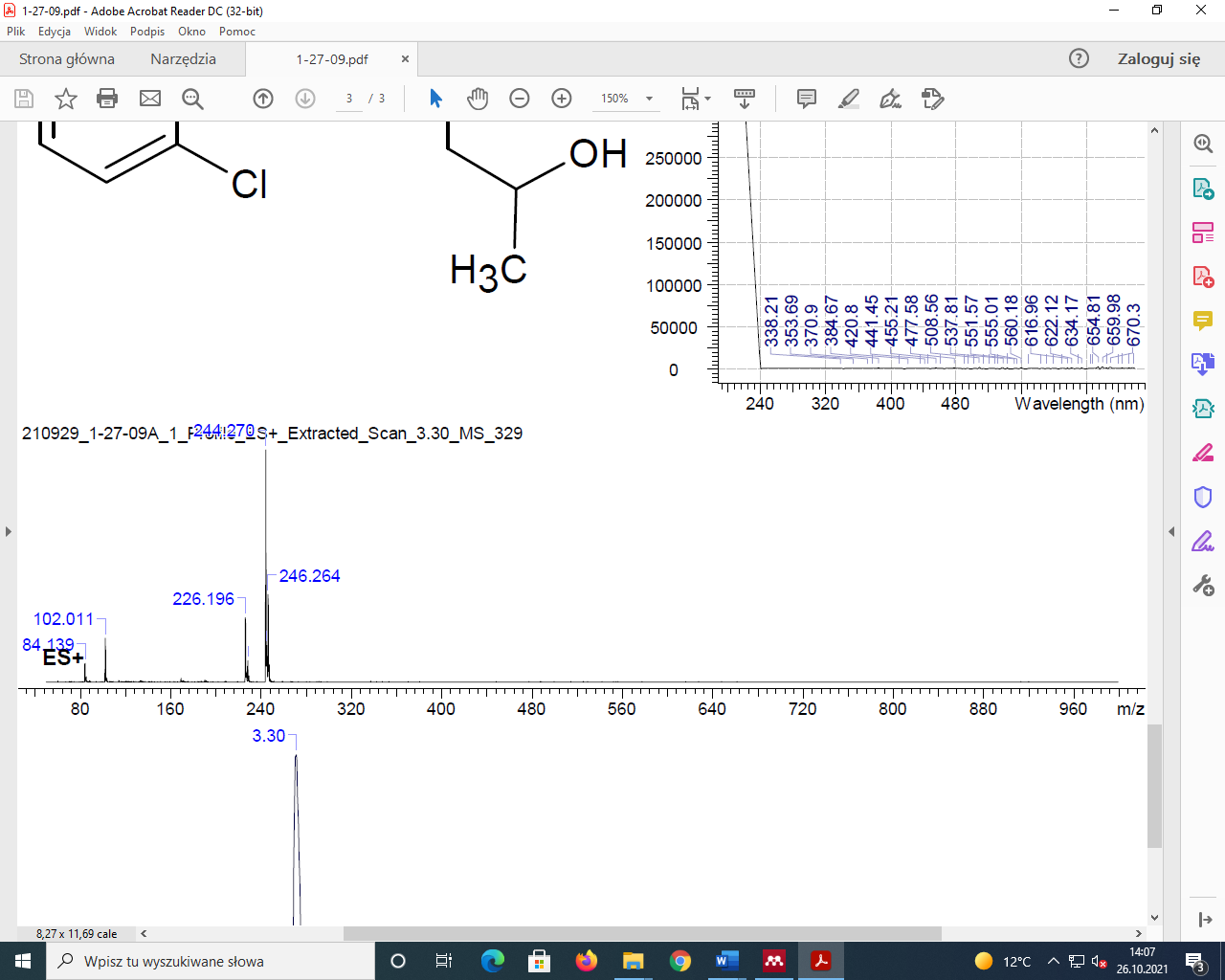


^1^HNMR


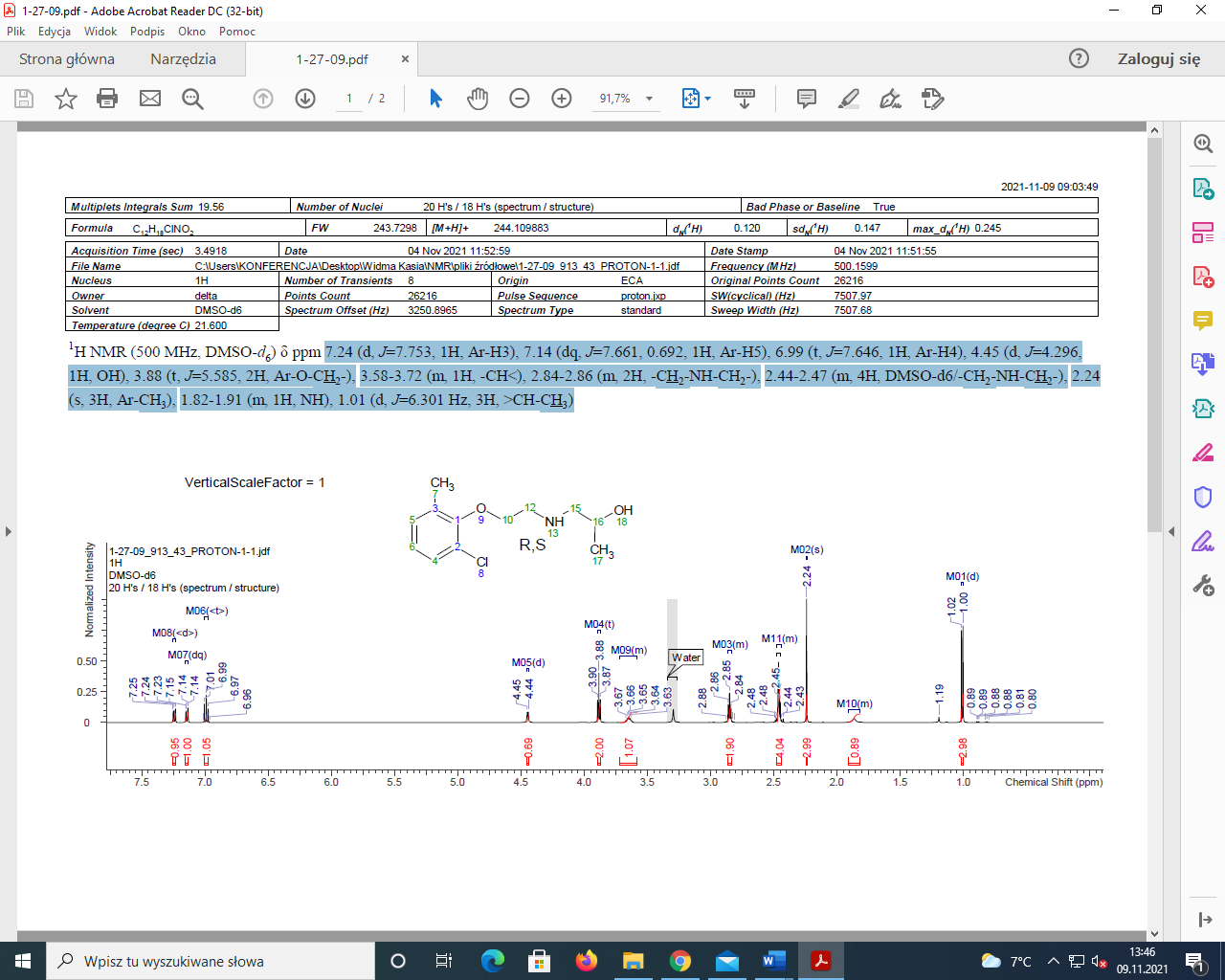


**Compound 2**

LCMS


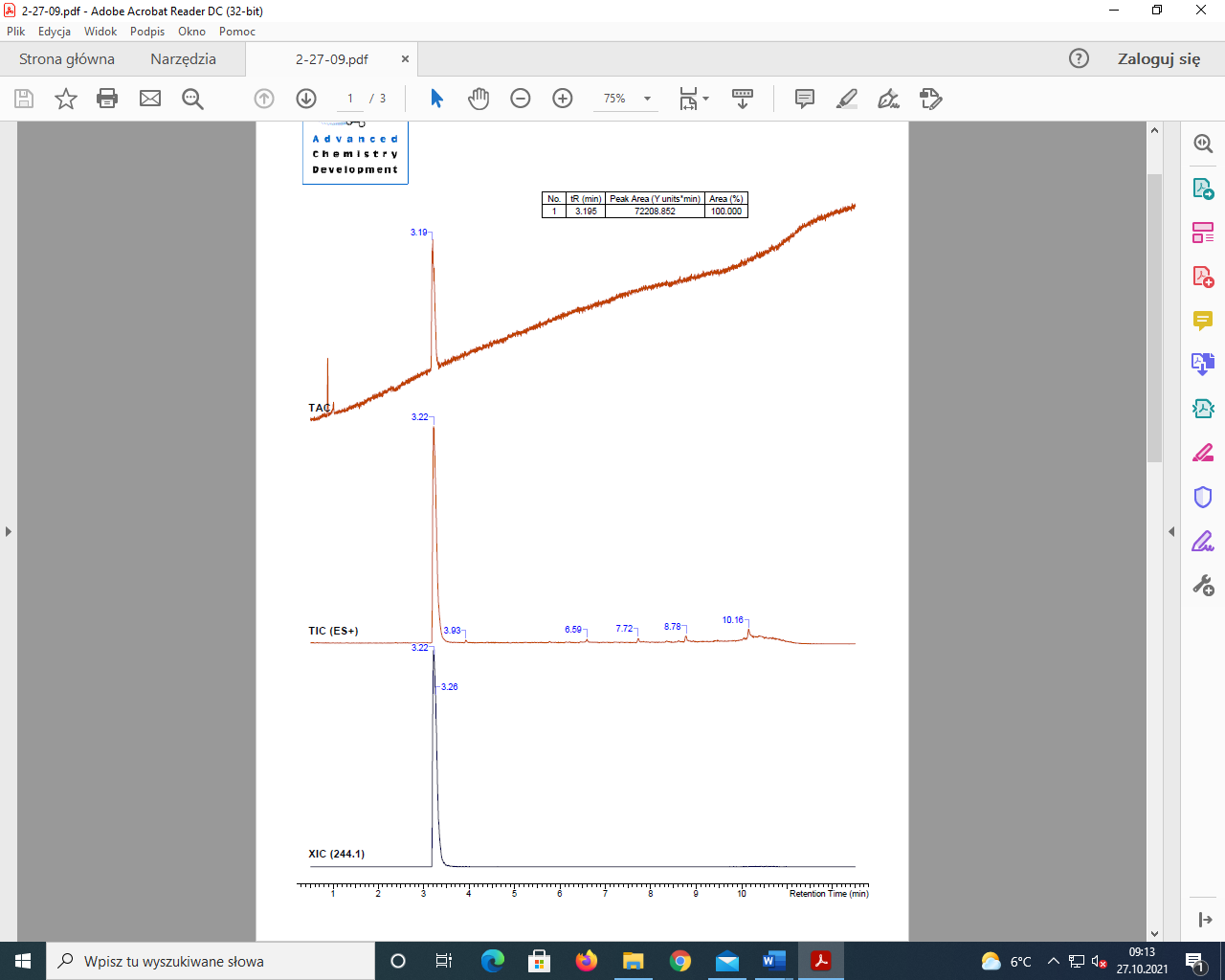


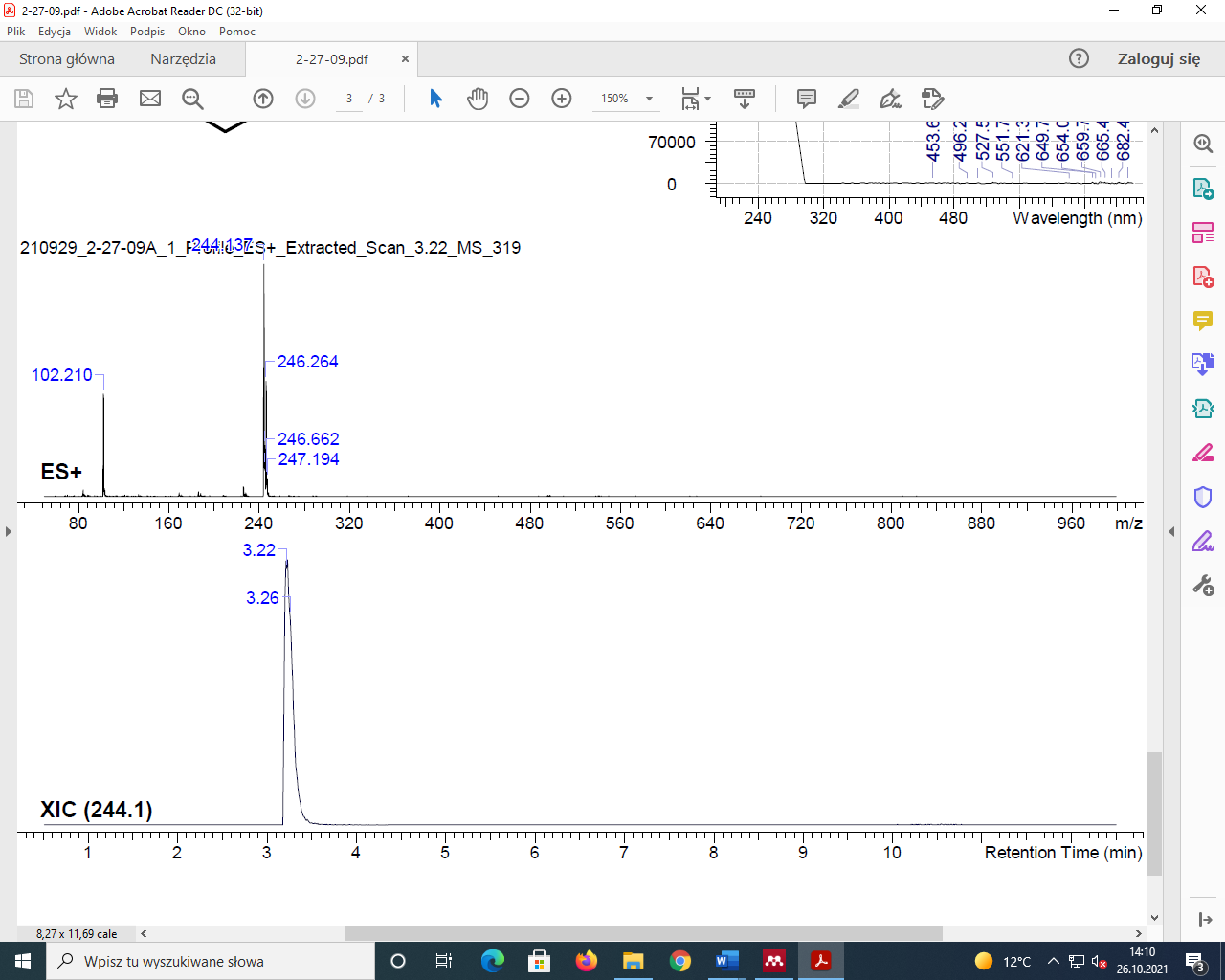


^1^HNMR


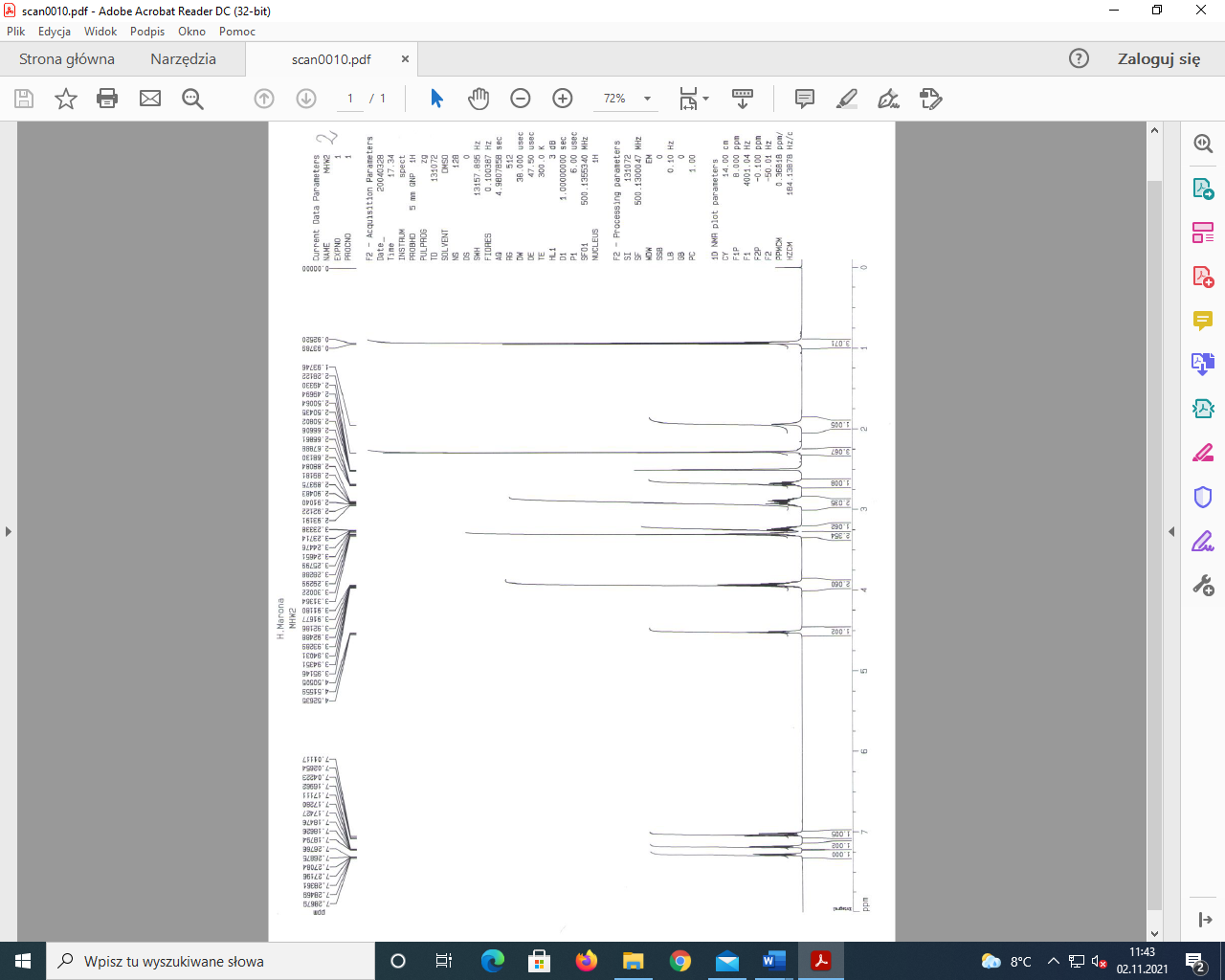


**Compound 3**

LCMS


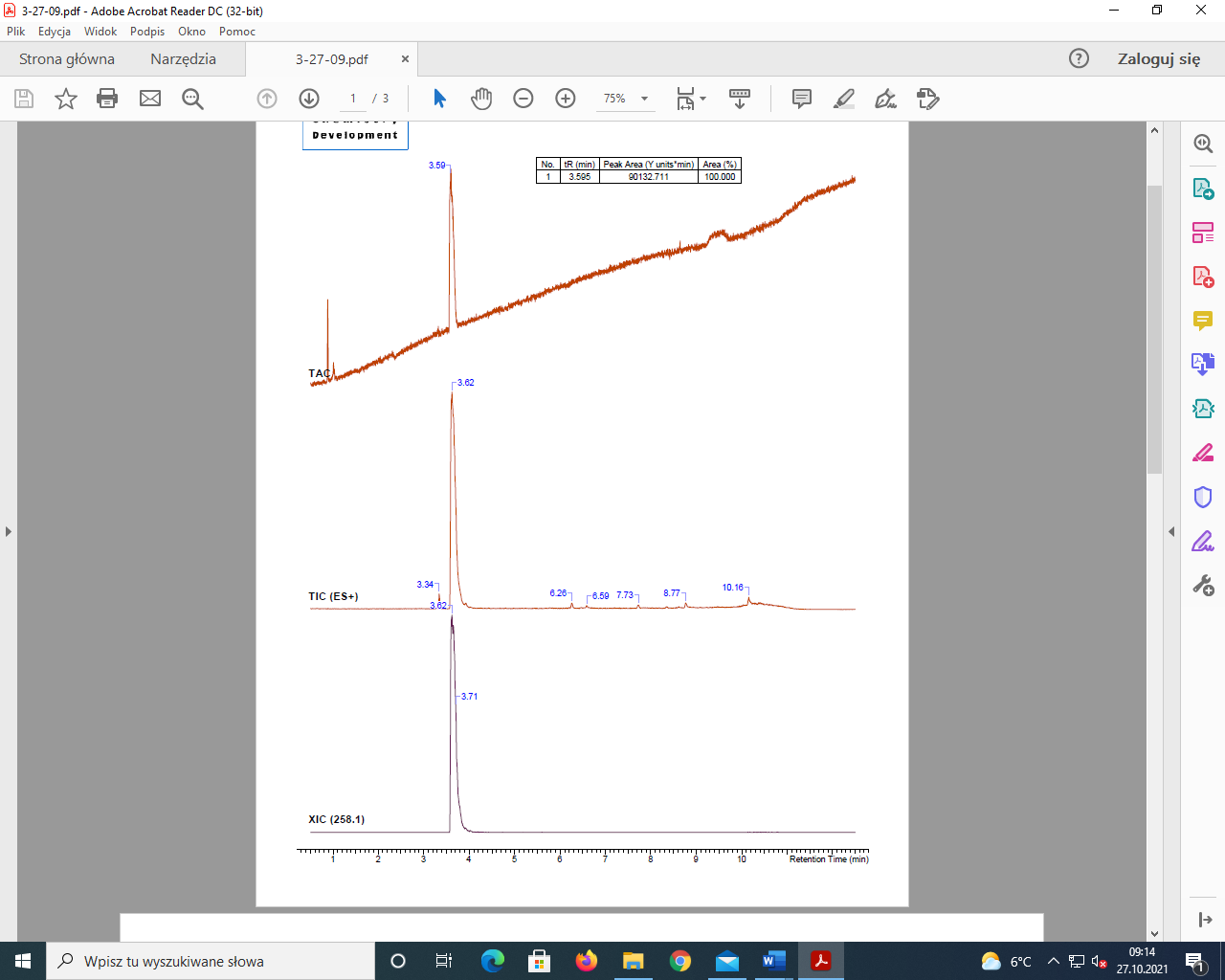


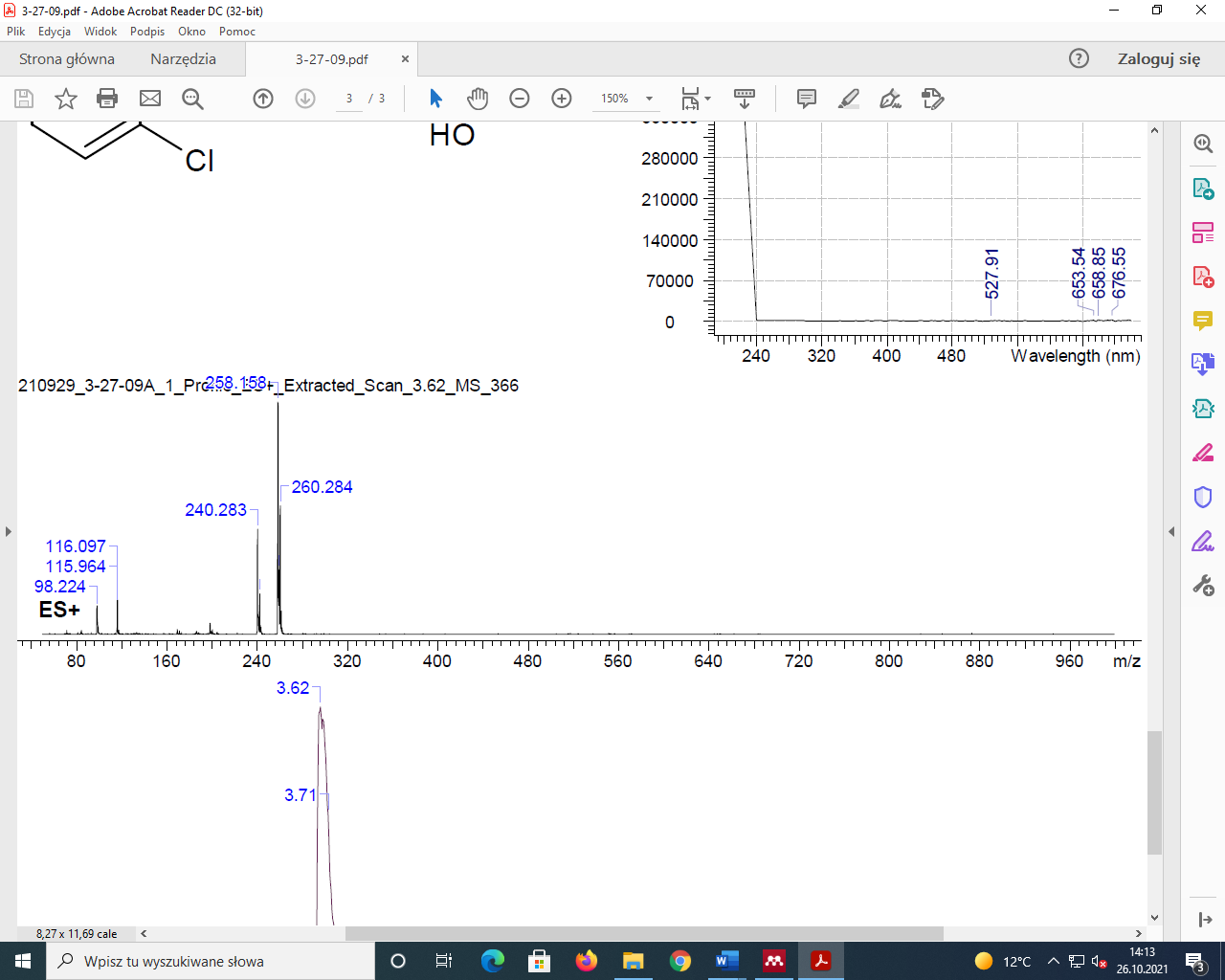


^1^HNMR


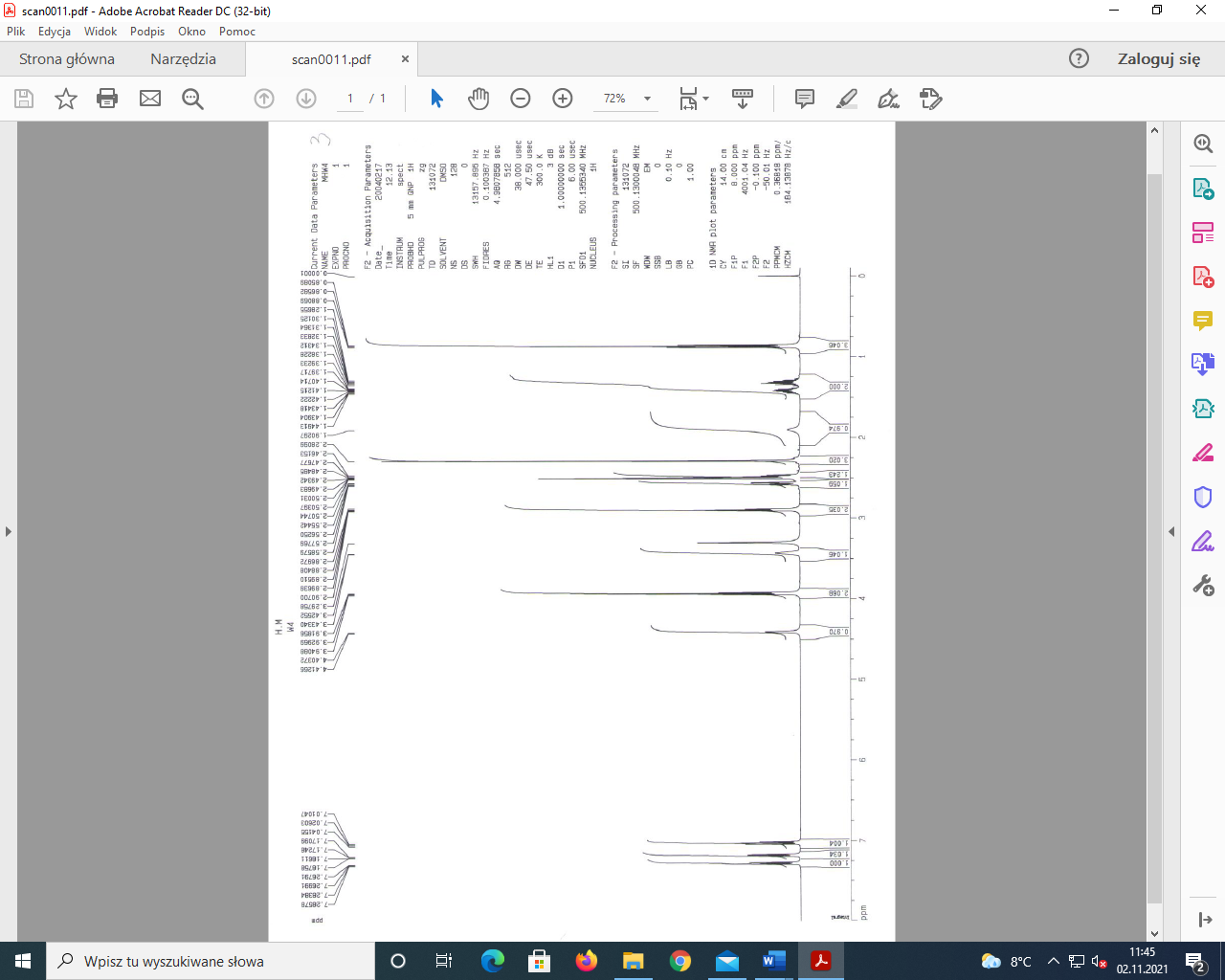


**Compound 4**

LCMS


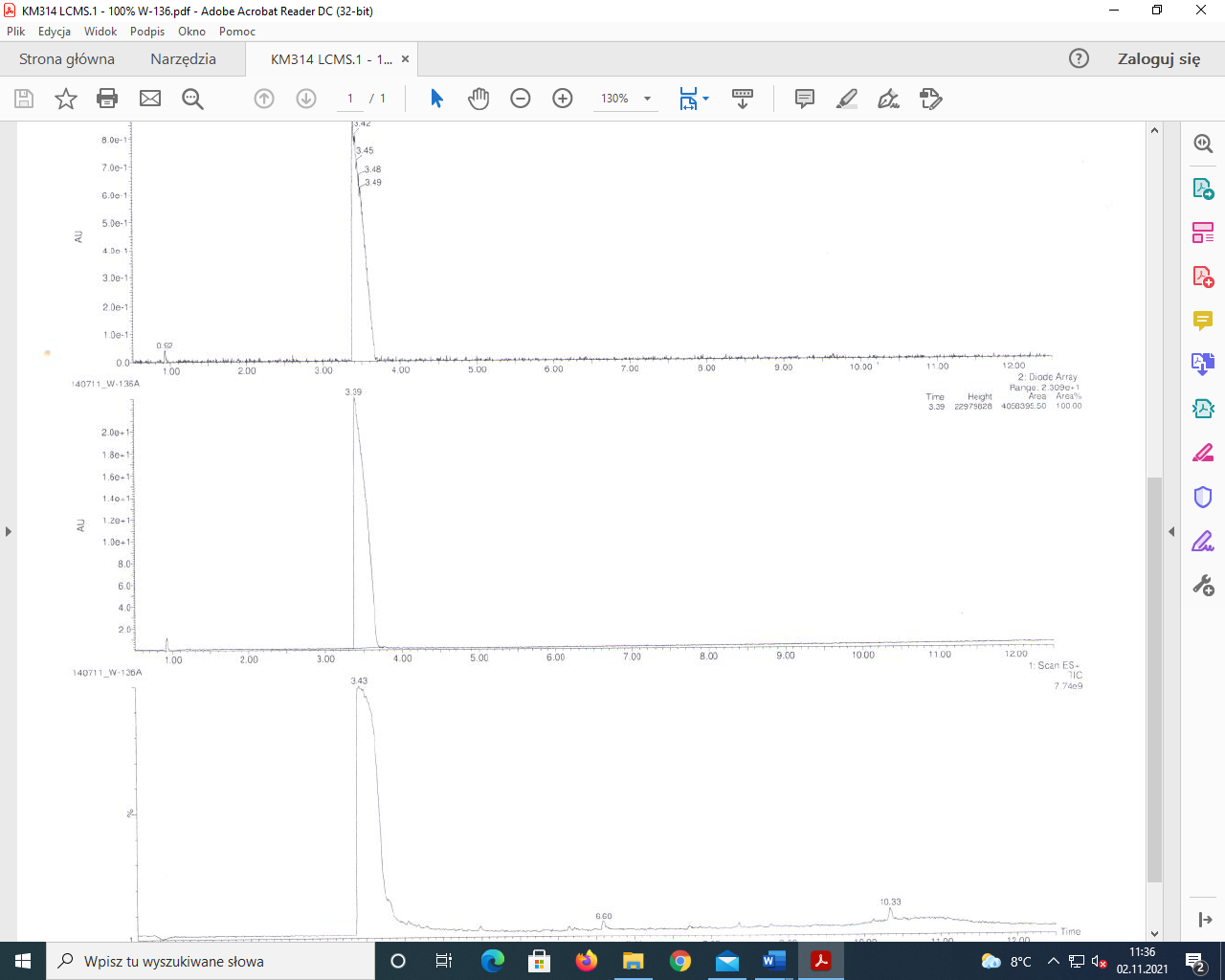


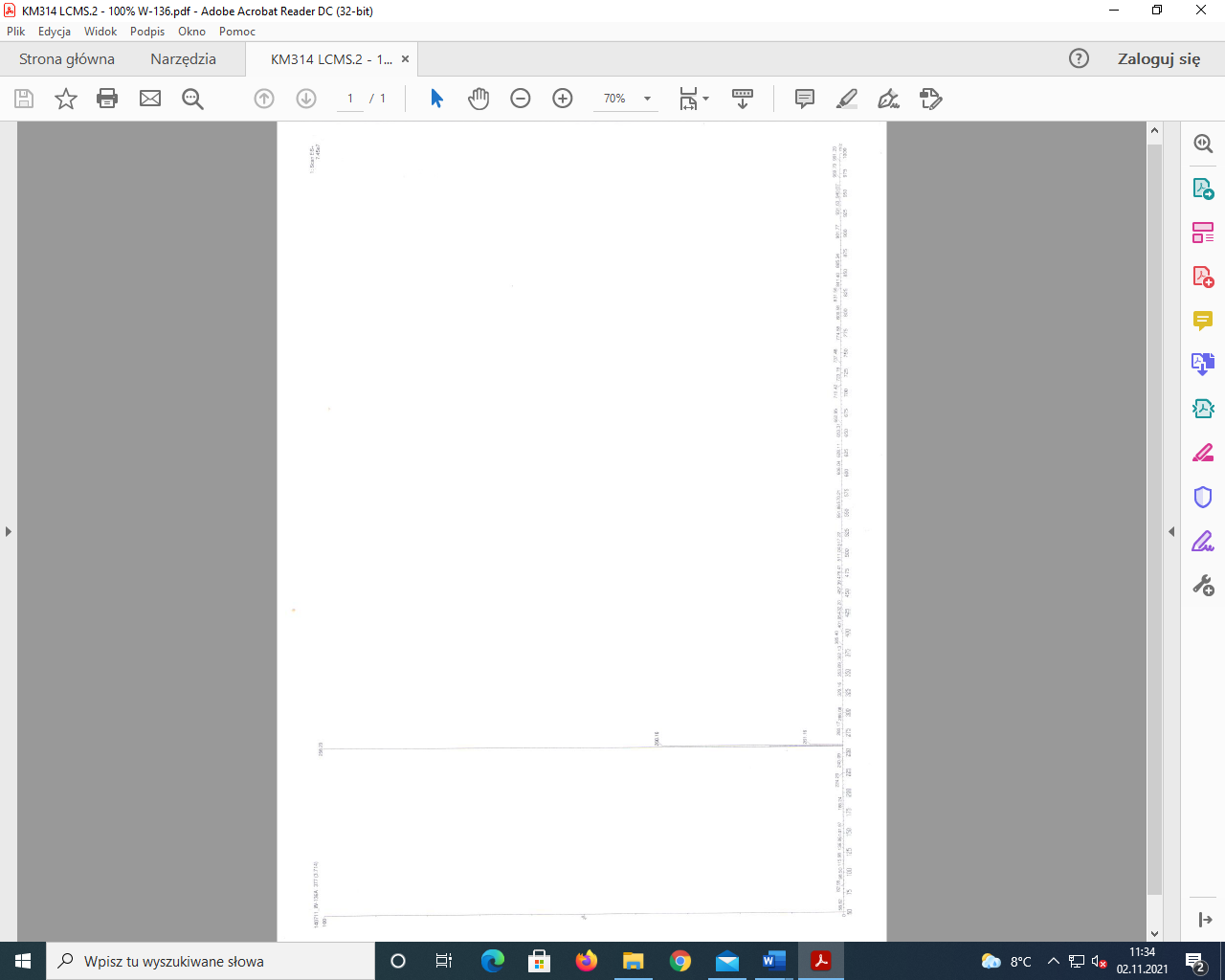


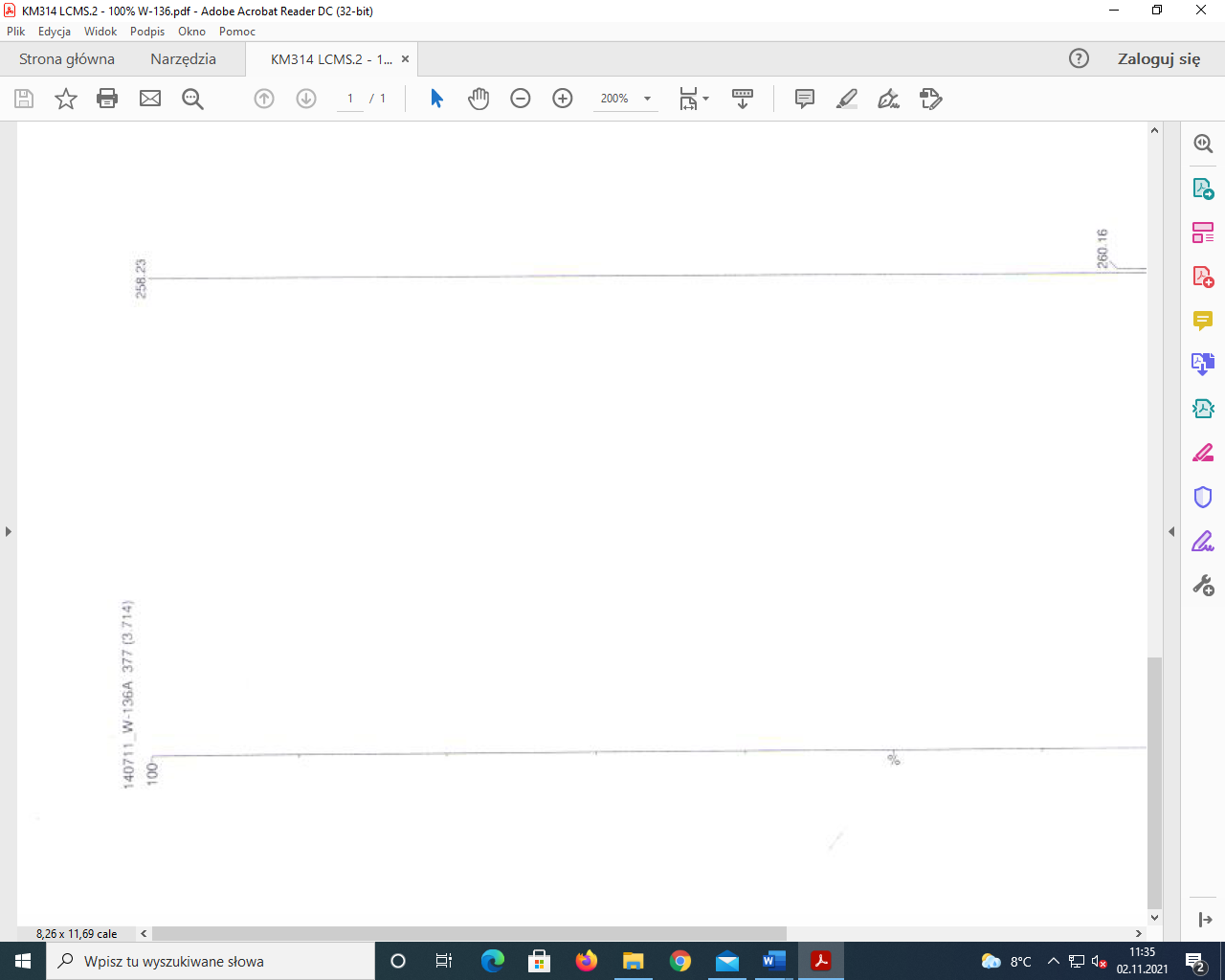


^1^HNMR


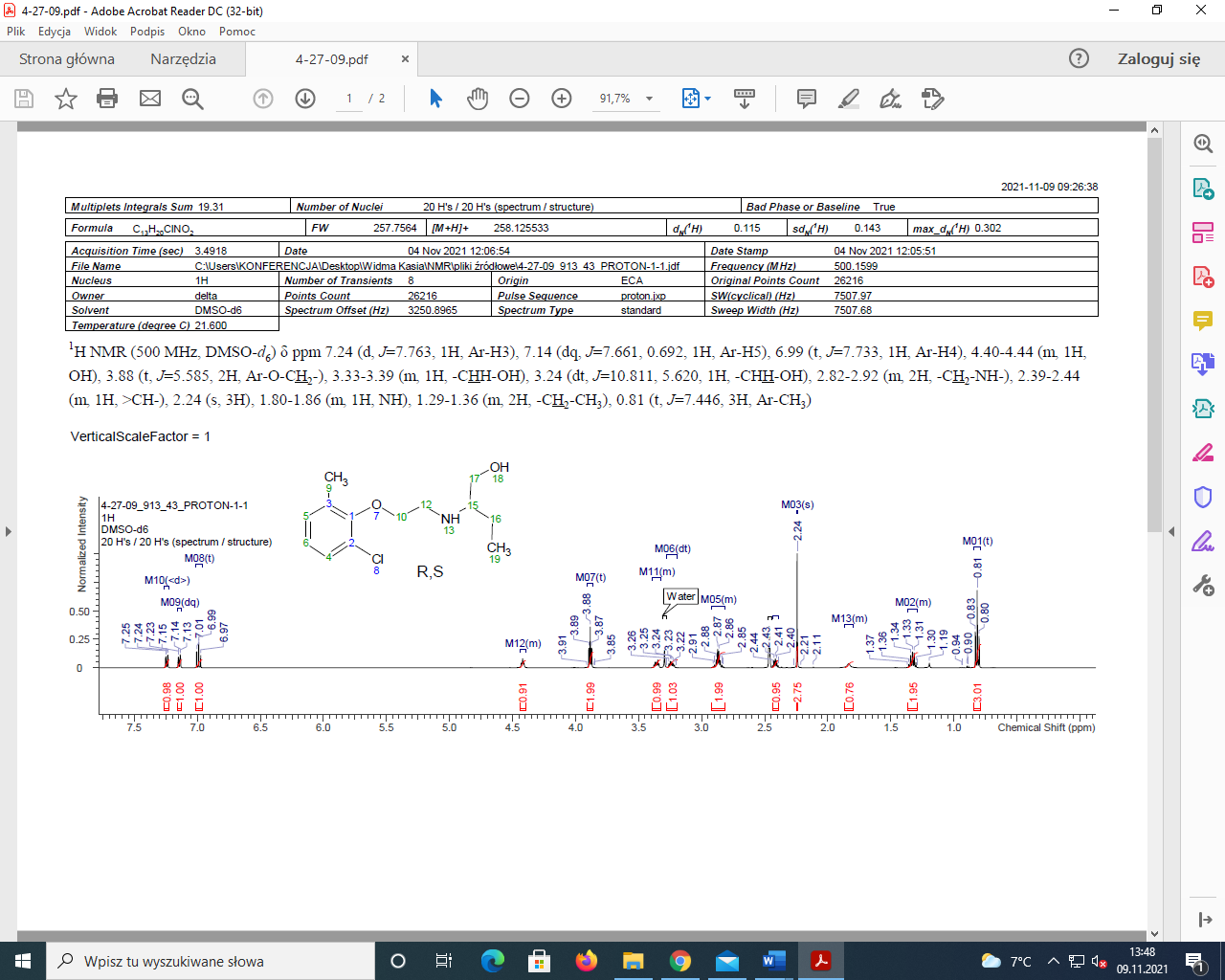


^13^CNMR


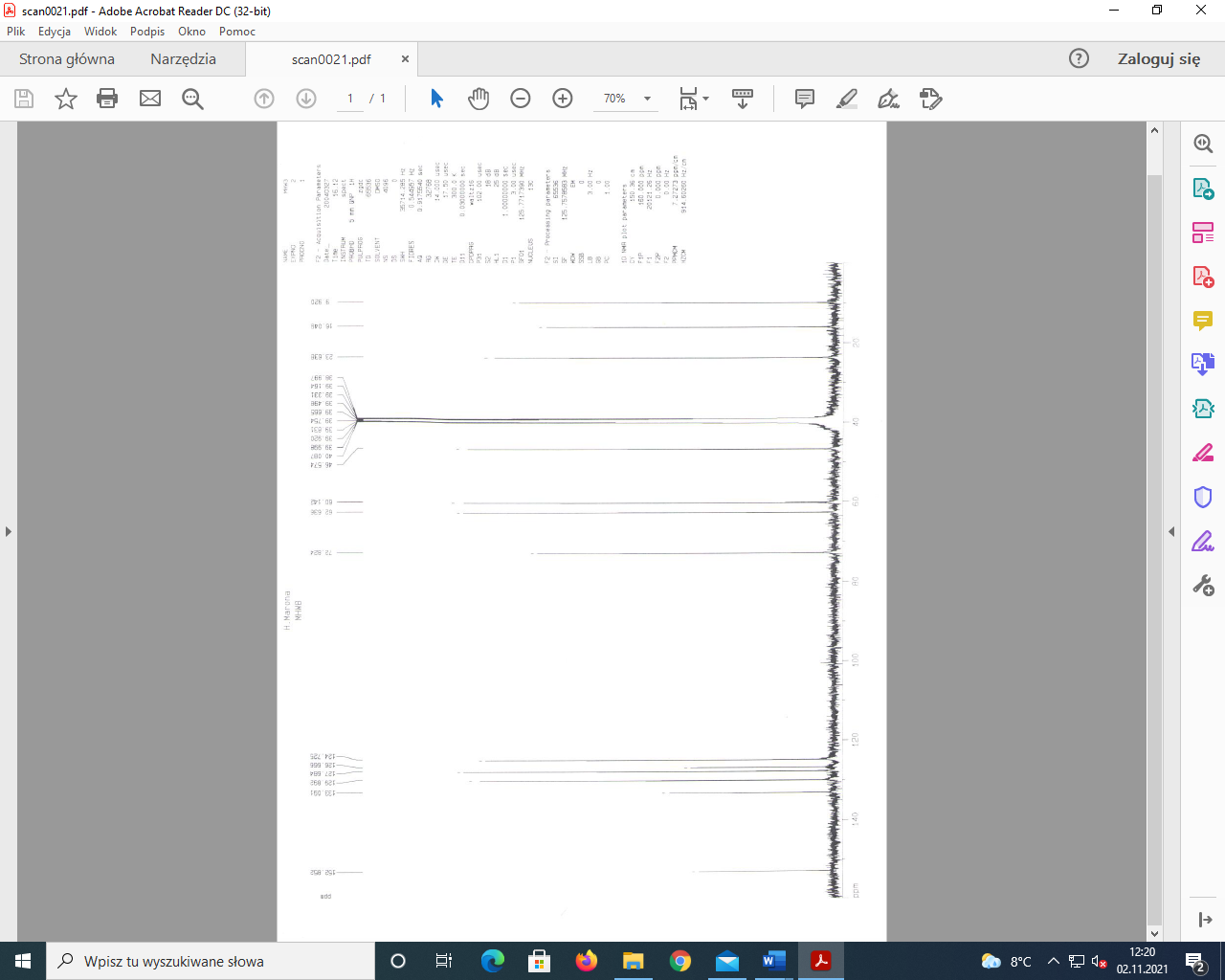


**Compound 4a**

LCMS

^1^HNMR


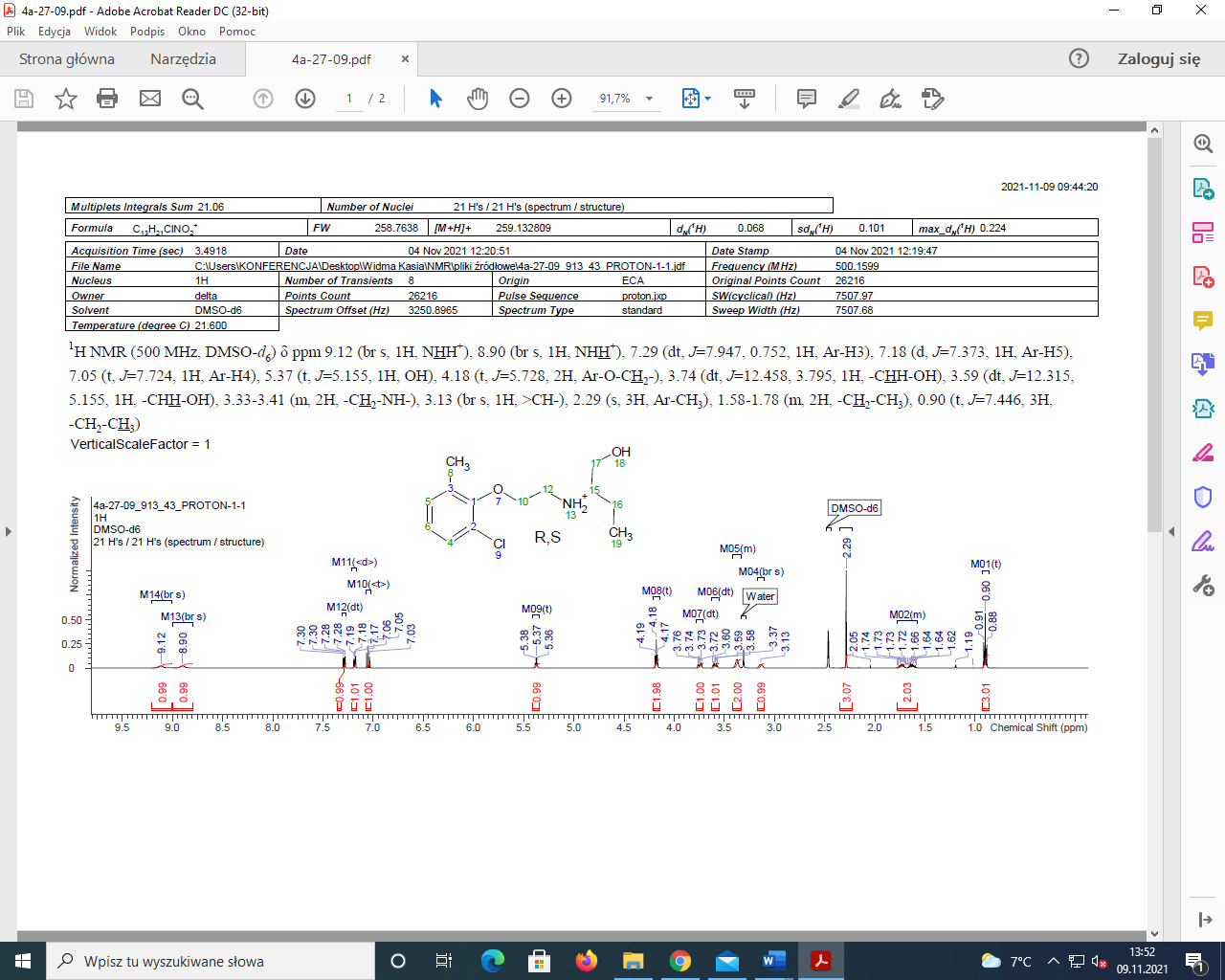


**Compound 5**

LCMS


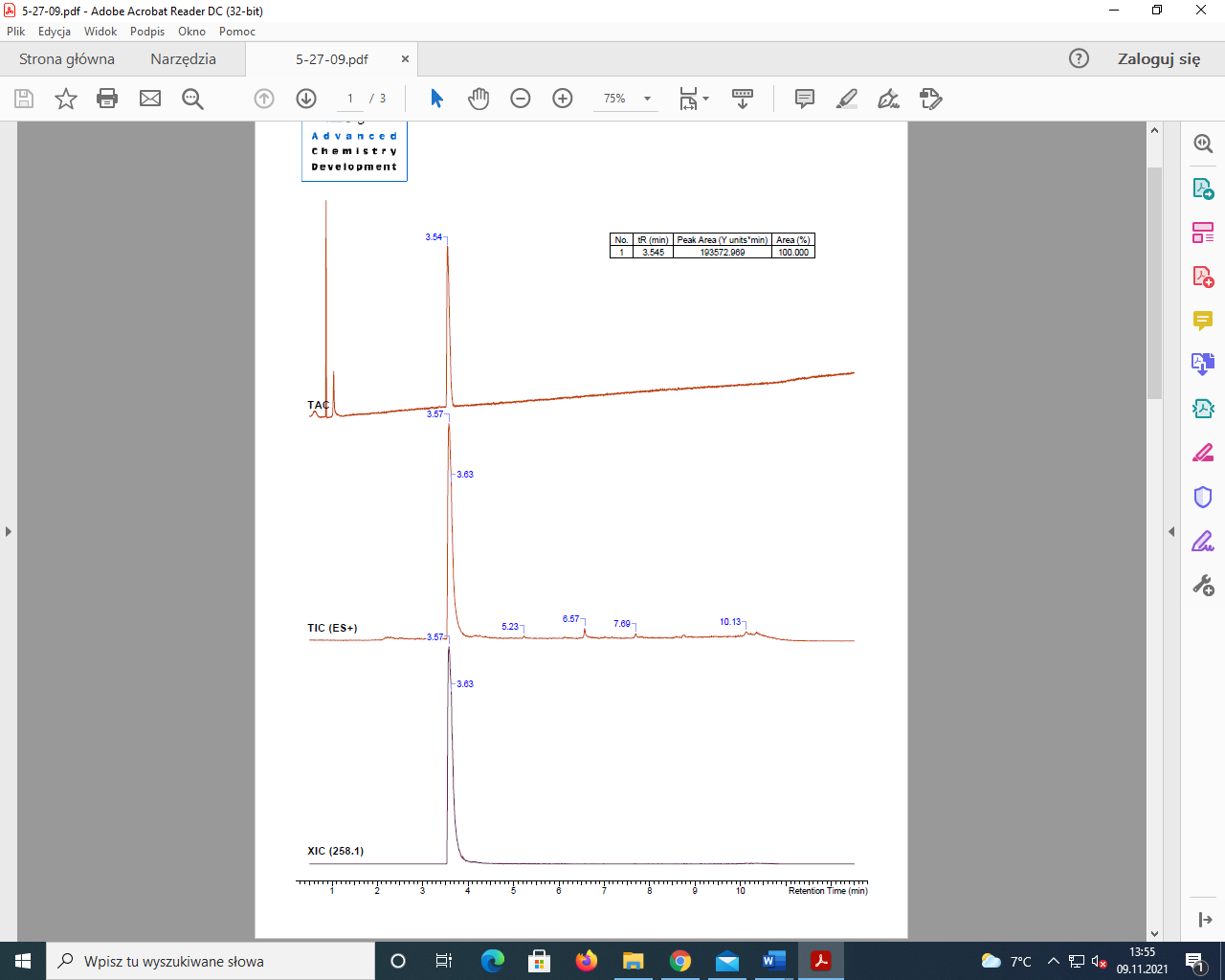


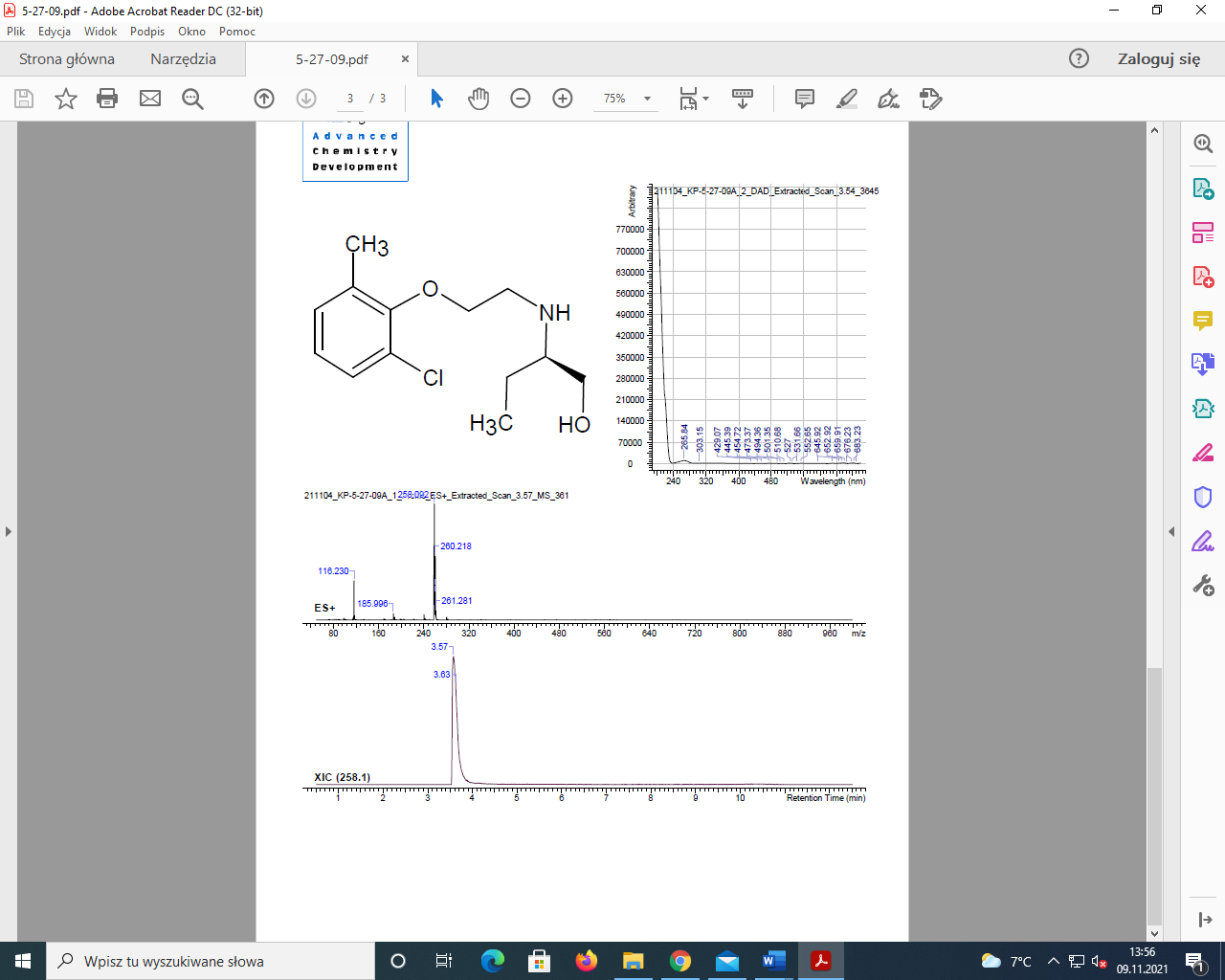


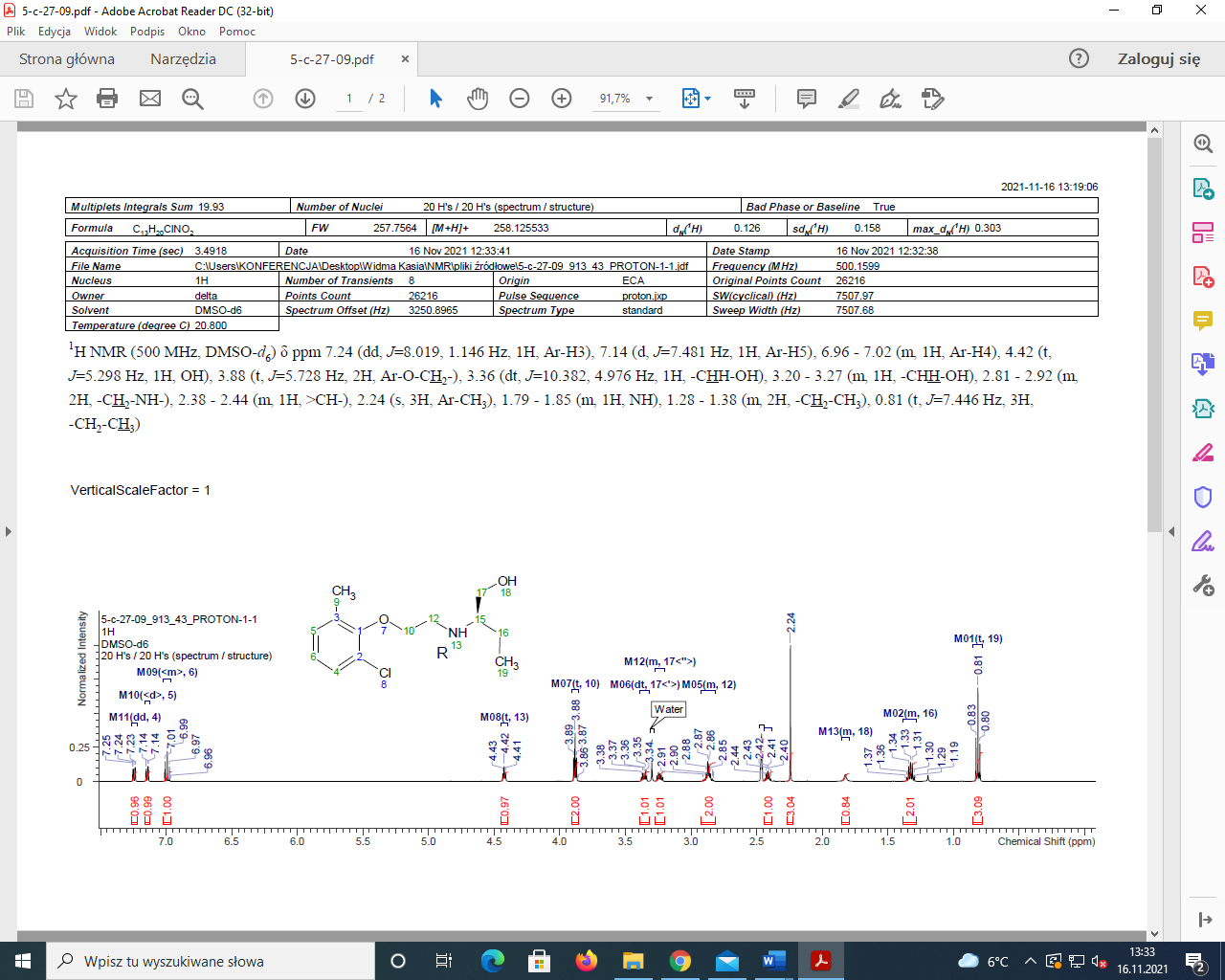


**Compound 5a**

LCMS


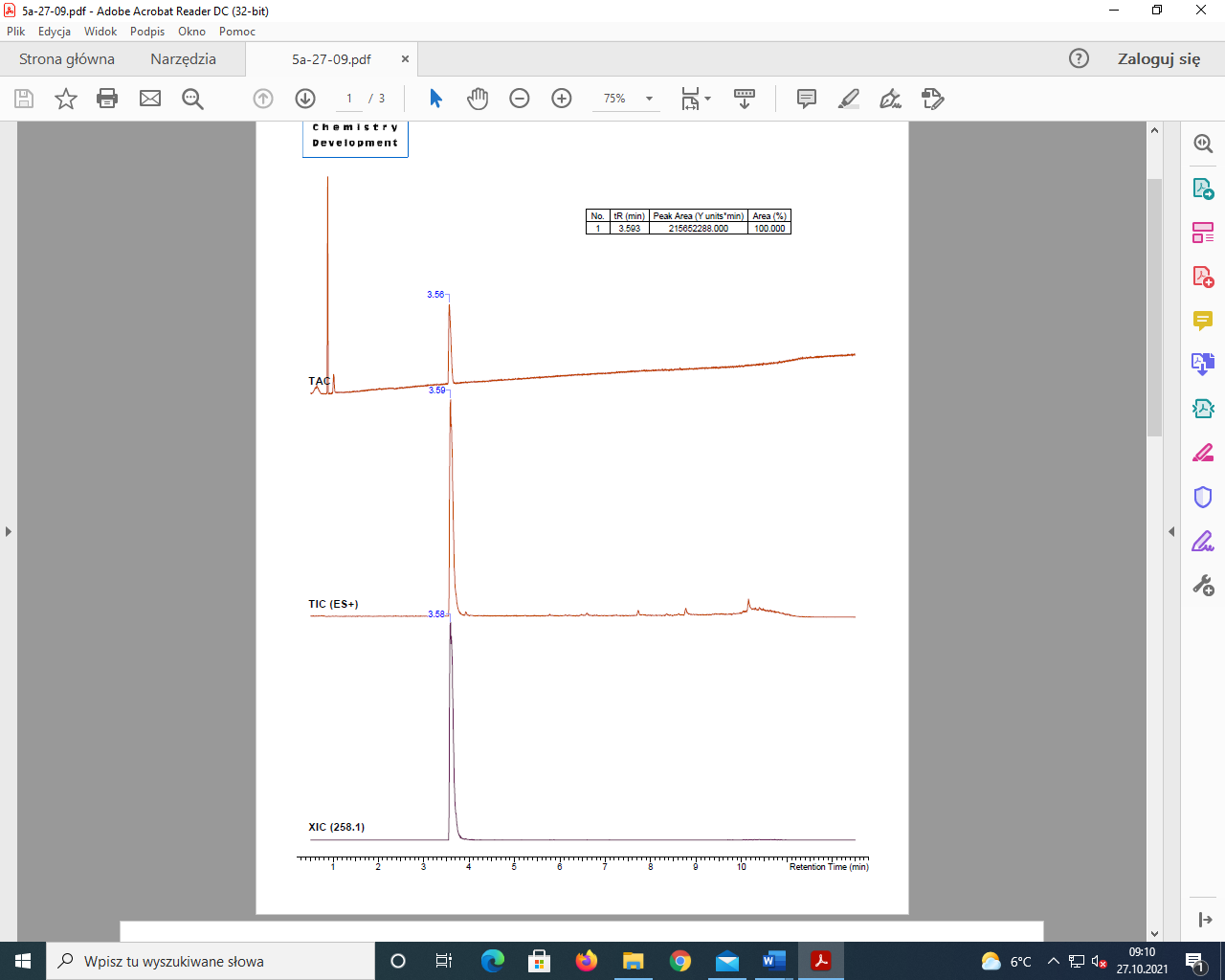


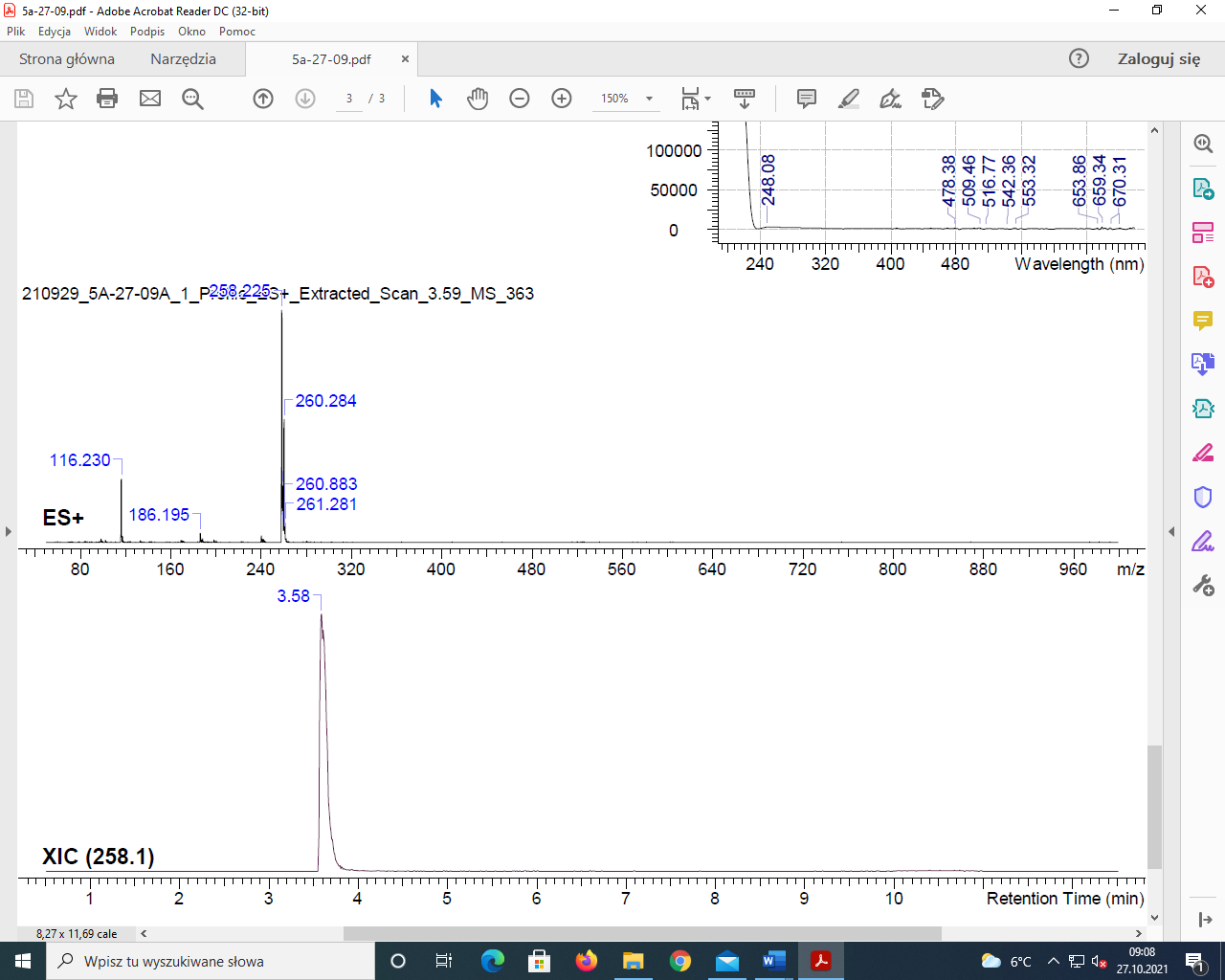


^1^HNMR


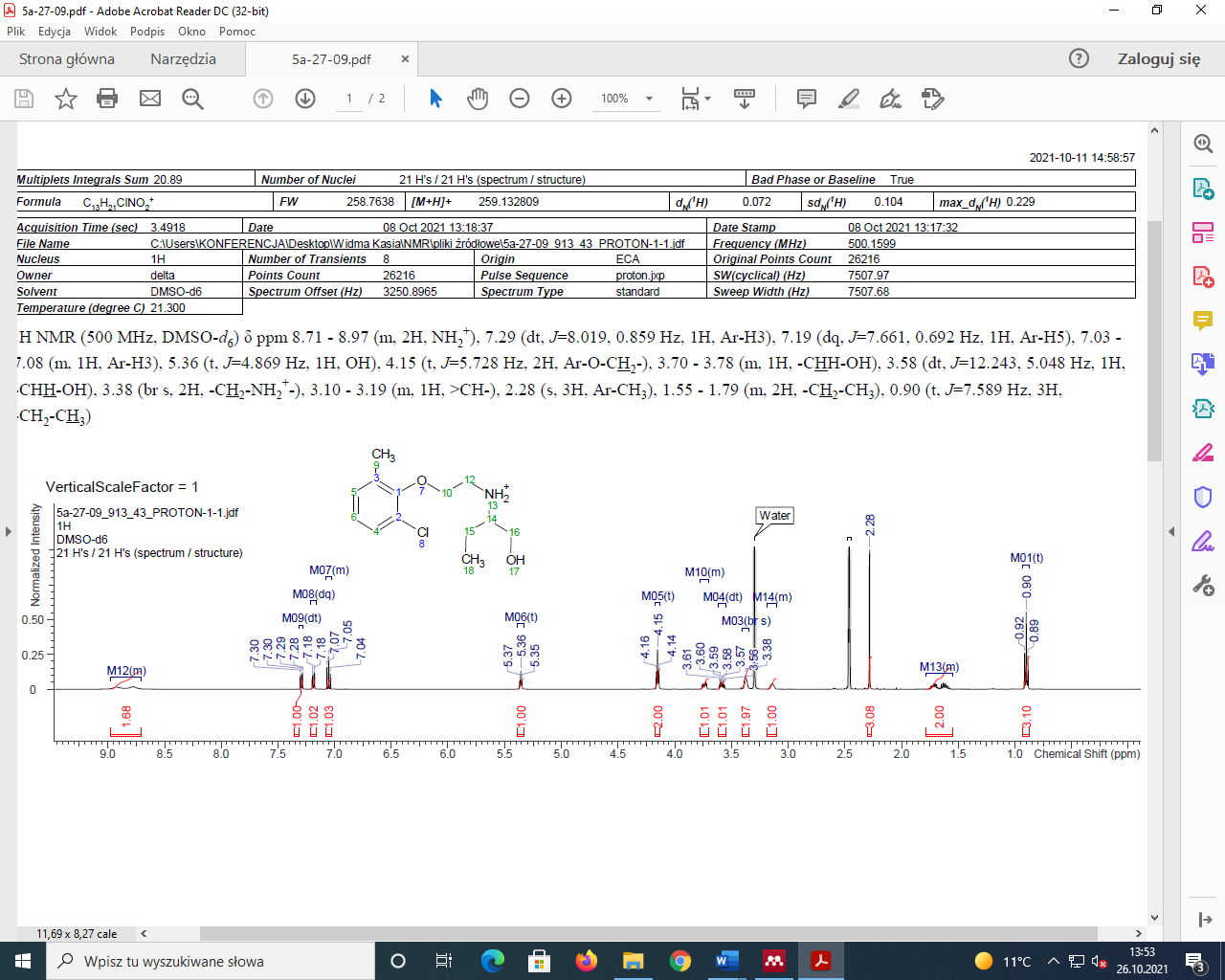


**Compound 6**

LCMS


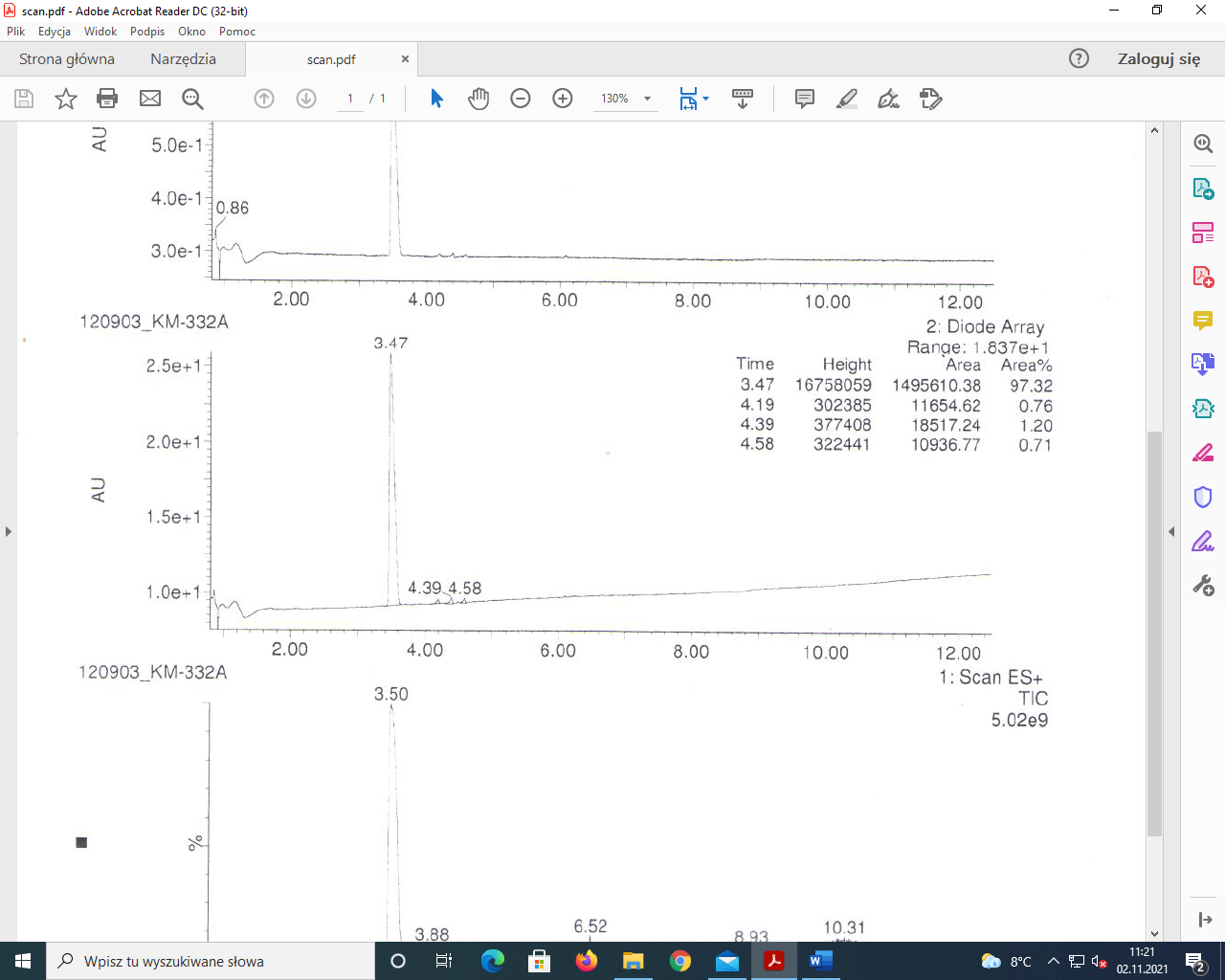


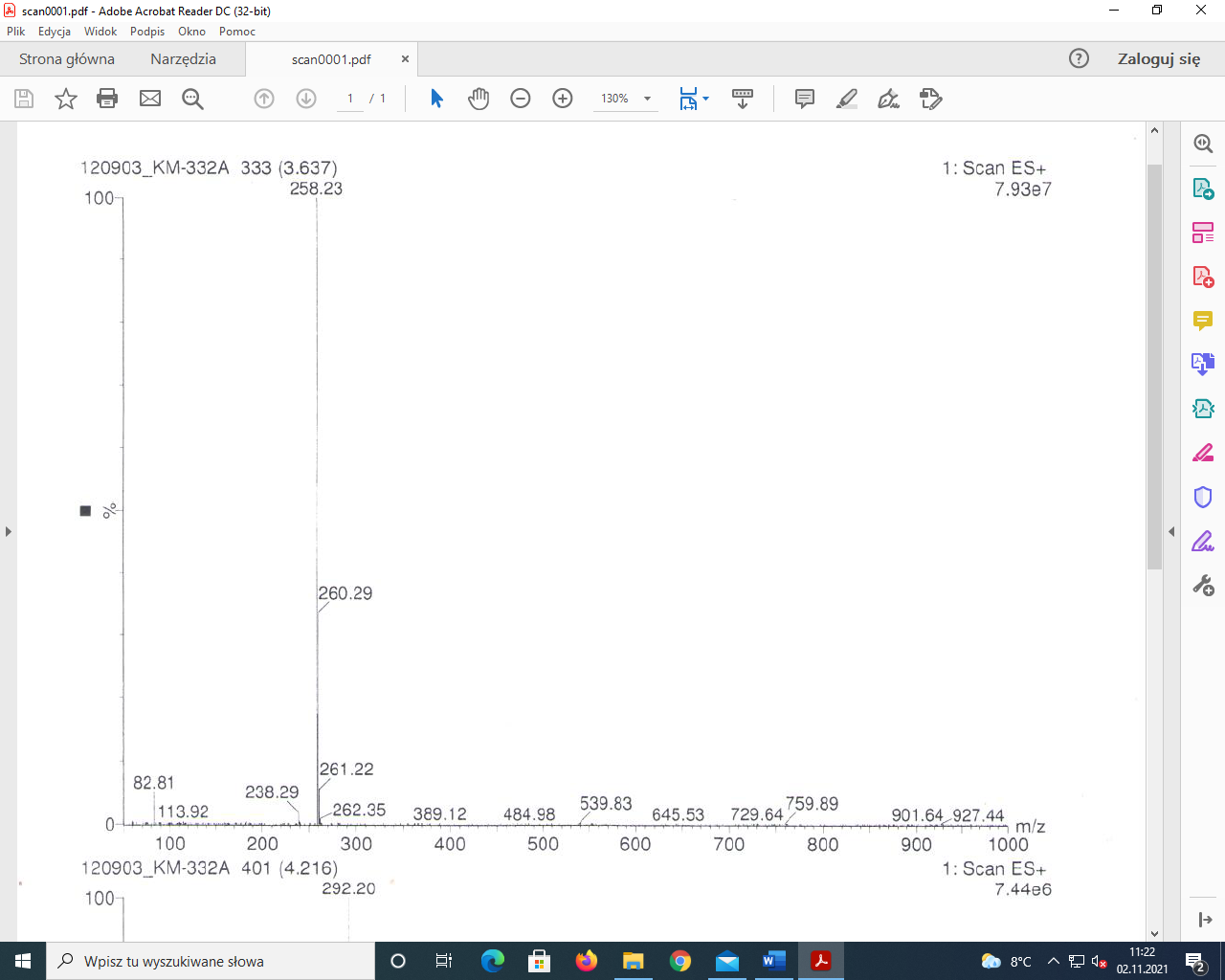


^1^HNMR


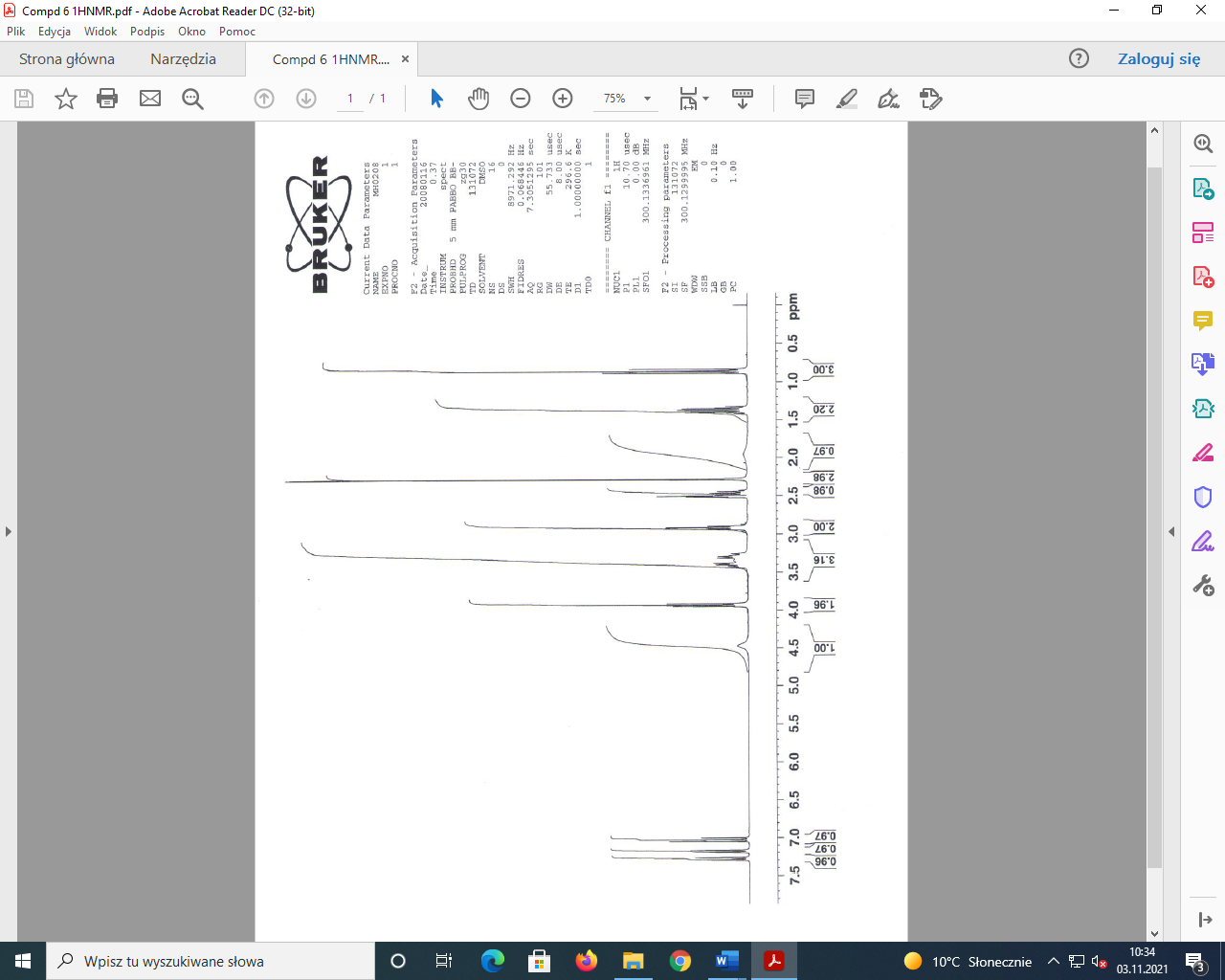


**Compound 6a**

LCMS


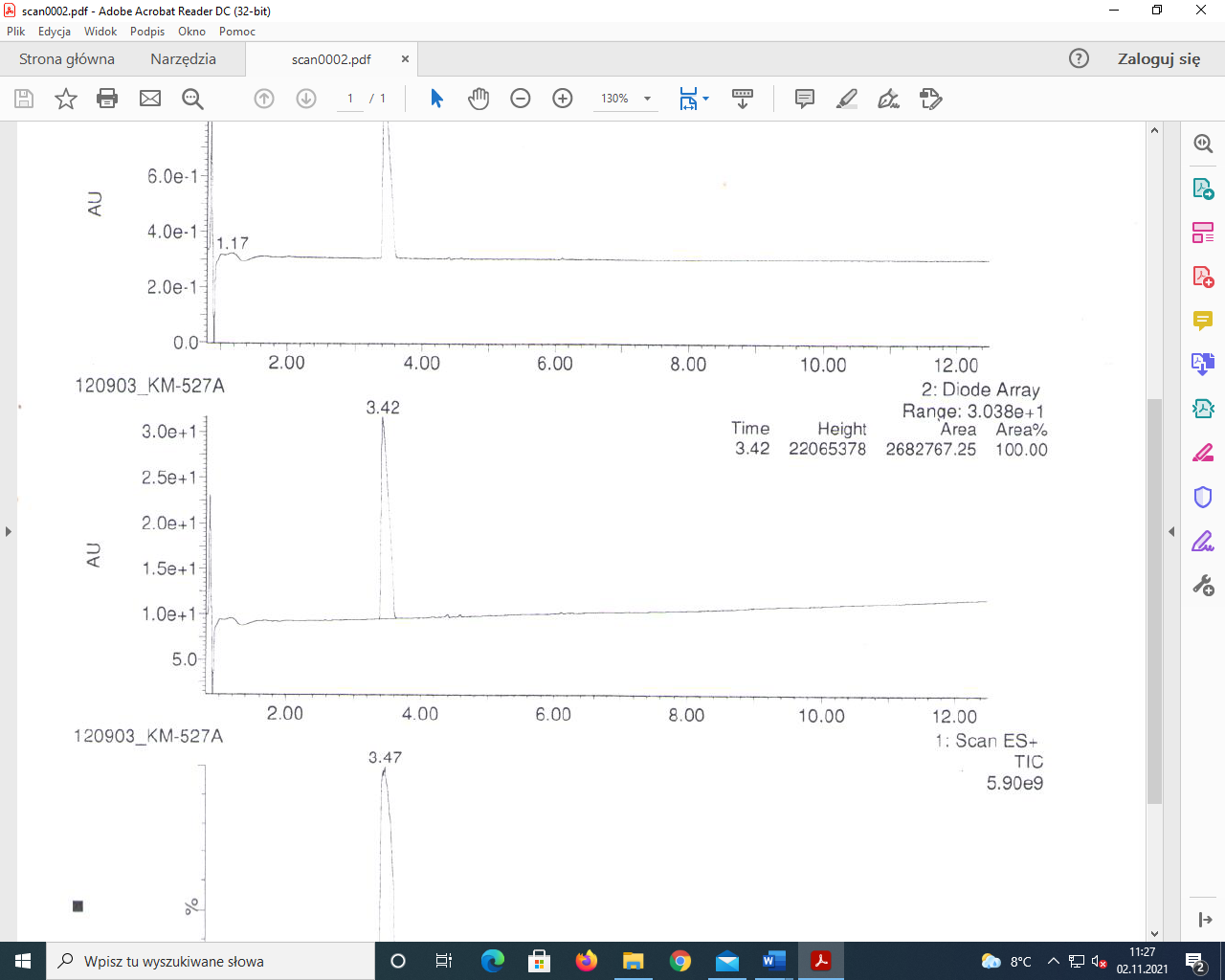


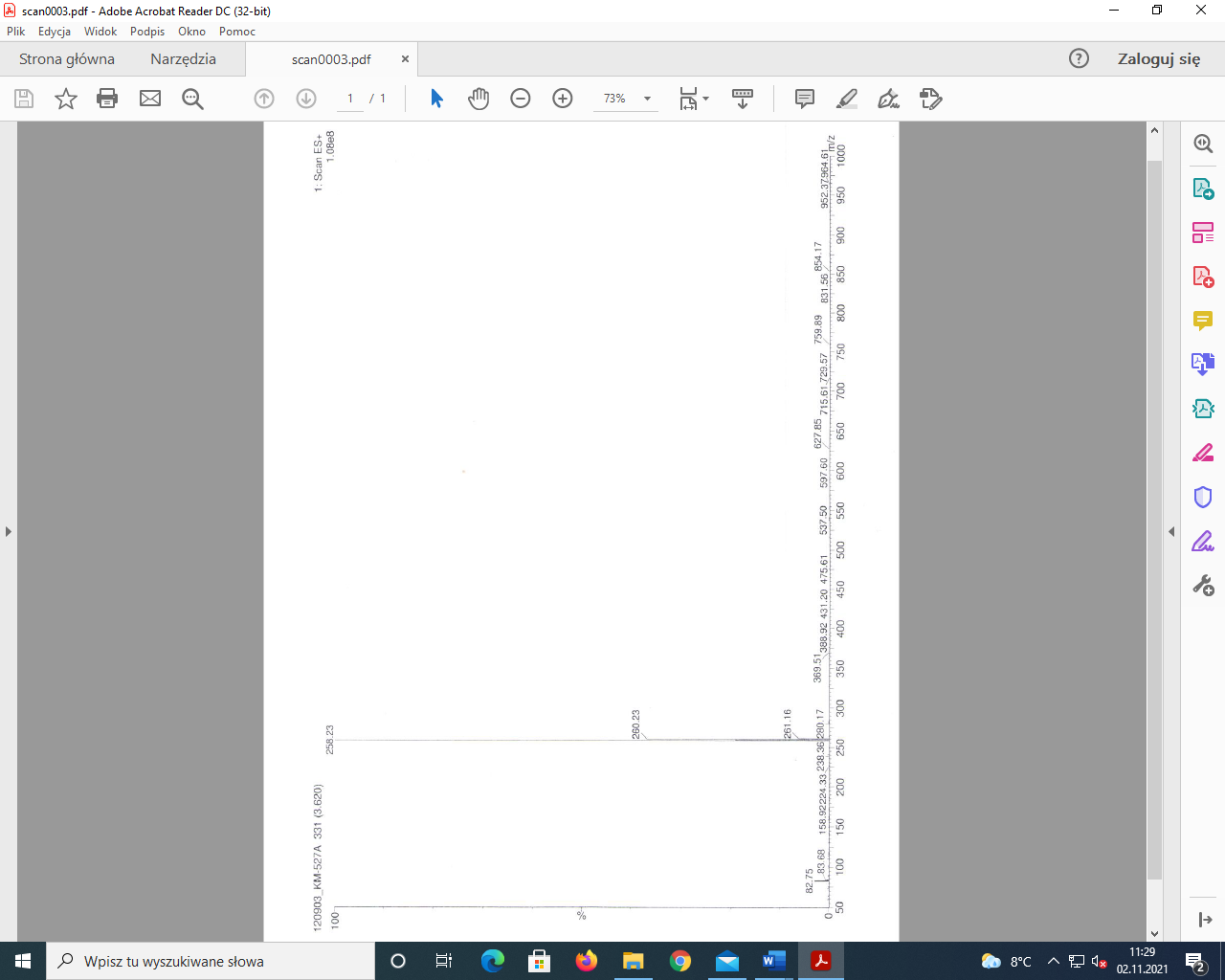


^1^HNMR


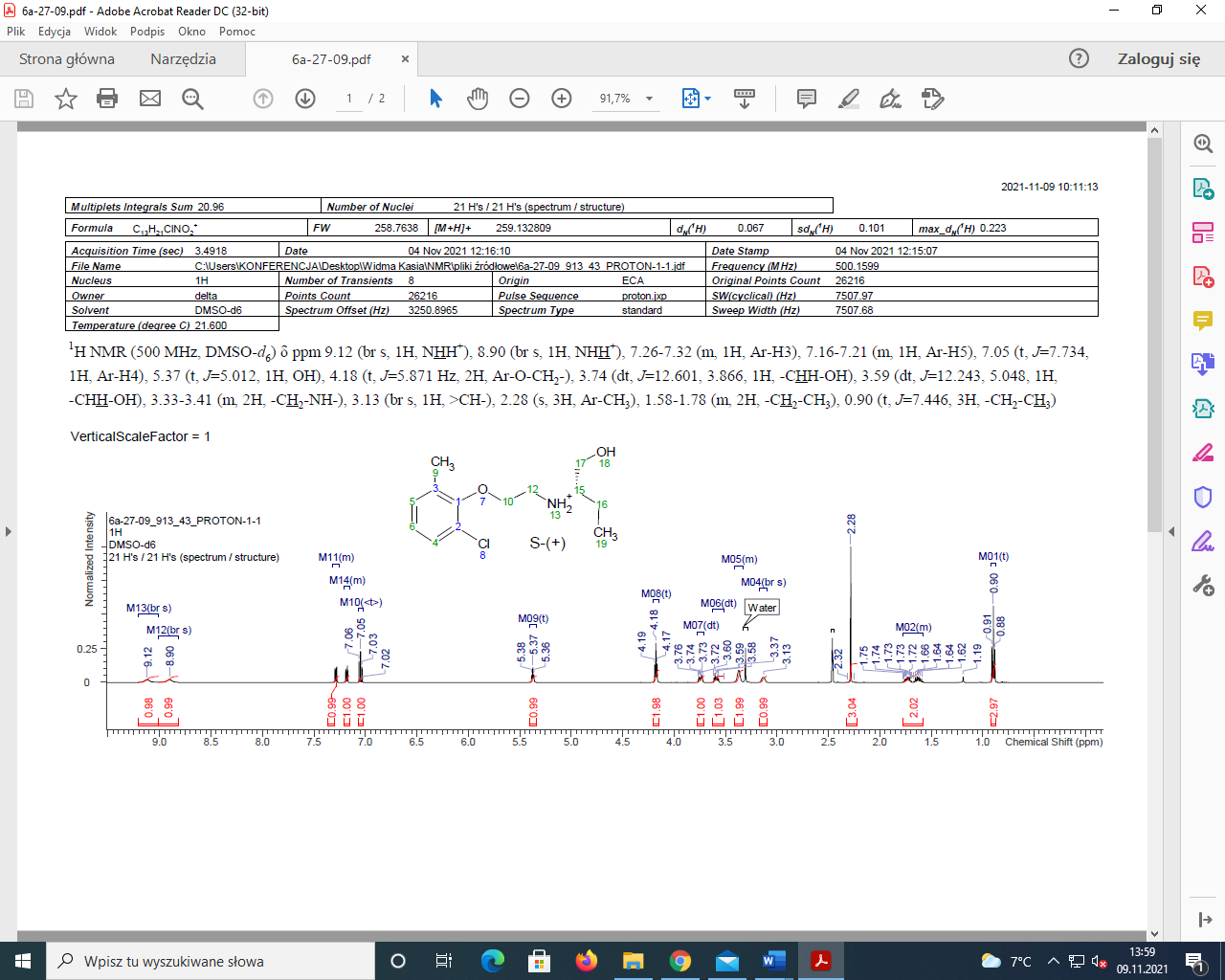


**Compound 7**

LCMS


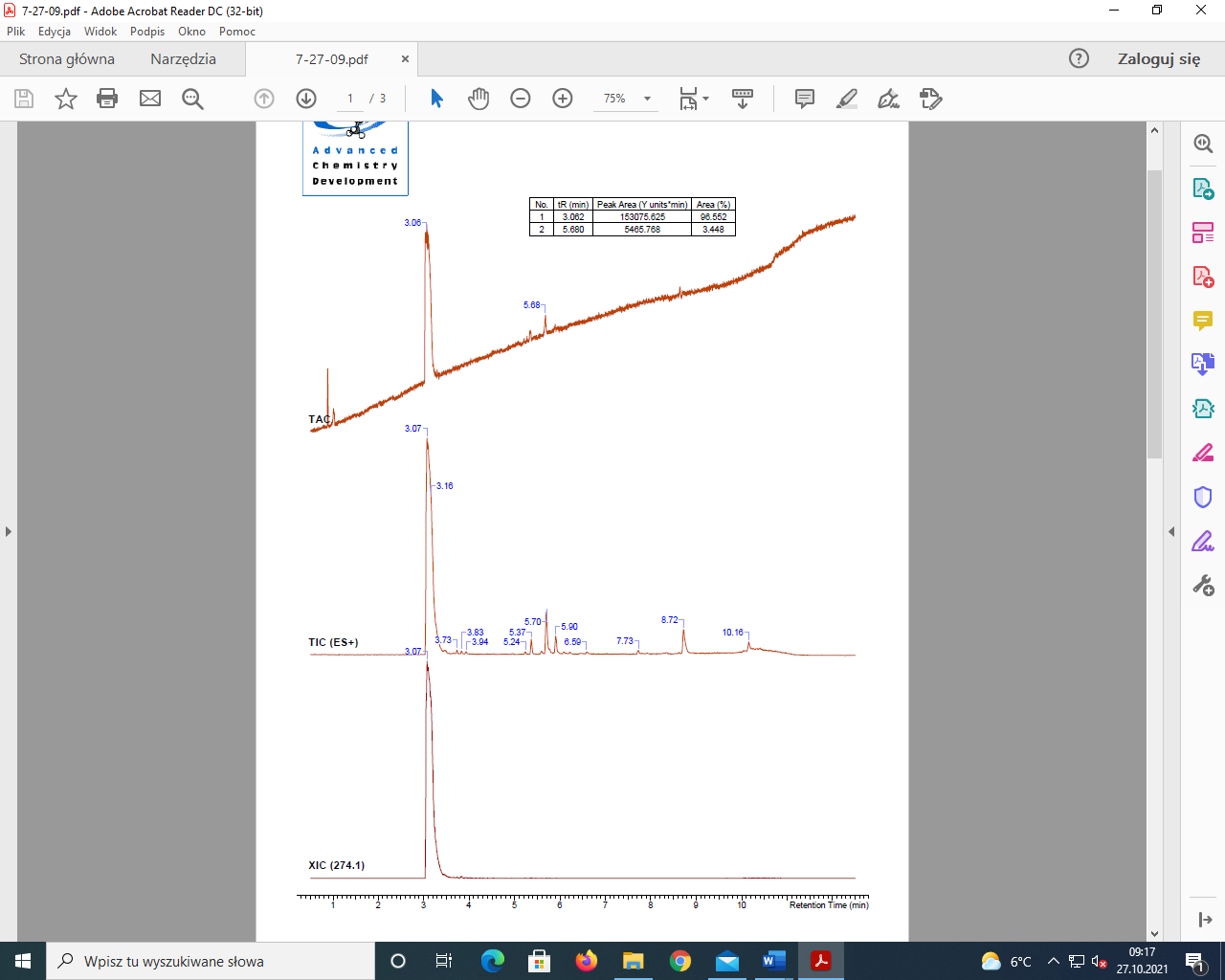


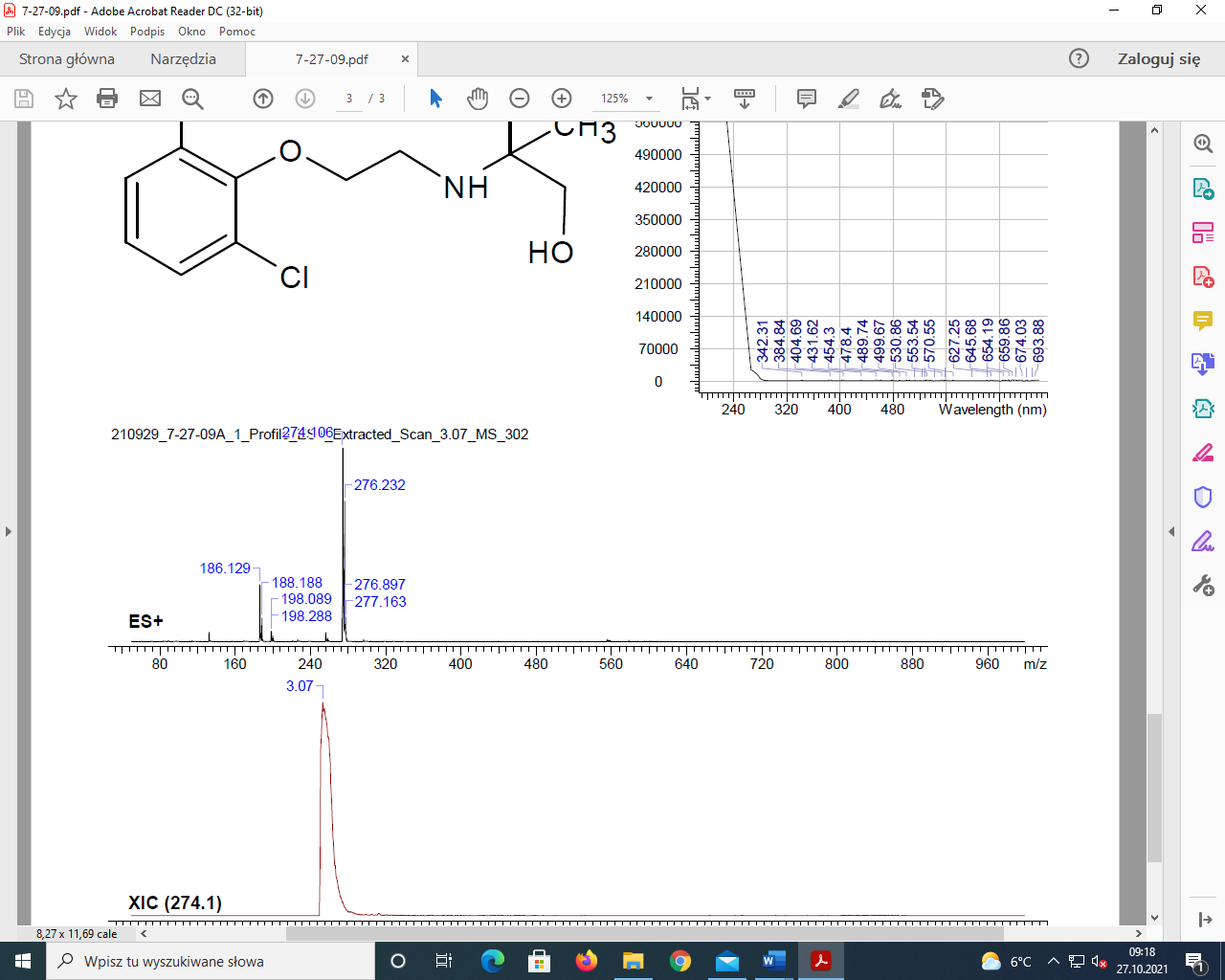


^1^HNMR


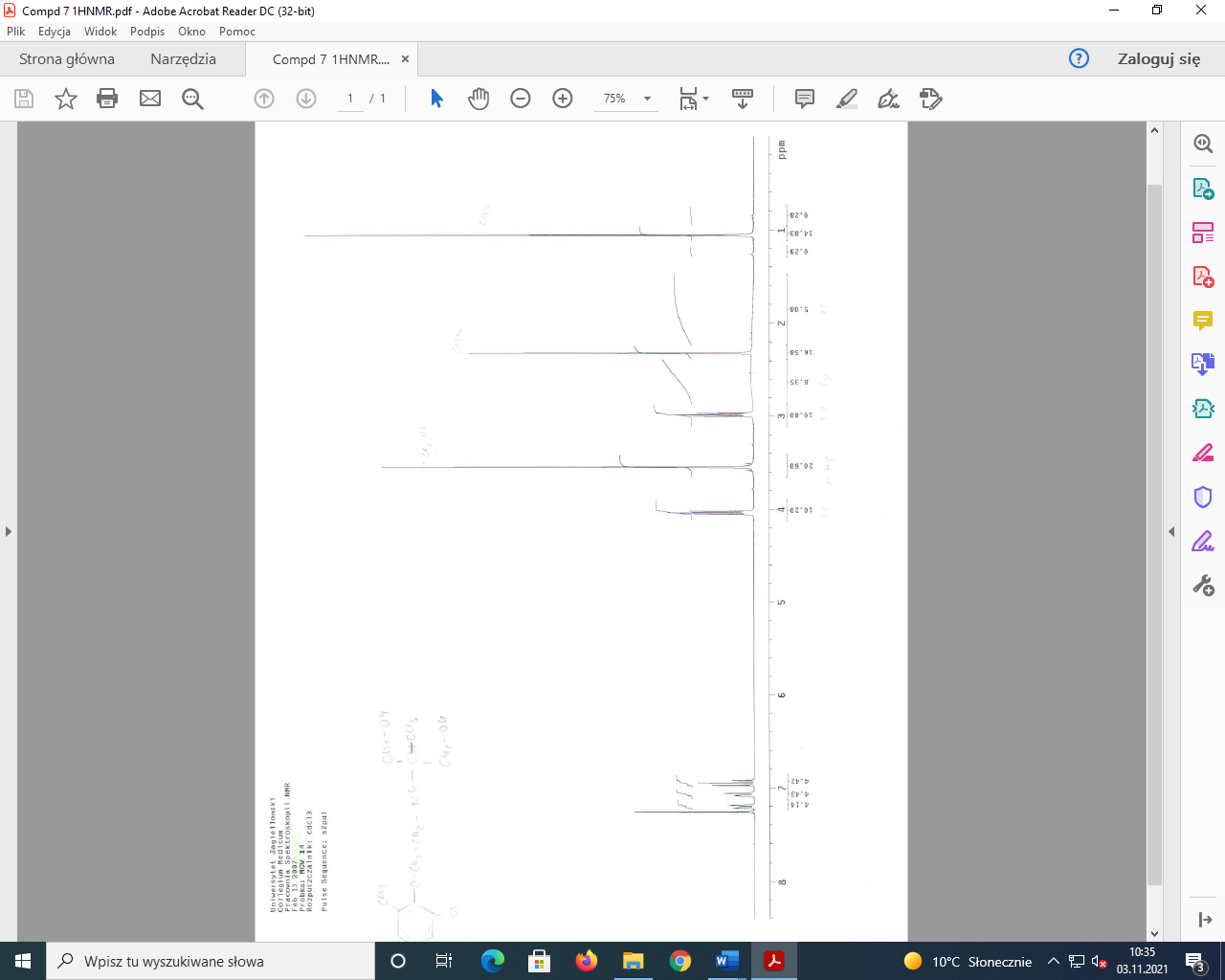


**Compound 8**

LCMS


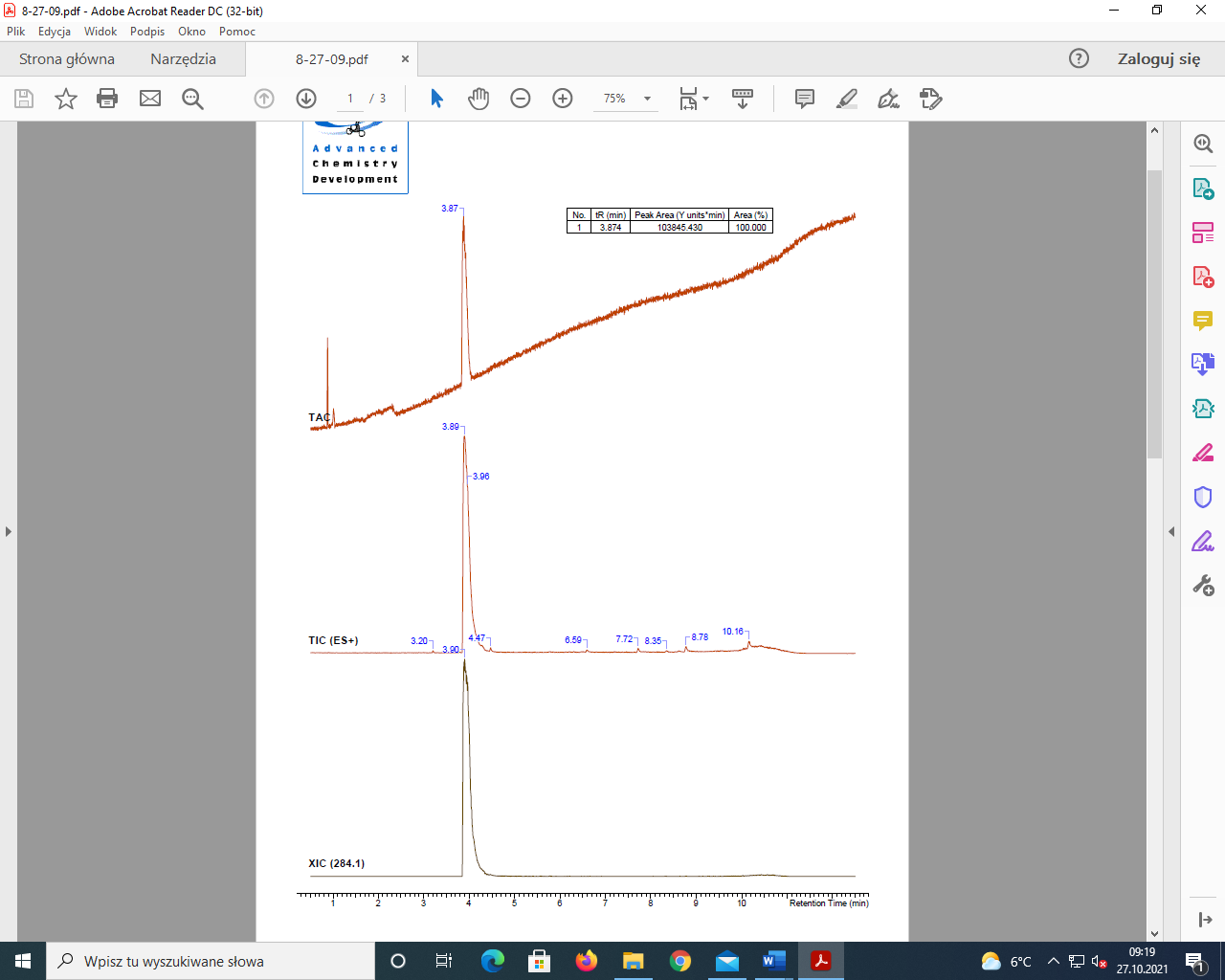


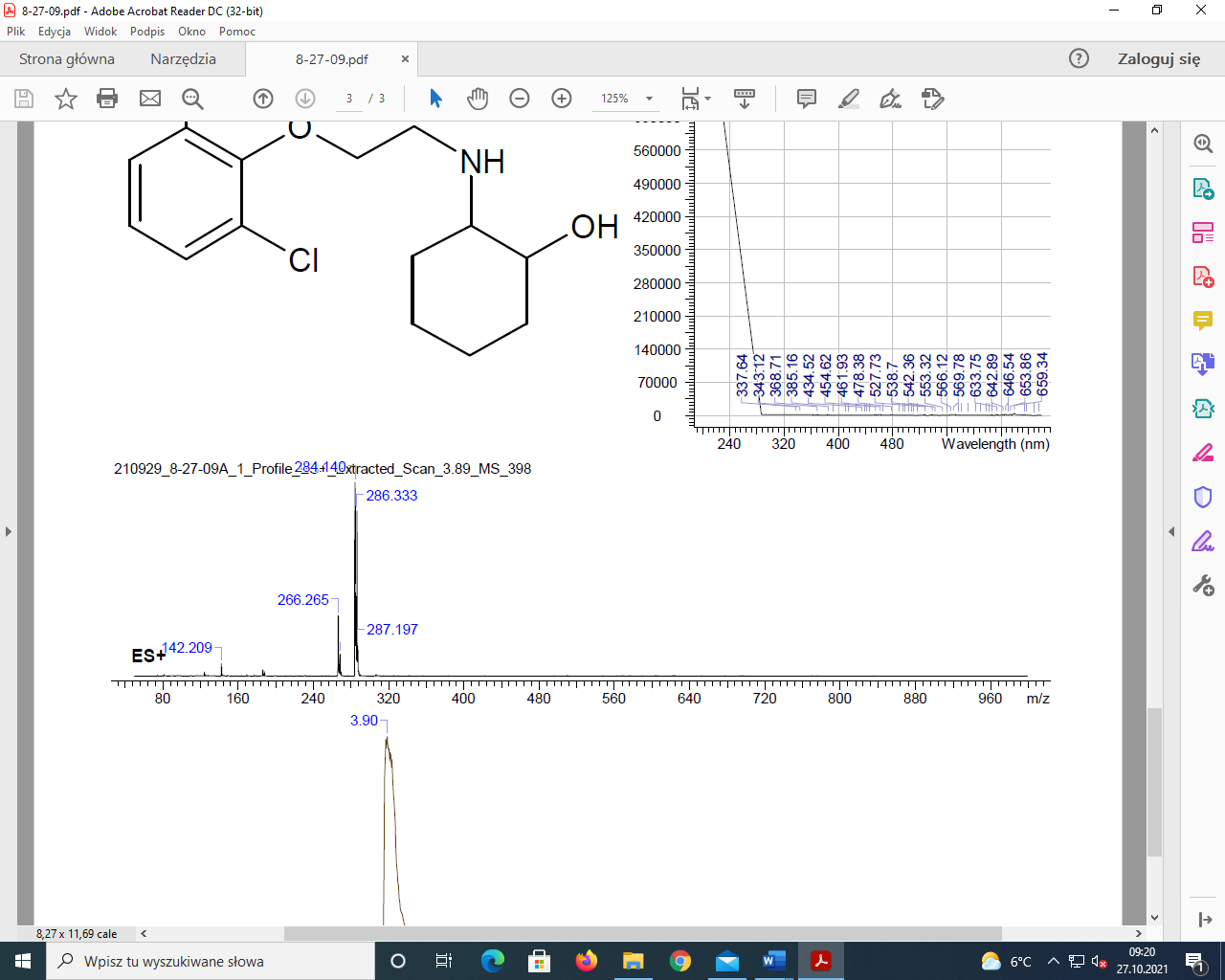


^1^HNMR


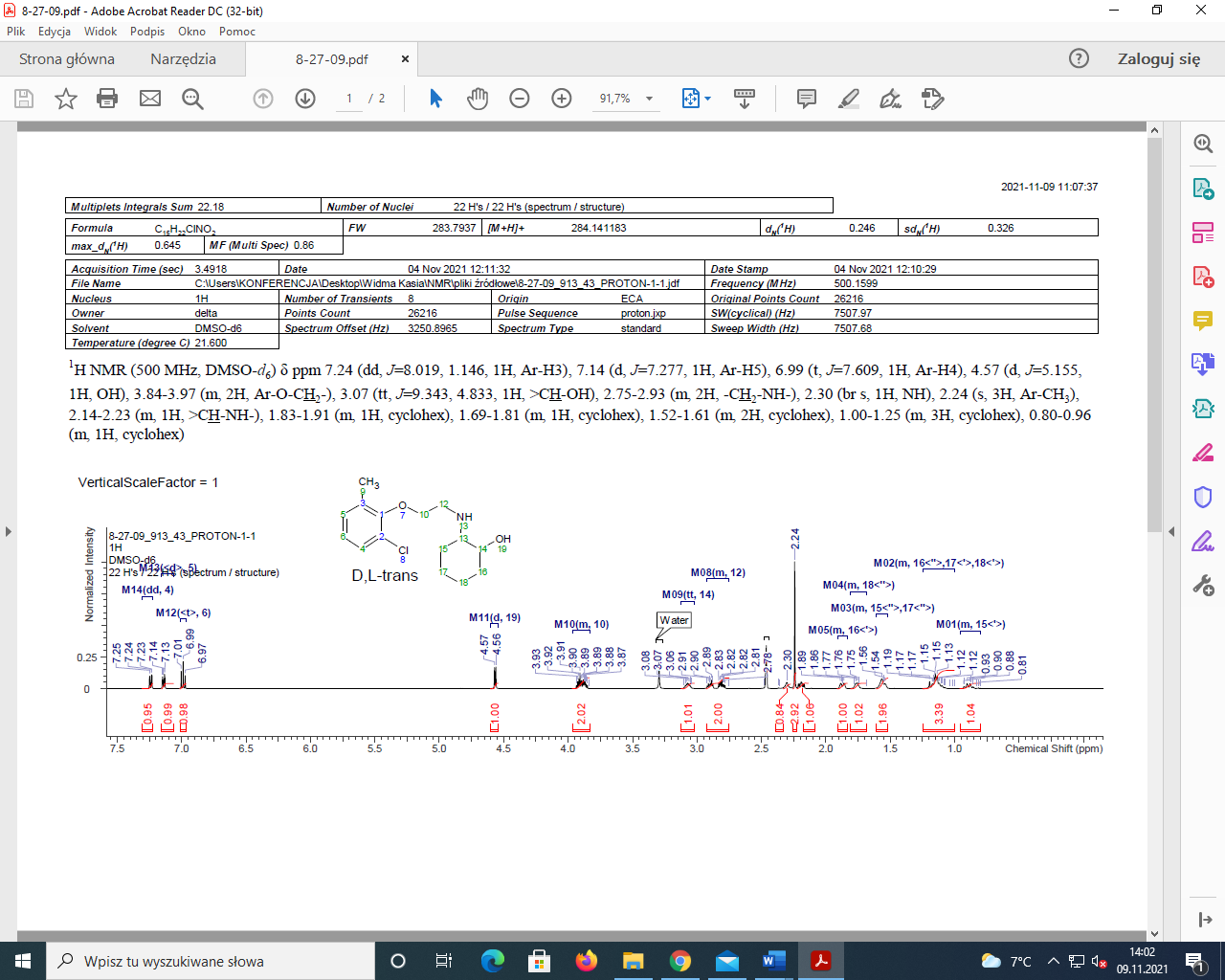


**Compound 9**

LCMS


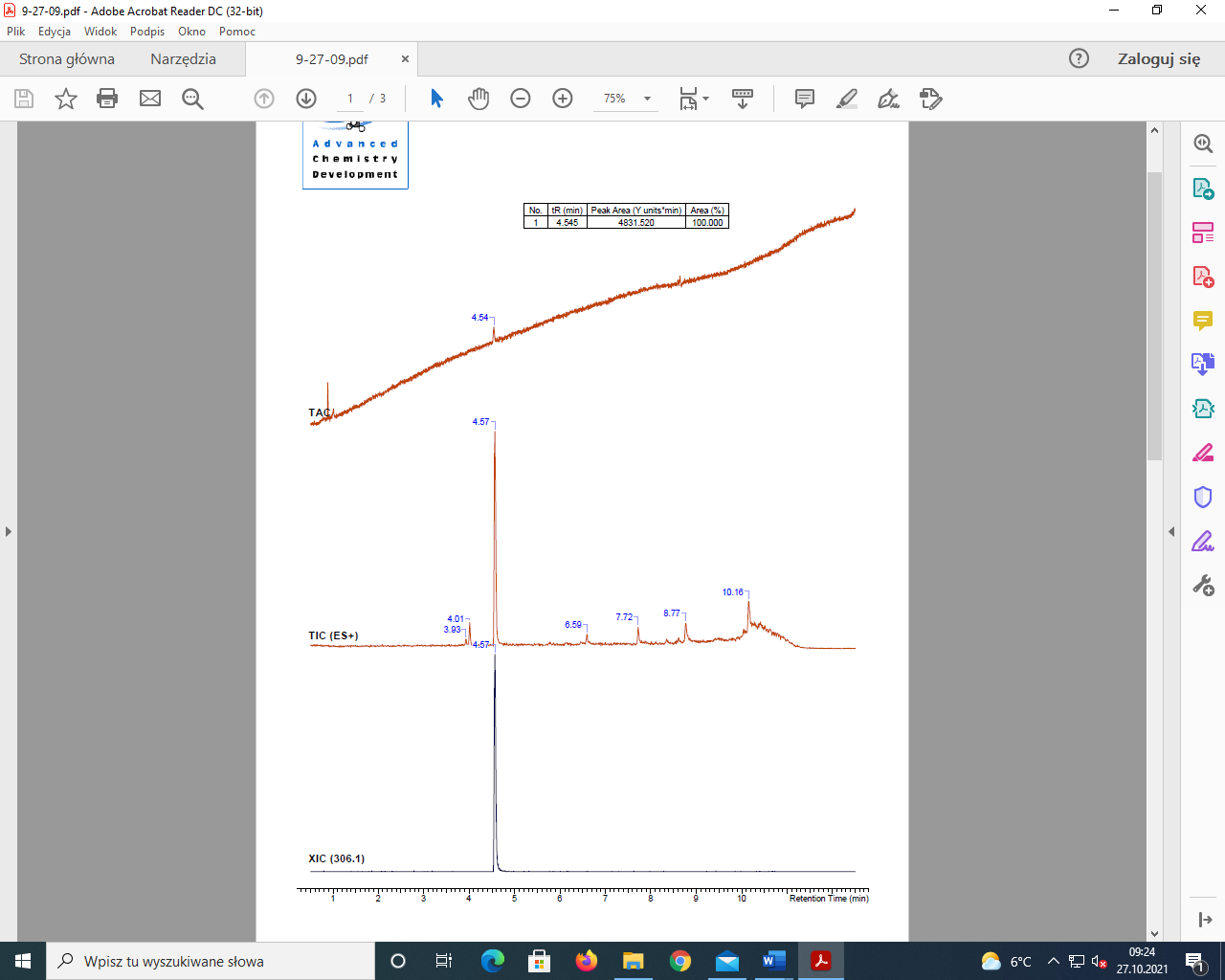


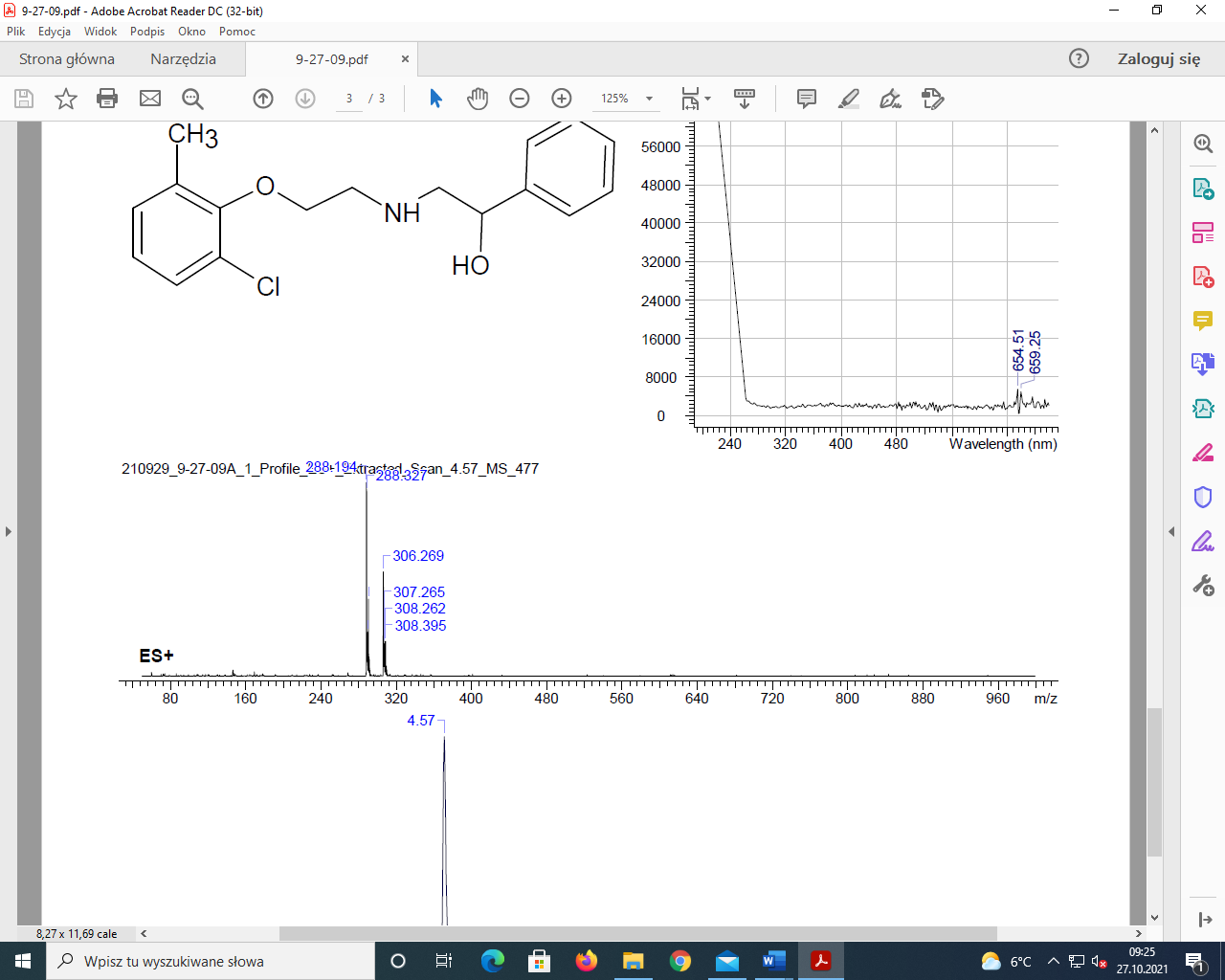


^1^HNMR


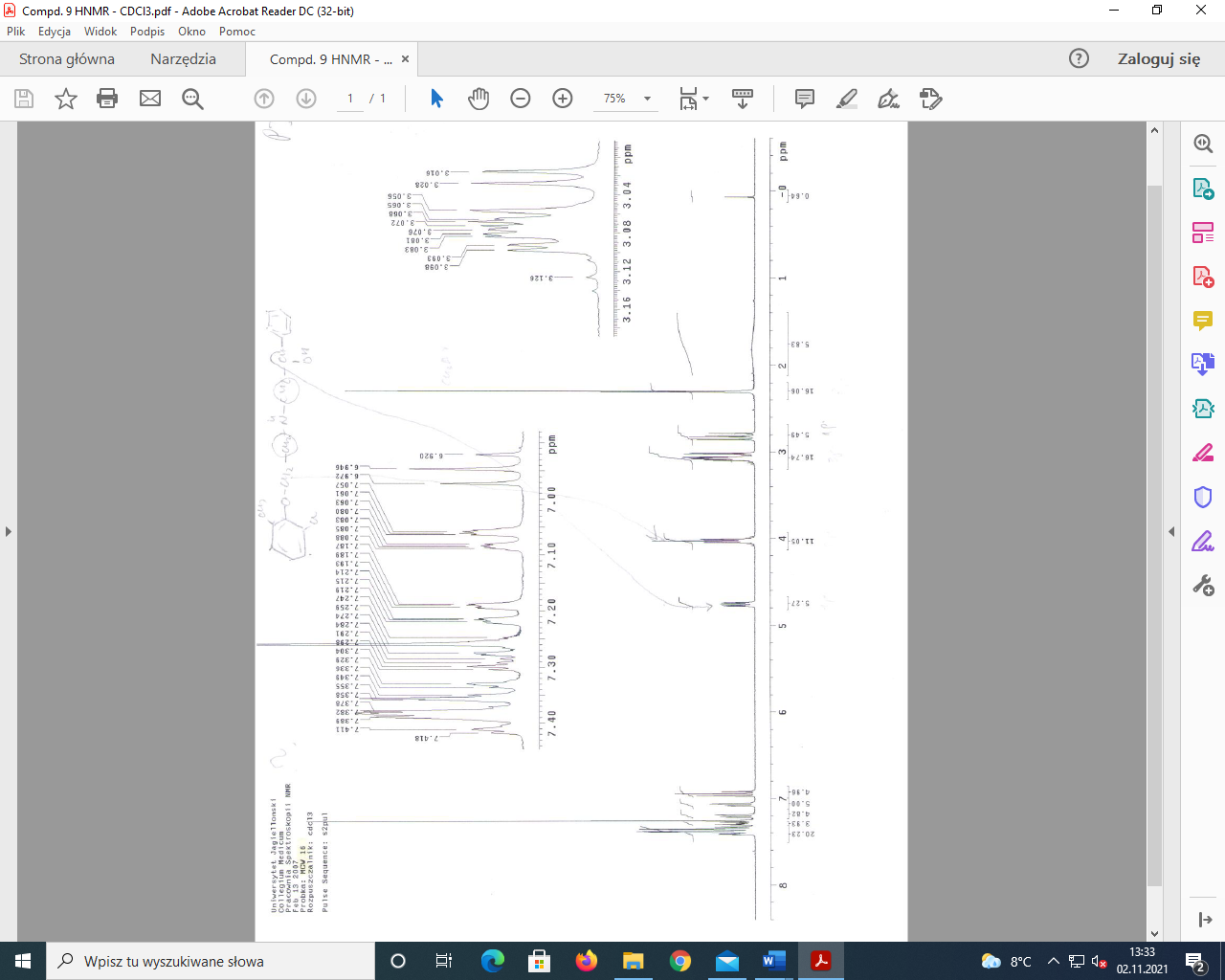


**Compound 10**

^1^HNMR


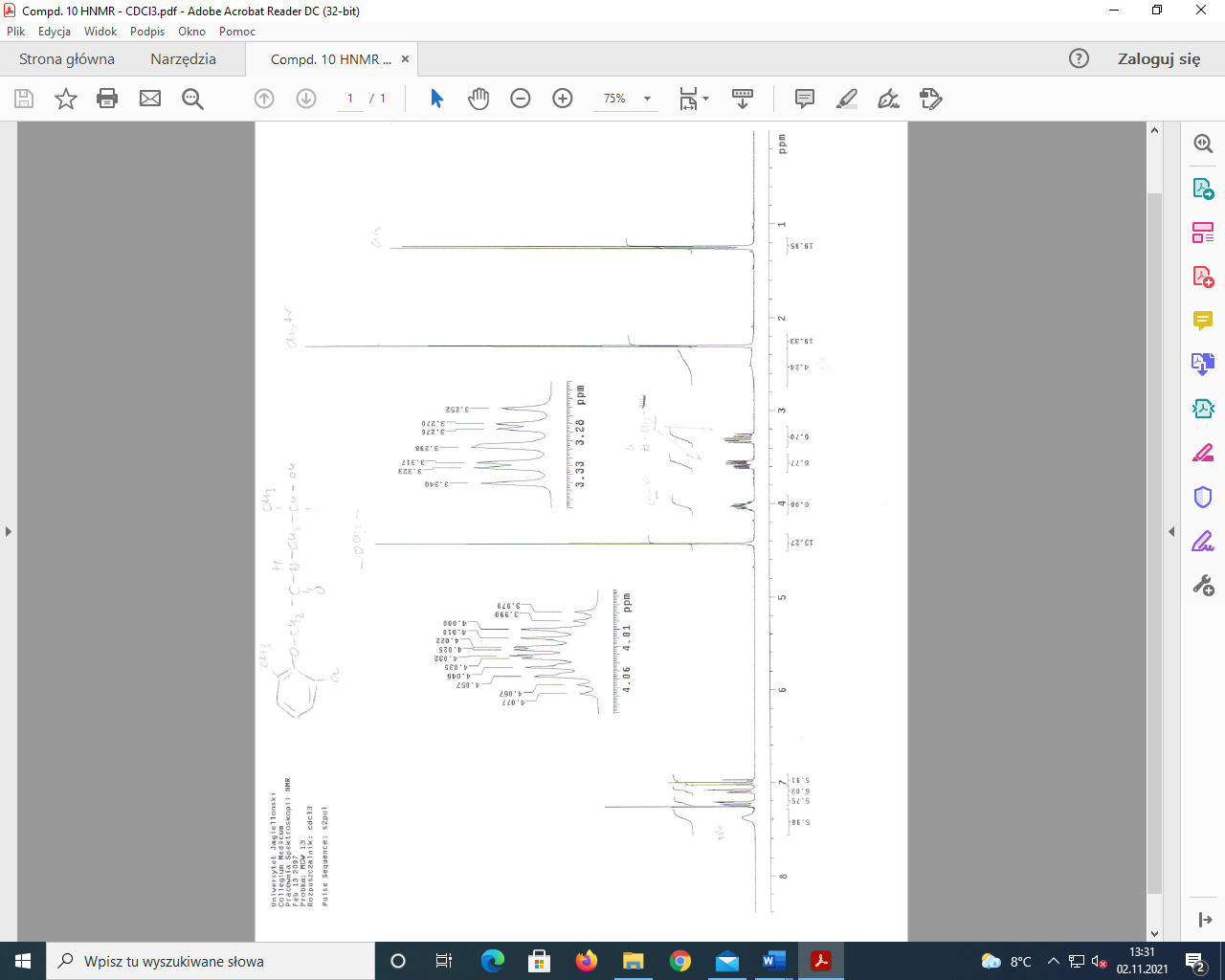


**Compound 11**

LCMS


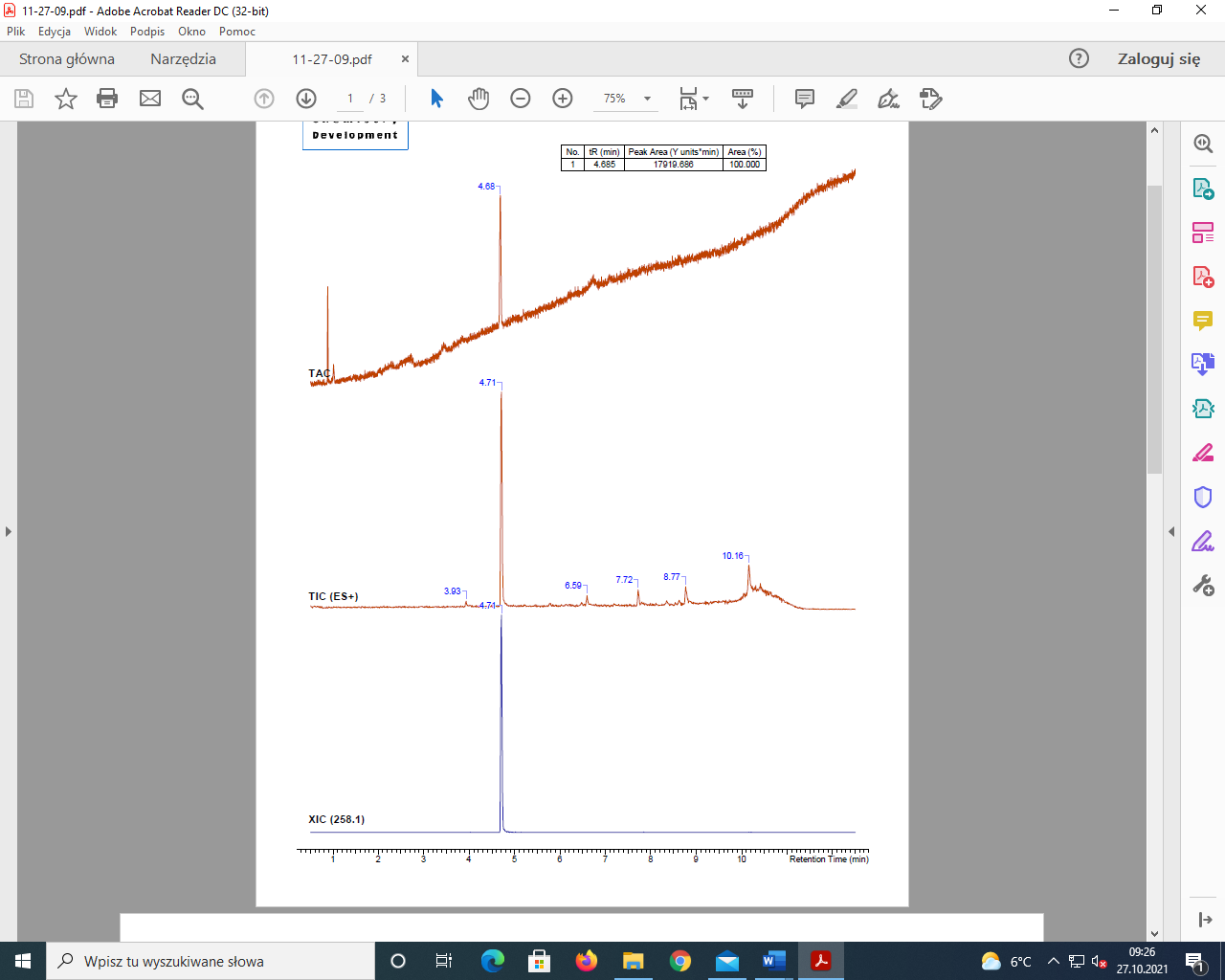


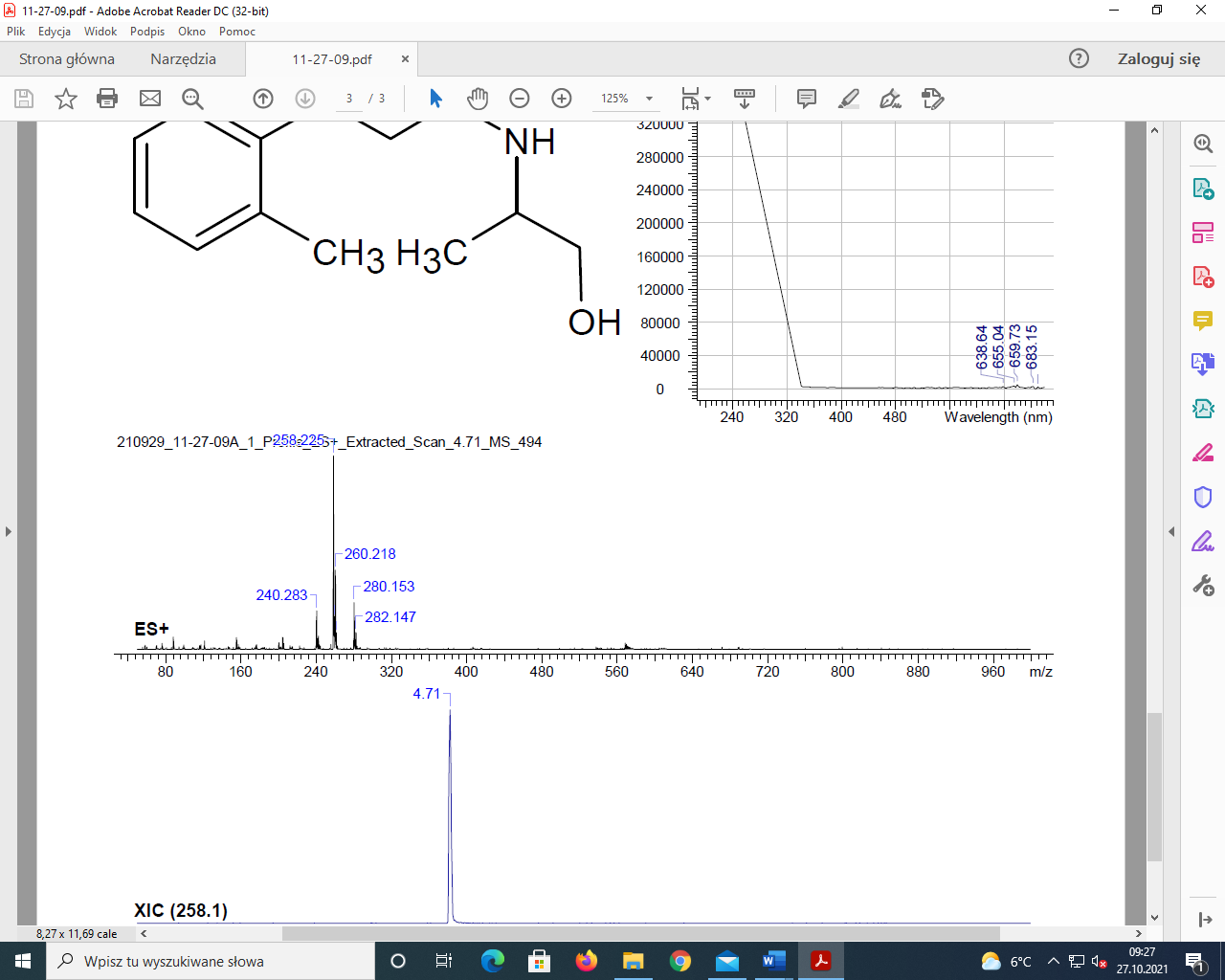


^1^HNMR


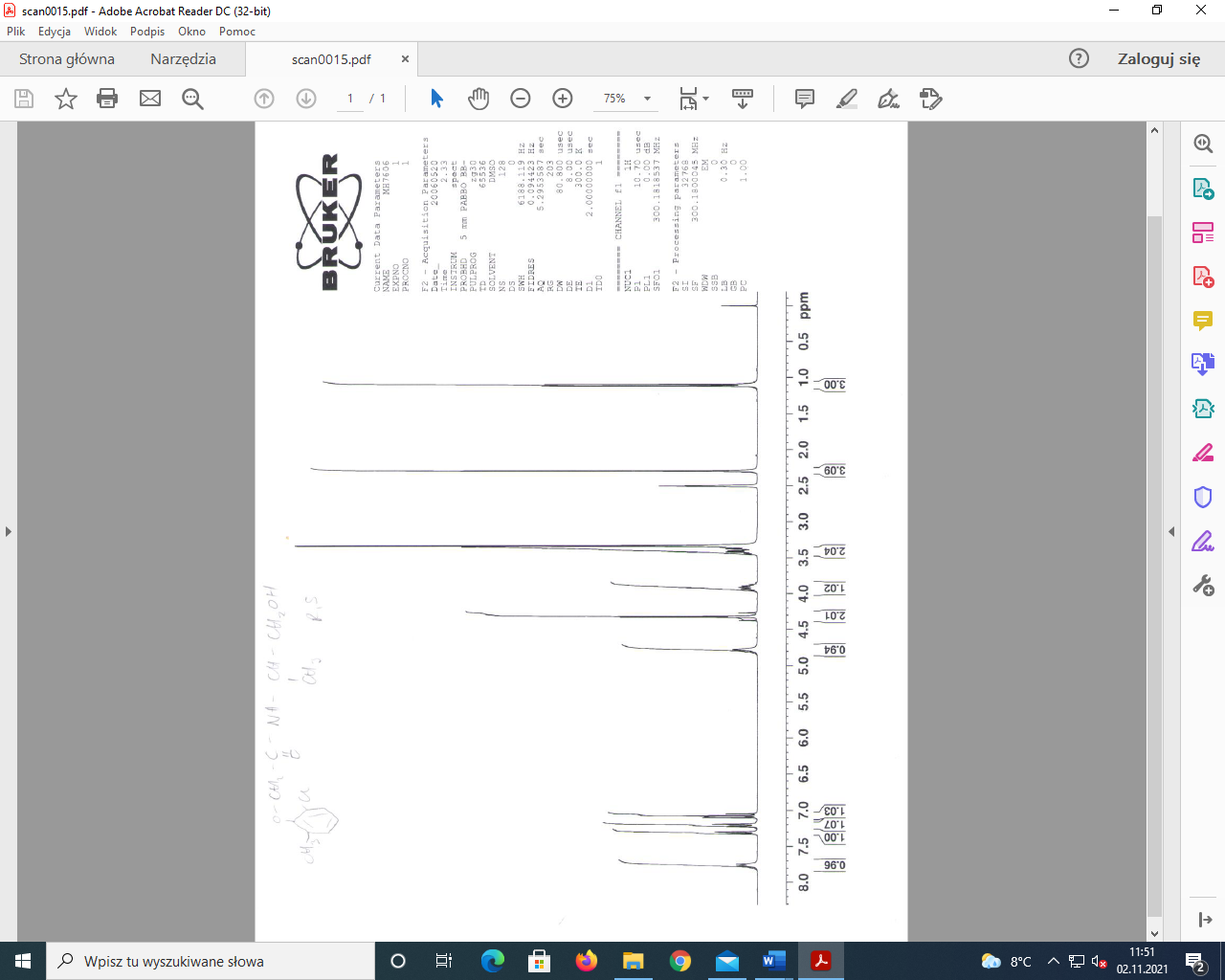


**Compound 12**

LCMS


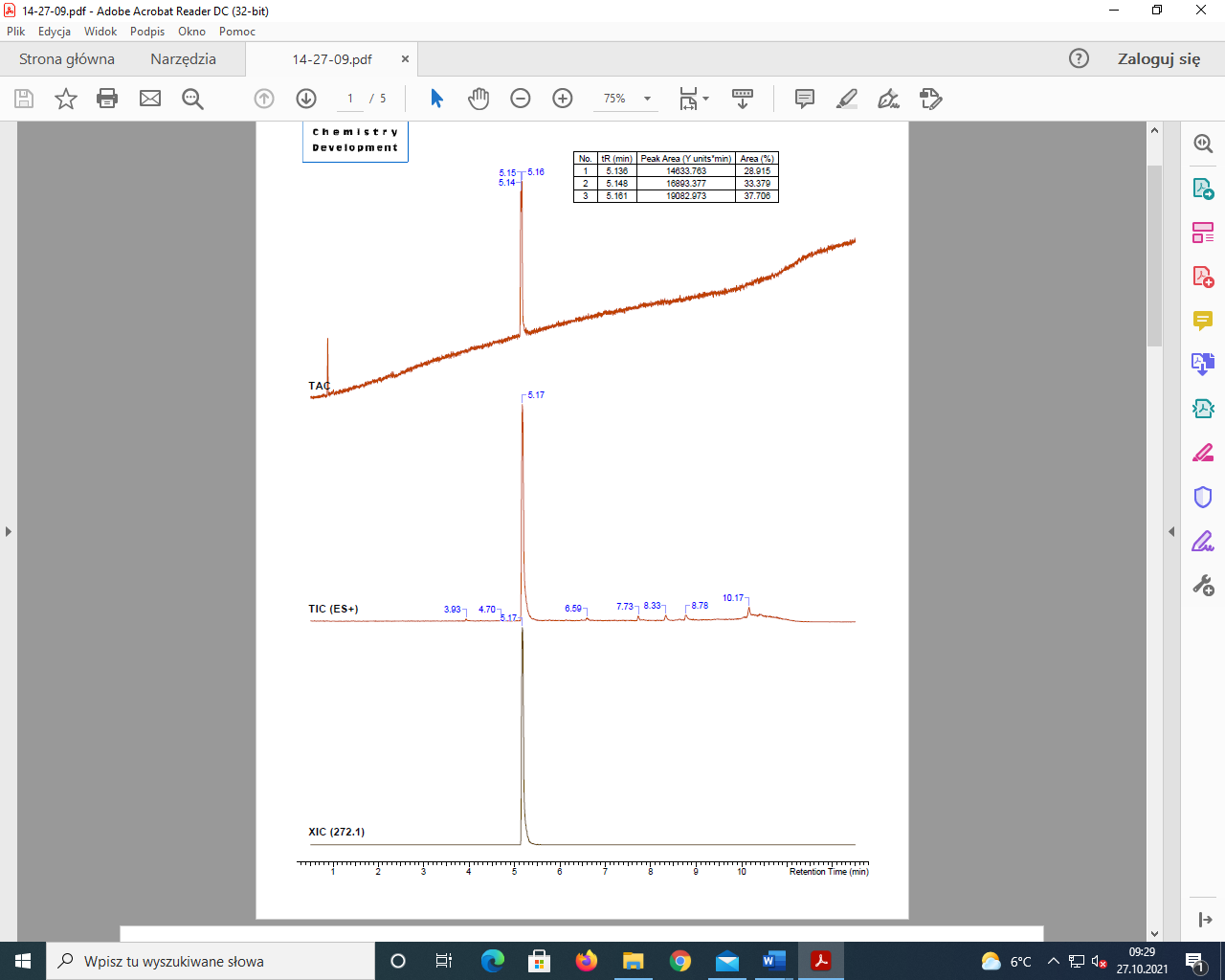


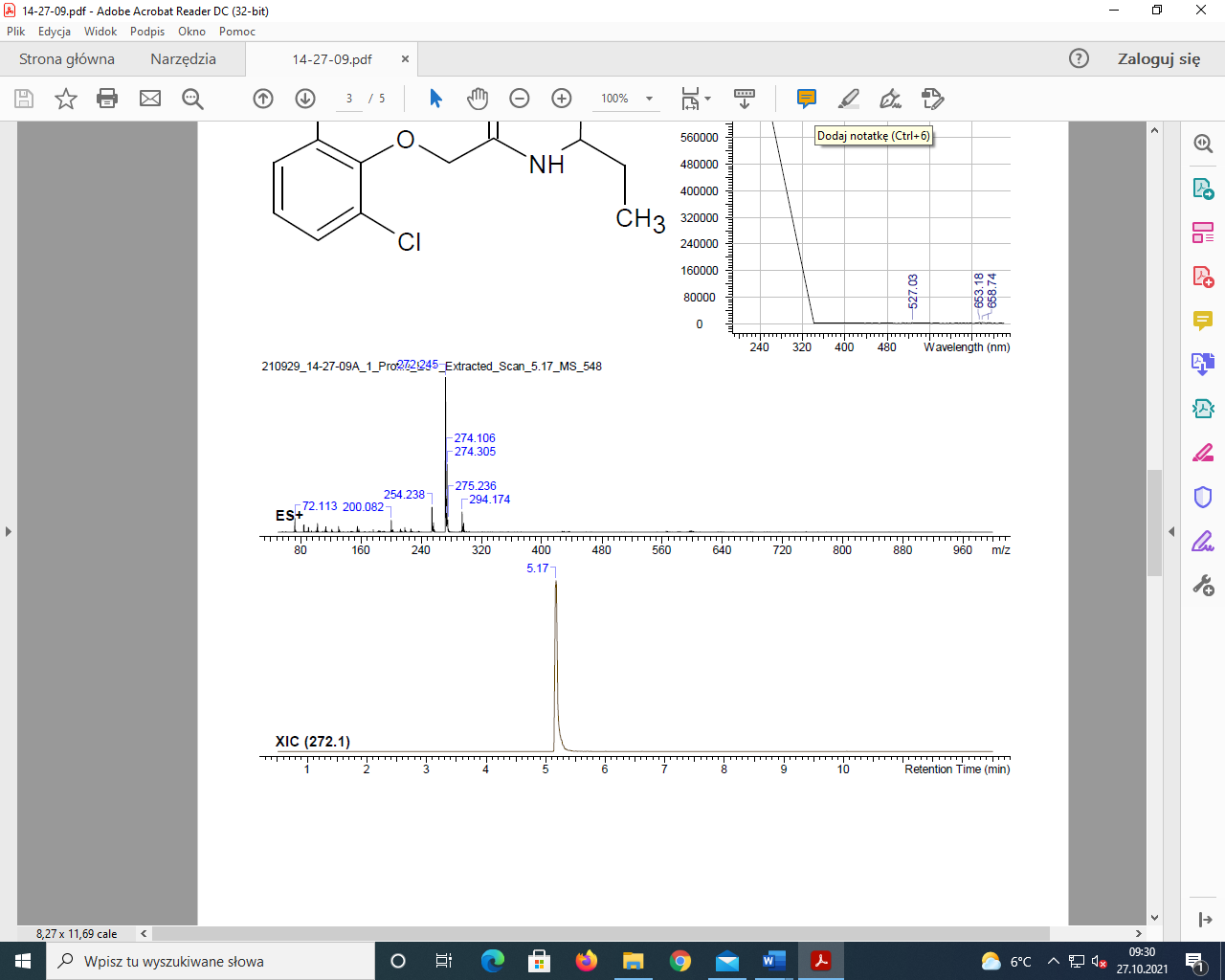


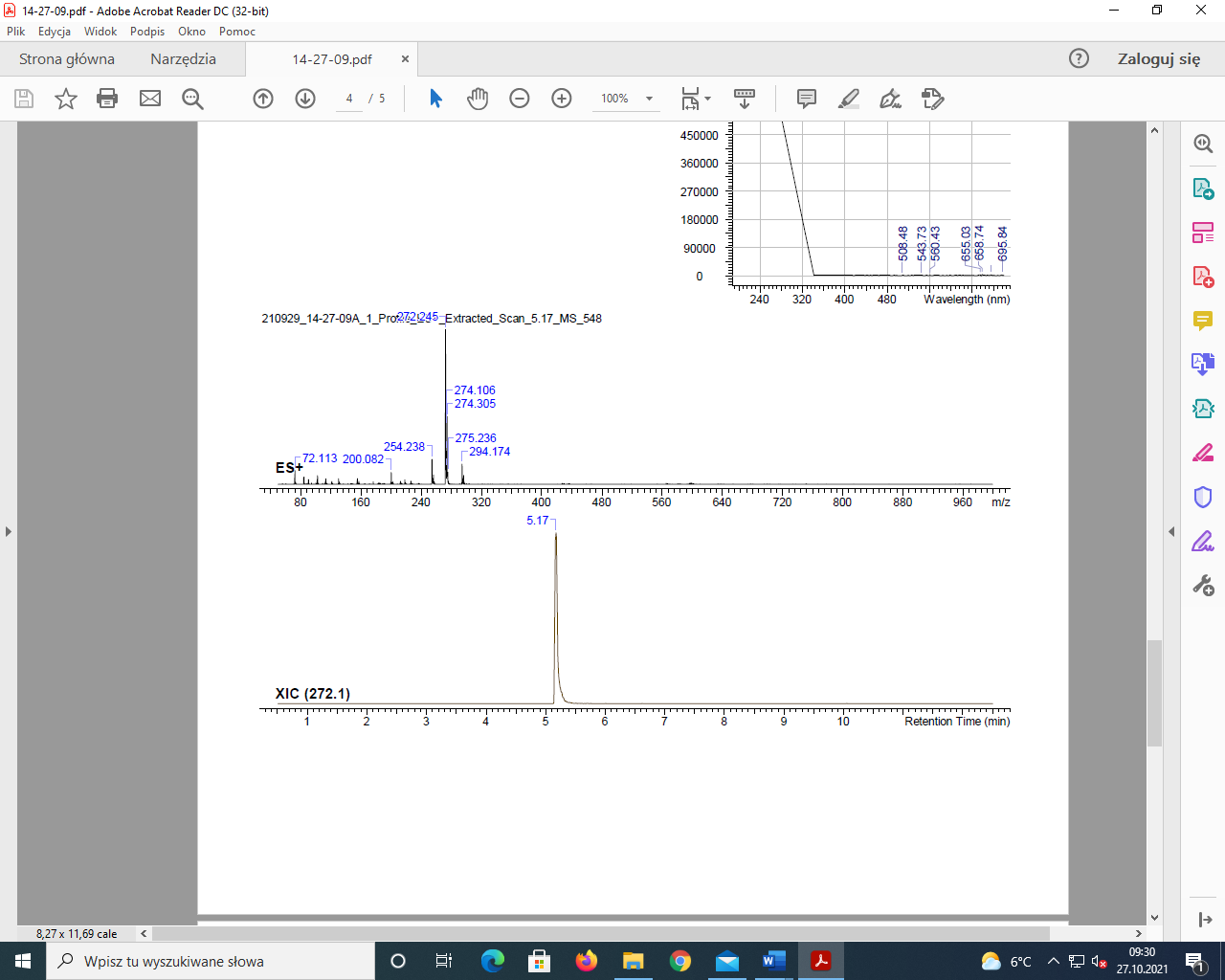


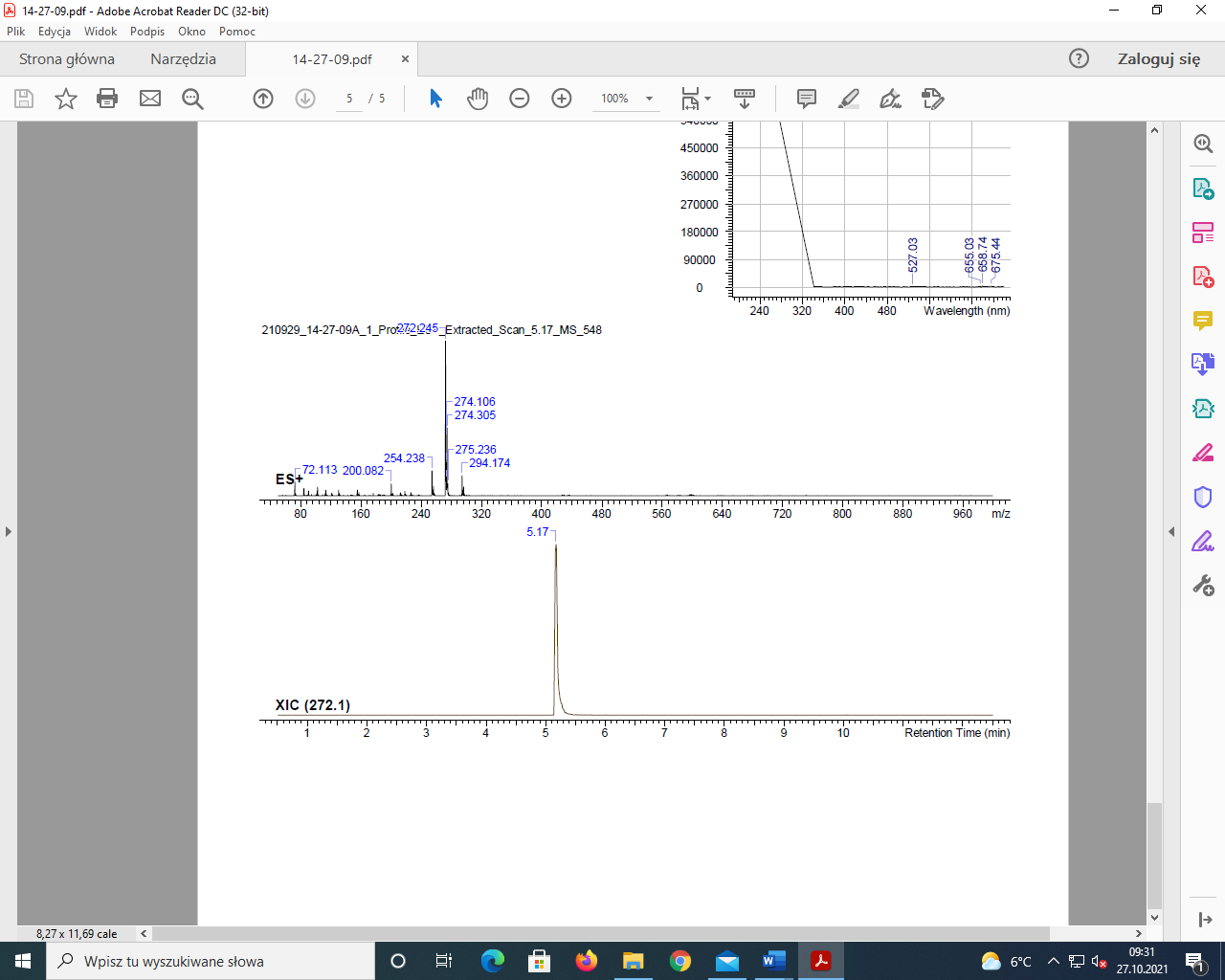


IR


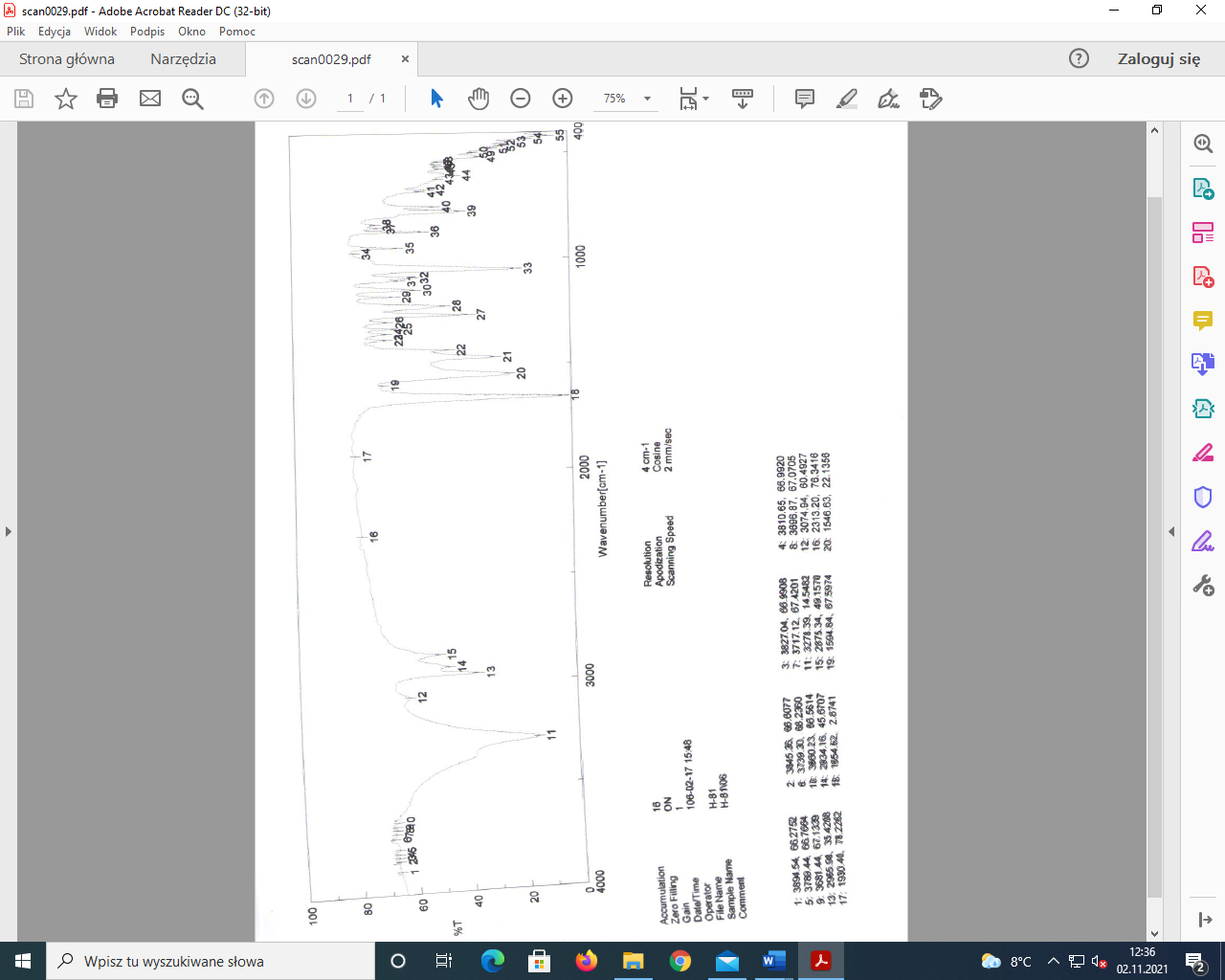


^1^HNMR


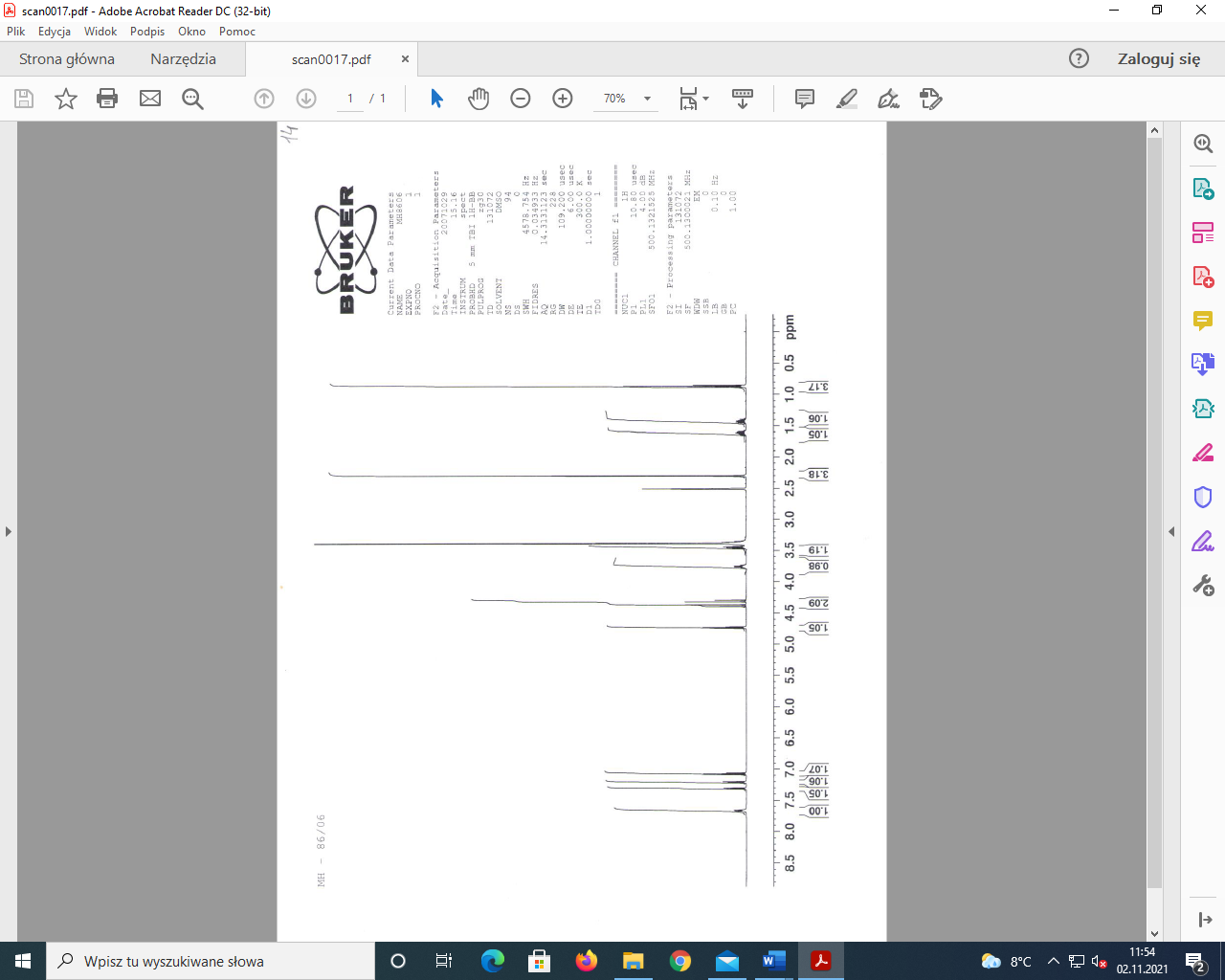


**Compound 13**

LCMS


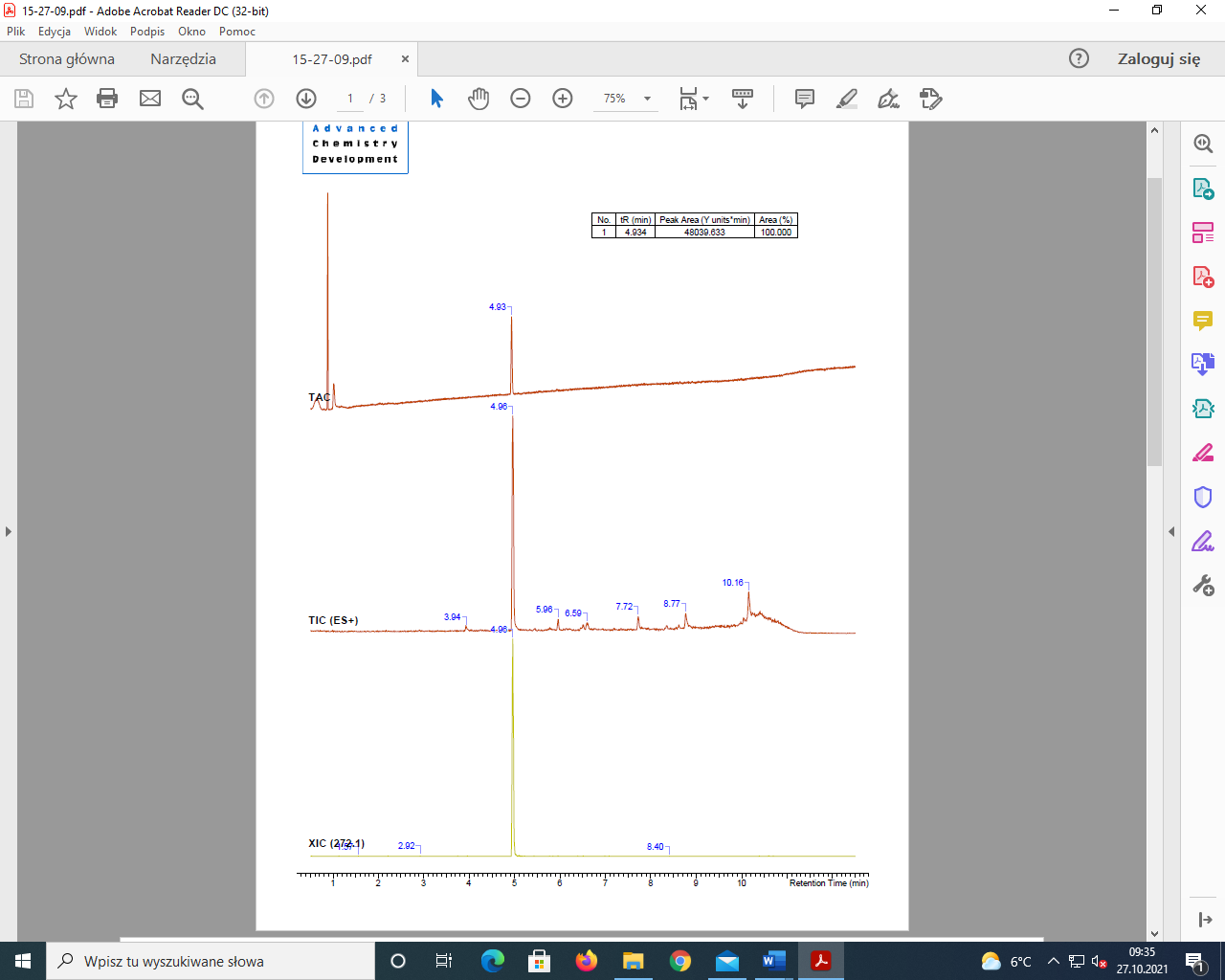


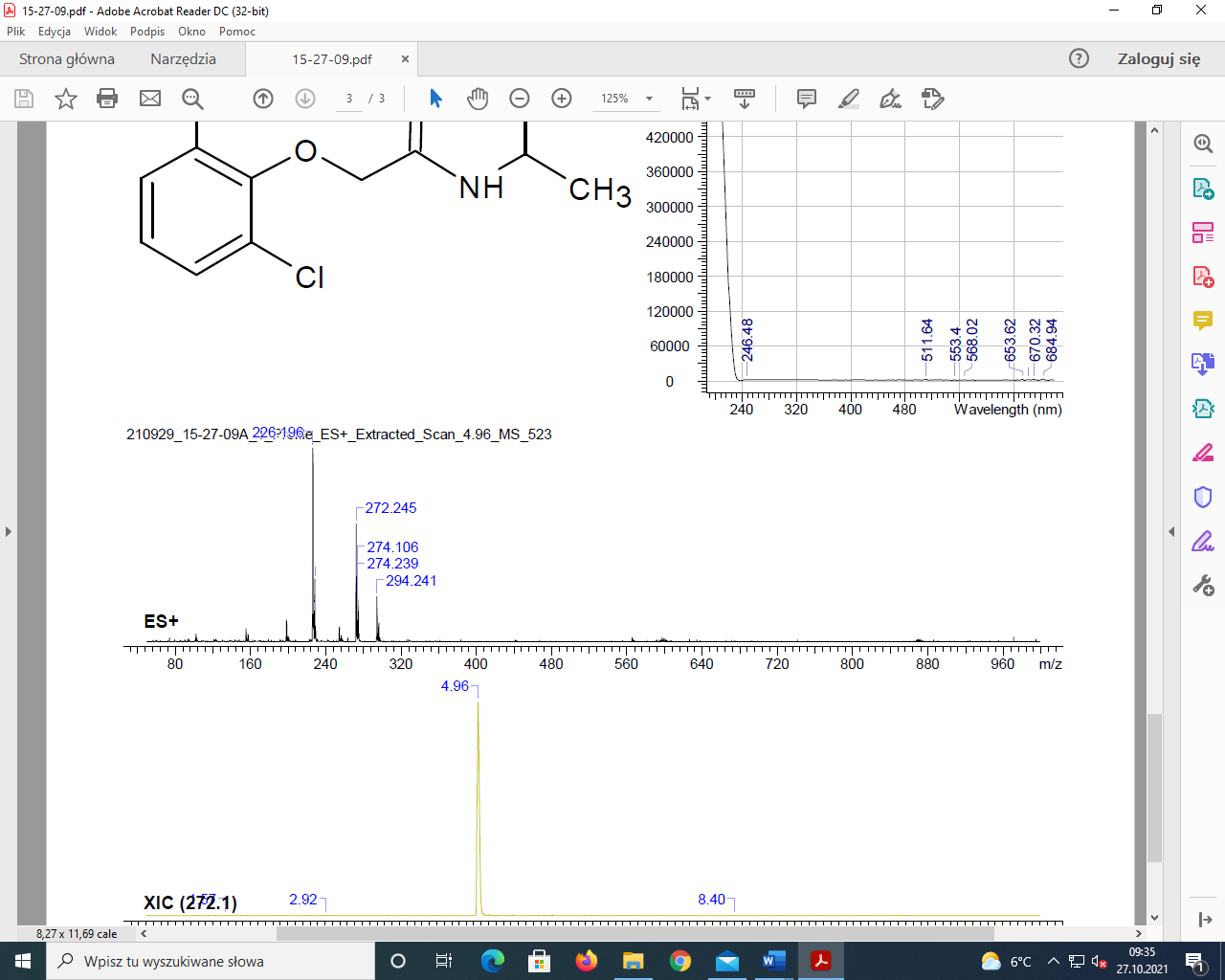


IR


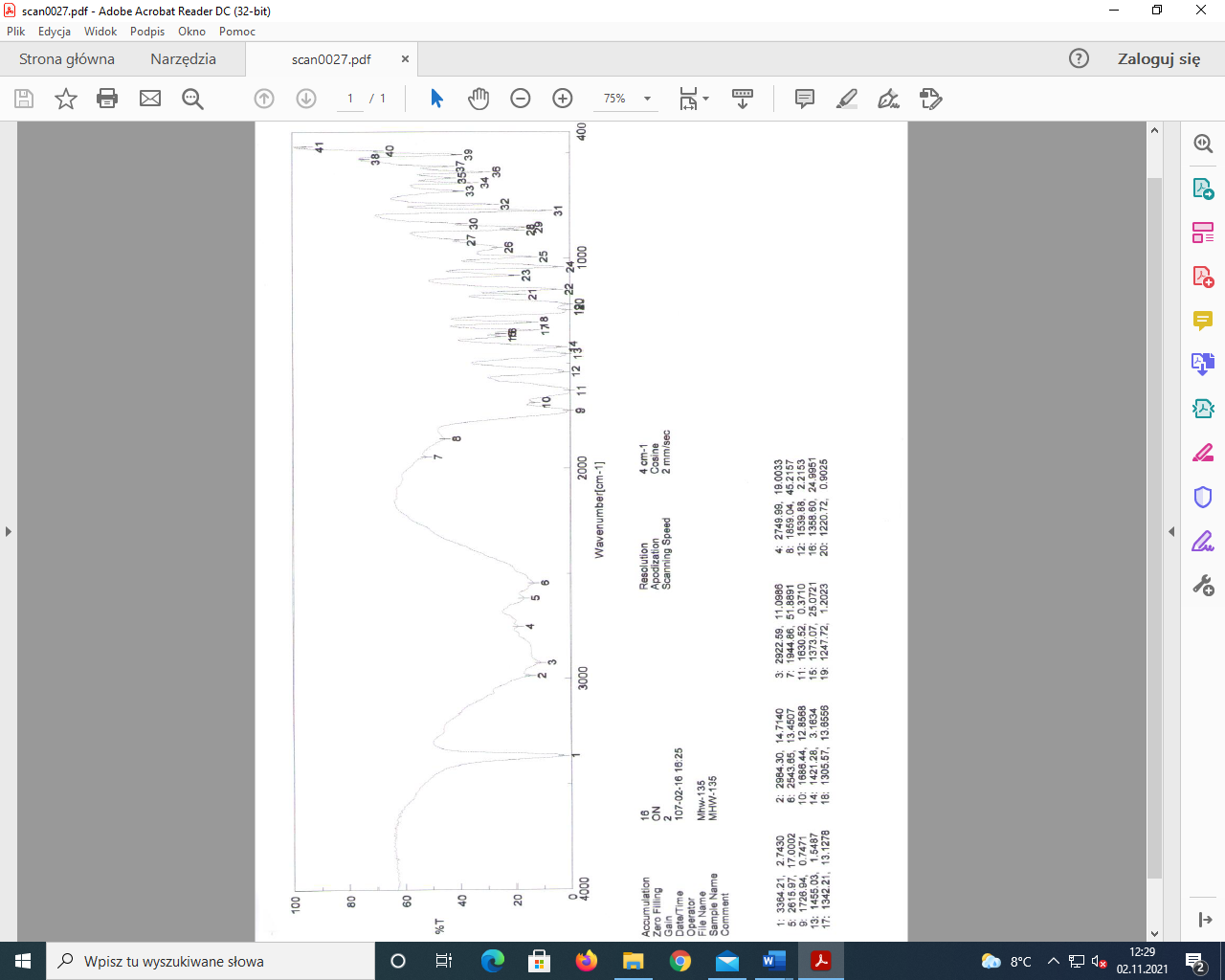


^1^HNMR


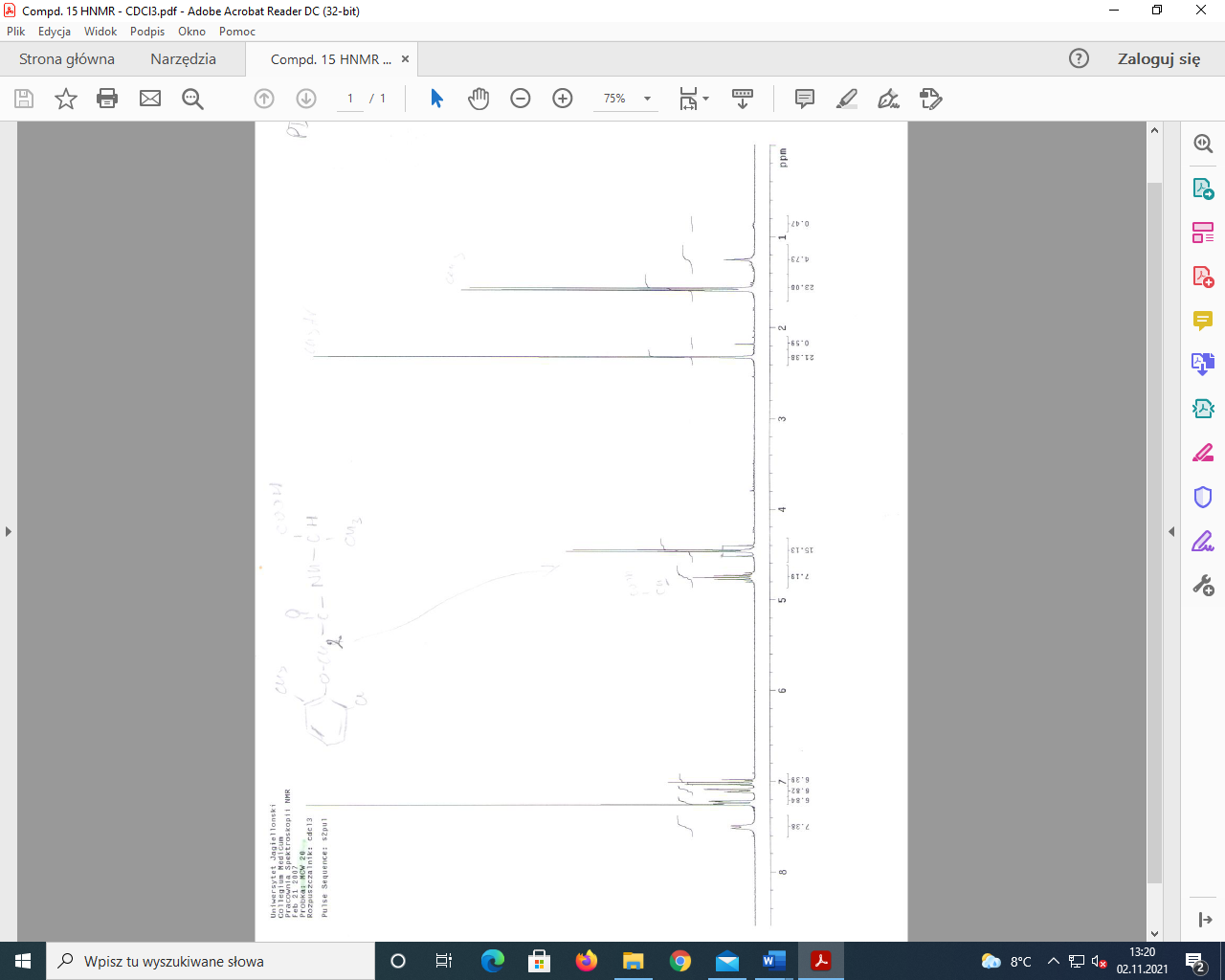


**Compound 14**

LCMS


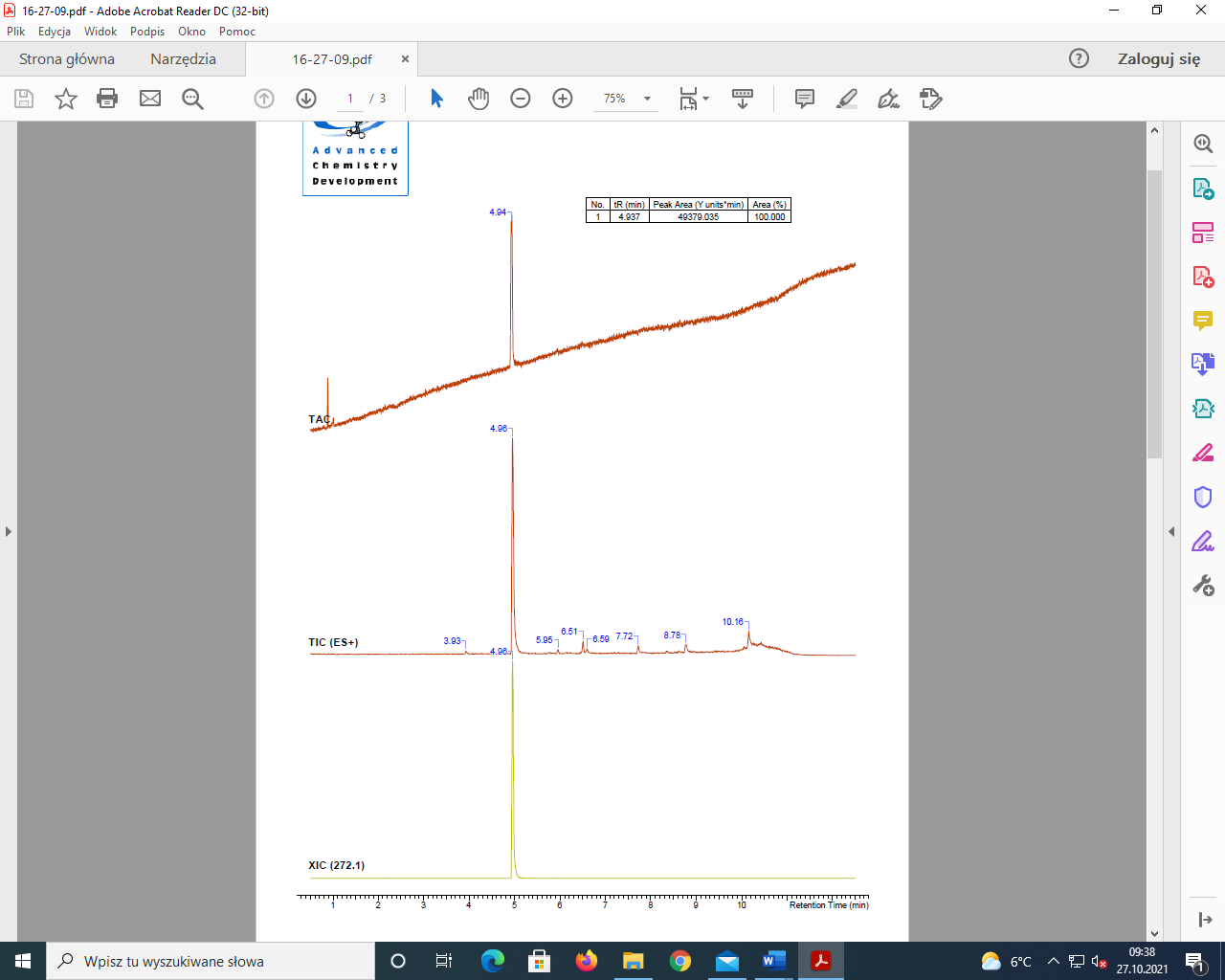


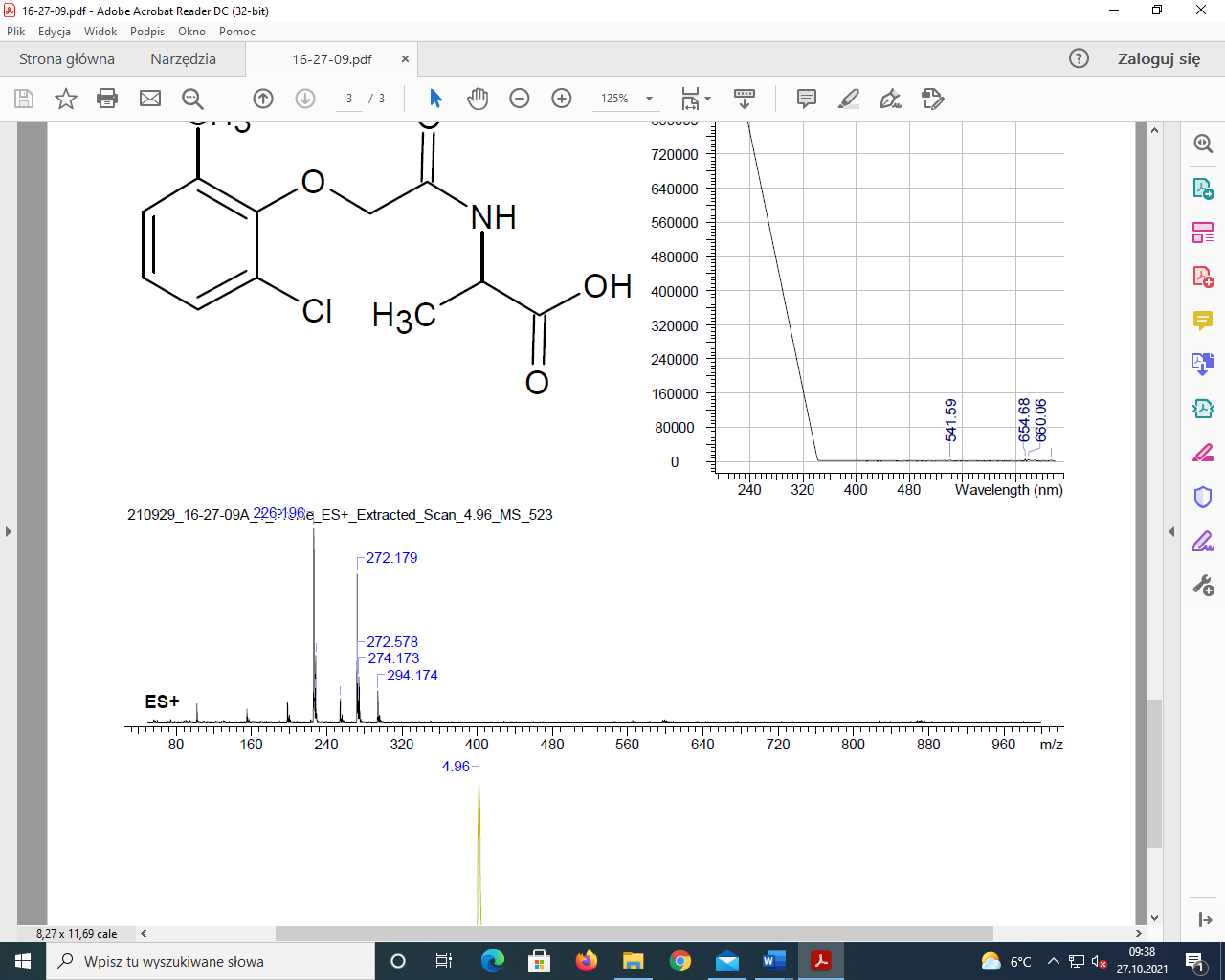


^1^HNMR


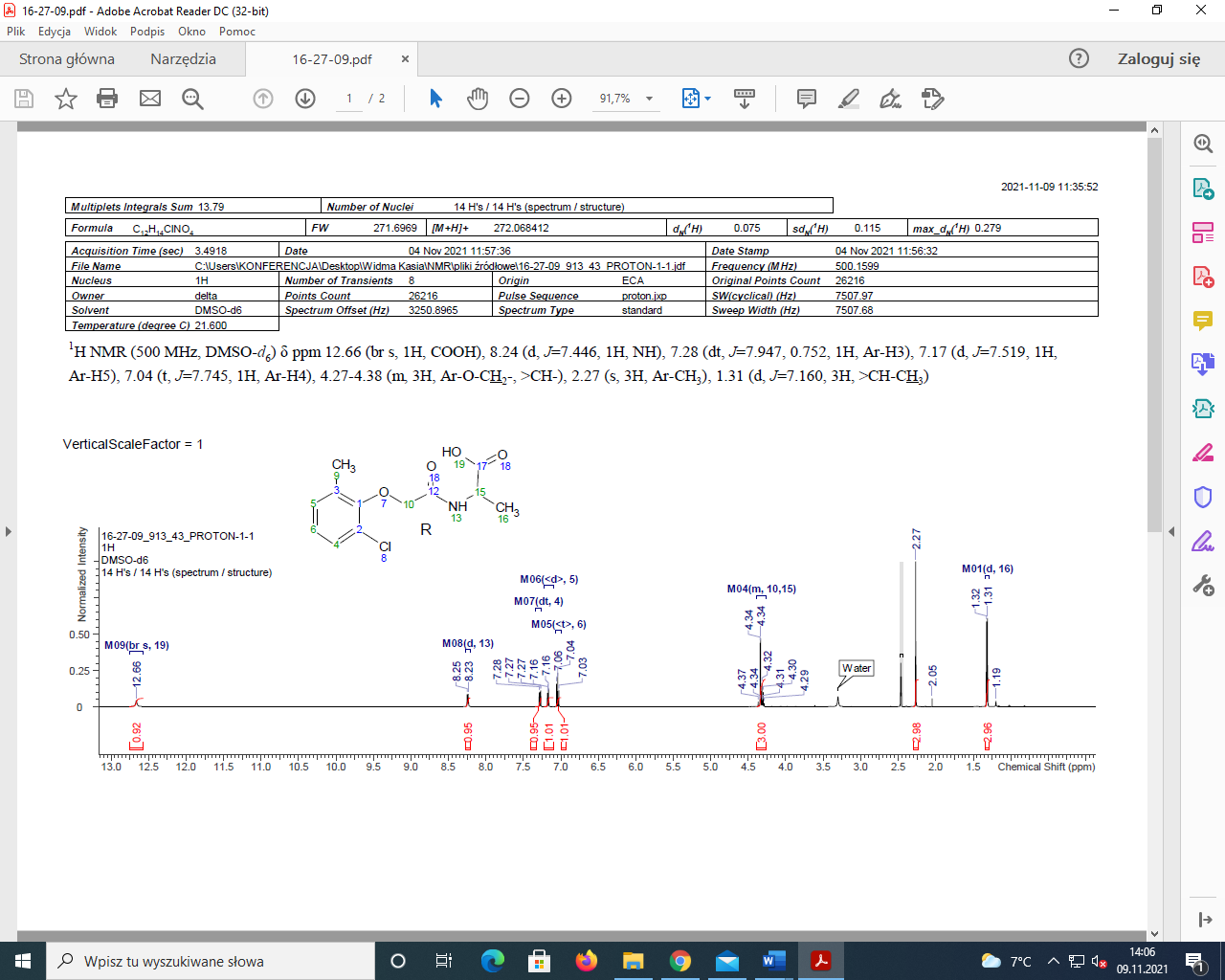


**Compound 15**

LCMS


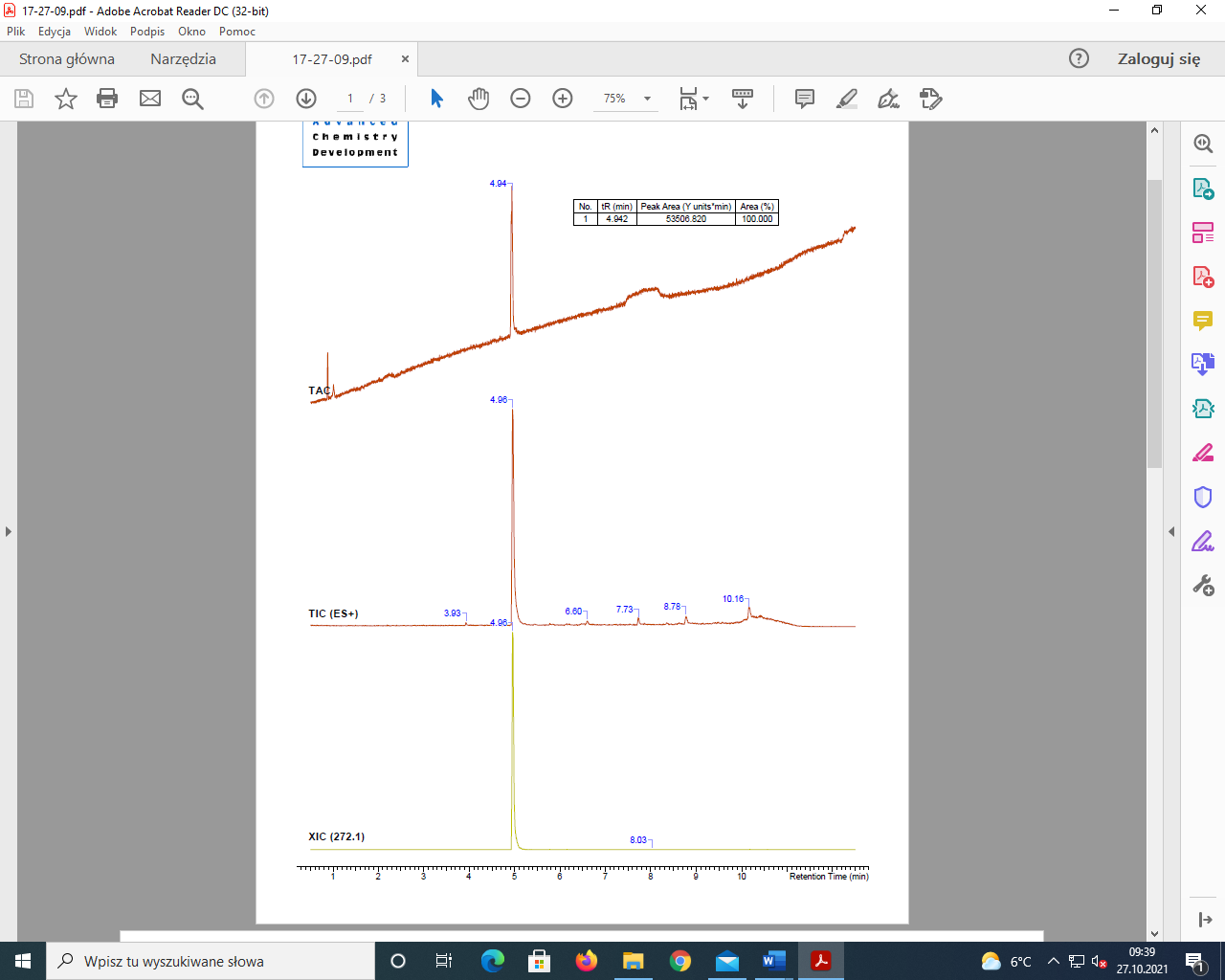


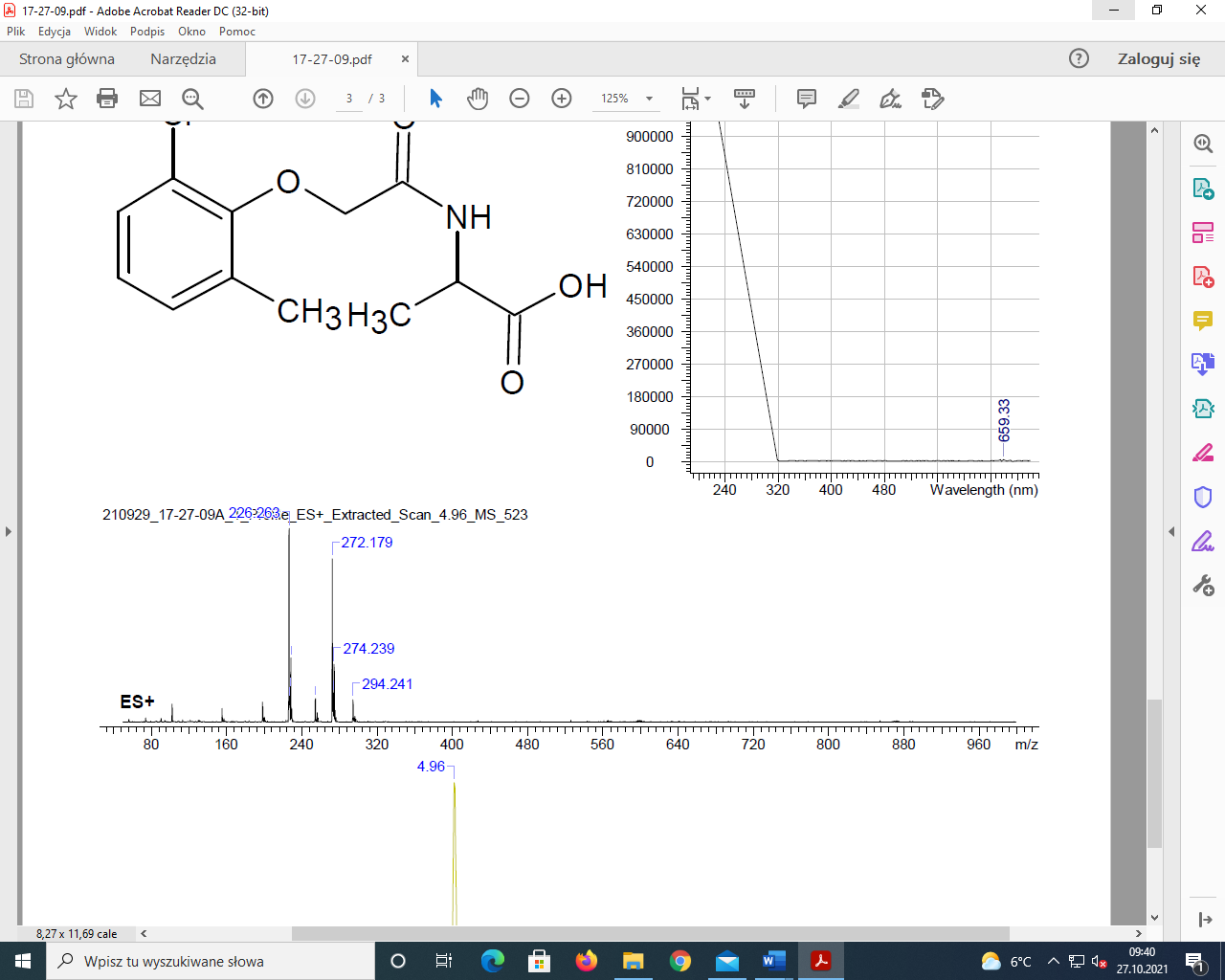


^1^HNMR


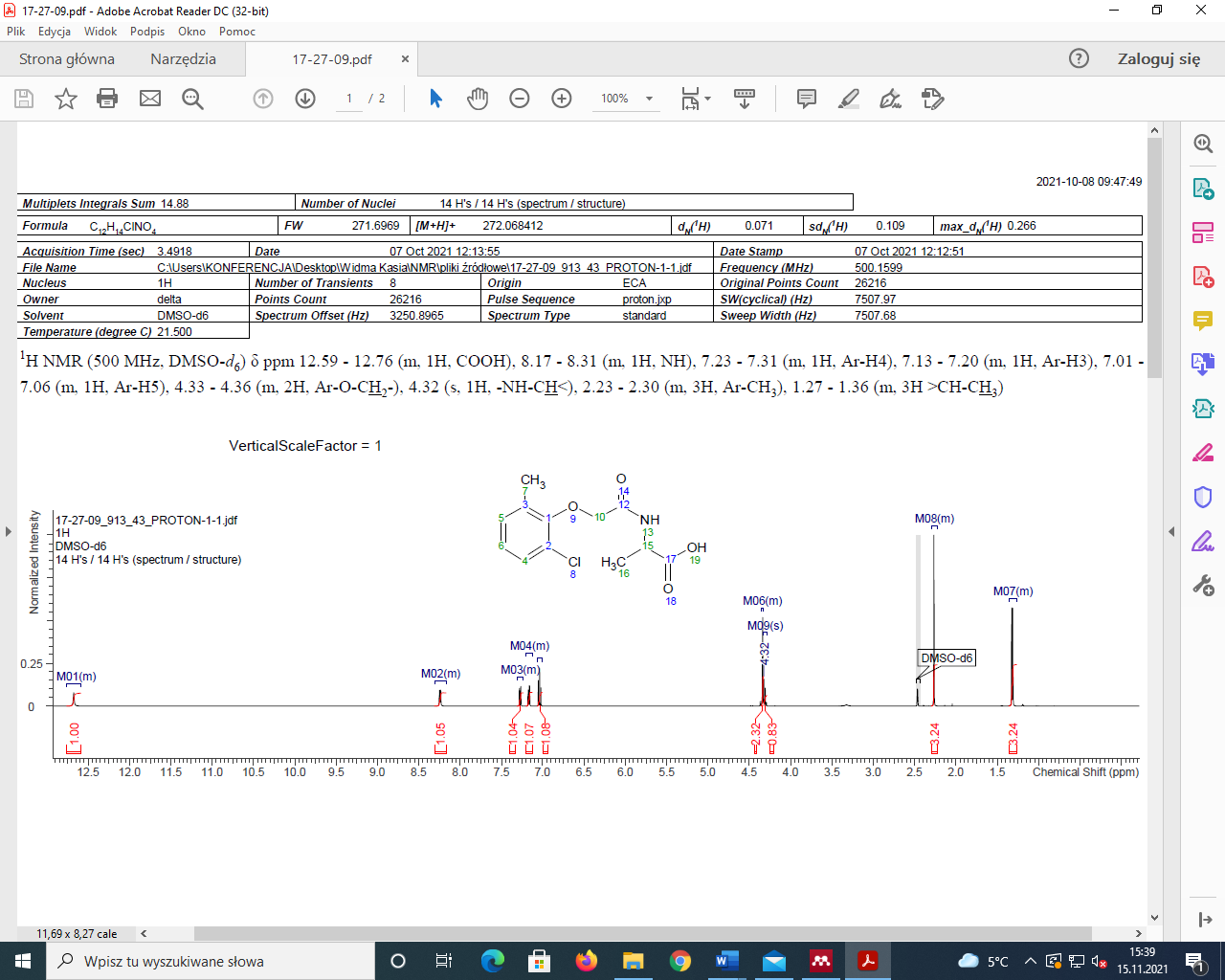


^13^CNMR


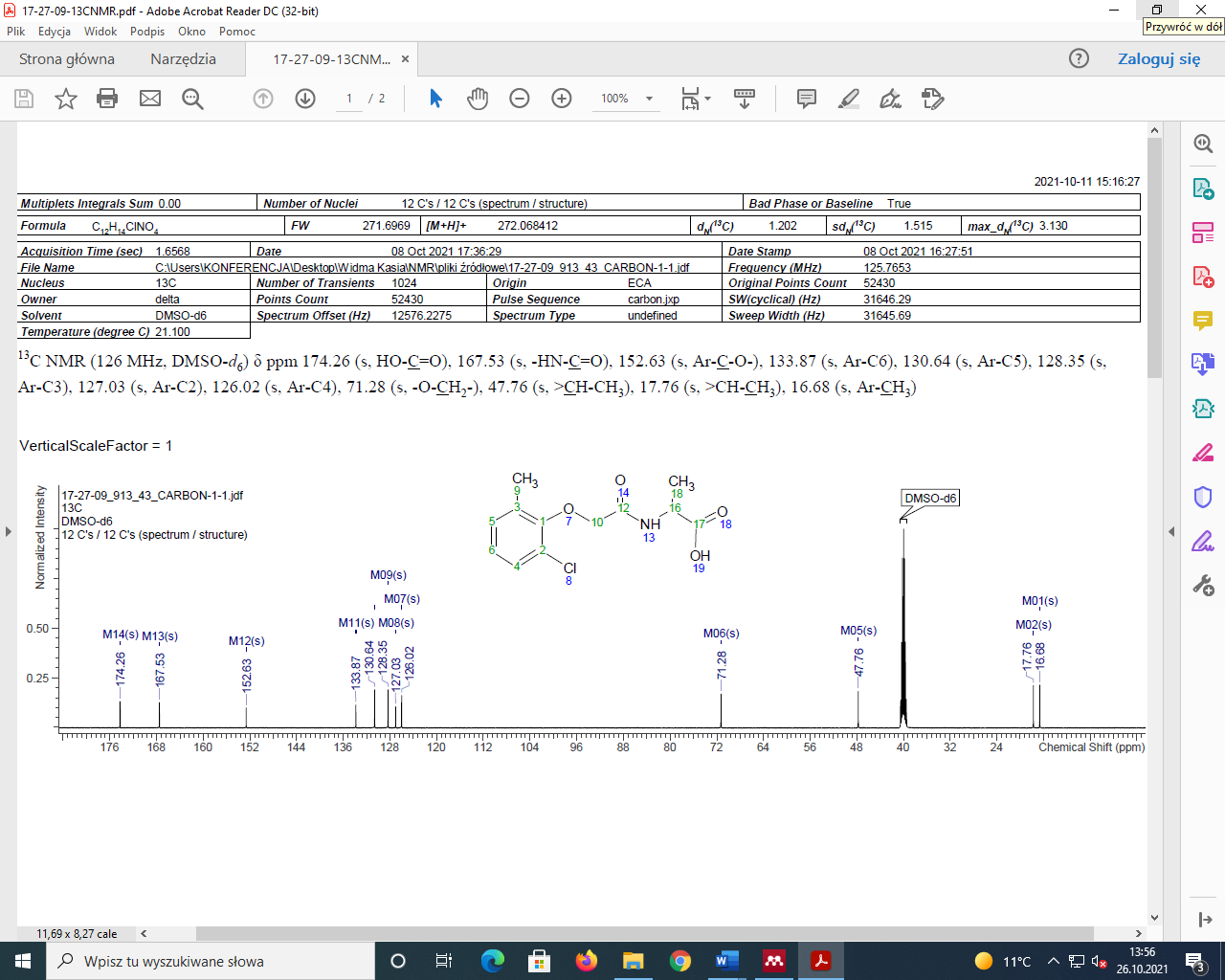


**Compound 16**

LCMS


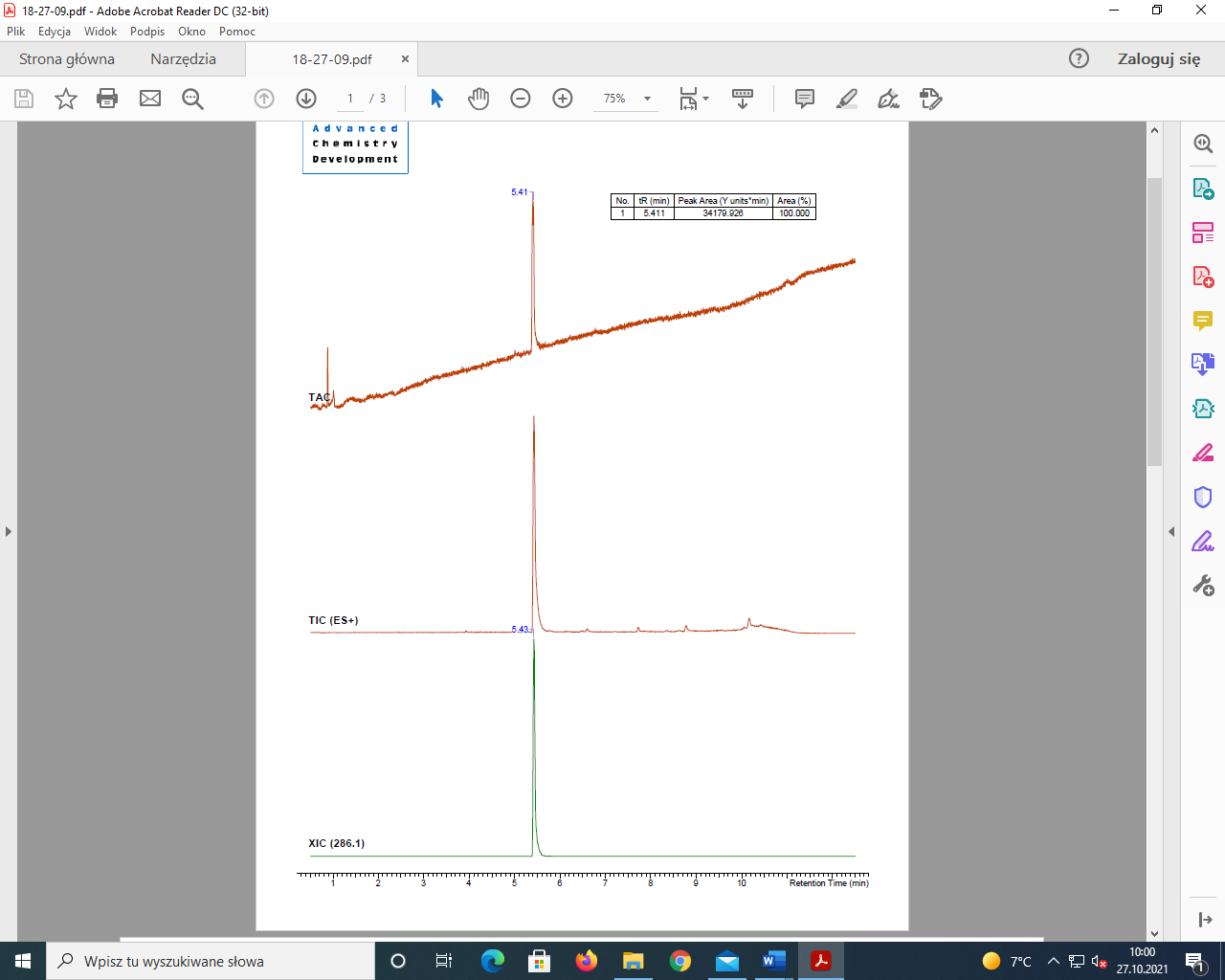


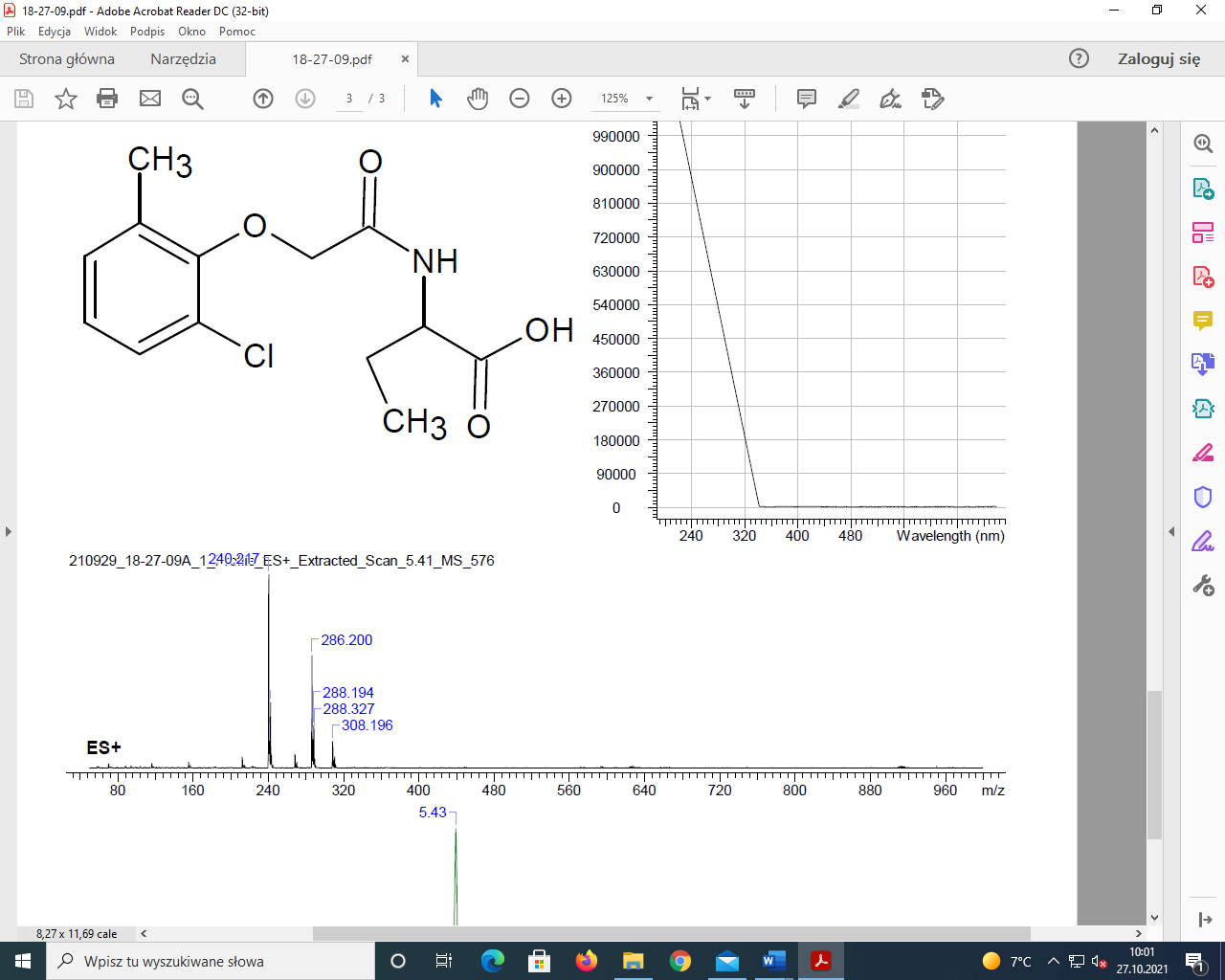


IR


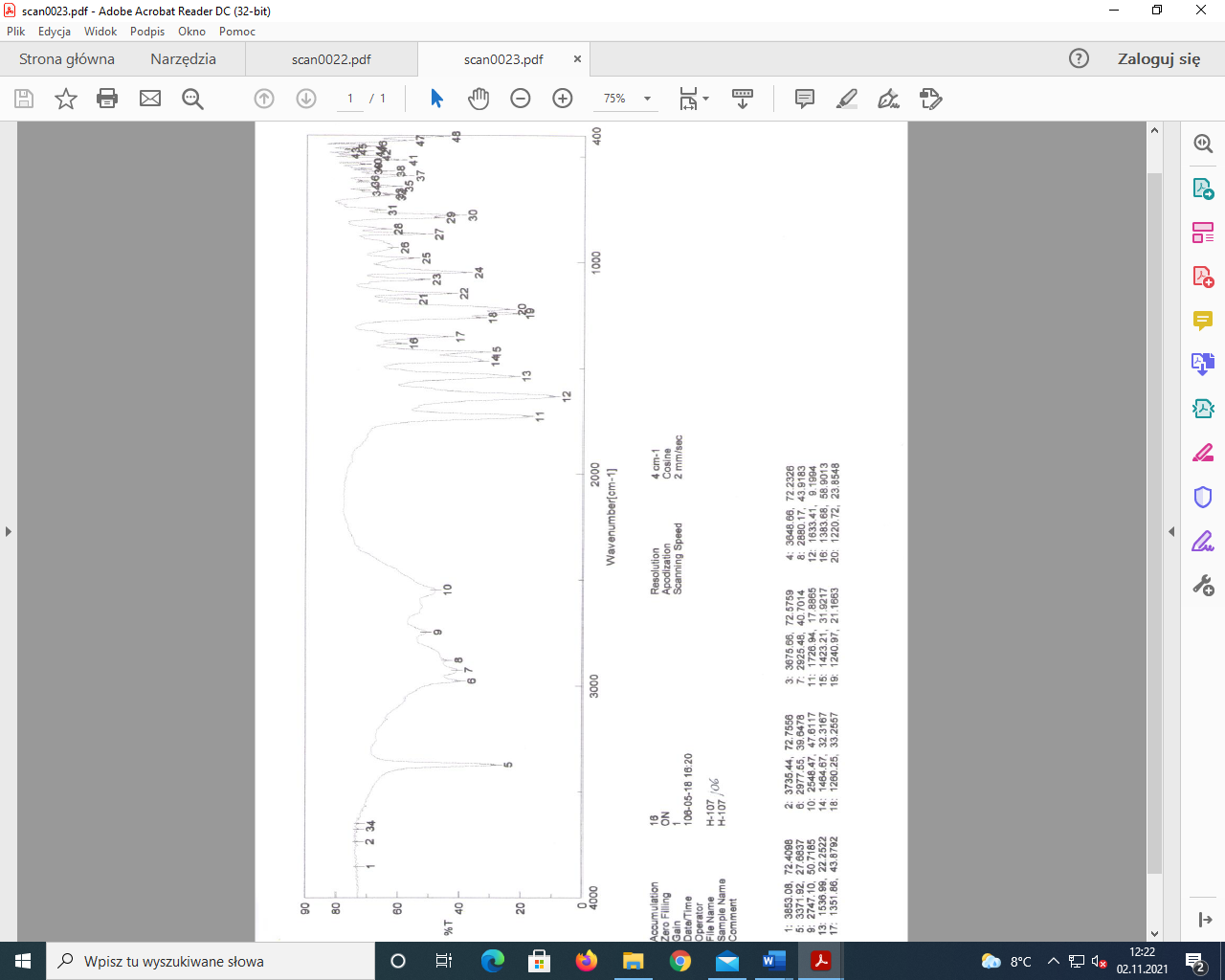


^1^HNMR


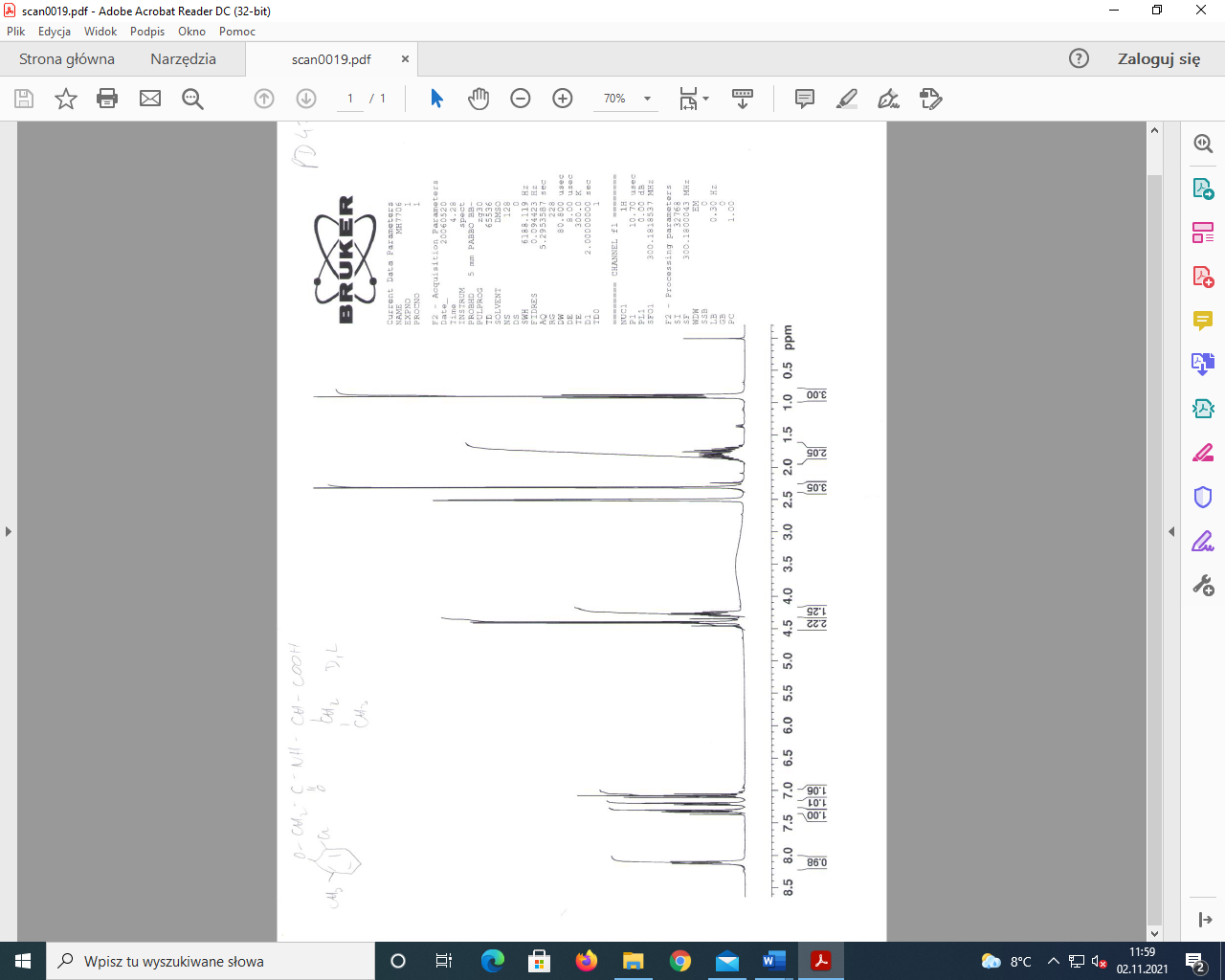


**Compound 17**

IR


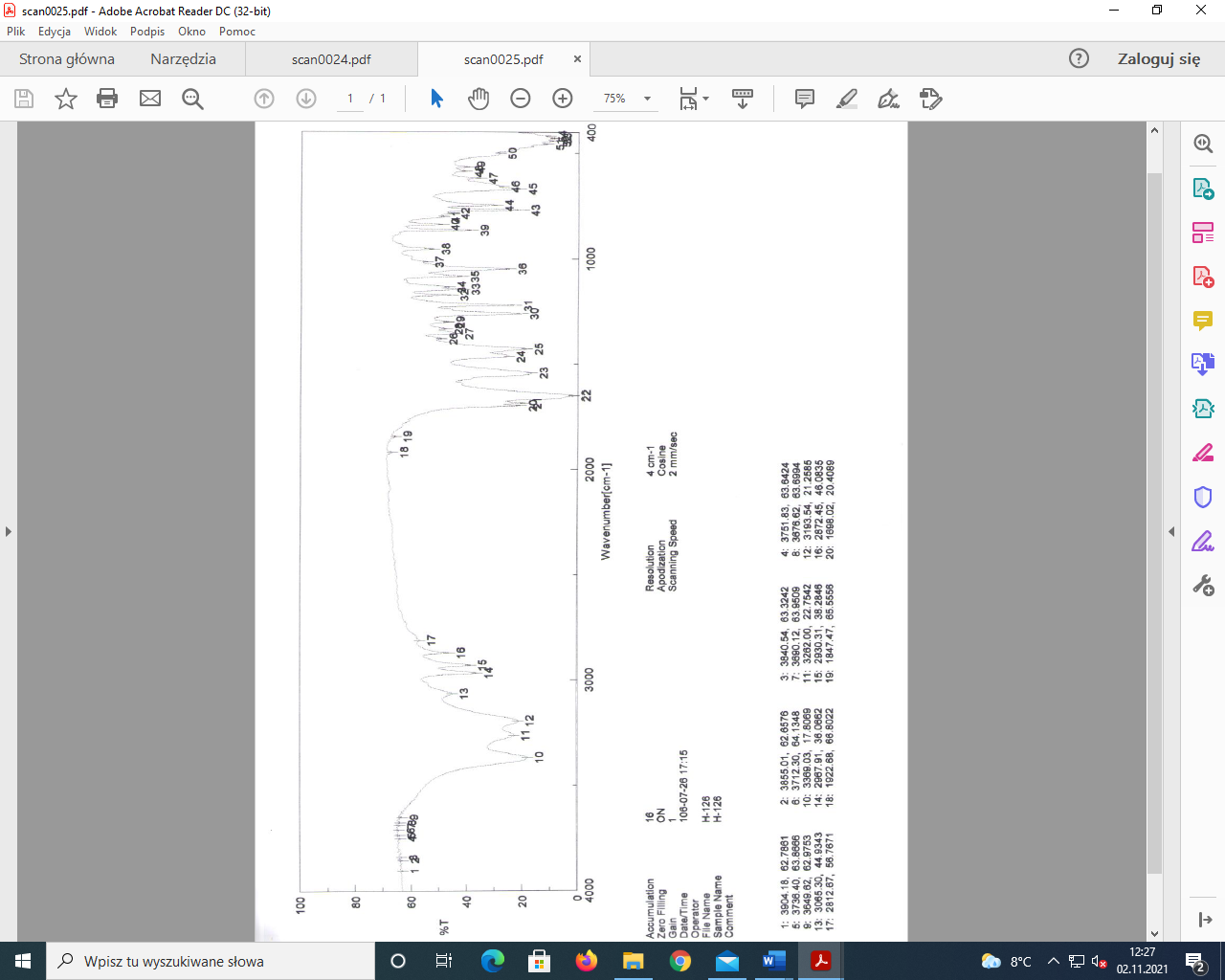


^1^HNMR


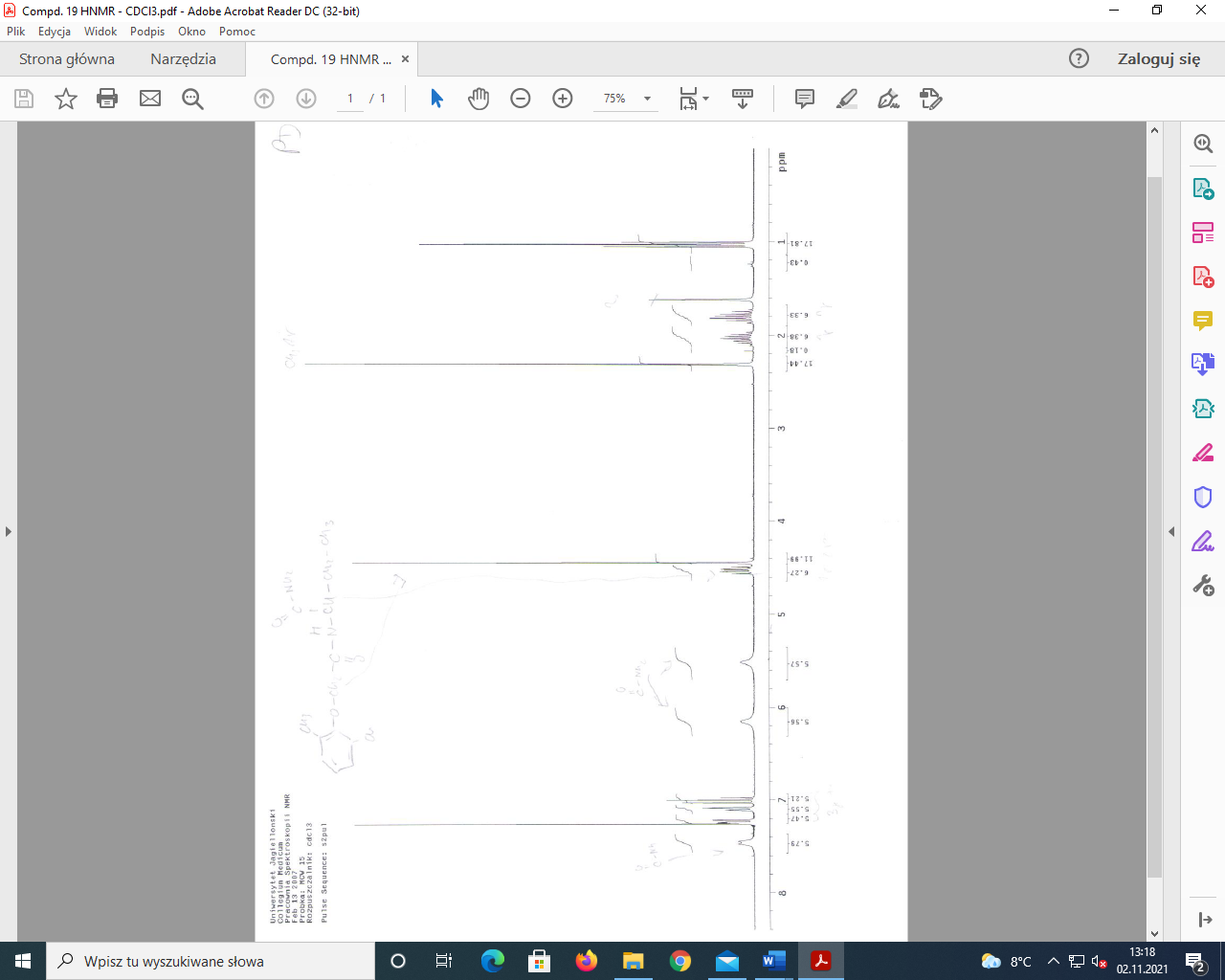


**Compound 18**

LCMS


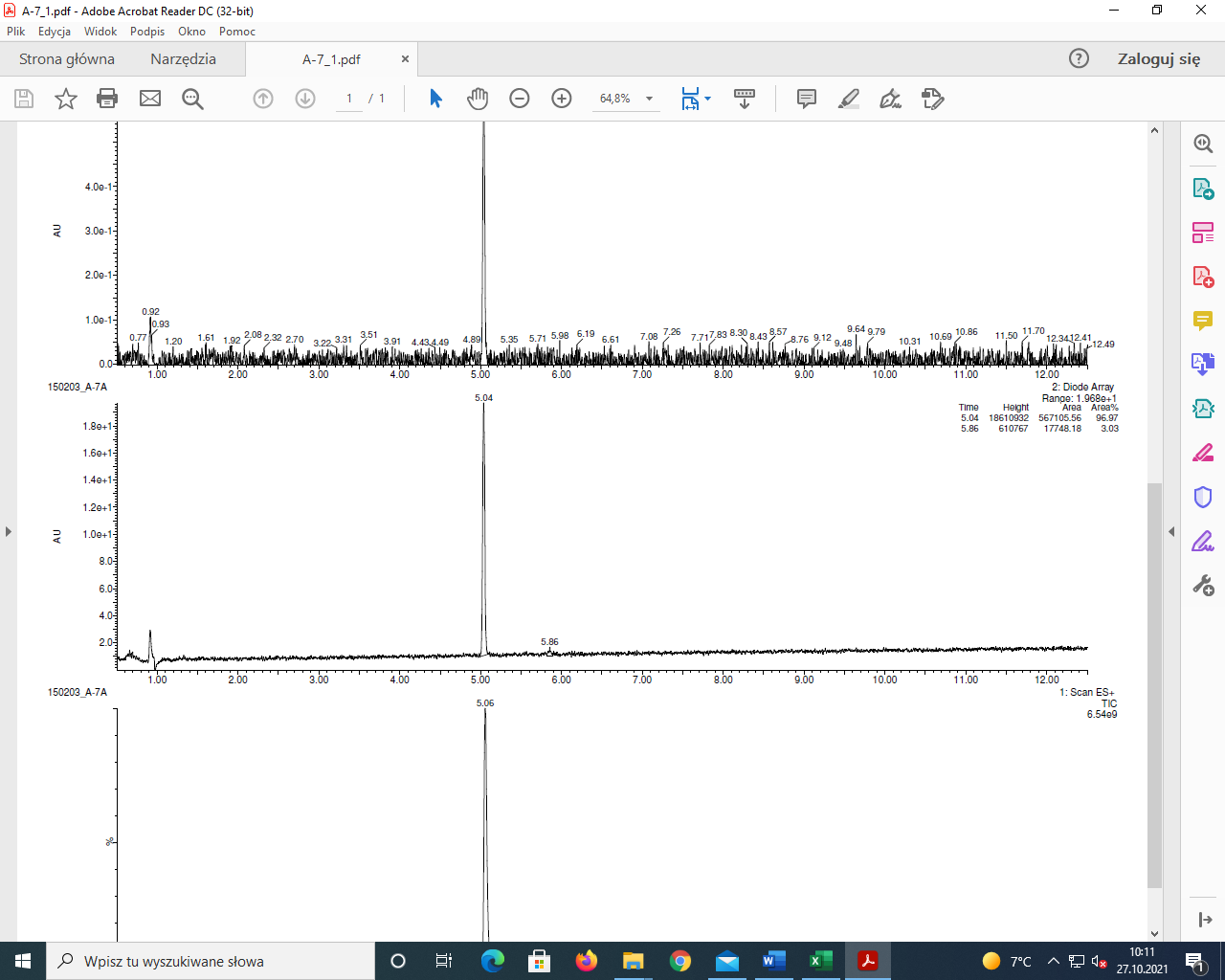


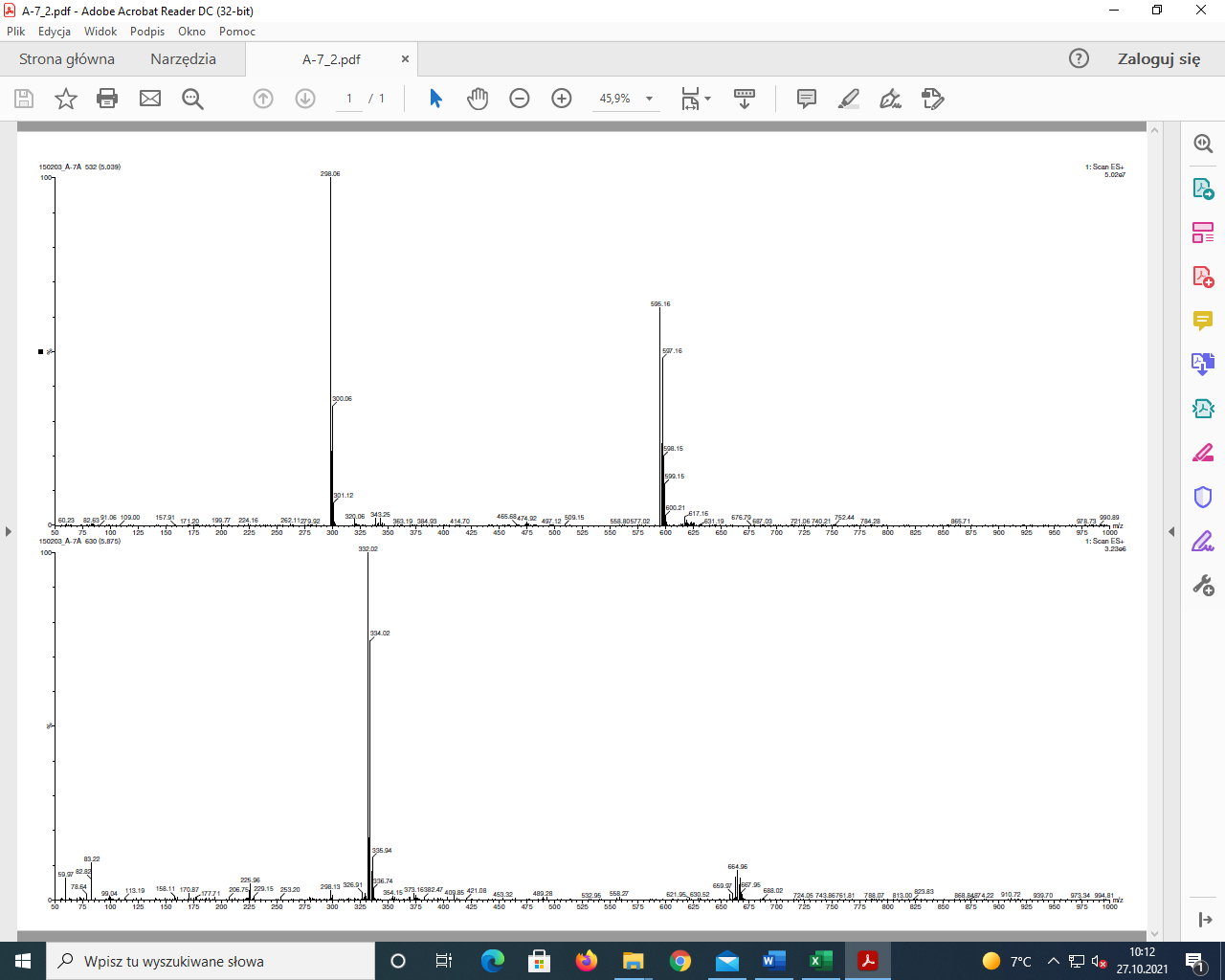


^1^H NMR


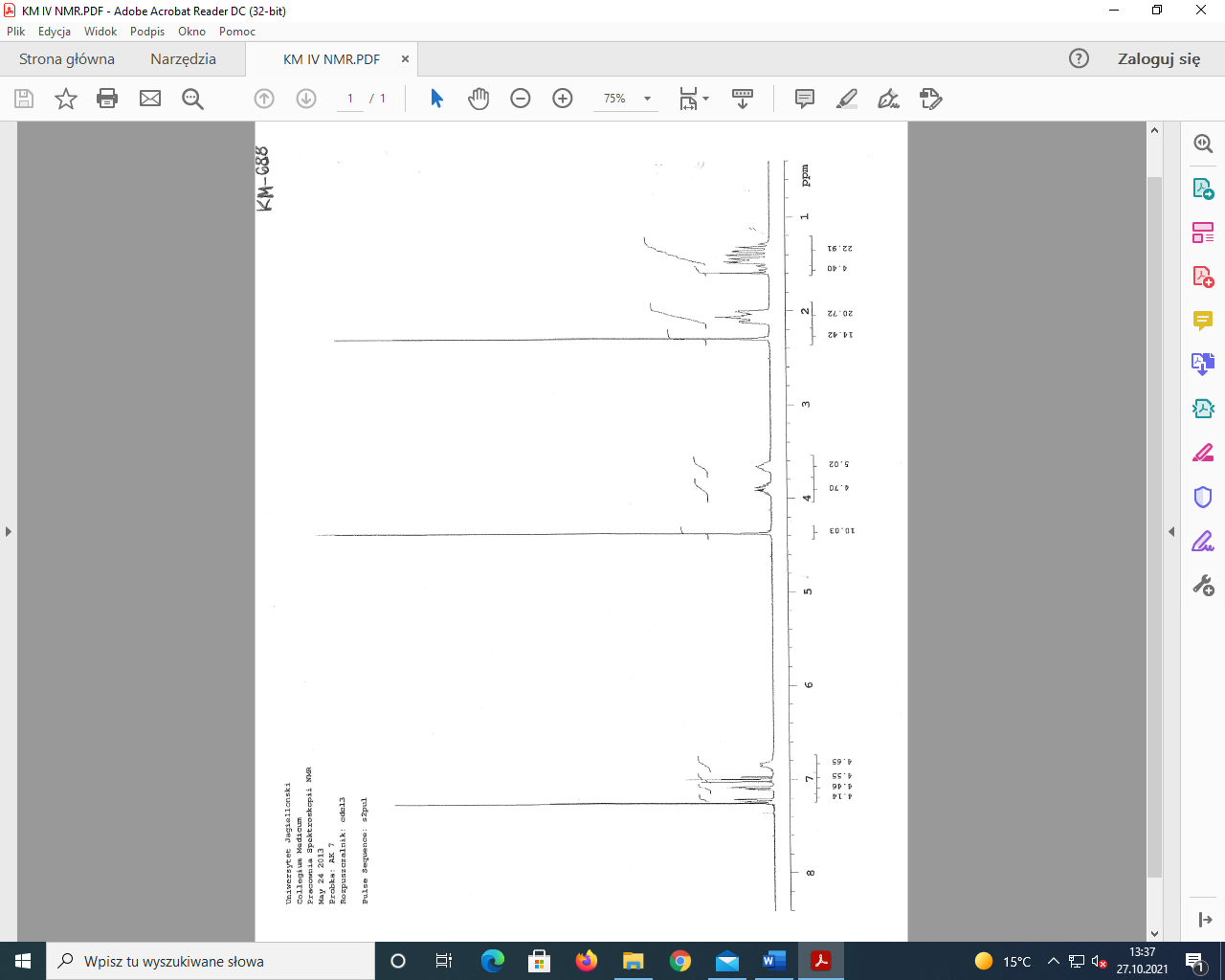

Supplement: Supplementary file 1 — Supplementary file1 (DOCX 12181 KB) [file 43440_2022_431_MOESM1_ESM.docx]
