# Supplementary material for: KM-408, a novel phenoxyalkyl derivative as a potential anticonvulsant and analgesic compound for the treatment of neuropathic pain
Source: Pharmacol Rep. 2022 Nov 19;75(1):128–65. doi: 10.1007/s43440-022-00431-7 (PMC9889419; doi:10.1007/s43440-022-00431-7)
Supplement: Supplementary file 2 — Supplementary file2 (PDF 11580 KB) [file 43440_2022_431_MOESM2_ESM.pdf]

KM-314

Anticonvulsant Screening Project

Test 1 Results - Mice I.P. Identification

dd ID: 348014 U Screen ID: 1

ılvent Code: MC Solvent Prep: M&P,SB

ıimal Weight: 18.5 to 23.0 g

ıte Started: 19-Dec-2003 Date Completed: 19-Dec-2003

ference: 378:28

sponse

Time (Hours)				0.5		4.0		0.25		1.0		2.0		6.0		3.0		8.0		24	
est	Dose	Form	Dths	N / F	C	N / F	C	N / F	C	N / F	C	N / F	C	N / F	C	N / F	C	N / F	C	N / F	C
IES	3	SOL		0 / 4		/		/		/		/		/		/		/		/	
IES	10	SUS		2 / 4		/		/		/		/		/		/		/		/	
ES	30	SUS		1 / 1		0 / 1		/		/		/		/		/		/		/	
ES	100	SUS		1 / 1		0 / 1		/		/		/		/		/		/		/	
MET	30	SUS		0 / 1		0 / 1		/		/		/		/		/		/		/	
OX	3	SOL		0 / 4		/		/		/		/		/		/		/		/	
OX	10	SUS		0 / 4		/		/		/		/		/		/		/		/	
OX	30	SUS		0 / 4		0 / 2		/		/		/		/		/		/		/	
OX	100	SUS	6	8 / 8	*	0 / 1		/		/		/		/		/		/		/	
OX	300	SUS	4	4 / 4	1	/		/		/		/		/		/		/		/	

sponse Comments

EST	DOSE (mg/kg	TIME	CODE	COMMENT
OX	100	0.5	34	Muscle spasms
OX	100	0.5	14	Unable to grasp rotorod
OX	100	0.5	1	Death
OX	300	0.5	1	Death

nments to Supplier:

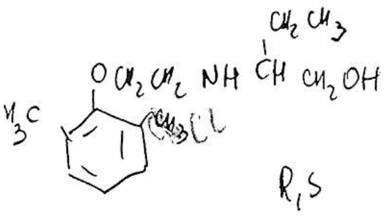

KM-314

Anticonvulsant Screening Project

Test 8 Results -Anticonvulsant Identification (Rats I.P.)

Add ID: 348014 U **Screen ID:** 1

Solvent Code: MC Solvent Prep: M&P,SB

Animal Weight: 125 to 150 g

Date Started: 27-Jan-2004 Date Completed 27-Jan-2004

Reference: 379:50

Time to Peak Effect

Test	Dose (mg/kg)	# Dths	0.25		0.5		1.0		2.0		4.0		6.0		8.0		24		3.0	
			N / F	C	N / F	C	N / F	C	N / F	C	N / F	C	N / F	C	N / F	C	N / F	C	N / F	C
MES	15		4 / 4		4 / 4		1 / 4		0 / 4		0 / 4		/		/		/		/	
TOX	15		0 / 4		0 / 4		0 / 4		0 / 4		0 / 4		/		/		/		/	

Comments to Supplier:

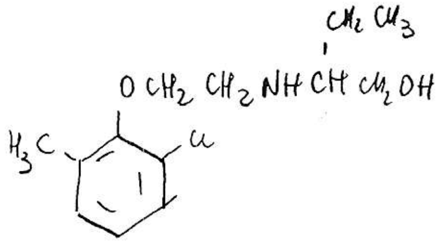

R, S

KM-314

Anticonvulsant Screening Project

Test 10 Results -Anticonvulsant Quantification (Rats I.P.)

Add ID:	348014 U	Screen ID:	1

Solvent Code: MC Solvent Prep: M&P,SB Route Code: IP

Animal Weight: to g

Date Started: 12-Feb-2004 Date Completed 19-Feb-2004

Reference: 379:80-89

ED50 Value

Test	Time (Hrs)	ED50	95% Confidence Interval LOW	HIGH	SLOPE	STD. ERR.
MES	0.25	7.3	5.39	9.89	4.34	1.13
SCMET	0.25	> 50				
TOX	0.25	< 50				

ED50 Biological Response

Test	Dose (mg/kg)	Dths	N / F	C:
MES	3.5		0 / 8	
MES	5.3		2 / 8	
MES	7		6 / 8	
MES	14		6 / 8	
MES	21		8 / 8	
SCMET	50		0 / 3	

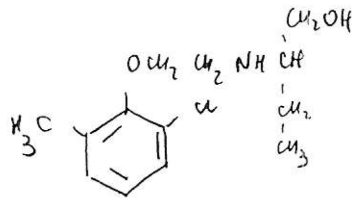

(R,S)

Time to Peak Effect

Test	Dose (mg/kg)	# Dths	0.25 N / F	C	0.5 N / F	C	1.0 N / F	C	2.0 N / F	C	4.0 N / F	C	6.0 N / F	C	8.0 N / F	C	24 N / F	C	3.0 N / F	C
MES	12		4 / 4		3 / 4		/		/		/		/		/		/		/	
TOX	50	1	4 / 4	1	/		/		/		/		/		/		/		/	
TOX	100	4	4 / 4	1	/		/		/		/		/		/		/		/	

Response Comments

TEST	DOSE (mg/kg)	TIME	CODE	COMMENT
TOX	50	0.25	1	Death
TOX	100	0.25	1	Death

Anticonvulsant Screening Project

Test 10 Results -Anticonvulsant Quantification (Rats I.P.)

Add ID: 348014 U   Screen ID: 1

omments to Supplier:

KM319

Anticonvulsant Screening Project

Test 10 Results -Anticonvulsant Quantification (Rats I.P.)

Add ID: 348014 A **Screen ID:** 2

Solvent Code: MC **Solvent Prep:** M&P,SB **Route Code:** IP

Animal Weight: to g

Date Started: 07-Jun-2004 **Date Completed** 10-Aug-2004

Reference: 379:200-237;381:74

ED50 Value

Test	Time (Hrs)	ED50	95% Confidence Interval LOW	HIGH	SLOPE	STD. ERR.
TOX	0.25	32.85	29.25	35.51	20.81	7.16

ED50 Biological Response

Test	Dose (mg/kg)	Dths	N / F	C:
TOX	20		0 / 8	
TOX	30		2 / 8	
TOX	35		5 / 8	
TOX	40		8 / 8	

Time to Peak Effect

Test	Dose (mg/kg)	# Dths	0.25		0.5		1.0		2.0		4.0		6.0		8.0		24		3.0	
			N / F	C	N / F	C	N / F	C	N / F	C	N / F	C	N / F	C	N / F	C	N / F	C	N / F	C
TOX	30		2 / 8		0 / 8		0 / 8		0 / 8		0 / 8		0 / 8		/		0 / 8		/	
TOX	40		8 / 8		3 / 8		0 / 8		0 / 8		/		/		/		/		/	

Comments to Supplier:

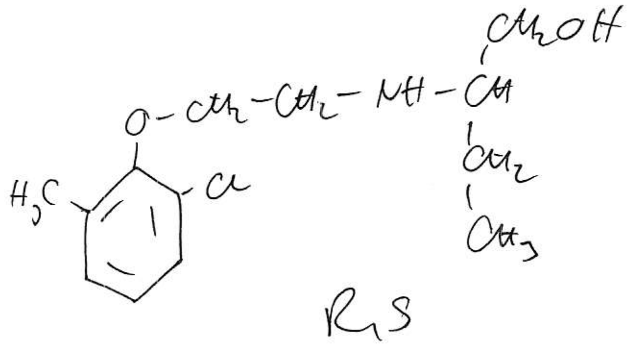

R,S

UM-314

Anticonvulsant Screening Project

Test 3 Results - Rat P.O. Quantification

dd ID: 348014 A **Screen ID:** 1

olvent Code: MC **Solvent Prep:** M&P,SB

nimal Weight: to g

ate Started: 16-Aug-2004 **Date Completed** 16-Aug-2004

eference: 381:78-81

D50 Value

Test	Time (Hrs)	ED50	95% Confidence Interval LOW	HIGH	SLOPE	STD. ERR.	PI VALUE
IES	0.5	> 60	0	0			
TOX	0	> 60	0	0			

me to Peak Effect

Test	Dose (mg/kg)	# Dths	0.25 N / F	C	0.5 N / F	C	1.0 N / F	C	2.0 N / F	C	4.0 N / F	C	6.0 N / F	C	8.0 N / F	C	24 N / F	C	3.0 N / F	C
MES	30		0 / 4		1 / 4		1 / 4		0 / 4		1 / 4		/		/		/		/	
MES	60		0 / 4		0 / 4		0 / 4		/		/		/		/		/		/	
TOX	60		0 / 4		0 / 4		0 / 4		/		/		/		/		/		/	

omments to Supplier:

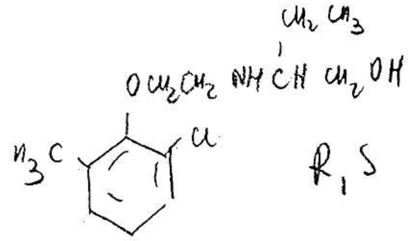

8

onday, August 30, 2004

Anticonvulsant Screening Project

Test 4 Results - Mice I.P. Quantification

ıd ID: 348014 B **Screen ID:** 1

lvent Code: MC **Solvent Prep:** M&P,SB

imal Weight: to g

KM-314

te Started: 04-Feb-2005 **Date Completed:** 11-Feb-2005

ference: 390-7-18

50 Values

est	Time (Hrs)	ED50	95% Confidence Interval LOW	HIGH	SLOPE	STD. ERR.	PI VALUE
ES	0.25	21.44	18.63	25.32	9.93	3.03	
MET	0.25	> 62	0	0			
OX	0.25	54.99	52.95	56.94	55	17.38	

50 Biological Response

ʼest	Dose (mg/kg)	Dths	N / F	C
IES	15		0 / 8	
IES	19		3 / 8	
IES	22		5 / 8	
IES	30		7 / 8	
MET	37		0 / 8	3
MET	62		0 / 8	3
OX	50		0 / 8	
OX	54		3 / 8	*
OX	58		7 / 8	14
OX	62		8 / 8	*

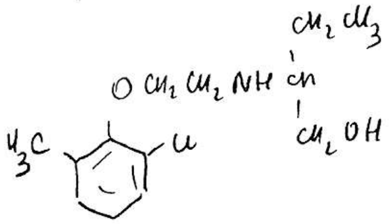

50 Biological Response Comments

EST	DOSE (mg/kg)	TIME	CODE	COMMENT
MET	37	0.25	3	Death following continuous seizure
MET	62	0.25	3	Death following continuous seizure
OX	54	0.25	34	Muscle spasms
OX	54	0.25	14	Unable to grasp rotorod
OX	58	0.25	14	Unable to grasp rotorod

Anticonvulsant Screening Project

KM-314

Test 4 Results - Mice I.P. Quantification

[illegible] 2004, 58, 9-10
ISSN 0043-5104

d ID: 348014-B   Screen ID: 1

OX	62	0.25	34	Muscle spasms
OX	62	0.25	14	Unable to grasp rotorod

e to Peak Effect

est	Dose (mg/kg)	# Dths	0.25		0.5		1.0		2.0		4.0		6.0		8.0		24		3.0	
			N / F	C	N / F	C	N / F	C	N / F	C	N / F	C	N / F	C	N / F	C	N / F	C	N / F	C
ES	30		3 / 4		2 / 4		0 / 4		0 / 4		/		/		/		/		/	
OX	54		3 / 8	14	0 / 8		0 / 8		0 / 8		/		/		/		/		/	

ponse Comments

ST	DOSE (mg/kg)	TIME	CODE	COMMENT
X	54	0.25	14	Unable to grasp rotorod

mments to Supplier:

KM-314

Anticonvulsant Screening Project

Test 3 Results - Rat P.O. Quantification

Add ID: 348014 B **Screen ID:** 2

Solvent Code: MC Solvent Prep: M&P,SB

Animal Weight: to g

Date Started: 30-Mar-2005 Date Completed: 08-Apr-2005

Reference: 386:110-123

ED50 Values

Test	Time (Hrs)	ED50	95% Confidence Interval LOW	HIGH	SLOPE	STD. ERR.	PI VALUE
MES	0.25	67.69	43.01	93.82	3.87	1.1	
SCMET	0	> 240	0	0			
TOX	0.25	> 240	0	0			

ED50 Biological Response

Test	Dose (mg/kg)	Dths	N / F	C
MES	30		1 / 8	
MES	60		3 / 8	
MES	120		6 / 8	
MES	150		8 / 8	
SCMET	240		0 / 4	3
TOX	240		4 / 8	*

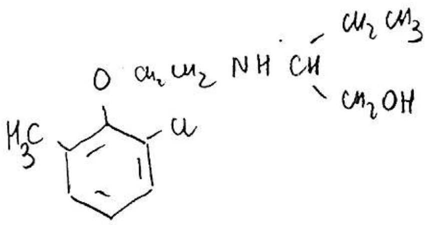

R,S

ED50 Biological Response Comments

TEST	DOSE (mg/kg	TIME	CODE	COMMENT
SCMET	240	0.25	3	Death following continuous seizure
TOX	240	0.25	3	Death following continuous seizure
TOX	240	0.25	19	Sedated

Time to Peak Effect

Test	Dose (mg/kg)	# Dths	0.25 N / F	C	0.5 N / F	C	1.0 N / F	C	2.0 N / F	C	4.0 N / F	C	6.0 N / F	C	8.0 N / F	C	24 N / F	C	3.0 N / F	C
MES	90		2 / 4		0 / 4		1 / 4		/		/		/		/		/		/	
MES	120		3 / 4		1 / 4		3 / 4		1 / 4		0 / 4		/		/		/		/	
SCMET	50		0 / 4		0 / 4		0 / 4		0 / 4		0 / 4		/		/		/		/	

KM-314

Anticonvulsant Screening Project

Test 3 Results - Rat P.O. Quantification

Add ID: 348014 B	Screen ID: 2											
SCMET	240		0 / 4 3	/	/	/	/	/	/	/	/	
TOX	200		0 / 4	0 / 4	0 / 4	0 / 4	0 / 4	/	/	/	/	
TOX	240		3 / 4	0 / 4	0 / 4	0 / 4	0 / 4	/	/	/	/	

Response Comments

TEST	DOSE (mg/kg	TIME	CODE	COMMENT
SCMET	240	0.25	3	Death following continuous seizure

Comments to Supplier:

Anticonvulsant Screening Project

KM-314

Test 15 Results - IV Metrazol

Add ID: 348014 C Screen ID: 1

Solvent Code: MC Solvent Prep: M&P,SB Route Code: I.P.

Time of Test: 0.25 (hrs Infusion Rate: 0.34 (ml/min)

MES ED50: 21.44 (mg/kg) TD50: 55 (mg/kg)

Date Started: 04-Aug-2005 Date Completed: 04-Aug-2005

Reference: 386: 148-149

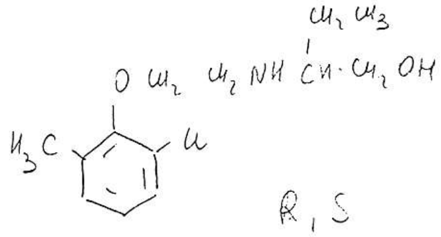

ANALYSIS

Dose (mg/kg)		Weight (grams)	Time to Twitch	Twitch (mg/kg)	Time to Clonus	Clonus (mg/kg)
0	Mean	27.70	25.8	26.4	34.2	34.9
	Std.Err	0.50	1.37	1.26	3.37	3.26
	P-Value					
21.44	Mean	27.20	26.1	27.1	40.0	41.6
	Std.Err	0.28	1.76	1.64	2.17	2.22
	P-Value	0.199	0.447	0.364	0.086	0.053
55	Mean	26.90	34.8	36.7	51.1	53.7
	Std.Err	0.48	3.04	3.15	3.49	3.39
	P-Value	0.132	0.009	0.005	0.001	0.000

Response

Dose (mg/kg)	Animal #	Weight (grams)	Time to Twitch	Twitch (mg/kg)	Time to Clonus	Clonus (mg/kg)
0	01	24.5	24.00	27.75	30.00	34.69
0	02	28.5	26.00	25.85	46.50	46.23
0	03	28.0	33.50	33.90	56.50	57.17
0	04	29.0	27.00	26.38	36.00	35.17
0	05	28.0	26.00	26.31	29.50	29.85
0	06	28.5	28.00	27.84	37.50	37.28
0	07	29.5	30.50	29.29	34.00	32.66
0	08	25.5	23.00	25.56	25.50	28.33
0	09	27.0	19.00	19.94	22.00	23.09
0	10	28.5	21.00	20.88	24.50	24.36
Avg.		27.7	25.80	26.37	34.20	34.88
Std. Err.		0.50	1.37	1.26	3.37	3.26

KM-314

Anticonvulsant Screening Project

Test 7 Results - Anticonvulsant Evaluation (6Hz, Mice)

Add ID: 348014 C **Screen ID:** 1

Solvent Code: MC Solvent Prep: M&P,SB Route Code: IP Current (mA): 32

Animal Weight: to g

Date Started: 29-Aug-2005 Date Completed: 29-Aug-2005

Reference: 396:78

Time to Peak Effect

Test	Dose (mg/kg)	# Dths	0.25 N / F	0.25 C	0.5 N / F	0.5 C	1.0 N / F	1.0 C	2.0 N / F	2.0 C	4.0 N / F	4.0 C	6.0 N / F	6.0 C	8.0 N / F	8.0 C	24 N / F	24 C	3.0 N / F	3.0 C
6HZ	30		0 / 4	15	3 / 4	15	1 / 4	15	0 / 4		0 / 4	15	/		/		/		/	

Response Comments

TEST	DOSE (mg/kg	TIME	CODE	COMMENT
6HZ	100	4	15	Minimal motor impairment
6HZ	100	1	15	Minimal motor impairment
6HZ	100	0.5	15	Minimal motor impairment
6HZ	100	0.25	15	Minimal motor impairment

Comments to Supplier:

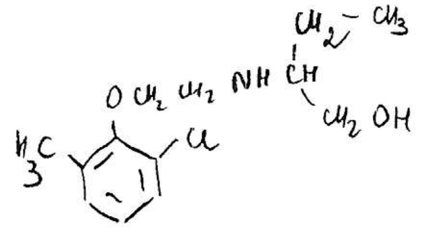

R,S

KN-314

Anticonvulsant Screening Project

Test 11 Results - Toxicity Screen (Rats I.P.)

Add ID: 348014 C   Screen ID: 1

Solvent Code: MC   Solvent Prep: M&P,SB   Route Code: IP

Animal Weight: to g

Date Started: 22-Sep-2005   Date Completed: 20-Dec-2005

Reference: 394:243

Time to Peak Effect

Test	Dose (mg/kg)	# Dths	0.25		0.5		1.0		2.0		4.0		6.0		8.0		24		3.0	
			N / F	C	N / F	C	N / F	C	N / F	C	N / F	C	N / F	C	N / F	C	N / F	C	N / F	C
TOX	3		0 / 2		0 / 2		0 / 2		0 / 2		0 / 2		/		/		/		/	
TOX	10		0 / 2		0 / 2		0 / 2		0 / 2		0 / 2		/		/		/		/	
TOX	30		0 / 2		0 / 2		0 / 2		0 / 2		0 / 2		/		/		/		/	
TOX	40		1 / 2		0 / 2		0 / 2		0 / 2		0 / 2		/		/		/		/	

Comments to Supplier:

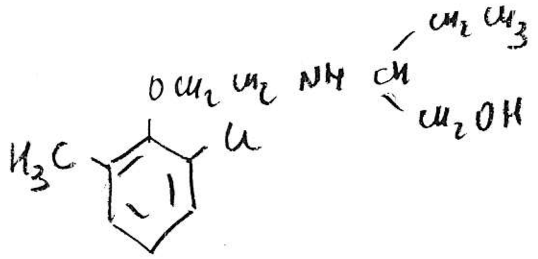

KM-314

Anticonvulsant Screening Project

Test 11 Results - Preliminary Hippocampal Kindling Screen - Rats IP

Add ID: 348014 C **Screen ID:** 1

Solvent Code: MC Solvent Prep: M&P,SB Route Code: IP

Animal Weight: to g

Date Started: 22-Dec-2005 Date Completed: 22-Dec-2005

Reference: 399:131-134

Dose: 40 mg/kg Time of Maximum Effect: 15 to 135 min

Rat #	Comment Code	Seizure Score				Afterdischarge Duration (secs)			
		Pre-Drug		Drug		Pre-Drug		Drug	
		Low	High	Low	High	Low	High	Low	High
1		5 -		5 -		28 -	42	41 -	43
		5 -		5 -		67 -	81	66 -	103

Comments to Supplier: No apparent activity against seizure score or afterdischarge duration.

KM-314

Anticonvulsant Screening Project

Test 6 Results - BIC, PIC (Mice I.P.)

Add ID: 348014 C **Screen ID:** 1

Solvent Code: MC Solvent Prep: M&P,SB

Animal Weight: to g

Date Started: 31-Jan-2006 Date Completed: 31-Jan-2006

Reference: 395:249-250

ED50 Values

Test	Time (Hrs)	ED50	95% Confidence Interval LOW	HIGH	SLOPE	STD. ERR.	PI VALUE
BIC	0.25	> 70	0	0			
PIC	0.25	> 70	0	0			

ED50 Biological Response

Test	Dose (mg/kg)	Dths	N / F	C
BIC	12.5		0 / 3	
BIC	25		0 / 3	
BIC	50		1 / 3	
BIC	70		1 / 8	*
PIC	12.5		0 / 3	
PIC	25		0 / 3	
PIC	50		0 / 3	15
PIC	70		3 / 8	*

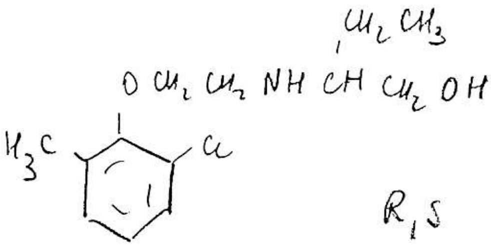

R,S

ED50 Biological Response Comments

TEST	DOSE (mg/kg	TIME	CODE	COMMENT
BIC	70	0.25	34	Muscle spasms
BIC	70	0.25	25	Myoclonic jerks
BIC	70	0.25	15	Minimal motor impairment
PIC	50	0.25	15	Minimal motor impairment
PIC	70	0.25	34	Muscle spasms
PIC	70	0.25	25	Myoclonic jerks
PIC	70	0.25	15	Minimal motor impairment

Anticonvulsant Screening Project

Test 6 Results - BIC, PIC (Mice I.P.)

Add ID: 348014 C Screen ID: 1

Comments to Supplier:

KM-314

Anticonvulsant Screening Project

Test 21 Results - Ames Mutagenicity Assay (Preliminary) - Part A

Add ID: 348014 C **Screen ID:** 1

Solvent Code: DMSO **Solvent Prep:**

Date Started: 25-Jul-2006 **Date Completed:** 27-Jul-2006

Reference: G5:248

S9 Source:

Chemical Addition		% of benzo[a]pyrene(+NADPH) control (+) NADPH		(-) NADPH	
		S1	S2	S1	S2
None	0.00 mM	6.2	7.3		
DMSO	0.04 mM	5.8	6.3		
ADD	0.10 mM	7.8	7.8	5.5	7.4
ADD	0.20 mM	5.2	7.4	5.4	5.3
ADD	0.50 mM	5.2	7	4.9	6.7
Benzo[a]pyrene	0.02 mM	100	100	6.5	6
Acridine orange	0.02 mM	117	128	17.9	21.1

Comments to Supplier:

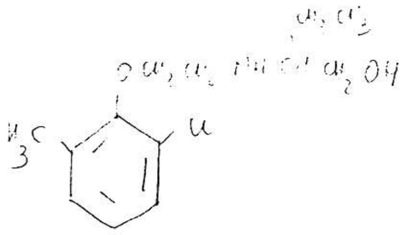

KM 314

Anticonvulsant Screening Program

Test 22 Results - Formalin Test (Mice I.P.)

Add ID: 348014 C   **Screen ID:** 1

Solvent Code: MC   Solvent Prep: M&P,SB

Time of Test: 0.25 (hrs)   Route Code: IP

ED50: 21.44 (mg/kg)   TD50: 55 (mg/kg)

Date Started: 22-Sep-2006   Date Completed: 27-Oct-2006

Reference: F3: 1

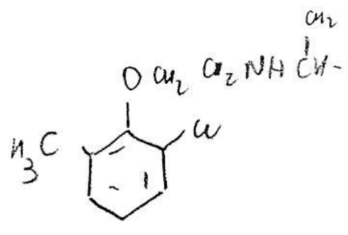

R,S

Analysis

Dose (mg/kg)	Test	Area Under the Curve: Control	Drug Treated	% of control	S.E.M	p Value
21	Acute	194.9	124.8	64.0	6.3	< 0.01
21	Inflammatory	680.9	569.2	72.7	24.1	> 0.05

Reponse

Trial 1 — Duration of Licking (sec)

Dose (mg/kg)	Animal #	0 min	5 min	10 min	15 min	20 min	25 min	30 min	35 min	40 min	45 min	50 min	55 min
0	01	58.12	0.00	0.00	14.80	39.20	15.03	21.74	3.66	1.60			
0	02	73.98	0.00	0.00	15.34	2.34	45.76	16.62	21.56	0.00			
0	03	65.34	16.86	0.00	2.32	77.06	0.00	40.17	10.85	0.00			
0	04	42.42	11.07	0.00	0.00	27.31	42.15	66.08	5.18	0.00			
0	05	68.59	5.74	0.00	2.80	32.00	50.78	72.89	65.98	31.57			
0	06	51.48	8.32	9.55	9.38	41.90	23.34	43.37	29.11	15.14			
0	07	76.65	0.00	0.00	32.05	55.65	1.12	54.34	9.81	0.00			
0	08	73.70	10.03	0.00	1.46	19.60	76.59	14.94	0.00	0.00			

Trial 1 — Duration of Licking (sec)

Dose (mg/kg)	Animal #	0 min	5 min	10 min	15 min	20 min	25 min	30 min	35 min	40 min	45 min	50 min	55 min
21	01	45.68	0.00	0.00	0.00	13.47	0.00	70.06	23.24	5.49			
21	02	57.37	0.00	20.66	0.00	6.28	47.96	37.09	30.45	6.77			
21	03	52.44	0.00	0.00	15.86	0.00	68.02	21.56	0.00	0.00			
21	04	43.44	0.00	0.00	0.00	0.00	0.00	0.00	0.00	0.00			
21	05	40.32	0.00	0.00	0.00	0.00	100.87	78.50	76.92	41.25			
21	06	53.03	0.00	0.00	0.00	0.00	0.00	85.75	70.96	0.00			
21	07	30.58	0.00	0.00	24.66	0.00	0.00	0.00	0.00	0.00			
21	08	25.04	6.14	18.56	63.11	12.77	79.33	3.71	5.13	0.00			

Anticonvulsant Screening Program

Test 22 Results - Formalin Test (Mice I.P.)

Add ID: 348014 C **Screen ID:** 1

Comments to Supplier:

KM 314

Anticonvulsant Screening Program

Test 25 Results - LTG-Resistant Amygdala Kindled Rat

\dd ID: 348014 C Screen ID: 1

Solvent Code: MC Solvent Prep: M&P,SB Route Code: IP

Date Started: 03-Oct-2006 Date Completed: 04-Oct-2006

Reference: AS2:133-136

Results

Dose (mg/kg)	Time of Test (hrs)	Seizure Score ± S.E.M.		Duration (sec) ± S.D		# Prot / # Tested
Control	0	5.0 ± 0.0	☐	52 ± 22	☐	0 / 6
15	0.25	0.7 ± 0.7	☑	3 ± 7	☑	5 / 6

Note: Box checked if data is significantly different from control.

Comments to Supplier:

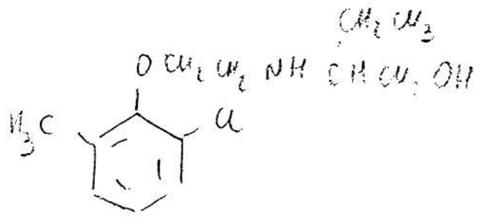

R,S

KM-314

Anticonvulsant Screening Project

Test 3 Results - Rat P.O. Quantification

Add ID: 367061 U **Screen ID:** 1

Solvent Code: MC **Solvent Prep:** TT

Animal Weight: to g

Date Started: 24-Aug-2007 **Date Completed:** 28-Aug-2007

Reference: 409:195,198

Time to Peak Effect

Test	Dose (mg/kg)	# Dths	0.25	0.5	1.0	2.0	4.0	6.0	8.0	24	3.0
			N / F C	N / F C	N / F C	N / F C	N / F C	N / F C	N / F C	N / F C	N / F C
MES	30		0 / 4	0 / 4	0 / 4	1 / 4	0 / 4	/	/	/	/
MES	60		1 / 4	0 / 4	0 / 4	0 / 4	0 / 4	/	/	/	/
TOX	30		0 / 4	0 / 4	0 / 4	0 / 4	0 / 4	/	/	/	/
TOX	60		0 / 4	0 / 4	0 / 4	0 / 4	0 / 4	/	/	/	/

Comments to Supplier:

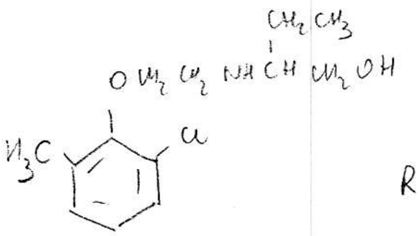

R,S

KM-314

Anticonvulsant Screening Project

Test 16 Results - Anticonvulsant Quantification (Frings Mice)

Add ID: 348014 D **Screen ID:** 1

Solvent Code: MC **Solvent Prep:** M&P,SB **Route Code:** IP

Date Started: 20-Sep-2007 **Date Completed:** 20-Sep-2007

Reference: 414:140-141

ED50 Values

Test	Time (Hrs)	ED50	95% Confidence Interval LOW	95% Confidence Interval HIGH	SLOPE	STD. ERR.
FRINGS	0.25	6.05	4.23	8.45	5.73	1.8

[illegible]50 Biological Response

Test	Dose (mg/kg)	Dths	N / F	C
FRINGS	2.5		0 / 8	
FRINGS	5		3 / 8	
FRINGS	10		7 / 8	
FRINGS	20		8 / 8	

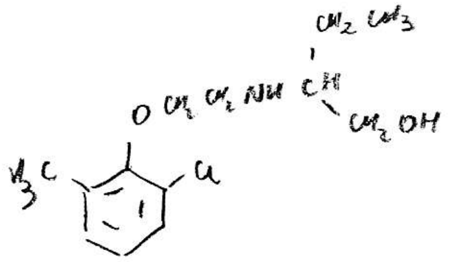

R,S

Comments to Supplier:

KM-314

Anticonvulsant Screening Program

Test 71 Results - Pilocarpine-induced Status, Rats - Time 0 Min

Add ID: 348014 D **Screen ID:** 1

Solvent Code: MC **Solvent Prep:** M&P,SB **Route Code:** IP

Date Started: 11-Nov-2007 **Date Completed:** 13-Nov-2007

Reference: CM1:51-52

Response Data

Dose (mg/kg)	Time (hrs) [a]	N / F	C	Dths	Avg. Weight Change(g) +/- S.E.M [b]			
					Protected Rats		Non-Protected Rats	
40.00	0.0	0 / 7		4			–	20.0 +/- 0.0

[a] Post first Stage III seizure
[b] Weight change 24 hours Post first Stage III seizure

Comments to Supplier: Dose calculated from Test 10 results; TD97 is 40.53 mg/kg

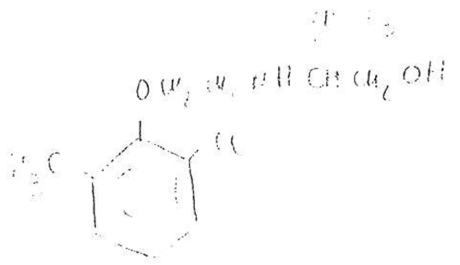

R,S

KM-314

Anticonvulsant Screening Program
Test 17 Results - Electrophysiology Studies

Add ID: 348014 D Screen ID: 3

**Solvent Code:**	DMSO	**Solvent Prep:**		**Molecular Weight:**	257.759
**Date Started:**	27-Feb-2009	**Date Completed:**	27-Feb-2009		

Reference: EP8:144

Response

Test	Add Compound Conc.(uM)	# Cells	% Control +/- SEM		Holding Potential (mV)
SODIUM	100	7	55 +/- 5	☑	-90
SODIUM	100	7	26 +/- 3	☑	-60

Note: box is checked if data is significant.

Comments to Supplier:

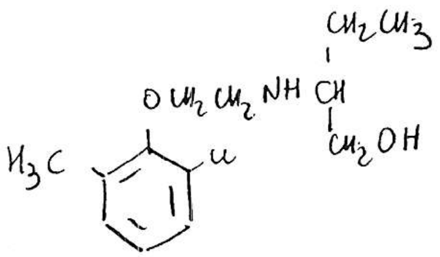

R,S

UM-314

Anticonvulsant Screening Program

Test 17 Results - Electrophysiology Studies

Add ID: 348014 D Screen ID: 2

Solvent Code: DMSO **Solvent Prep:** **Molecular Weight:** 257.759

Date Started: 16-Mar-2009 **Date Completed:** 27-Mar-2009

Reference: EP8:147-150

Response

Test	Add Compound Conc.(uM)	# Cells	% Control +/- SEM	Holding Potential (mV)
KAINATE	100	7	91 +/- 3 ☑	-70

Note: box is checked if data is significant.

Comments to Supplier:

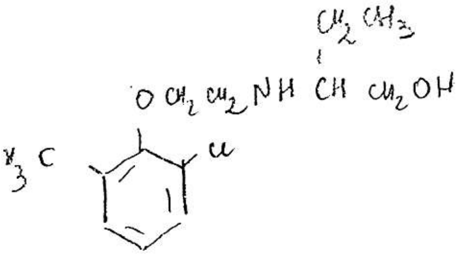

R,S

Anticonvulsant Screening Program

KM-314

Test 17 Results - Electrophysiology Studies

Add ID: 348014 D Screen ID: 1

Solvent Code: DMSO Solvent Prep: Molecular Weight: 257.759

Date Started: 16-Mar-2009 Date Completed: 27-Mar-2009

Reference: EP8:147-150

Response

Test	Add Compound Conc.(uM)	# Cells	% Control +/- SEM	Holding Potential (mV)
GABA	100	8	96 +/- 3 ☐	-70

Note: box is checked if data is significant.

Comments to Supplier:

KM-314

Anticonvulsant Screening Program
Test 17 Results - Electrophysiology Studies

Add ID: 348014 D Screen ID: 4

Solvent Code: DMSO Solvent Prep: Molecular Weight: 257.759

Date Started: 16-Mar-2009 Date Completed: 27-Mar-2009

Reference: EP8:147-150

Response

Test	Add Compound Conc.(uM)	# Cells	% Control +/- SEM		Holding Potential (mV)
NMDA [10uM] + Glycine [1 uM]	100	8	63 +/- 5	☑	-70

Note: box is checked if data is significant.

Comments to Supplier:

Anticonvulsant Screening Program
Test 28 - In Vitro Metabolic Gene Induction Studies

Add ID: 348014 D Screen ID: 2

Solvent Code:	DMSO	Solvent Prep:		Unit:	uM
Cell line source:	Puracyp			Molecular Weight:	257.7590
Date Started:	05-Feb-2010	Date Completed:	15-Feb-2010		
Reference:	Lamb book#8 pg. 77				

Response

Isoform Code: CYP3A1 Cell line: RPXR

Induction mechanism: pregnane X receptor

Concentration	Induction		Cell	
	Fold +/- S.D		Viability +/- S.D	
0	1 +/- 0.24	☐	100 +/- 9.60	☐
10	1.10 +/- 0.11	☐	95 +/- 6.50	☐
25	1 +/- 0.08	☐	90 +/- 8.30	☐
50	1.11 +/- 0.11	☐	99 +/- 8.60	☐
100	1.35 +/- 0.20	☑	91 +/- 6.10	☐
200	2.35 +/- 0.27	☑	67 +/- 4.80	☑
500	2.22 +/- 0.15	☑	43 +/- 5.40	☑

Note: Box checked if data is significantly different.

EC50 (calculated): 74

Max induction (calculated): 2 fold @ 200uM
significant decrease in viability at this concentration in vitro.

Comments to Supplier: Drugs shows induction of rat CYP3A1, via the rat pregnane X receptor, in vitro. A two fold induction was seen at 200uM.

KM-314

Anticonvulsant Screening Program

Test 28 - In Vitro Metabolic Gene Induction Studies

Add ID: 348014 D Screen ID: 3

Solvent Code: DMSO **Solvent Prep:** **Unit:** uM

Cell line source: Puracyp **Molecular Weight:** 257.7590

Date Started: 05-Feb-2010 **Date Completed:** 15-Feb-2010

Reference: Lamb book#8 pg. 77

Response

Isoform Code: P-glycoprotein **Cell line:** MDR1.C

Induction mechanism: pregnane X receptor

Concentration	Induction		Cell	
	Fold +/- S.D		Viability +/- S.D	
0	1 +/- 0.07	☐	100 +/- 6.90	☐
10	1.09 +/- 0.10	☐	94 +/- 4.30	☐
25	1.35 +/- 0.08	☑	103 +/- 3.90	☑
50	1.32 +/- 0.08	☑	87 +/- 5	☐
100	1.69 +/- 0.15	☑	94 +/- 5.90	☐
200	1.66 +/- 0.11	☑	85 +/- 2.30	☑
500	1.69 +/- 0.13	☑	87 +/- 2.10	☑

Note: Box checked if data is significantly different.

EC50 (calculated):

Max induction (calculated): slight induction (under two fold) in vitro.

Comments to Supplier: Drugs shows slight induction of human P-glycoprotein, via the pregnane X receptor, in vitro. The highest response was less than that seen with 10uM rifampicin.

VM-314

Anticonvulsant Screening Program

Test 28 - In Vitro Metabolic Gene Induction Studies

Add ID: 348014 D **Screen ID:** 1

Solvent Code:	DMSO	Solvent Prep:		Unit:	uM
Cell line source:	Puracyp			Molecular Weight:	257.7590
Date Started:	05-Feb-2010	Date Completed:	15-Feb-2010		
Reference:	Lamb book#8 pg. 77				

Response

Isoform Code: CYP3A4 **Cell line:** DPX2

Induction mechanism: pregnane X receptor

Concentration	Induction		Cell	
	Fold +/- S.D		Viability +/- S.D	
0	1 +/- 0.25	☐	100 +/- 4	☐
10	1.18 +/- 0.19	☐	98 +/- 2.30	☐
25	1.33 +/- 0.26	☑	82 +/- 14.10	☑
50	2.50 +/- 0.27	☑	78 +/- 4	☑
100	5.59 +/- 0.63	☑	56 +/- 7.20	☑
200	12 +/- 1.20	☑	60 +/- 3	☑
500	12.60 +/- 1.80	☑	40 +/- 6.40	☑

Note: Box checked if data is significantly different.

EC50 (calculated): 100

Max induction (calculated): 12 fold @ 200uM

significant decrease in viability at 50uM and above, in vitro.

Comments to Supplier: Drug shows induction of human CYP3A4, via the pregnane X receptor, in vitro. Moderate potential for drug-drug interations (DDI) if liver cells are exposed to 50uM, high potential for DDI if liver cells are exposed to 50-100uM.

KM-314

Anticonvulsant Screening Program

Test 28 - In Vitro Metabolic Gene Induction Studies

Add ID: 348014 D Screen ID: 4

**Solvent Code:**	DMSO	**Solvent Prep:**		**Unit:**	uM
**Cell line source:**	Puracyp			**Molecular Weight:**	257.7590
**Date Started:**	05-Feb-2010	**Date Completed:**	15-Feb-2010		
**Reference:**	Lamb book#8 pg.77				

Response

Isoform Code: CYP1A2 **Cell line:** 1A2DRE

Induction mechanism: aryl hydrocarbon receptor

Concentration	Induction		Cell	
	Fold +/- S.D		Viability +/- S.D	
0	1 +/- 0.05	☐	100 +/- 2.20	☐
10	0.95 +/- 0.06	☐	106 +/- 7.10	☐
25	1.12 +/- 0.19	☐	96 +/- 9.90	☐
50	1.39 +/- 0.17	☑	80 +/- 6.60	☑
100	1.67 +/- 0.16	☑	77 +/- 6.20	☑
200	1.92 +/- 0.13	☑	71 +/- 5.40	☑
500	2.78 +/- 0.16	☑	49 +/- 13.70	☑

Note: Box checked if data is significantly different.

EC50 (calculated): 37

Max induction (calculated): 2 fold @ 200uM

significant decrease in viability at this concentration in vitro.

Comments to Supplier: Drugs shows slight induction of human CYP1A2, via the aryl hydrocarbon receptor, in vitro. A two fold induction was seen at 200uM, this response is similar that seen with 25uM omeprazole.

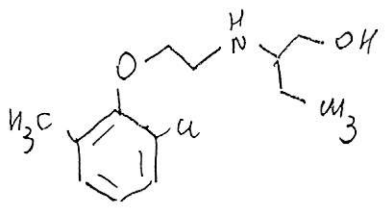

R, S

KM 314

Anticonvulsant Screening Program

Test 19 Results - Human Cytochrome P450 Inhibition Studies

Add ID: 348014 D **Screen ID:** 1

Solvent Code: MEOH **Solvent Prep:**

Date Started: 18-Feb-2010 **Date Completed:** 19-Feb-2010

Isoform Code: CYP1A2 **Enzyme Source:** celsis F008085-CST

Reaction: Methoxyresorufin O-demethylation

ADD Cmpd Conc. (uM)	Activity @ [S]=2.0uM	Activity @ [S]=0.25uM	Activity @	Activity @ [S]=2.0uM	Activity @ [S]=1.0uM	Activity @ [S]=0.5uM	Activity @ [S]=0.25uM	Activity @	Activity @	Activity @
0	100.5 (0)	46.3 (0)		100 (0)	89.8 (0)	75.5 (0)	50.9 (0)			
500	34.9 (65)	5.45 (88)								
6.25				97.7 (2)	91.6 (-2)	71.2 (6)	45.2 (11)			
25.0				84.4 (16)	73 (19)	58.2 (23)	33.4 (34)			
100.0				65 (35)	48.6 (46)	31.2 (59)	16.6 (67)			
500.0				27.6 (72)	18.1 (80)	9.9 (87)	5.8 (89)			
IC50		< 500.0		Approx 250.0			Approx 60.0			

Note: Activities are reported in protein/min in bold print. In parentheses below each activity is (%) inhibition relative to the activity determined at the same substrate concentration [S] in the absence of ADD Cmpd (i.e. 0 uM). When calculated, an ADD Cmpd IC50 value (uM) is also shown in bold print in the bottom row for each substrate concentration.

Ki(uM): 23.0 **Inhibition (Type):** mixed/competitive

Comments to Supplier: ADD 348014 is an inhibitor of CYP1A2 in vitro and could inhibit the metabolism of other drugs metabolized by CYP1A2 in vivo.

6/3/2010 1:22:31 PM

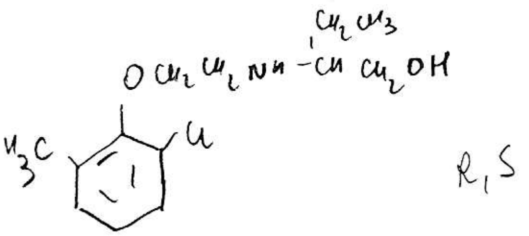

KM 314

Anticonvulsant Screening Program

Test 19 Results - Human Cytochrome P450 Inhibition Studies

Add ID: 348014 D Screen ID: 8 [illegible]

Solvent Code: MEOH **Solvent Prep:**

Date Started: 20-Feb-2010 **Date Completed:** 20-Feb-2010

Isoform Code: CYP3A4 **Enzyme Source:** HBI # 501

Reaction: Testosterone 6beta-hydroxylation

ADD Cmpd Conc. (uM)	Activity @ [S]=200.0uM	Activity @ [S]=50.0uM	Activity @	Activity @	Activity @	Activity @	Activity @	Activity @	Activity @	Activity @
0	**4,701.2** (0)	**2,544.7** (0)								
500	**2,268.4** (52)	**1,190** (53)								
IC50		**Approx 500.0**								

Note: Activities are reported in protein/min in bold print. In parentheses below each activity is (%) inhibition relative to the activity determined at the same substrate concentration [S] in the absence of ADD Cmpd (i.e. 0 uM). When calculated, an ADD Cmpd IC50 value (uM) is also shown in bold print in the bottom row for each substrate concentration.

Ki(uM): **Inhibition (Type):**

omments to Supplier: **ADD 348014 is a weak inhibitor of CYP3A4 in vitro and high concentrations in vivo could cause minor inhibition of other drugs metabolized by CYP3A4 although the possibility appears remote.**

/3/2010 1:22:23 PM

KM 314

Anticonvulsant Screening Program

Test 19 Results - Human Cytochrome P450 Inhibition Studies

Add ID: 348014 D Screen ID: 3

Solvent Code: MEOH **Solvent Prep:**

Date Started: 22-Feb-2010 **Date Completed:** 03-Mar-2010

Isoform Code: CYP2B6 **Enzyme Source:** celsis M008085-JTA

Reaction: 7-ethoxy-4-trifluoromethylcoumarin O-deethylation

ADD Cmpd Conc. (uM)	Activity @ [S]=25.0uM	Activity @ [S]=6.25uM	Activity @	Activity @ [S]=6.25uM	Activity @	Activity @	Activity @	Activity @	Activity @	Activity @
0	**200.2** (0)	**130.3** (0)		**139.8** (0)						
500	**62.7** (69)	**28.7** (78)								
25.0				**126.5** (10)						
50.0				**92.7** (34)						
100.0				**62.7** (55)						
250.0				**45.8** (67)						
500.0				**31** (78)						
IC50		**< 500.0**		**Approx 100.0**						

Note: Activities are reported in protein/min in bold print. In parentheses below each activity is (%) inhibition relative to the activity determined at the same substrate concentration [S] in the absence of ADD Cmpd (i.e. 0 uM). When calculated, an ADD Cmpd IC50 value (uM) is also shown in bold print in the bottom row for each substrate concentration.

Ki(uM): **Inhibition (Type):**

Comments to Supplier: **ADD 348014 is a weak inhibitor of CYP2B6 in vitro and high concentrations in vivo could cause minor inhibition of other drugs metabolized by CYP2B6 although the possibility appears remote.**

6/3/2010 1:22:29 PM

VM314

Anticonvulsant Screening Program

Test 19 Results - Human Cytochrome P450 Inhibition Studies

Add ID: 348014 D Screen ID: 2

Solvent Code: MEOH Solvent Prep:

Date Started: 22-Feb-2010 Date Completed: 23-Feb-2010

Isoform Code: CYP2A6 Enzyme Source: celsis X008064-LSL

Reaction: Coumarin 7-hydroxylation

ADD Cmpd Conc. (uM)	Activity @ [S]=10.0uM	Activity @ [S]=1.25uM	Activity @	Activity @ [S]=10.0uM	Activity @ [S]=5.0uM	Activity @ [S]=2.5uM	Activity @ [S]=1.25uM	Activity @	Activity @	Activity @
0	**192.3** (0)	**74.6** (0)		**256.6** (0)	**259.3** (0)	**177** (0)	**137.3** (0)			
500	**107.3** (44)	**23.3** (69)								
25.0				**254.3** (1)	**246.8** (5)	**168** (5)	**122** (11)			
50.0				**231.4** (10)	**228.3** (12)	**165.2** (7)	**121.9** (11)			
100.0				**225** (12)	**220** (15)	**148.2** (16)	**100.4** (27)			
250.0				**193.3** (25)	**181.8** (30)	**141.6** (20)	**87.9** (36)			
500.0				**163.2** (36)	**156.8** (40)	**103.1** (42)	**61.7** (55)			
IC50		< 500.0		> 500.0			Approx 400.0			

Note: Activities are reported in protein/min in bold print. In parentheses below each activity is (%) inhibition relative to the activity determined at the same substrate concentration [S] in the absence of ADD Cmpd (i.e. 0 uM). When calculated, an ADD Cmpd IC50 value (uM) is also shown in bold print in the bottom row for each substrate concentration.

Ki(uM): 371.0 Inhibition (Type): mixed/competitive

Comments to Supplier: ADD 348014 is a weak inhibitor of CYP2A6 in vitro and high concentrations in vivo could cause minor inhibition of other drugs metabolized by CYP2A6 although the possibility appears remote.

6/3/2010 1:22:30 PM

KM314

Anticonvulsant Screening Program

Test 19 Results - Human Cytochrome P450 Inhibition Studies

Add ID: 348014 D Screen ID: 4

Solvent Code: MEOH Solvent Prep:

Date Started: 25-Feb-2010 Date Completed: 25-Feb-2010

Isoform Code: CYP2C9 Enzyme Source: HBI #506

Reaction: Luciferin-H hydroxylation

ADD Cmpd Conc. (uM)	Activity @ [S]=400.0uM	Activity @ [S]=100.0uM	Activity @	Activity @	Activity @	Activity @	Activity @	Activity @	Activity @	Activity @
0	**37** (0)	**11.8** (0)								
500	**30** (19)	**9.5** (19)								
IC50		**> 500.0**								

Note: Activities are reported in protein/min in bold print. In parentheses below each activity is (%) inhibition relative to the activity determined at the same substrate concentration [S] in the absence of ADD Cmpd (i.e. 0 uM). When calculated, an ADD Cmpd IC50 value (uM) is also shown in bold print in the bottom row for each substrate concentration.

Ki(uM): Inhibition (Type):

Comments to Supplier: ADD 348014 showed only slight inhibition of CYP2C9 activity in vitro and is unlikely to show inhibitory interactions with other CYP2C9 metabolized drugs in vivo.

6/3/2010 1:22:28 PM

KM 314

Anticonvulsant Screening Program

Test 19 Results - Human Cytochrome P450 Inhibition Studies

Add ID: 348014 D Screen ID: 7

Solvent Code: MEOH Solvent Prep:

Date Started: 27-Feb-2010 **Date Completed:** 27-Feb-2010

Isoform Code: CYP2E1 Enzyme Source: HBI #227

Reaction: Chlorzoxazone 6-hydroxylation

ADD Cmpd Conc. (uM)	Activity @ [S]=500.0uM	Activity @ [S]=100.0uM	Activity @	Activity @	Activity @	Activity @	Activity @	Activity @	Activity @	Activity @
0	1,755.3 (0)	579.1 (0)								
500	1,462.6 (17)	477.9 (17)								
IC50		> 500.0								

Note: Activities are reported in protein/min in bold print. In parentheses below each activity is (%) inhibition relative to the activity determined at the same substrate concentration [S] in the absence of ADD Cmpd (i.e. 0 uM). When calculated, an ADD Cmpd IC50 value (uM) is also shown in bold print in the bottom row for each substrate concentration.

Ki(uM): Inhibition (Type):

Comments to Supplier: **ADD 348014 showed only slight inhibition of CYP2E1 activity in vitro and is unlikely to show inhibitory interactions with other CYP2E1 metabolized drugs in vivo.**

[illegible]/3/2010 1:22:24 PM

KM-314

Anticonvulsant Screening Program

Test 19 Results - Human Cytochrome P450 Inhibition Studies

Add ID: 348014 D Screen ID: 5

Solvent Code: MEOH Solvent Prep:

Date Started: 04-Mar-2010 Date Completed: 17-Mar-2010

Isoform Code: CYP2C19 Enzyme Source: Puracyp #011

Reaction: S-Mephenytoin 4-hydroxylation

ADD Cmpd Conc. (uM)	Activity @ [S]=150.0uM	Activity @ [S]=50.0uM	Activity @	Activity @ [S]=50.0uM	Activity @	Activity @ [S]=150.0uM	Activity @ [S]=100.0uM	Activity @ [S]=50.0uM	Activity @ [S]=25.0uM	Activity @
0	202.5 (0)	156.3 (0)		154.3 (0)		215.8 (0)	192.9 (0)	168.1 (0)	125.1 (0)	
500	40.3 (80)	28.2 (82)								
25.0				142.3 (8)		198.6 (8)	180.9 (6)	139.1 (17)	101.7 (19)	
50.0				102.3 (34)		185.9 (14)	158.3 (18)	123.6 (26)	79.8 (36)	
100.0				79.5 (48)		157.3 (27)	130.4 (32)	88.8 (47)	61.1 (51)	
250.0				46.2 (70)		86.7 (60)	79.1 (59)	51.6 (69)	30.6 (76)	
500.0				22.6 (85)		44.6 (79)	44.4 (77)	29.2 (83)	21.3 (83)	
IC50		< 500.0		Approx 100.0						

Note: Activities are reported in pmol/mg protein/min in bold print. In parentheses below each activity is (%) inhibition relative to the activity determined at the same substrate concentration [S] in the absence of ADD Cmpd (i.e. 0 uM). When calculated, an ADD Cmpd IC50 value (uM) is also shown in bold print in the bottom row for each substrate concentration.

Ki(uM): 54.0 Inhibition (Type): mixed/competitive

Comments to Supplier: ADD 348014 is an inhibitor of CYP2C19 in vitro and could inhibit the metabolism of other drugs metabolized by CYP2C19 in vivo.

3/31/2011 5:14:56 PM

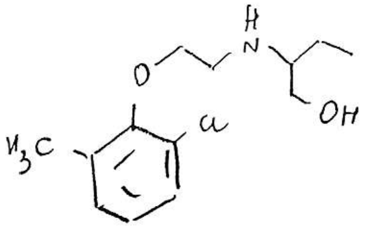

R, S

KM314

Anticonvulsant Screening Program

Test 19 Results - Human Cytochrome P450 Inhibition Studies

Add ID: 348014 D Screen ID: 6

Solvent Code: MEOH **Solvent Prep:**

Date Started: 08-Mar-2010 **Date Completed:** 09-Mar-2010

Isoform Code: CYP2D6 **Enzyme Source:** celsis M008085-JTA

Reaction: 7-Methoxy-4-(aminomethyl) coumarin O-demethylation

ADD Cmpd Conc. (uM)	Activity @ [S]=100.0uM	Activity @ [S]=25.0uM	Activity @	Activity @ [S]=100.0uM	Activity @ [S]=50.0uM	Activity @ [S]=25.0uM	Activity @ [S]=12.5uM	Activity @	Activity @	Activity @
0	75.1 (0)	26.9 (0)		52.4 (0)	47.7 (0)	19.8 (0)	8 (0)			
500	0 (100)	0 (100)								
0.39				44 (16)	40.3 (16)	16.1 (19)	5.6 (30)			
1.56				32.7 (38)	26.3 (45)	8.4 (58)	2 (75)			
6.25				13.8 (74)	7.3 (85)	0 (100)	0 (100)			
25.0				6 (89)	3.1 (94)	0 (100)	0 (100)			
IC50		< 500.0				Approx 1.3				

Note: Activities are reported in protein/min in bold print. In parentheses below each activity is (%) inhibition relative to the activity determined at the same substrate concentration [S] in the absence of ADD Cmpd (i.e. 0 uM). When calculated, an ADD Cmpd IC50 value (uM) is also shown in bold print in the bottom row for each substrate concentration.

Ki(uM): 1.4 **Inhibition (Type):** mixed/competitive

Comments to Supplier: ADD 348014 is a strong inhibitor of CYP2D6 in vitro and will likely inhibit the metabolism of drugs metabolized by CYP2D6 in vivo.

6/3/2010 1:22:26 PM

Anticonvulsant Screening Program

Test 76 Results - In-vitro Hippocampal Slice Culture Neuroprotection Assay (NP)

Add ID: 389080 B **Screen ID:** 2

Solvent Code: DMSO **Solvent Prep:**

Date Started: 24-Mar-2010 **Date Completed:** 26-Mar-2010

Reference: 450:103

Summary of NP Assay: NMDA

- **Test Result:** No Neuroprotection
- **ADD compounds evaluated:** 389080 348014
  BM-233 KM-314

Note: This experiment is run at two different concentrations of candidate drug against a fixed concentration of excitotoxin. If multiple candidates from the same participant source are scheduled for NP screening we will test compounds in pairs whenever possible.

Comments to Supplier:

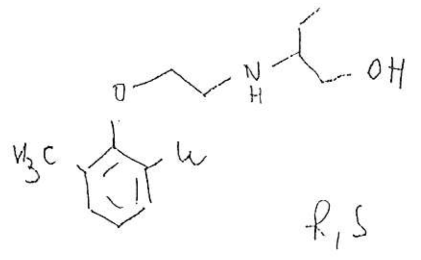

348014
KM-314

4/6/2010 11:23:42 AM

TEST 76: *in vitro* HIPPOCAMPAL SLICE CULTURE NEUROPROTECTION ASSAY

Compound 1 : **ADD Number:** 348014 KM-314 **Batch:** C **Date Started:** 24-Mar-2010

Compound 2 : **ADD Number:** 389080 BM233 **Batch:** B **Date Completed:** 26-Mar-2010

References: 450: 103

Excitotoxin: NMDA **Insult Duration:** 4 Hours **Solvent:** DMSO

Primary Screen Results: No neuroprotection observed

EXPERIMENT IMAGES & WELL DESCRIPTION

A1 NMDA 10µM

A2 NMDA 10µM + 348014 10µM+389080 10µM

A3 NMDA 10µM + 348014 10µM+389080 10µM

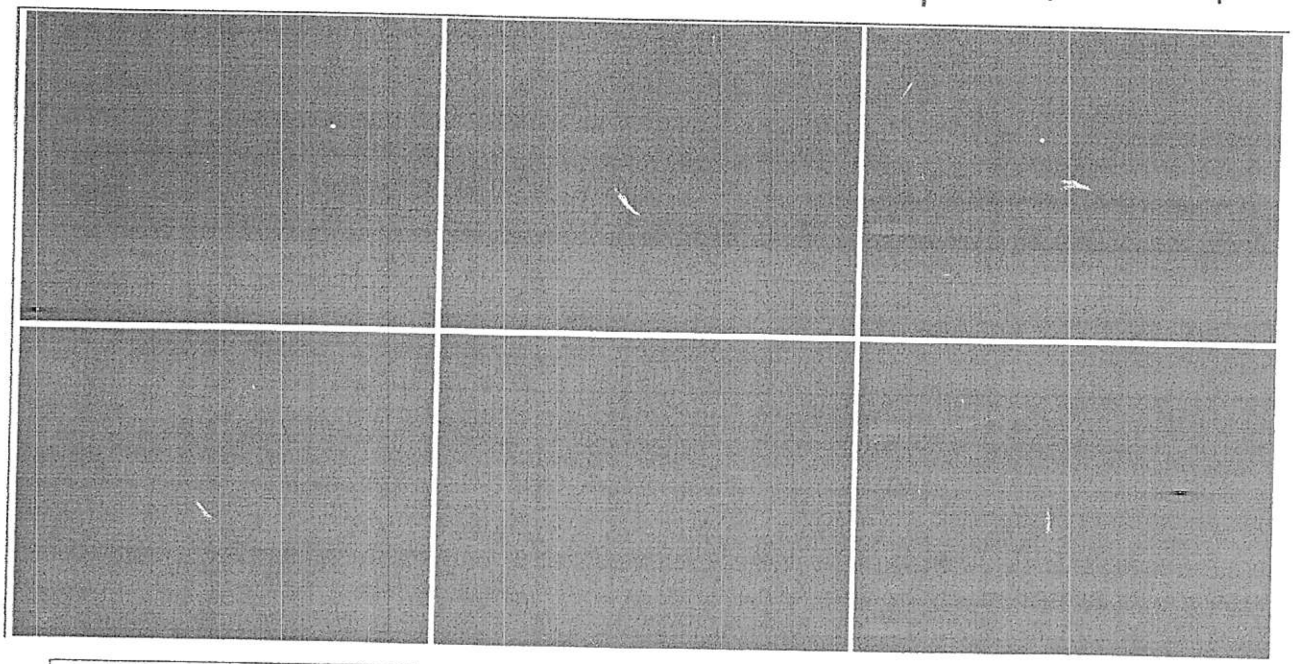

B1 NMDA 10µM

B2 NMDA 10µM + 348014 100µM+389080 100µM

B3 NMDA 10µM + 348014 100µM+389080 100µM

PRIMARY SCREEN EXPERIMENT DESCRIPTION

The "Primary Screen Experiment" is a qualitative assessment of the ability of a compound to prevent excitotoxic cell death. Organotypic hippocampal slice cultures are treated with N-methyl-D-aspartate (NMDA) or kainic acid (KA) to induce neuronal cell death. Propidium iodide (PI), a membrane-impermeant compound, is included in all wells of the culture plate. Dying cells have compromised cell membranes, thus PI may diffuse into the cell, intercalate with DNA and fluoresce. Thus, the intensity of the PI fluorescence is proportional to the amount of cell death in the individual slices. Hippocampal slice cultures are treated with the excitotoxin alone, or where indicated above, with the excitotoxin and either one or two investigational compounds at the concentrations indicated. If neuroprotection occurs as a consequence of the added compound, slice cultures will have a visibly reduced fluorescent intensity when compared to the slice cultures that have been treated with the excitotoxin alone.

Anticonvulsant Screening Program

Test 76 Results - In-vitro Hippocampal Slice Culture Neuroprotection Assay (NP)

Add ID: 348014 C **Screen ID:** 1

Solvent Code: DMSO **Solvent Prep:**

Date Started: 24-Mar-2010 **Date Completed:** 26-Mar-2010

Reference: 450:103

Summary of NP Assay: NMDA

- **Test Result:** No Neuroprotection
- **ADD compounds evaluated:** 348014 389080
  KM-314 BM-233

Note: This experiment is run at two different concentrations of candidate drug against a fixed concentration of excitotoxin. If multiple candidates from the same participant source are scheduled for NP screening we will test compounds in pairs whenever possible.

Comments to Supplier:

TEST 76: *in vitro* HIPPOCAMPAL SLICE CULTURE NEUROPROTECTION ASSAY

Compound 1 : **ADD Number:** 348014 **Batch:** C **Date Started:** 24-Mar-2010

KM-314

Compound 2 : **ADD Number:** 389080 **Batch:** B **Date Completed:** 26-Mar-2010

BM-233

References: 450: 103

Excitotoxin: NMDA **Insult Duration:** 4 Hours **Solvent:** DMSO

Primary Screen Results: No neuroprotection observed

EXPERIMENT IMAGES & WELL DESCRIPTION

A1 NMDA 10μM

A2 NMDA 10μM + 348014 10μM+389080 10μM

A3 NMDA 10μM + 348014 10μM+389080 10μM

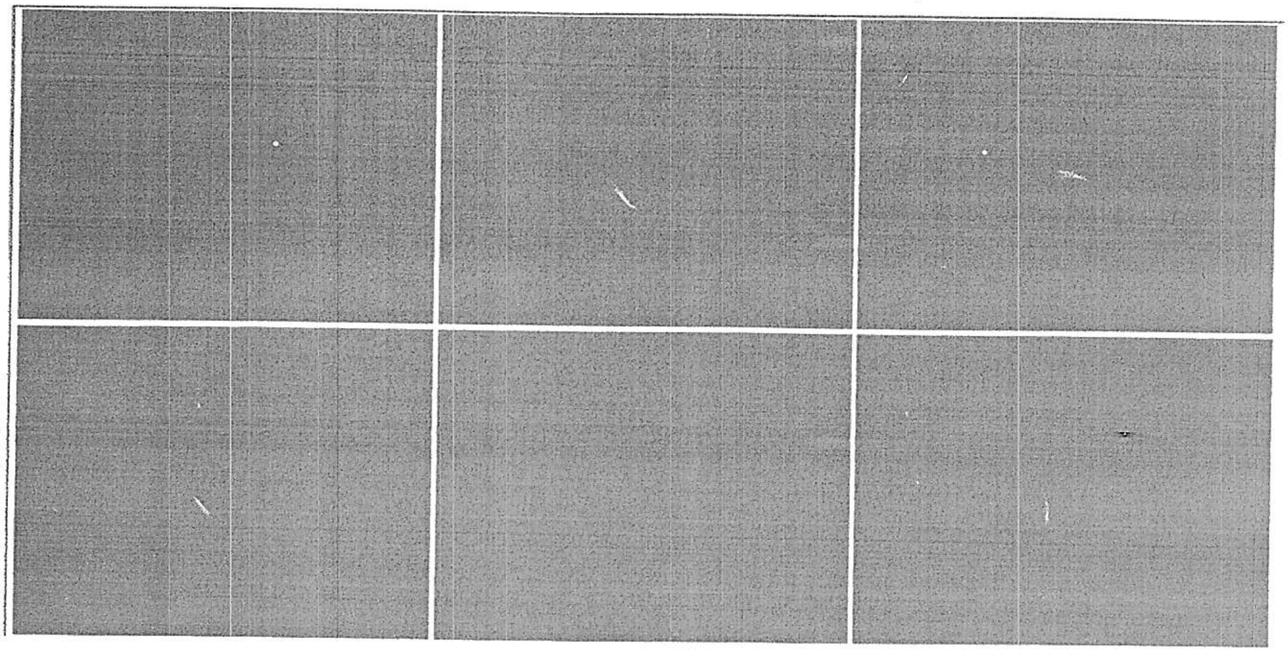

B1 NMDA 10μM

B2 NMDA 10μM + 348014 100μM+389080 100μM

B3 NMDA 10μM + 348014 100μM+389080 100μM

PRIMARY SCREEN EXPERIMENT DESCRIPTION

The "Primary Screen Experiment" is a qualitative assessment of the ability of a compound to prevent excitotoxic cell death. Organotypic hippocampal slice cultures are treated with N-methyl-D-aspartate (NMDA) or kainic acid (KA) to induce neuronal cell death. Propidium iodide (PI), a membrane-impermeant compound, is included in all wells of the culture plate. Dying cells have compromised cell membranes, thus PI may diffuse into the cell, intercalate with DNA and fluoresce. Thus, the intensity of the PI fluorescence is proportional to the amount of cell death in the individual slices. Hippocampal slice cultures are treated with the excitotoxin alone, or where indicated above, with the excitotoxin and either <u>one or two</u> investigational compounds at the concentrations indicated. If neuroprotection occurs as a consequence of the added compound, slice cultures will have a visibly reduced fluorescent intensity when compared to the slice cultures that have been treated with the excitotoxin alone.

Anticonvulsant Screening Program

Test 76 Results - In-vitro Hippocampal Slice Culture Neuroprotection Assay (NP)

Add ID: 389080 **B** **Screen ID: 3**

Solvent Code: DMSO **Solvent Prep:**

Date Started: 24-Mar-2010 **Date Completed:** 26-Mar-2010

Reference: 450:103

Summary of NP Assay: Kainic acid

- Test Result: No Neuroprotection
- ADD compounds evaluated: 389080 348014

BM-233 KM-314

Note: This experiment is run at two different concentrations of candidate drug against a fixed concentration of excitotoxin. If multiple candidates from the same participant source are scheduled for NP screening we will test compounds in pairs whenever possible.

Comments to Supplier:

TEST 76: *in vitro* HIPPOCAMPAL SLICE CULTURE NEUROPROTECTION ASSAY

Compound 1 : **ADD Number:** 348014 **Batch:** C **Date Started:** 24-Mar-2010

KM 314

Compound 2 : **ADD Number:** 389080 **Batch:** B **Date Completed:** 26-Mar-2010

BM-233

References: 450: 103

Excitotoxin: Kainic Acid **Insult Duration:** 4 Hours **Solvent:** DMSO

Primary Screen Results: No neuroprotection observed

EXPERIMENT IMAGES & WELL DESCRIPTION

A1 KA 20µM

A2 KA 20µM +
348014 10µM+389080 10µM

A3 KA 20µM +
348014 10µM+389080 10µM

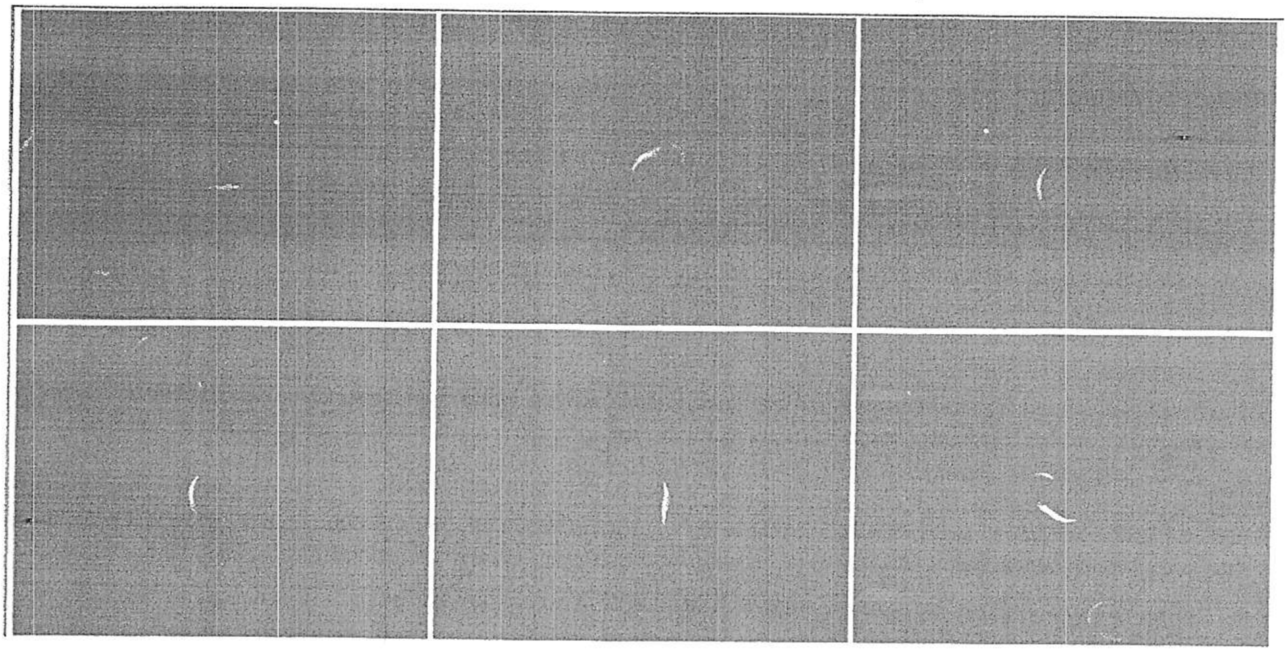

B1 KA 20µM

B2 KA 20µM +
348014 100µM+389080 100µM

B3 KA 20µM +
348014 100µM+389080 100µM

PRIMARY SCREEN EXPERIMENT DESCRIPTION

The "Primary Screen Experiment" is a qualitative assessment of the ability of a compound to prevent excitotoxic cell death. Organotypic hippocampal slice cultures are treated with N-methyl-D-aspartate (NMDA) or kainic acid (KA) to induce neuronal cell death. Propidium iodide (PI), a membrane-impermeant compound, is included in all wells of the culture plate. Dying cells have compromised cell membranes, thus PI may diffuse into the cell, intercalate with DNA and fluoresce. Thus, the intensity of the PI fluorescence is proportional to the amount of cell death in the individual slices. Hippocampal slice cultures are treated with the excitotoxin alone, or where indicated above, with the excitotoxin and either <u>one or two</u> investigational compounds at the concentrations indicated. If neuroprotection occurs as a consequence of the added compound, slice cultures will have a visibly reduced fluorescent intensity when compared to the slice cultures that have been treated with the excitotoxin alone.

Anticonvulsant Screening Program

Test 76 Results - In-vitro Hippocampal Slice Culture Neuroprotection Assay (NP)

Add ID: 348014 C Screen ID: 2

Solvent Code: DMSO **Solvent Prep:**

Date Started: 24-Mar-2010 **Date Completed:** 26-Mar-2010

Reference: 450:103

Summary of NP Assay: Kainic acid

- **Test Result:** No Neuroprotection
- **ADD compounds evaluated:** 348014 389080
  KM-314 BM-233

Note: This experiment is run at two different concentrations of candidate drug against a fixed concentration of excitotoxin. If multiple candidates from the same participant source are scheduled for NP screening we will test compounds in pairs whenever possible.

Comments to Supplier:

TEST 76: *in vitro* HIPPOCAMPAL SLICE CULTURE NEUROPROTECTION ASSAY

Compound 1 : ADD Number: 348014 KM-314 Batch: C Date Started: 24-Mar-2010

Compound 2 : ADD Number: 389080 BM 233 Batch: B Date Completed: 26-Mar-2010

References: 450: 103

Excitotoxin: Kainic Acid Insult Duration: 4 Hours Solvent: DMSO

Primary Screen Results: No neuroprotection observed

EXPERIMENT IMAGES & WELL DESCRIPTION

A1 KA 20μM

A2 KA 20μM + 348014 10μM+389080 10μM

A3 KA 20μM + 348014 10μM+389080 10μM

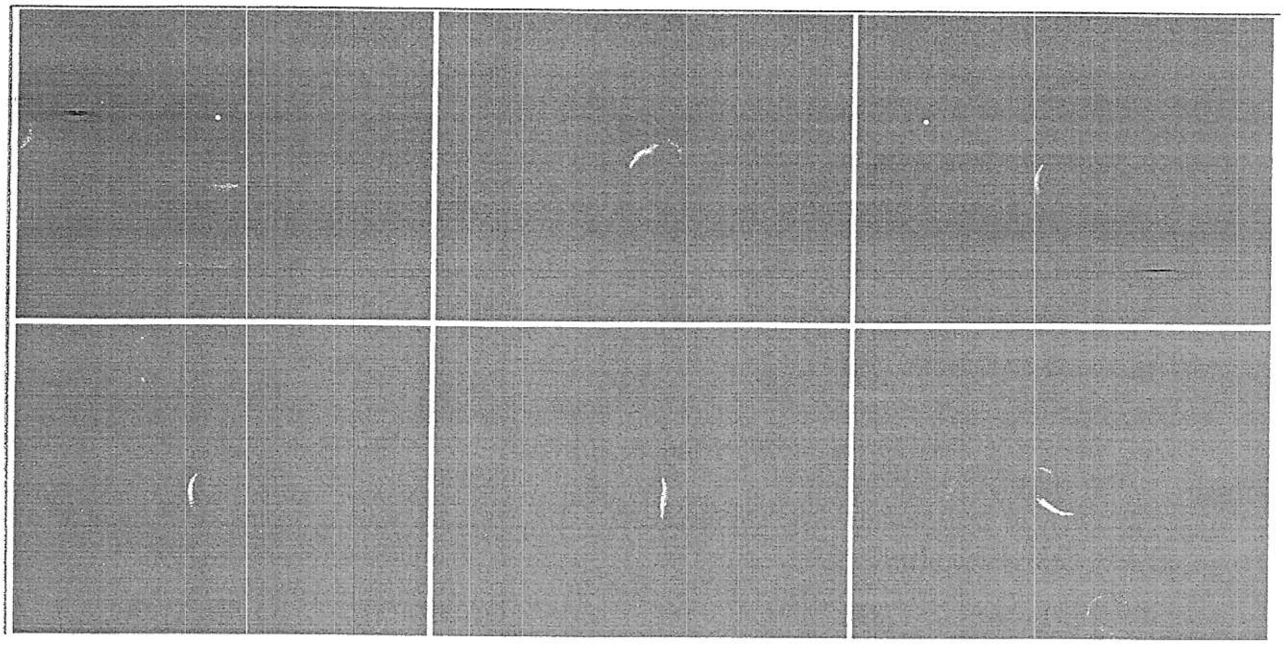

B1 KA 20μM

B2 KA 20μM + 348014 100μM+389080 100μM

B3 KA 20μM + 348014 100μM+389080 100μM

PRIMARY SCREEN EXPERIMENT DESCRIPTION

The "Primary Screen Experiment" is a qualitative assessment of the ability of a compound to prevent excitotoxic cell death. Organotypic hippocampal slice cultures are treated with N-methyl-D-aspartate (NMDA) or kainic acid (KA) to induce neuronal cell death. Propidium iodide (PI), a membrane-impermeant compound, is included in all wells of the culture plate. Dying cells have compromised cell membranes, thus PI may diffuse into the cell, intercalate with DNA and fluoresce. Thus, the intensity of the PI fluorescence is proportional to the amount of cell death in the individual slices. Hippocampal slice cultures are treated with the excitotoxin alone, or where indicated above, with the excitotoxin and either <u>one or two</u> investigational compounds at the concentrations indicated. If neuroprotection occurs as a consequence of the added compound, slice cultures will have a visibly reduced fluorescent intensity when compared to the slice cultures that have been treated with the excitotoxin alone.

KM-314

Anticonvulsant Screening Program

Test 7 Results - Anticonvulsant Evaluation (6Hz, Mice)

Add ID: 348014 C Screen ID: 2

Solvent Code: MC Solvent Prep: M&P,SB Route Code: IP

Animal Weight: - g Current(mA): 32

Date Started: 17-Jun-2010 Date Completed: 25-Jun-2010

Reference: 456:47-51, 58

ED50 Value

Test	Time(Hrs)	ED50	95% Confidence Interval	Slope	STD Err	PI Value
6HZ	0.25	32.4	28.4 - 36	15.2	4.6	

ED50 Biological Response

Test	Dose (mg/kg)	Dths	N / F C
6HZ	23		0 / 8
6HZ	30		3 / 8
6HZ	37		6 / 8
6HZ	45		8 / 8

Time to Peak Effect

Time (Hours)			0.25	0.5	1.0	2.0	4.0	6.0	8.0	24	3.0
Test	Dose	Dths	N / F C	N / F C	N / F C	N / F C	N / F C	N / F C	N / F C	N / F C	N / F C
6HZ	30		3 / 8	1 / 8	/	/	/	/	/	/	/
6HZ	60		4 / 4	4 / 4	0 / 4	0 / 4	0 / 4	/	/	/	/

Note: N/F = number of animals active or toxic over the number tested.

C= Comment code

Comments to Supplier:

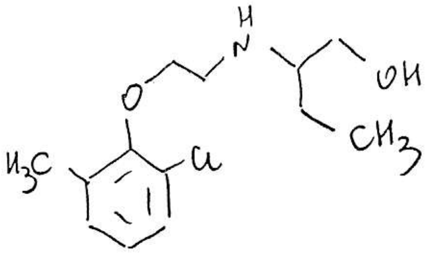

R,S

8/2/2010 11:59:18 AM
