# Supplementary material for: KM-408, a novel phenoxyalkyl derivative as a potential anticonvulsant and analgesic compound for the treatment of neuropathic pain
Source: Pharmacol Rep. 2022 Nov 19;75(1):128–65. doi: 10.1007/s43440-022-00431-7 (PMC9889419; doi:10.1007/s43440-022-00431-7)
Supplement: Supplementary file 3 — Supplementary file3 (PDF 5755 KB) [file 43440_2022_431_MOESM3_ESM.pdf]

KH-408

Anticonvulsant Screening Project

Test 1 Results - Mice I.P. Identification

Add ID: 367061 U **Screen ID:** 1

Solvent Code: MC **Solvent Prep:** TT

Animal Weight: 21.0 to 25.5 g

Date Started: 09-Aug-2005 **Date Completed:** 15-Aug-2005

Reference: 386:157,163

Response

Time (Hours)				0.5		4.0		0.25		1.0		2.0		6.0		3.0		8.0		24	
Test	Dose	Form	Dths	N / F	C	N / F	C	N / F	C	N / F	C	N / F	C	N / F	C	N / F	C	N / F	C	N / F	C
MES	3	SOL		0 / 4		/		/		/		/		/		/		/		/	
MES	10	SOL		0 / 4		/		/		/		/		/		/		/		/	
MES	30	SOL		1 / 1		0 / 1		/		/		/		/		/		/		/	
MES	100	SOL		1 / 1		0 / 3		/		/		/		/		/		/		/	
SCMET	30	SOL		0 / 1		0 / 1		/		/		/		/		/		/		/	
SCMET	100	SOL		0 / 0		0 / 1		/		/		/		/		/		/		/	
TOX	3	SOL		0 / 4		/		/		/		/		/		/		/		/	
TOX	10	SOL		0 / 4		/		/		/		/		/		/		/		/	
TOX	30	SOL		3 / 4		0 / 2		/		/		/		/		/		/		/	
TOX	100	SOL	3	8 / 8	*	1 / 4		/		/		/		/		/		/		/	
TOX	300	SOL	4	4 / 4	1	/		/		/		/		/		/		/		/	

Response Comments

TEST	DOSE (mg/kg	TIME	CODE	COMMENT
TOX	100	0.5	14	Unable to grasp rotorod
TOX	100	0.5	1	Death
TOX	300	0.5	1	Death

Comments to Supplier:

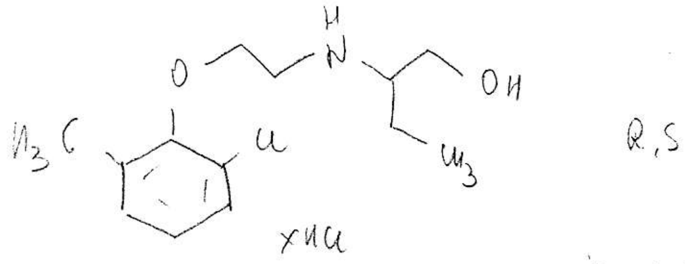

VM 408

Anticonvulsant Screening Project

Test 8 Results -Anticonvulsant Identification (Rats I.P.)

Add ID: 367061 U **Screen ID:** 1

Solvent Code: MC **Solvent Prep:** TT

Animal Weight: 105 to 135 g

Date Started: 04-May-2007 **Date Completed:** 04-May-2007

Reference: 412:124

Time to Peak Effect

Test	Dose (mg/kg)	# Dths	0.25	0.5	1.0	2.0	4.0	6.0	8.0	24	3.0
			N / F C	N / F C	N / F C	N / F C	N / F C	N / F C	N / F C	N / F C	N / F C
'ES	5		1 / 4	2 / 4	0 / 4	1 / 4	0 / 4	/	/	/	/
TOX	5		0 / 4	0 / 4	0 / 4	0 / 4	0 / 4	/	/	/	/

Comments to Supplier:

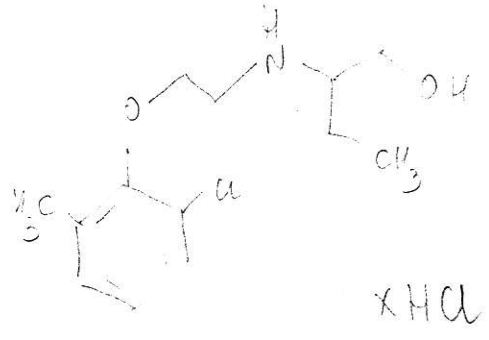

P.S

KM-408

Anticonvulsant Screening Project

Test 11 Results - Toxicity Screen (Rats I.P.)

Add ID: 367061 A **Screen ID:** 1

Solvent Code: MC **Solvent Prep:** TT,SB **Route Code:** IP

Animal Weight: to g

Date Started: 27-Sep-2007 **Date Completed:** 18-Oct-2007

Reference: 418:192,242

Time to Peak Effect

Test	Dose (mg/kg)	# Dths	0.25		0.5		1.0		2.0		4.0		6.0		8.0		24		3.0	
			N / F	C	N / F	C	N / F	C	N / F	C	N / F	C	N / F	C	N / F	C	N / F	C	N / F	C
TOX	10		0 / 2		0 / 2		0 / 2		0 / 2		0 / 2		/		/		/		/	
TOX	30		0 / 2		0 / 2		0 / 2		0 / 2		0 / 2		/		/		/		/	
T[illegible]	100		0 / 2		0 / 2		0 / 2		0 / 2		0 / 2		/		/		/		/	
TOX	300	2	2 / 2	*	/		/		/		/		/		/		/		/	

Response Comments

TEST	DOSE (mg/kg)	TIME	CODE	COMMENT
TOX	300	0.25	1	Death
TOX	300	0.25	25	Myoclonic jerks

Comments to Supplier:

KM-408

Anticonvulsant Screening Project

Test 11 Results - Preliminary Hippocampal Kindling Screen - Rats IP

Add ID: 367061 B **Screen ID:** 1

olvent Code: MC **Solvent Prep:** M&P,SB **Route Code:** IP

nimal Weight: to g

ate Started: 30-Oct-2007 **Date Completed:** 30-Oct-2007

eference: 418:252-256

Dose: 200 mg/kg Time of Maximum Effect: 15 to min

Rat #	Comment Code	Seizure Score				Afterdischarge Duration (secs)			
		Pre-Drug		Drug		Pre-Drug		Drug	
		Low	High	Low	High	Low	High	Low	High
[illegible]	1	4 - 5		-		21 - 31		-	
2	1	5 -		-		29 - 48		-	

esponse Comments

RAT #	DOSE (mg/kg)	CODE	COMMENT
1	200	1	Death
2	200	1	Death

omments to Supplier: Rats were dead before the 15 min stimulation.

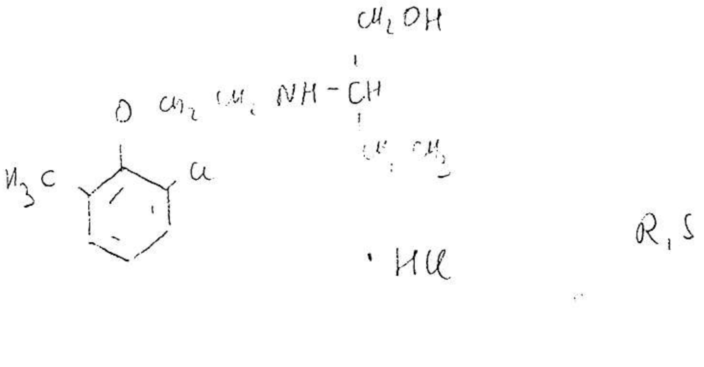

KM-408

Anticonvulsant Screening Program

Test 71 Results - Pilocarpine-induced Status, Rats - Time 0 Min

Add ID: 367061 U **Screen ID:** 1

**Solvent Code:**	MC	**Solvent Prep:**	M&P,SB	**Route Code:**	IP
**Date Started:**	30-Jan-2008	**Date Completed:**	31-Jan-2008		

Reference: CM1:153-154

Response Data

Dose (mg/kg)	Time (hrs) [a]	N / F	C	Dths	Avg. Weight Change(g) +/- S.E.M [b]	
					Protected Rats	Non-Protected Rats
100.00	0.0	0 / 8		8		

a Post first Stage III seizure
b Weight change 24 hours Post first Stage III seizure

Comments to Supplier:

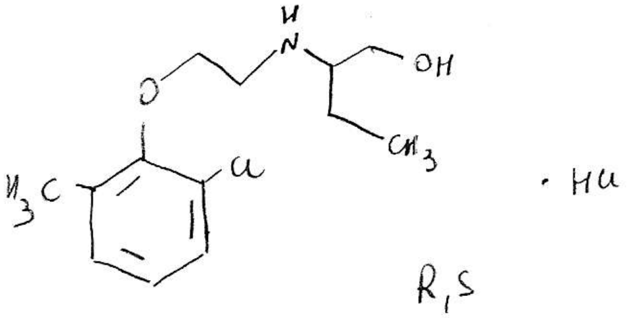

2/13/2008 3:14:51 PM

Anticonvulsant Screening Program

KM-408

Test 4 Results - Mice I.P. Quantification

Add ID: 367061 B Screen ID: 1

Solvent Code: MC **Solvent Prep:** TT,SB

Animal Weight: - g

Date Started: 13-Sep-2009 **Date Completed:** 15-Sep-2009

Reference: 446:74-80

ED50 Value

Test	Time(Hrs)	ED50	95% Confidence Interval	Slope	STD Err	PI Value
MES	0.25	13.3	11 - 15.6	6.5	1.7	
SCMET	0.25	> 100	-			
TOX	0.25	64.3	45.1 - 82.4	4.7	1.3	

ED50 Biological Response

Test	Dose (mg/kg)	Dths	N / F	C
MES	4		0 / 8	
MES	7.5		1 / 8	
MES	12		3 / 16	
MES	15		7 / 8	
MES	18		8 / 8	
MES	25		7 / 8	
SCMET	50		0 / 6	3
SCMET	100		0 / 8	22
TOX	25		0 / 8	
TOX	50		3 / 8	14
TOX	85		7 / 8	*
TOX	100		5 / 8	*
TOX	170	7	8 / 8	*

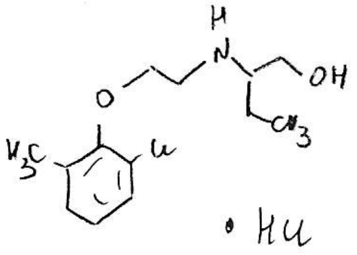

ED50 Biological Response Comments

Test	Dose (mg/kg)	Time	Code	Comment
SCMET	50	0.25	3	Death following continuous seizure
SCMET	100	0.25	22	Continuous seizure activity
TOX	50	0.25	14	Unable to grasp rotorod

KM-408

Anticonvulsant Screening Program

Test 4 Results - Mice I.P. Quantification

Add ID: 367061 B Screen ID: 1

TOX	85	0.25	14	Unable to grasp rotorod
TOX	85	0.25	34	Muscle spasms
TOX	100	0.25	14	Unable to grasp rotorod
TOX	100	0.25	34	Muscle spasms
TOX	170	0.25	1	Death
TOX	170	0.25	14	Unable to grasp rotorod

.ime to Peak Effect

Time (Hours)			0.25		0.5		1.0		2.0		4.0		6.0		8.0		24		3.0
Test	Dose	Dths	N / F	C	N / F	C	N / F	C	N / F	C	N / F	C	N / F	C	N / F	C	N / F	C	N / F
MES	25		3 / 4		0 / 4		0 / 4		0 / 4		0 / 4		/		/		/		/
TOX	50		3 / 8	14	0 / 8		0 / 8		0 / 8		/		/		/		/		/

Note: N/F = number of animals active or toxic over the number tested.

C= Comment code

Response Comments

Test	Dose (mg/kg)	Time	Code	Comments
TOX	50	0.25	14	Unable to grasp rotorod

Comments to Supplier:

Anticonvulsant Screening Program

KM-408

Test 15 Results - IV Metrazol

Add ID: 367061 U **Screen ID:** 1

**Solvent Code:**	MC	**Solvent Prep:**	M&P,SB	**Route Code:**	IP
**Time of Test:**	0.25 (hrs)	**Infusion Rate:**	0.34 (ml/min)		
**MES ED50:**	13.00 (mg/kg)	**TD50:**	64.00 (mg/kg)		
**Date Started:**	18-Feb-2010	**Date Completed:**	18-Feb-2010		
**Reference:**	449: 60-61				

Anlaysis

Dose (mg/kg)		Weight (grams)	Time to Twitch	Twitch (mg/kg)	Time to Clonus	Clonus (mg/kg)
0	Mean	29.10	29.4	28.7	32.1	31.4
	Std. Err	0.66	1.12	1.21	1.21	1.37
	P-value					
13	Mean	28.10	26.0	26.2	28.5	28.7
	Std. Err	0.57	1.11	1.12	1.25	1.25
	P-value	0.132	0.022	0.074	0.024	0.082
64	Mean	28.15	22.1	22.4	25.5	25.8
	Std. Err	0.90	1.11	1.19	0.82	1.00
	P-value	0.202	0.000	0.000	0.000	0.002

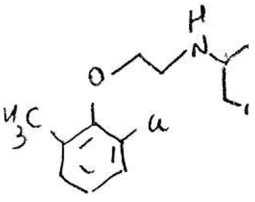

o HCl

R,S

Response

Dose (mg/kg)	Animal #	Weight (grams)	Time to Twitch	Twitch (mg/kg)	Time to Clonus	Clonus (mg/kg)
0	01	28.5	29.50	29.33	32.00	31.81
0	02	27.0	25.00	26.23	27.00	28.33
0	03	33.0	31.00	26.62	33.00	28.33
0	04	29.5	31.50	30.25	33.00	31.69
0	05	30.0	25.00	23.61	26.00	24.56
0	06	28.0	34.00	34.40	36.00	36.43
0	07	29.5	26.00	24.97	28.00	26.89
0	08	31.5	33.50	30.13	36.00	32.38
0	09	28.0	26.00	26.31	36.00	36.43
0	10	26.0	32.00	34.87	34.00	37.05
0	11					
0	12					
0	13					
0	14					

Anticonvulsant Screening Program

Test 15 Results - IV Metrazol

Add ID: 367061 U Screen ID: 1

0	15					
0	16					
0	17					
0	18					
0	19					
0	20					
13	01	30.0	29.00	27.39	33.00	31.17
13	02	26.5	24.50	26.19	25.50	27.26
13	03	26.0	30.50	33.24	34.00	37.05
13	04	27.5	25.00	25.76	27.00	27.82
13	05	28.0	29.00	29.35	31.00	31.37
13	06	25.5	18.50	20.56	20.50	22.78
13	07	31.0	28.00	25.59	29.50	26.96
13	08	29.5	24.00	23.05	27.00	25.93
13	09	29.0	24.00	23.45	27.00	26.38
13	10	28.0	27.00	27.32	30.00	30.36
13	11					
13	12					
13	13					
13	14					
13	15					
13	16					
13	17					
13	18					
13	19					
13	20					
64	01	26.0	24.50	26.70	26.00	28.33
64	02	30.5	27.00	25.08	29.00	26.94
64	03	26.5	22.00	23.52	23.50	25.13
64	04	26.0	20.50	22.34	25.00	27.24
64	05	30.5	18.00	16.72	24.50	22.76
64	06	28.5	25.00	24.85	28.00	27.84
64	07	30.5	17.00	15.79	20.50	19.04
64	08	31.5	26.50	23.84	28.50	25.63
64	09	22.5	20.00	25.19	24.00	30.22
64	10	29.0	20.00	19.54	25.50	24.91

Anticonvulsant Screening Program

Test 15 Results - IV Metrazol

Add ID: 367061 U Screen ID: 1

64	11					
64	12					
64	13					
64	14					
64	15					
64	16					
64	17					
64	18					
64	19					
64	20					

Comments to Supplier:

KM-408

Anticonvulsant Screening Program

Test 7 Results - Anticonvulsant Evaluation (6Hz, Mice)

Add ID: 367061 C Screen ID: 1

Solvent Code:	MC	Solvent Prep:	M&P,SB	Route Code:	IP
Animal Weight:	- g			Current(mA):	32
Date Started:	19-Oct-2010	Date Completed:	20-Oct-2010		
Reference:	456:278-280				

ED50 Value

Test	Time(Hrs)	ED50	95% Confidence Interval	Slope	STD Err	PI Value
6HZ	0.25	29.4	23.5 - 41.8	6	1.7	

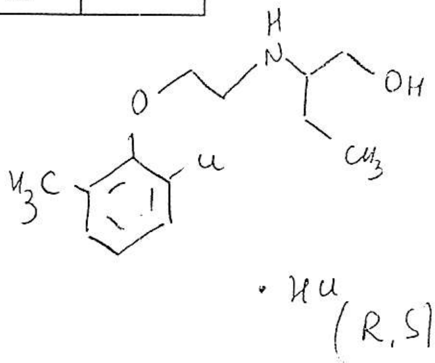

ED50 Biological Response

Test	Dose (mg/kg)	Dths	N / F C
6HZ	15		0 / 8
6HZ	20		1 / 8
6HZ	25		4 / 8
6HZ	50		7 / 8

Time to Peak Effect

Time (Hours)			0.25	0.5	1.0	2.0	4.0	6.0	8.0	24	3.0
Test	Dose	Dths	N / F C	N / F C	N / F C	N / F C	N / F C	N / F C	N / F C	N / F C	N / F C
6HZ	25		2 / 4	1 / 4	0 / 4	2 / 4	0 / 4	/	/	/	/

Note: N/F = number of animals active or toxic over the number tested.

C= Comment code

Comments to Supplier: Test-4 has already been done and TD-50 has been determined.

KM-408

Anticonvulsant Screening Program
Test 22 Results - Formalin Test (Mice I.P.)

Add ID: 367061 C Screen ID: 1

Solvent Code:	MC	Solvent Prep:	TT,SB	Route Code:	IP
Time of Test:	0.25 (hrs)				
ED50:	13.25 (mg/kg)	TD50:	64.28 (mg/kg)		
Date Started:	05-Jan-2011	Date Completed:	06-Jan-2011		
Reference:	F5:27				

Analysis

		Area Under the Curve				
Dose (mg/kg)	Test	Control	Drug Treated	% of Control	S.E.M	p Value
13.25	Acute	277.86	185.3	66.68	9.49	< 0.05
13.25	Inflammatory	636.44	531.5	83.52	14.14	> 0.05

Response

Trial 1

		Duration of Licking (sec)											
Dose (mg/kg)	Animal #	0 min	5 min	10 min	15 min	20 min	25 min	30 min	35 min	40 min	45 min	50 min	55 min
0.0	01	56.61	0.00	0.00	0.00	0.00	0.00	0.00	0.00	45.77			
0.0	02	51.45	0.00	0.00	14.64	29.04	13.09	43.45	39.20	43.82			
0.0	03	55.53	0.00	0.00	5.21	44.56	80.54	34.07	73.77	14.91			
0.0	04	30.48	8.47	21.63	59.52	44.19	60.61	43.74	40.19	60.27			
0.0	05	53.92	3.60	0.00	0.00	36.60	31.36	53.87	0.00	0.00			
0.0	06	64.65	0.00	0.00	0.00	51.63	0.74	0.00	0.00	0.55			
0.0	07	67.53	2.80	0.00	1.62	9.07	0.00	0.00	0.00	0.00			
0.0	08	44.99	4.56	0.00	0.00	38.54	52.19	0.00	0.00	6.56			

Trial 1

		Duration of Licking (sec)											
Dose (mg/kg)	Animal #	0 min	5 min	10 min	15 min	20 min	25 min	30 min	35 min	40 min	45 min	50 min	55 min
13.25	01	27.75	0.00	7.70	0.00	27.42	0.00	0.00	0.00	13.76			
13.25	02	26.02	38.27	0.00	24.71	56.13	36.86	49.52	3.58	17.73			
13.25	03	17.67	0.00	0.00	33.50	51.97	9.22	15.70	0.00	0.40			
13.25	04	23.83	0.00	0.00	0.00	19.17	12.21	51.71	39.64	12.67			

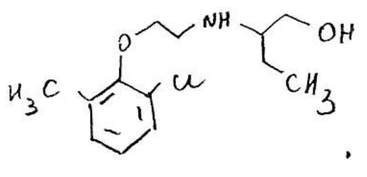

R,S

· HCl

KM408

Anticonvulsant Screening Program
Test 22 Results - Formalin Test (Mice I.P.)

Add ID: 367061 C Screen ID: 1

**13.25**	05	27.54	22.10	0.00	0.00	13.85	15.31	0.00	44.21	23.81			
**13.25**	06	35.73	0.00	0.00	0.00	0.00	60.24	0.00	0.00	0.00			
**13.25**	07	40.83	0.00	0.00	0.00	47.38	11.29	11.26	0.00	0.00			
**13.25**	08	36.65	0.00	0.00	0.00	51.70	73.81	18.01	0.00	0.00			

Comments to Supplier:

KM-408

Anticonvulsant Screening Program
Test 26 Results - Corneal Kindled Mouse

Add ID: 367061 C Screen ID: 1

Solvent Code: MC Solvent Prep: M&P Route Code: IP

Date Started: 14-Jan-2011 Date Completed: 02-Feb-2011

Reference: 457: 241-266

Response

Dose (mg/kg)	Time (hrs)	N / F	C	Individual Seizure Scores	Average Seizure Score	Toxicity N / F
15	0.25	0 / 8		5, 4, 5, 5, 4, 5, 4, 5	4.6	0 / 8
30	0.25	6 / 16		3, 1, 3, 1, 4, 4, 0, 4, 1, 4, 4, 4, 4, 4, 4, 5	2.5	1 / 8
60	0.25	4 / 8	14	0, 0, 5, 4, 5, 0, 3, 5	2.8	8 /-8
120	0.25	/	3	4/4 died before test.		4 / 4

Response Comments

Test	Dose (mg/kg)	Time (Hrs)	Code	Comment
CKM	60	0.25	14	Unable to grasp rotorod
CKM	120	0.25	3	Death following continuous seizure

Time Course

Dose (mg/kg)	Time (hrs)	N / F	C	Individual Seizure Scores	Average Seizure Score
30	0.25	4 / 4		3, 1, 3, 1	2.0

Comments to Supplier: The 4 mice used in the Time Course were used in the dose-response data. Unable to get a good ED 50 and 95% confidence interval with this data.

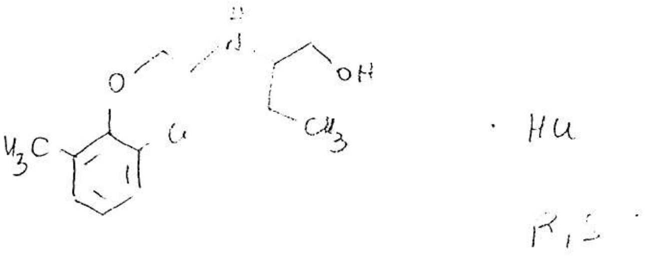

KM-408

Anticonvulsant Screening Program

Test 10 Results - Anticonvulsant Quantification (Rats I.P.)

Add ID: 367061 C Screen ID: 1

Solvent Code: MC

Solvent Prep: TT

Route Code: IP

Animal Weight: - g

Date Started: 26-May-2011

Date Completed: 10-Jun-2011

Reference: 466:52-67

ED50 Value

Test	Time(Hrs)	ED50	95% Confidence Interval	Slope	STD Err	PI Value
MES	0.25	5.69	3.99 - 7.25	5.69	1.79	
SCMET	0.25	> 54.0	-			
TOX	0.25	54.32	44.12 - 60.57	17.38	7.02	

ED50 Biological Response

Test	Time (hr)	Dose (mg/kg)	Dths	N / F C
MES	0.25	2.5		0 / 8
MES	0.25	5.0		3 / 7
MES	0.25	7.0		6 / 8
MES	0.25	10.0		7 / 8
SCMET	0.25	54.0		0 / 8
TOX	0.25	25.0		0 / 8
TOX	0.25	50.0		2 / 7
TOX	0.25	60.0		6 / 8
TOX	0.25	75.0		8 / 8 23

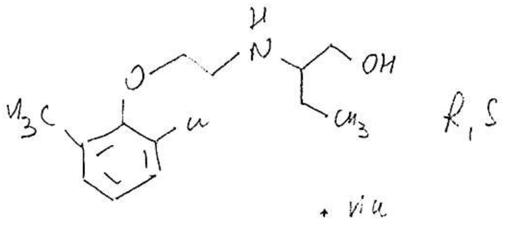

ED50 Biological Response Comments

Test	Dose (mg/kg)	Time (Hrs)	Code	Comment
TOX	75	0.25	23	Clonic seizures

Time to Peak Effect

Time (Hours)			0.25		0.5		1.0		2.0		4.0		6.0		8.0		24		3.0	
Test	Dose	Dths	N / F	C	N / F	C	N / F	C	N / F	C	N / F	C	N / F	C	N / F	C	N / F	C	N / F	C
MES	5.0		1 / 4		0 / 4		0 / 4		0 / 4		0 / 4		/		/		/		/	
TOX	75.0		8 / 8	23	8 / 8		0 / 8		/		/		/		/		/		/	

KM-408

Anticonvulsant Screening Program

Test 10 Results - Anticonvulsant Quantification (Rats I.P.)

Add ID: 367061 C Screen ID: 1

TOX	100.0	4	7 / 8	*	3 / 4	19	2 / 4	0 / 4	/	/	/	/	/
TOX	150.0	7	7 / 8	5	0 / 1		0 / 1	0 / 1	0 / 1	0 / 1	/	/	/
TOX	200.0	8	8 / 8	Z	/		/	/	/	/	/	/	/

Note: N/F = number of animals active or toxic over the number tested.
C= Comment code

Response Comments

Test	Dose (mg/kg)	Time	Code	Comments
TOX	75	0.25	23	Clonic seizures
TOX	100	0.25	23	Clonic seizures
TOX	100	0.25	5	Death following clonic seizure
TOX	100	0.50	19	Sedated
TOX	150	0.25	5	Death following clonic seizure
TOX	200	0.25	Z	All died approx 5 minutes post injection

Comments to Supplier:

KM-408

Anticonvulsant Screening Program
Test 23 Results - Sciatic Ligation Model In Rats

Add ID: 367061 B Screen ID: 1

Solvent Code: MC Solvent Prep: M&P,SB Route Code: IP

Date Started: 08-Dec-2011 Date Completed: 08-Dec-2011

Reference: SL4:43

Analysis

Dose (mg/kg)	Time (hrs)	Mean values +/- S.E.M		
		Threshold +/- S.E.M	% Pre-Drug +/- S.E.M	
6.0	0.0	2.88 +/- 0.59	100 +/- 21	☐
6.0	0.5	3.07 +/- 0.51	131 +/- 15	☐
6.0	1.0	5.94 +/- 1.19	202 +/- 19	☐
6.0	2.0	8.23 +/- 1.56	301 +/- 45	☑
6.0	4.0	7.09 +/- 1.31	260 +/- 39	☑
6.0	6.0	5.34 +/- 1.32	186 +/- 31	☐

* % threshold of ligated leg prior to drug administration.

Note: Box checked if data is significantly different from control.

Results of Analysis

Dose (mg/kg)	Time of Peak Effect (hrs)	* % Pre-Drug	
		Threshold +/- SEM	
6.0	2.0	301 +/- 45	☑

Note: Box checked if data is significantly different from pre-drug.

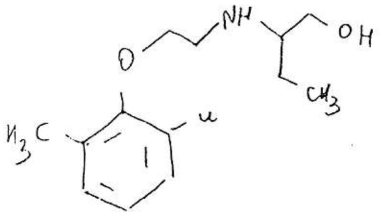

x HCl (R,S)

Anticonvulsant Screening Program
Test 23 Results - Sciatic Ligation Model In Rats

Add ID: 367061 B Screen ID: 1

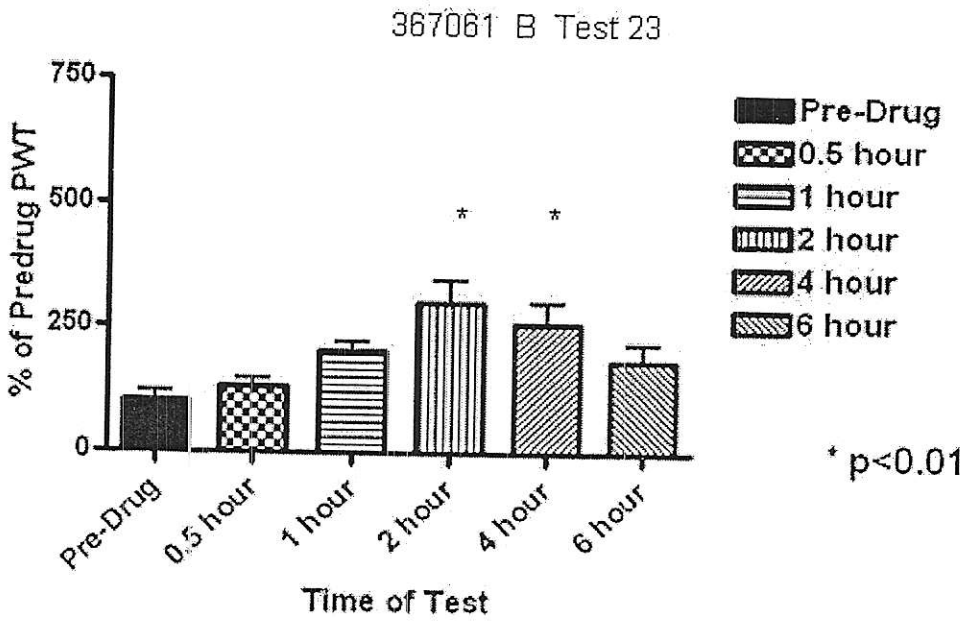

Response per Animal

		Threshold for Foot Withdrawal (grams)									
Dose (mg/kg)	Animal #	Pre-Drug	1hr	2hr	4hr	6hr	24hr	0.5hr			
6.0	1	6.73	11.96	11.96	11.96	11.96		11.96			
6.0	2	2.87	6.73	11.96	4.34	6.73		2.87			
6.0	3	2.87	6.73	59.96	11.96	9.29		4.34			
6.0	4	1.61	1.61	6.73	6.73	4.34		1.61			
6.0	5	1.61	2.87	2.87	2.87	2.87		1.61			
6.0	6	2.87	8.0	9.29	6.73	4.34		2.87			
6.0	7	1.61	2.87	2.87	2.87	1.61		2.87			
6.0	8	2.87	6.73	11.96	9.29	1.61		5.29			

Comment for Response: Animal 01 at 0.5 hr and animal 03 at 2 hr timepoints, are significant outliers as defined by Grubb's test ($p<0.05$) on Graphpad website(http://www.graphpad.com/quickcalcs/grubbs1.cfm)

Comments to Supplier:

12/15/2011 11:51:17 AM

KM-408

Anticonvulsant Screening Program

Test 76 Results - In-vitro Hippocampal Slice Culture Neuroprotection Assay (NP)

Add ID: 367061 C Screen ID: 1

Solvent Code:	DMSO	Solvent Prep:	
Date Started:	22-Feb-2012	Date Completed:	24-Feb-2012
Reference:	469: 243		

Summary of NP Assay: NMDA

- Test Result: No Neuroprotection

Comments to Supplier:

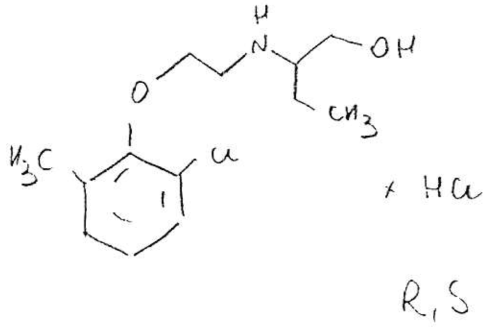

TEST 76: *in vitro* HIPPOCAMPAL SLICE CULTURE NEUROPROTECTION ASSAY

Compound 1 : ADD Number: 367061 Batch: C Date Started: 22-Feb-2012

Compound 2 : ADD Number: Batch: Date Completed: 24-Feb-2012

References: 469: 243

Excitotoxin: NMDA Insult Duration: 4 Hours Solvent: DMSO

Primary Screen Results: No neuroprotection observed

EXPERIMENT IMAGES & WELL DESCRIPTION

A1 NMDA 10μM

A2 NMDA 10μM + 367061 10μM

A3 NMDA 10μM + 367061 10μM

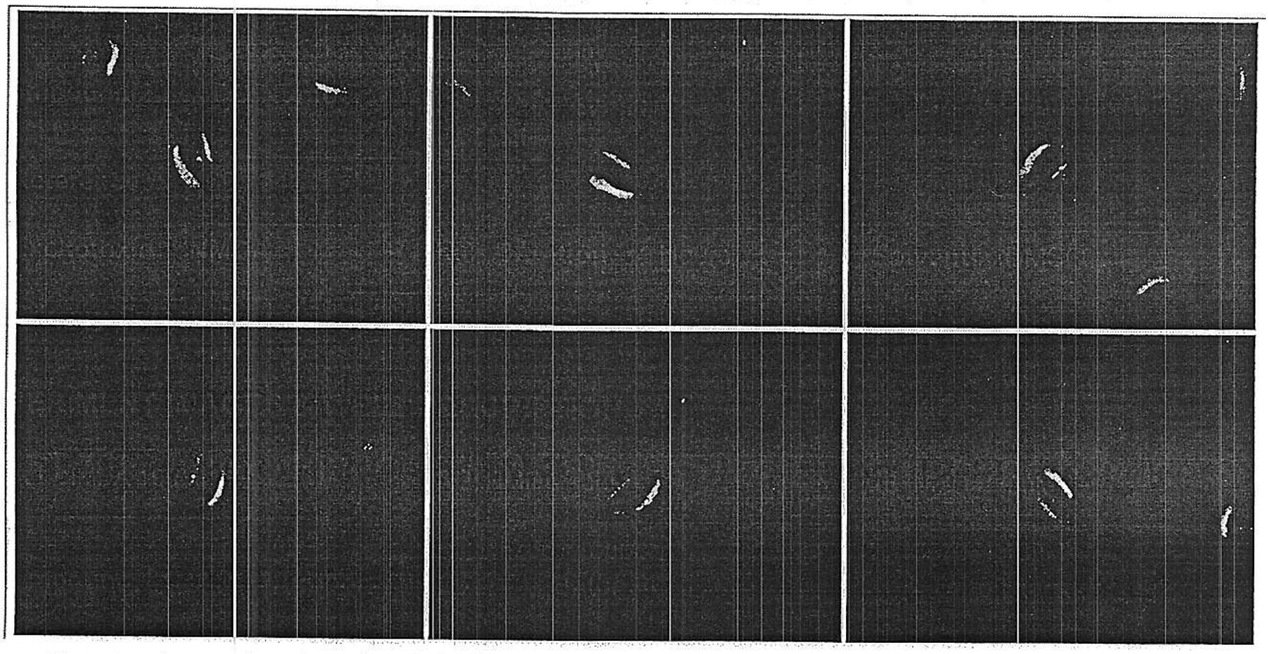

B1 NMDA 10μM

B2 NMDA 10μM + 367061 100μM

B3 NMDA 10μM + 367061 100μM

PRIMARY SCREEN EXPERIMENT DESCRIPTION

The "Primary Screen Experiment" is a qualitative assessment of the ability of a compound to prevent excitotoxic cell death. Organotypic hippocampal slice cultures are treated with N-methyl-D-aspartate (NMDA) or kainic acid (KA) to induce neuronal cell death. Propidium iodide (PI), a membrane-impermeant compound, is included in all wells of the culture plate. Dying cells have compromised cell membranes, thus PI may diffuse into the cell, intercalate with DNA and fluoresce. Thus, the intensity of the PI fluorescence is proportional to the amount of cell death in the individual slices. Hippocampal slice cultures are treated with the excitotoxin alone, or where indicated above, with the excitotoxin and either one or two investigational compounds at the concentrations indicated. If neuroprotection occurs as a consequence of the added compound, slice cultures will have a visibly reduced fluorescent intensity when compared to the slice cultures that have been treated with the excitotoxin alone.

KN-408

Anticonvulsant Screening Program

Test 76 Results - In-vitro Hippocampal Slice Culture Neuroprotection Assay (NP)

Add ID: 367061 C Screen ID: 2

Solvent Code: DMSO Solvent Prep:

Date Started: 22-Feb-2012 Date Completed: 24-Feb-2012

Reference: 469: 243

Summary of NP Assay: Kainic acid

- Test Result: No Neuroprotection

Comments to Supplier:

TEST 76: *in vitro* HIPPOCAMPAL SLICE CULTURE NEUROPROTECTION ASSAY

Compound 1 : ADD Number: 367061 Batch: C Date Started: 22-Feb-2012

Compound 2 : ADD Number: Batch: Date Completed: 24-Feb-2012

References: 469: 243

Excitotoxin: Kainic Acid Insult Duration: 4 Hours Solvent: DMSO

Primary Screen Results: No neuroprotection observed

EXPERIMENT IMAGES & WELL DESCRIPTION

A1 KA 20μM

A2 KA 20μM + 367061 10μM

A3 KA 20μM + 367061 10μM

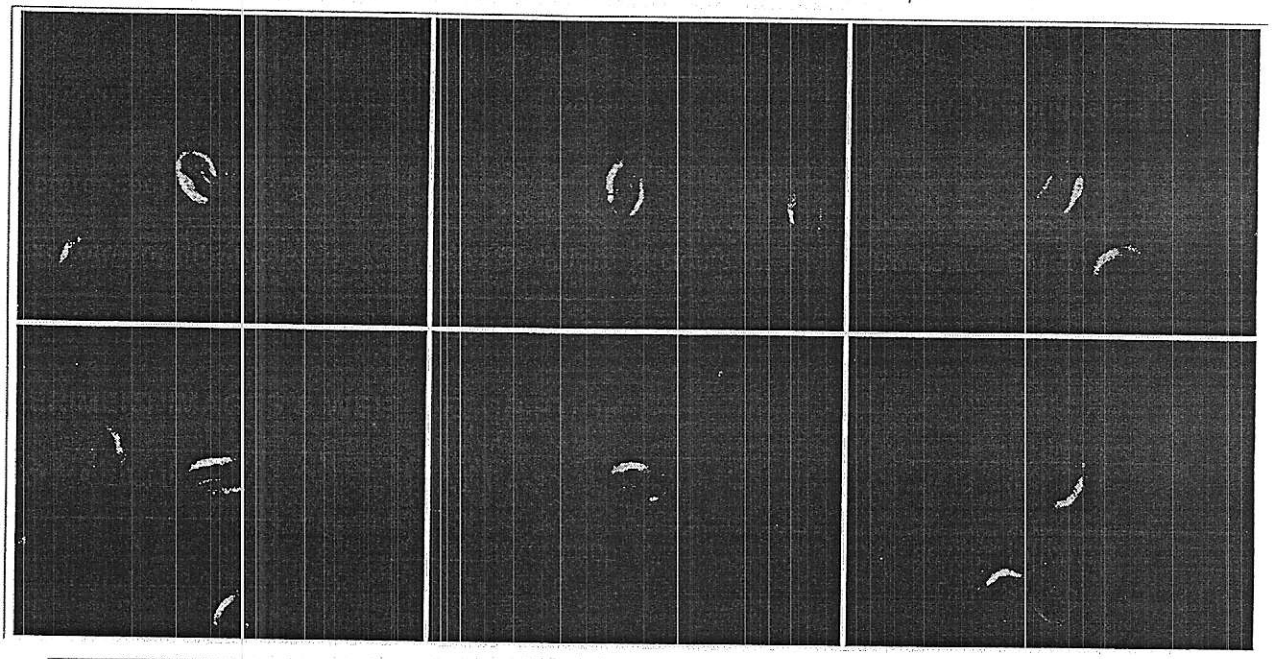

B1 KA 20μM

B2 KA 20μM + 367061 100μM

B3 KA 20μM + 367061 100μM

PRIMARY SCREEN EXPERIMENT DESCRIPTION

The "Primary Screen Experiment" is a qualitative assessment of the ability of a compound to prevent excitotoxic cell death. Organotypic hippocampal slice cultures are treated with N-methyl-D-aspartate (NMDA) or kainic acid (KA) to induce neuronal cell death. Propidium iodide (PI), a membrane-impermeant compound, is included in all wells of the culture plate. Dying cells have compromised cell membranes, thus PI may diffuse into the cell, intercalate with DNA and fluoresce. Thus, the intensity of the PI fluorescence is proportional to the amount of cell death in the individual slices. Hippocampal slice cultures are treated with the excitotoxin alone, or where indicated above, with the excitotoxin and either one or two investigational compounds at the concentrations indicated. If neuroprotection occurs as a consequence of the added compound, slice cultures will have a visibly reduced fluorescent intensity when compared to the slice cultures that have been treated with the excitotoxin alone.
