# Supplementary material for: KM-408, a novel phenoxyalkyl derivative as a potential anticonvulsant and analgesic compound for the treatment of neuropathic pain
Source: Pharmacol Rep. 2022 Nov 19;75(1):128–65. doi: 10.1007/s43440-022-00431-7 (PMC9889419; doi:10.1007/s43440-022-00431-7)
Supplement: Supplementary file 4 — Supplementary file4 (PDF 5760 KB) [file 43440_2022_431_MOESM4_ESM.pdf]

K.N-331

Anticonvulsant Screening Project

Test 1 Results - Mice I.P. Identification

Add ID: 354009 U **Screen ID:** 1

Solvent Code: MC **Solvent Prep:** M&P,SB

Animal Weight: 19.0 to 24.5 g

Date Started: 23-Jun-2004 **Date Completed** 23-Jun-2004

Reference: 383:74

Response

Time (Hours)				0.5		4.0		0.25		1.0		2.0		6.0		3.0		8.0		24	
Test	Dose	Form	Dth	N / F	C	N / F	C	N / F	C	N / F	C	N / F	C	N / F	C	N / F	C	N / F	C	N / F	C
MES	3	SUS		0 / 4		/		/		/		/		/		/		/		/	
[illegible]S	10	SUS		0 / 4		/		/		/		/		/		/		/		/	
MES	30	SUS		1 / 1		0 / 1		/		/		/		/		/		/		/	
MES	100	SUS		1 / 1		0 / 1		/		/		/		/		/		/		/	
SCMET	30	SUS		0 / 1		0 / 1		/		/		/		/		/		/		/	
TOX	3	SUS		0 / 4		/		/		/		/		/		/		/		/	
TOX	10	SUS		0 / 4		/		/		/		/		/		/		/		/	
TOX	30	SUS		0 / 4		0 / 2		/		/		/		/		/		/		/	
TOX	100	SUS	6	8 / 8	*	0 / 1		/		/		/		/		/		/		/	
TOX	300	SUS	4	4 / 4	1	/		/		/		/		/		/		/		/	

Response Comments

[illegible]T	DOSE (mg/kg	TIME	CODE	COMMENT
TOX	100	0.5	34	Muscle spasms
TOX	100	0.5	14	Unable to grasp rotorod
TOX	100	0.5	1	Death
TOX	300	0.5	1	Death

Comments to Supplier:

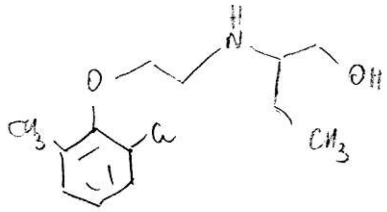

(R)

KM331

Anticonvulsant Screening Project

Test 8 Results -Anticonvulsant Identification (Rats I.P.)

Add ID: 354009 U **Screen ID:** 1

Solvent Code: MC Solvent Prep: M&P,SB

Animal Weight: 130 to 150 g

Date Started: 09-Nov-2006 Date Completed: 09-Nov-2006

Reference: 397:260

Time to Peak Effect

Test	Dose (mg/kg)	# Dths	0.25		0.5		1.0		2.0		4.0		6.0		8.0		24		3.0	
			N / F	C	N / F	C	N / F	C	N / F	C	N / F	C	N / F	C	N / F	C	N / F	C	N / F	C
ES	30		4 / 4		4 / 4		4 / 4		2 / 4		3 / 4		/		/		/		/	
TOX	30		0 / 4		0 / 4		0 / 4		0 / 4		0 / 4		/		/		/		/	

Comments to Supplier:

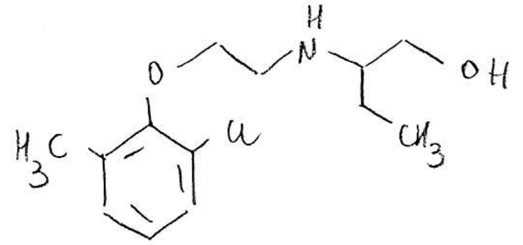

R(−)

U114-331

Anticonvulsant Screening Program

Test 8 Results - Anticonvulsant Identification (Rats I.P.)

Add ID: 354009 **Screen ID:** 1

Solvent Code: MC **Solvent Prep:** M&P,SB

Animal Weight: 130 - 150 g

Date Started: 09-Nov-2006 **Date Completed:** 09-Nov-2006

Reference: 397:260

Time to Peak Effect

Time (Hours)				0.25		0.5		1.0		2.0		4.0		6.0		3.0		8.0		24	
Test	Dos	Form	Dths	N/F	C	N/F	C	N/F	C	N/F	C	N/F	C	N/F	C	N/F	C	N/F	C	N/F	C
MES	30			4 / 4		4 / 4		4 / 4		2/ 4		3 / 4		/		/		/		/	
TOX	30			0 / 4		0 / 4		0 / 4		0/ 4		0 / 4		/		/		/		/	

Note: 1. N/F = number of animals active or toxic over the number tested.

2. C= Comment code

Comments to Supplier:

KM331

Anticonvulsant Screening Project

Test 8 Results -Anticonvulsant Identification (Rats I.P.)

Add ID: 354009 U Screen ID: 1

Solvent Code: MC Solvent Prep: M&P,SB

Animal Weight: 130 to 150 g

(22)

Date Started: 09-Nov-2006 Date Completed: 09-Nov-2006

Reference: 397:260

Time to Peak Effect

Test	Dose (mg/kg)	# Dths	0.25		0.5		1.0		2.0		4.0		6.0		8.0		24		3.0	
			N / F	C	N / F	C	N / F	C	N / F	C	N / F	C	N / F	C	N / F	C	N / F	C	N / F	C
MES	30		4 / 4		4 / 4		4 / 4		2 / 4		3 / 4		/		/		/		/	
X	30		0 / 4		0 / 4		0 / 4		0 / 4		0 / 4		/		/		/		/	

Comments to Supplier:

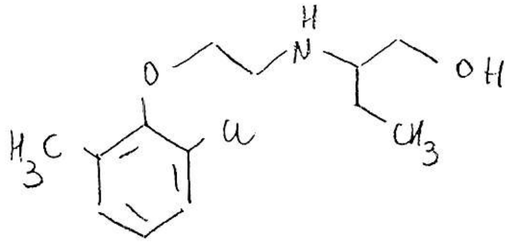

R(−)

KM 331

Anticonvulsant Screening Program
Test 10 Results - Anticonvulsant Quantification (Rats I.P.)

Add ID: 354009 A **Screen ID:** 1

Solvent Code:	MC	Solvent Prep:	M&P,SB	Route Code:	IP
Animal Weight:	- g				
Date Started:	20-Mar-2007	Date Completed:	22-Mar-2007		
Reference:	409:88-91				

ED50 Value

Test	Time(Hrs)	ED50	95% Confidence Interval	Slope	STD Err	PI Value
MES	0.25	4.65	2.94 - 6.88	3.67	1.1	
TOX	0.25	> 30.0	0.0 - 0.0			

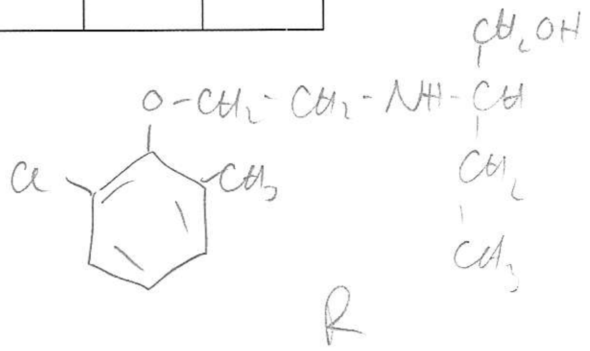

ED50 Biological Response

Test	Time (hr)	Dose (mg/kg)	Dths	N / F	C
MES	0.25	2.0		1 / 8	
MES	0.25	4.0		3 / 8	
MES	0.25	7.5		6 / 8	
MES	0.25	15.0		7 / 7	

Time to Peak Effect

Time (Hours)			0.25		0.5		1.0		2.0		4.0		6.0		8.0		24		3.0	
Test	Dose	Dths	N / F	C	N / F	C	N / F	C	N / F	C	N / F	C	N / F	C	N / F	C	N / F	C	N / F	C
MES	15.0		4 / 4		3 / 4		2 / 4		/		/		/		/		/		/	
TOX	30.0		0 / 4	Z	0 / 4		0 / 4		0 / 4		/		/		/		/		/	
TOX	125.0	1	1 / 2	1	1 / 2		1 / 2		1 / 2		/		/		/		/		/	
TOX	125.00 01	7	7 / 8	Z	7 / 8		7 / 8		7 / 8		/		/		/		/		/	
TOX	250.0	1	1 / 2	1	1 / 2		1 / 2		1 / 2		/		/		/		/		/	
TOX	500.0	1	1 / 2	1	1 / 2		1 / 2		1 / 2		/		/		/		/		/	

Note: N/F = number of animals active or toxic over the number tested.

C= Comment code

Response Comments

Test	Dose (mg/kg)	Time	Code	Comments

Anticonvulsant Screening Program

Test 10 Results - Anticonvulsant Quantification (Rats I.P.)

Add ID: 354009 A Screen ID: 1

TOX	30	0.25	Z	from Test 8 results
TOX	125.0001	0.25	Z	7 animals died within 10 mins of inject, remaining animal was NOT toxic.
TOX	250	0.25	1	Death
TOX	500	0.25	1	Death

Comments to Supplier:

KM-331

Anticonvulsant Screening Project

Test 10 Results -Anticonvulsant Quantification (Rats I.P.)

Add ID: 354009 A **Screen ID:** 1

Solvent Code: MC **Solvent Prep:** M&P,SB **Route Code:** IP

Animal Weight: to g

Date Started: 20-Mar-2007 **Date Completed:** 22-Mar-2007

Reference: 409:88-91

ED50 Values

Test	Time (Hrs)	ED50	95% Confidence Interval LOW	95% Confidence Interval HIGH	SLOPE	STD. ERR.
MES	0.25	4.65	2.94	6.88	3.67	1.1
[illegible]X	0.25	> 30	0	0		

ED50 Biological Response

Test	Dose (mg/kg)	Dths	N / F	C
MES	2		1 / 8	
MES	4		3 / 8	
MES	7.5		6 / 8	
MES	15		7 / 7	

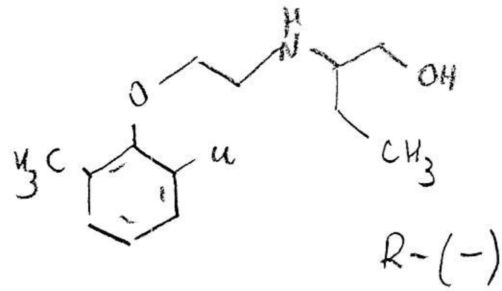

R-(-)

Time to Peak Effect

Test	Dose (mg/kg)	# Dths	0.25 N / F	0.25 C	0.5 N / F	0.5 C	1.0 N / F	1.0 C	2.0 N / F	2.0 C	4.0 N / F	4.0 C	6.0 N / F	6.0 C	8.0 N / F	8.0 C	24 N / F	24 C	3.0 N / F	3.0 C
[illegible]S	15		4 / 4		3 / 4		2 / 4		/		/		/		/		/		/	
TOX	30		0 / 4	Z	0 / 4		0 / 4		0 / 4		/		/		/		/		/	
TOX	125	1	1 / 2	1	1 / 2		1 / 2		1 / 2		/		/		/		/		/	
TOX	125.00	7	7 / 8	Z	7 / 8		7 / 8		7 / 8		/		/		/		/		/	
TOX	250	1	1 / 2	1	1 / 2		1 / 2		1 / 2		/		/		/		/		/	
TOX	500	1	1 / 2	1	1 / 2		1 / 2		1 / 2		/		/		/		/		/	

Response Comments

TEST	DOSE (mg/kg)	TIME	CODE	COMMENT
TOX	30	0.25	Z	from Test 8 results
TOX	125	0.25	Z	7 animals died within 10 mins of inject, remaining animal was NOT toxic.
TOX	250	0.25	1	Death

Anticonvulsant Screening Project

Test 10 Results -Anticonvulsant Quantification (Rats I.P.)

Add ID: 354009 A Screen ID: 1

TOX	500	0.25	1	Death

Comments to Supplier:

KM331

Anticonvulsant Screening Program

Test 25 Results - LTG-Resistant Amygdala Kindled Rat

Add ID: 354009 A **Screen ID:** 1

Solvent Code: MC **Solvent Prep:** M&P,SB **Route Code:** IP

Date Started: 31-May-2007 **Date Completed:** 08-Jun-2007

Reference: AS2:176

Results

Dose (mg/kg)	Time of Test (hrs)	Seizure Score	±	S.E.M.		Duration (sec)	±	S.D		# Prot	/	# Tested
Control	0.25	5.0	±		☐	61	±	14	☐	0	/	5
15	0.25	4.0	±	1.0	☐	33	±	19	☐	0	/	4
Control			±		☐		±		☐	0	/	
30	0.25	3.5	±	0.5	☐	41	±	10	☐	1	/	4
Control			±		☐		±		☐	0	/	
50	0.25	2.2	±	0.7	☑	24	±	26	☑	3	/	5

Note: Box checked if data is significantly different from control.

Comments to Supplier: At 30 mg/kg, 3/4 rats were toxic - ataxia, mildly sedated. At 50 mg/kg, 5/5 rats were toxi

KM331

ANTICONVULSANT SCREENING PROJECT TEST RESULTS
COUNTERMEASURES

Test 71: Prevention of Pilocarpine-induced Status, Rats

ADD Number: 354009
Solvent: MC Prep: M&P,SB
Reference: 411:115-116,132-140

Date Started: 07-JUN-2007
Date Completed: 14-JUN-2007
Route: i.p.

Toxicity Test

Dose (mg/kg)	Time in Hours				
	.25	.5	1	2	4
100	2/2 (2 died)				
50	2/2	2/2	0/2	0/2	0/2
25	0/2	0/2	0/2	0/2	0/2

PiSE TPE Determination

Dose (mg/kg)	Time of Test (hrs)*	Prot./Tested	Comment	# Died	Ave. wght loss ± S.E.M.
25	0	2/8	1/8 was still seizing next day	5	8.3 ± 7.1
					±
					±
					±

*post first Stage III seizure

Dose Response Data

PiSE Time of Test: hrs

mg/kg					
Pro./Test.					

Toxicity Time of Test: hrs

mg/kg					
Tox./Test.					

ED50 Values

TPE (hrs)		mg/kg	95% C.I.	Slope	± S.E.M.
	**TD50**				
	**ED50**				
	**ED97**				

Comment for NIH: No further testing recommended at this point.

KM331

Anticonvulsant Screening Project

Test 11 Results - Preliminary Hippocampal Kindling Screen - Rats IP

Add ID: 354009 U **Screen ID:** 1

PD 27

Solvent Code: MC **Solvent Prep:** M&P,SB **Route Code:** IP

Animal Weight: to g

Date Started: 30-Jul-2007 **Date Completed:** 30-Jul-2007

Reference: 418:43-47

Dose: 30 mg/kg Time of Maximum Effect: 45 to min

Rat #	Comment Code	Seizure Score Pre-Drug Low	Seizure Score Pre-Drug High	Seizure Score Drug Low	Seizure Score Drug High	Afterdischarge Duration (secs) Pre-Drug Low	Afterdischarge Duration (secs) Pre-Drug High	Afterdischarge Duration (secs) Drug Low	Afterdischarge Duration (secs) Drug High
1		5 -		4 -		13 -	51	63 -	
		5 -		0 -		18 -	55	42 -	

Comments to Supplier:

Anticonvulsant Screening Program

Test 2 Results - Rat P.O. Identification

Add ID: 354009 A Screen ID: 1

**Solvent Code:**	MC	**Solvent Prep:**	M&P,SB
**Animal Weight:**	120.0 - 150.0 g		
**Date Started:**	21-Jan-2009	**Date Completed:**	21-Jan-2009
**Reference:**	433:114		

Response

Time (Hours)				0.25		0.5		1.0		2.0		4.0		6.0		8.0		24		3.0	
Test	Dose		Dths	N / F	C	N / F	C	N / F	C	N / F	C	N / F	C	N / F	C	N / F	C	N / F	C	N / F	C
MES	30			0 / 4		1 / 4		0 / 4		0 / 4		0 / 4		/		/		/		/	
TOX	30			0 / 4		0 / 4		0 / 4		0 / 4		0 / 4		/		/		/		/	

Note: N/F = number of animals active or toxic over the number tested.

C= Comment code

Comments to Supplier:

KM-331

Anticonvulsant Screening Program
Test 4 Results - Mice I.P. Quantification

Add ID: 354009 U Screen ID: 1

Solvent Code:	MC	Solvent Prep:	M&P,SB
Animal Weight:	- g		
Date Started:	05-Mar-2009	Date Completed:	10-Mar-2009
Reference:	437:240-245		

ED50 Value

Test	Time(Hrs)	ED50	95% Confidence Interval	Slope	STD Err	PI Value
MES	0.25	18.2	16.6 - 20	18.3	5.9	
SCMET	0.25	> 70	-			
TOX	0.25	59.8	43.8 - 74.2	5.8	1.7	

ED50 Biological Response

Test	Dose (mg/kg)	Dths	N / F	C
MES	15		1 / 8	
MES	18		2 / 8	
MES	20		7 / 8	
MES	25		8 / 8	
SCMET	70		0 / 8	*
TOX	25		0 / 8	
TOX	50		2 / 8	
TOX	70		8 / 8	*
TOX	90	4	5 / 8	*
TOX	150	8	8 / 8	1

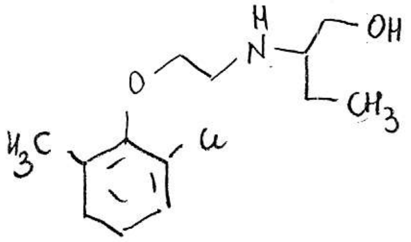

R-(-)

ED50 Biological Response Comments

Test	Dose (mg/kg)	Time	Code	Comment
SCMET	70	0.25	15	Minimal motor impairment
SCMET	70	0.25	22	Continuous seizure activity
SCMET	70	0.25	3	Death following continuous seizure
TOX	70	0.25	14	Unable to grasp rotorod
TOX	70	0.25	34	Muscle spasms

KM-331

Anticonvulsant Screening Program

Test 4 Results - Mice I.P. Quantification

Add ID: 354009 U **Screen ID: 1**

TOX	90	0.25	1	Death
TOX	90	0.25	14	Unable to grasp rotorod
TOX	150	0.25	1	Death

Time to Peak Effect

Time (Hours)			0.25		0.5		1.0		2.0		4.0		6.0		8.0		24		3.0	
Test	Dose	Dths	N / F	C	N / F	C	N / F	C	N / F	C	N / F	C	N / F	C	N / F	C	N / F	C	N / F	C
MES	30		4 / 4		3 / 4		0 / 4		0 / 4		0 / 4		/		/		/		/	
TOX	50		2 / 8		0 / 8		0 / 8		0 / 8		0 / 8		/		/		/		/	

Note: N/F = number of animals active or toxic over the number tested.

C= Comment code

Comments to Supplier:

KM-331

Anticonvulsant Screening Program

Test 7 Results - Anticonvulsant Evaluation (6Hz, Mice)

Add ID: 354009 A Screen ID: 1

Solvent Code:	MC	Solvent Prep:	M&P,SB	Route Code:	IP
Animal Weight:	- g			Current(mA):	32
Date Started:	04-May-2009	Date Completed:	04-May-2009		
Reference:	440:58-59				

Time to Peak Effect

Time (Hours)			0.25		0.5		1.0		2.0		4.0		6.0		8.0		24		3.0	
Test	Dose	Dths	N / F	C	N / F	C	N / F	C	N / F	C	N / F	C	N / F	C	N / F	C	N / F	C	N / F	C
6HZ	25		1 / 4		0 / 4		/		/		/		/		/		/		/	
6HZ	50		4 / 4		4 / 4		0 / 4		0 / 4		0 / 4		/		/		/		/	

Note: N/F = number of animals active or toxic over the number tested.

C= Comment code

Comments to Supplier:

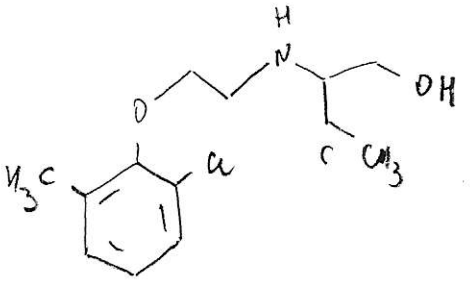

R (-)

KM-331

Anticonvulsant Screening Program

Test 15 Results - IV Metrazol

Add ID: 354009 A Screen ID: 1

Solvent Code:	MC	Solvent Prep:	M&P,SB	Route Code:	IP
Time of Test:	0.25 (hrs)	Infusion Rate:	0.34 (ml/min)		
MES ED50:	18.00 (mg/kg)	TD50:	60.00 (mg/kg)		
Date Started:	24-Jun-2009	Date Completed:	24-Jun-2009		
Reference:	428: 214-215				

Anlaysis

Dose (mg/kg)		Weight (grams)	Time to Twitch	Twitch (mg/kg)	Time to Clonus	Clonus (mg/kg)
0	Mean	36.75	30.1	23.3	33.1	25.6
	Std. Err	1.02	0.73	0.60	0.85	0.65
	P-value					
18	Mean	37.30	28.8	22.0	32.6	25.0
	Std. Err	0.90	1.39	1.27	1.64	1.53
	P-value	0.345	0.201	0.182	0.405	0.360
60	Mean	37.45	26.1	19.8	30.4	23.0
	Std. Err	0.49	1.68	1.28	1.38	1.08
	P-value	0.273	0.024	0.013	0.061	0.031

Response

Dose (mg/kg)	Animal #	Weight (grams)	Time to Twitch	Twitch (mg/kg)	Time to Clonus	Clonus (mg/kg)
0	01	33.0	27.50	23.61	28.50	24.47
0	02	39.0	32.00	23.25	36.00	26.15
0	03	37.5	28.50	21.53	33.00	24.93
0	04	34.5	29.00	23.82	30.00	24.64
0	05	37.0	28.00	21.44	35.50	27.18
0	06	40.0	31.50	22.31	34.00	24.08
0	07	37.0	34.50	26.42	36.00	27.57
0	08	42.5	32.00	21.33	34.00	22.67
0	09	35.0	28.00	22.67	30.00	24.29
0	10	32.0	30.00	26.56	33.50	29.66
18	01	34.0	24.50	20.42	34.00	28.33
18	02	41.5	31.50	21.51	38.00	25.94
18	03	40.5	26.00	18.19	28.00	19.59

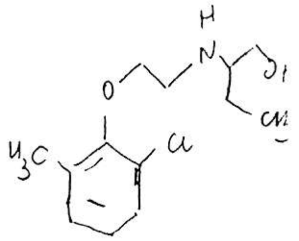

R-(-)

KN-331

Anticonvulsant Screening Program

Test 15 Results - IV Metrazol

Add ID: 354009 A Screen ID: 1

18	04	40.5	31.00	21.69	34.50	24.14
18	05	38.0	25.00	18.64	26.50	19.76
18	06	38.5	27.00	19.87	28.50	20.97
18	07	36.0	22.00	17.31	25.00	19.68
18	08	34.5	35.00	28.74	40.00	32.85
18	09	34.5	32.00	26.28	35.00	28.74
18	10	35.0	33.50	27.12	36.50	29.55
60	01	37.0	33.00	25.27	34.50	26.42
60	02	36.5	26.00	20.18	29.00	22.51
60	03	38.0	19.50	14.54	22.00	16.40
60	04	35.0	26.00	21.05	30.50	24.69
60	05	40.0	33.50	23.73	34.50	24.44
60	06	37.0	31.00	23.74	35.00	26.80
60	07	40.0	21.50	15.23	30.50	21.60
60	08	37.0	19.50	14.93	26.50	20.29
60	09	36.5	28.50	22.12	34.50	26.78
60	10	37.5	22.50	17.00	27.00	20.40

Comments to Supplier:

KM-331

Anticonvulsant Screening Program

Test 7 Results - Anticonvulsant Evaluation (6Hz, Mice)

Add ID: 354009 A Screen ID: 2

Solvent Code: MC
Solvent Prep: M&P,SB
Route Code: IP
Animal Weight: - g
Current(mA): 32
Date Started: 04-Aug-2009
Date Completed: 04-Aug-2009
Reference: 440:269-270

ED50 Value

Test	Time(Hrs)	ED50	95% Confidence Interval	Slope	STD Err	PI Value
6HZ	0.25	33.1	29 - 37.8	15.3	6.1	

ED50 Biological Response

Test	Dose (mg/kg)	Dths	N / F C
6HZ	19		0 / 8
6HZ	29		2 / 8
6HZ	33		3 / 8
6HZ	38		7 / 8

Comments to Supplier:

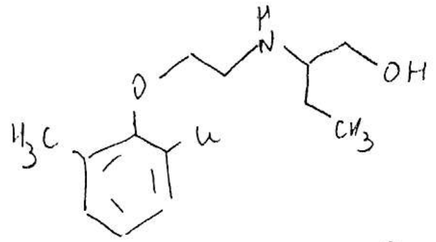

R-(-)

KM-331

Anticonvulsant Screening Program

Test 22 Results - Formalin Test (Mice I.P.)

Add ID: 354009 A **Screen ID:** 1

Solvent Code:	MC	Solvent Prep:	M&P,SB	Route Code:	IP
Time of Test:	0.25 (hrs)				
ED50:	18.24 (mg/kg)	TD50:	59.84 (mg/kg)		
Date Started:	02-Oct-2009	Date Completed:	14-Oct-2009		
Reference:	F4:36				

Analysis

		Area Under the Curve				
Dose (mg/kg)	Test	Control	Drug Treated	% of Control	S.E.M	p Value
20.0	Acute	197.2	155.2	78.68	8.48	> 0.05
20.0	Inflammatory	470.6	496.7	105.6	13.24	> 0.05

Response

Trial 1

		Duration of Licking (sec)											
Dose (mg/kg)	Animal #	0 min	5 min	10 min	15 min	20 min	25 min	30 min	35 min	40 min	45 min	50 min	55 min
0.0	01	35.41	0.00	0.00	24.43	0.00	54.41	0.00	57.05	20.68			
0.0	02	58.14	14.21	0.00	5.59	0.00	52.70	32.45	8.09	29.81			
0.0	03	37.17	7.93	0.00	1.87	23.70	13.62	15.64	12.23	0.00			
0.0	04	46.45	21.23	0.00	0.00	26.54	15.59	25.12	14.52	0.00			
0.0	05	51.54	10.82	0.00	3.34	22.08	34.12	29.47	5.56	23.44			
0.0	06	54.63	29.37	15.53	1.17	19.24	19.90	35.45	3.59	5.12			
0.0	07	36.63	0.00	18.42	31.36	20.99	23.60	30.10	0.00	0.00			
0.0	08	41.79	34.03	0.00	19.89	16.31	45.53	0.00	9.69	4.68			

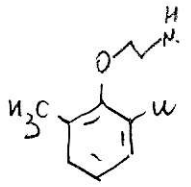

R-

Trial 1

		Duration of Licking (sec)											
Dose (mg/kg)	Animal #	0 min	5 min	10 min	15 min	20 min	25 min	30 min	35 min	40 min	45 min	50 min	55 min
20.0	01	42.92	0.00	2.59	14.46	16.24	17.40	19.81	48.61	55.63			
20.0	02	24.32	22.28	0.00	1.95	0.00	30.70	0.00	0.00	6.87			
20.0	03	48.53	0.00	4.80	0.00	13.53	15.97	25.80	0.00	29.66			

KM-331

Anticonvulsant Screening Program

Test 22 Results - Formalin Test (Mice I.P.)

Add ID: 354009 A Screen ID: 1

**20.0**	04	47.01	0.00	0.00	0.00	31.64	20.52	53.37	26.44	10.42				
**20.0**	05	46.91	13.75	0.00	4.73	28.65	36.03	8.74	46.12	10.97				
**20.0**	06	52.23	17.69	7.84	0.00	15.38	15.46	37.68	18.98	24.04				
**20.0**	07	52.54	9.92	0.00	37.79	8.65	0.00	50.13	0.00	29.59				
**20.0**	08	39.51	0.00	0.00	28.36	30.51	12.86	0.00	35.40	5.66				

Comments to Supplier:

KM-331

Anticonvulsant Screening Program

Test 76 Results - In-vitro Hippocampal Slice Culture Neuroprotection Assay (NP)

Add ID: 354009 A Screen ID: 2

Solvent Code: DMSO Solvent Prep:

Date Started: 11-Nov-2009 Date Completed: 20-Nov-2009

Reference: 439:251,259

Summary of NP Assay: Kainic acid

- Test Result: No Neuroprotection

Comments to Supplier:

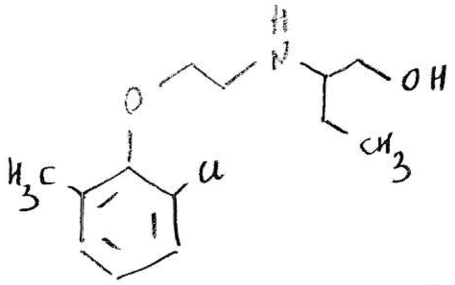

R (-)

TEST 76: *in vitro* HIPPOCAMPAL SLICE CULTURE NEUROPROTECTION ASSAY

Compound 1 : ADD Number: 354009 **Batch:** A **Date Started:** 11-Nov-2009

Compound 2 : ADD Number: **Batch:** **Date Completed:** 20-Nov-2009

References: 439: 251, 259

Excitotoxin: Kainic Acid **Insult Duration:** 4 Hours **Solvent:** DMSO

Primary Screen Results: **No neuroprotection observed**

EXPERIMENT IMAGES & WELL DESCRIPTION

A1 KA 20μM

A2 KA 20μM + 354009 10μM

A3 KA 20μM + 354009 10μM

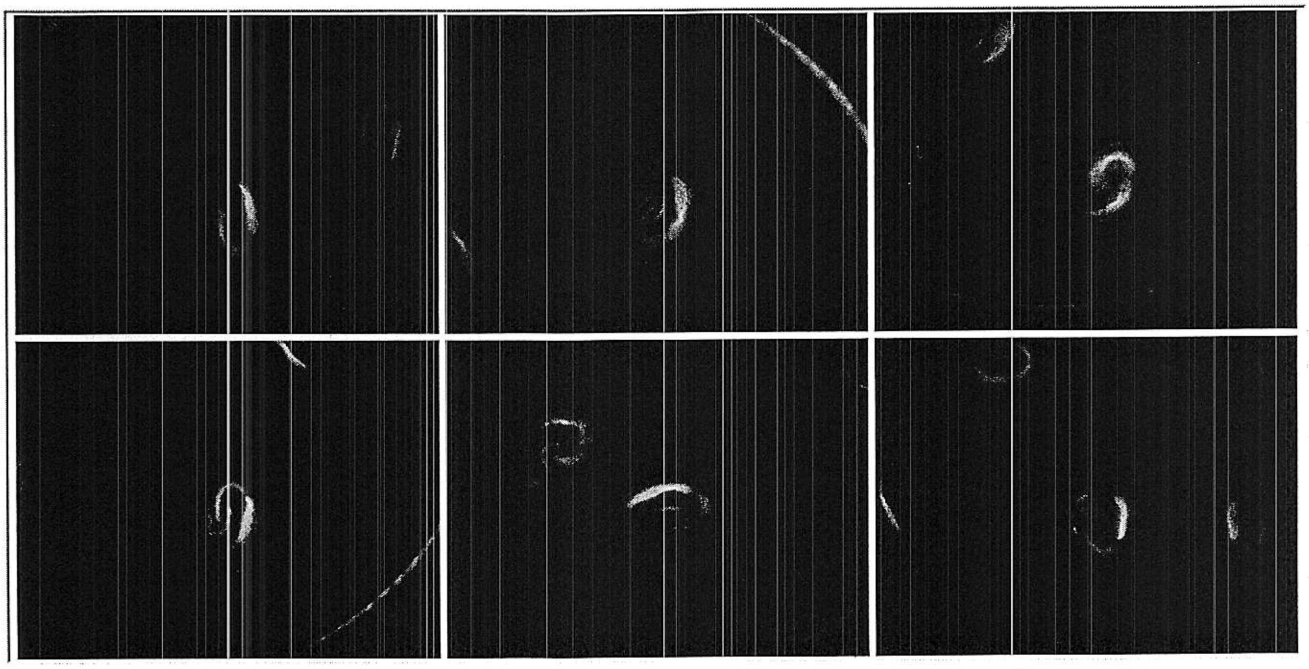

B1 KA 20μM

B2 KA 20μM + 354009 100μM

B3 KA 20μM + 354009 100μM

PRIMARY SCREEN EXPERIMENT DESCRIPTION

The "Primary Screen Experiment" is a qualitative assessment of the ability of a compound to prevent excitotoxic cell death. Organotypic hippocampal slice cultures are treated with N-methyl-D-aspartate (NMDA) or kainic acid (KA) to induce neuronal cell death. Propidium iodide (PI), a membrane-impermeant compound, is included in all wells of the culture plate. Dying cells have compromised cell membranes, thus PI may diffuse into the cell, intercalate with DNA and fluoresce. Thus, the intensity of the PI fluorescence is proportional to the amount of cell death in the individual slices. Hippocampal slice cultures are treated with the excitotoxin alone, or where indicated above, with the excitotoxin and either <u>**one or two**</u> investigational compounds at the concentrations indicated. If neuroprotection occurs as a consequence of the added compound, slice cultures will have a visibly reduced fluorescent intensity when compared to the slice cultures that have been treated with the excitotoxin alone.

Anticonvulsant Screening Program

Test 76 Results - In-vitro Hippocampal Slice Culture Neuroprotection Assay (NP)

Add ID: 354009 A Screen ID: 1

Solvent Code:	DMSO	Solvent Prep:	
Date Started:	11-Nov-2009	Date Completed:	20-Nov-2009
Reference:	439:251,259		

Summary of NP Assay: NMDA

- Test Result: No Neuroprotection

Comments to Supplier:

TEST 76: *in vitro* HIPPOCAMPAL SLICE CULTURE NEUROPROTECTION ASSAY

Compound 1 : **ADD Number:** 354009 **Batch:** A **Date Started:** 11-Nov-2009

Compound 2 : **ADD Number:** **Batch:** **Date Completed:** 20-Nov-2009

References: 439: 251, 259

Excitotoxin: **NMDA** **Insult Duration:** 4 Hours **Solvent:** DMSO

Primary Screen Results: **No neuroprotection observed**

EXPERIMENT IMAGES & WELL DESCRIPTION

A1 NMDA 10µM

A2 NMDA 10µM + 354009 10µM

A3 NMDA 10µM + 354009 10µM

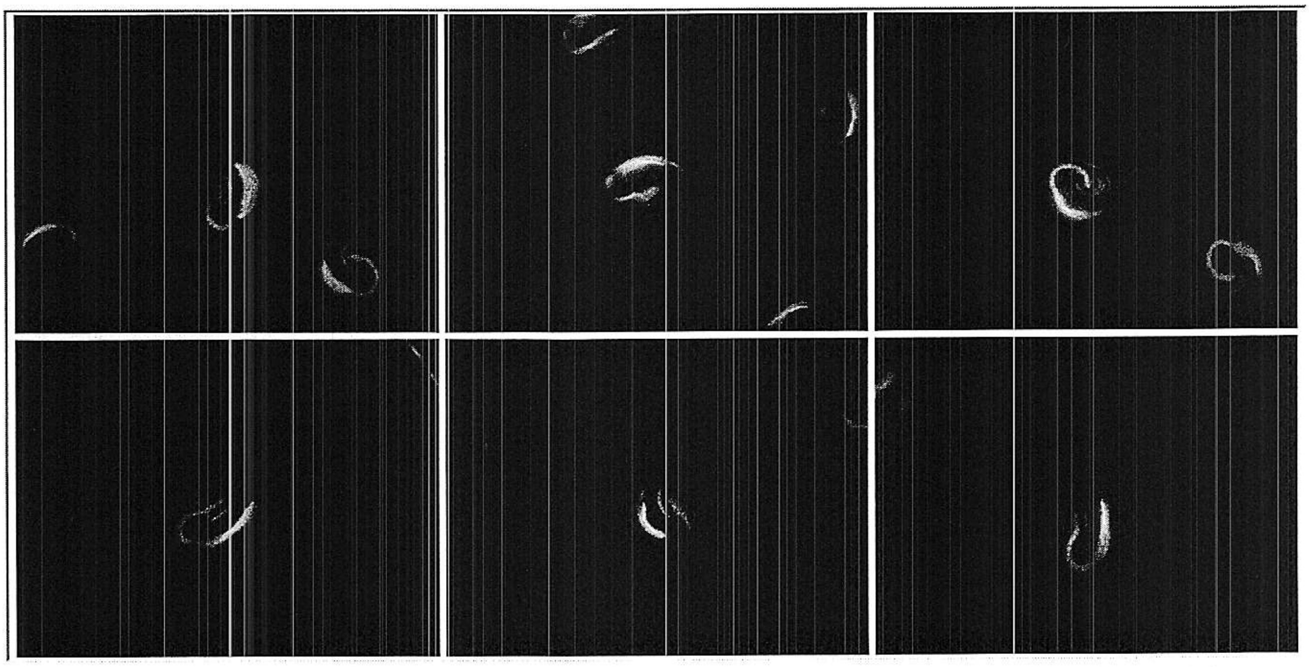

B1 NMDA 10µM

B2 NMDA 10µM + 354009 100µM

B3 NMDA 10µM + 354009 100µM

PRIMARY SCREEN EXPERIMENT DESCRIPTION

The "Primary Screen Experiment" is a qualitative assessment of the ability of a compound to prevent excitotoxic cell death. Organotypic hippocampal slice cultures are treated with N-methyl-D-aspartate (NMDA) or kainic acid (KA) to induce neuronal cell death. Propidium iodide (PI), a membrane-impermeant compound, is included in all wells of the culture plate. Dying cells have compromised cell membranes, thus PI may diffuse into the cell, intercalate with DNA and fluoresce. Thus, the intensity of the PI fluorescence is proportional to the amount of cell death in the individual slices. Hippocampal slice cultures are treated with the excitotoxin alone, or where indicated above, with the excitotoxin and either **one or two** investigational compounds at the concentrations indicated. If neuroprotection occurs as a consequence of the added compound, slice cultures will have a visibly reduced fluorescent intensity when compared to the slice cultures that have been treated with the excitotoxin alone.
