# Supplementary material for: KM-408, a novel phenoxyalkyl derivative as a potential anticonvulsant and analgesic compound for the treatment of neuropathic pain
Source: Pharmacol Rep. 2022 Nov 19;75(1):128–65. doi: 10.1007/s43440-022-00431-7 (PMC9889419; doi:10.1007/s43440-022-00431-7)

KM-526

Anticonvulsant Screening Program

Test 1 Results - Mice I.P. Identification

Add ID: 400031 U Screen ID: 1

Solvent Code: MC Solvent Prep: M&P,SB

Animal Weight: 20.5 - 25.5 g

Date Started: 05-May-2008 Date Completed: 05-May-2008

Reference: 427:205

Response

Time (Hours)				0.5		4.0		0.25		1.0		2.0		6.0		3.0		8.0		24	
Test	Dose	Form	Dths	N / F	C	N / F	C	N / F	C	N / F	C	N / F	C	N / F	C	N / F	C	N / F	C	N / F	C
MES	3	SOL		0 / 4		/		/		/		/		/		/		/		/	
MES	10	SOL		0 / 4		/		/		/		/		/		/		/		/	
MES	30	SOL		1 / 1		0 / 1		/		/		/		/		/		/		/	
MES	100	SOL		2 / 3		0 / 2		/		/		/		/		/		/		/	
SCMET	30	SOL		0 / 1		0 / 1		/		/		/		/		/		/		/	
SCMET	100	SOL		0 / 1		0 / 1		/		/		/		/		/		/		/	
TOX	3	SOL		0 / 4		/		/		/		/		/		/		/		/	
TOX	10	SOL		0 / 4		/		/		/		/		/		/		/		/	
TOX	30	SOL		0 / 4		0 / 2		/		/		/		/		/		/		/	
TOX	100	SOL	1	7 / 8	*	0 / 3		/		/		/		/		/		/		/	
TOX	300	SOL	4	4 / 4	1	/		/		/		/		/		/		/		/	

[illegible]e: N/F = number of animals active or toxic over the number tested.

C= Comment code

Response Comments

Test	Dose(mg/kg)	Time	Code	Comments
TOX	100	0.5	1	Death
TOX	100	0.5	14	Unable to grasp rotorod
TOX	300	0.5	1	Death

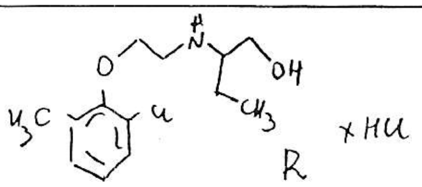

Anticonvulsant Screening Program

Test 1 Results - Mice I.P. Identification

Add ID: 400031	U	Screen ID: 1

Comments to Supplier:

KM 526

Anticonvulsant Screening Program

Test 2 Results - Rat P.O. Identification

Add ID: 400031 U Screen ID: 1

**Solvent Code:**	MC	**Solvent Prep:**	M&P,SB
**Animal Weight:**	105.0 - 140.0 g		
**Date Started:**	27-Jun-2008	**Date Completed:**	27-Jun-2008
**Reference:**	429:137		

Response

Time (Hours)				0.25		0.5		1.0		2.0		4.0		6.0		8.0		24		3.0	
Test	Dose	Form	Dths	N / F	C	N / F	C	N / F	C	N / F	C	N / F	C	N / F	C	N / F	C	N / F	C	N / F	C
MES	30			0 / 4		0 / 4		0 / 4		0 / 4		0 / 4		/		/		/		/	
TOX	30			0 / 4		0 / 4		0 / 4		0 / 4		0 / 4		/		/		/		/	

Note: N/F = number of animals active or toxic over the number tested.

C= Comment code

Comments to Supplier:

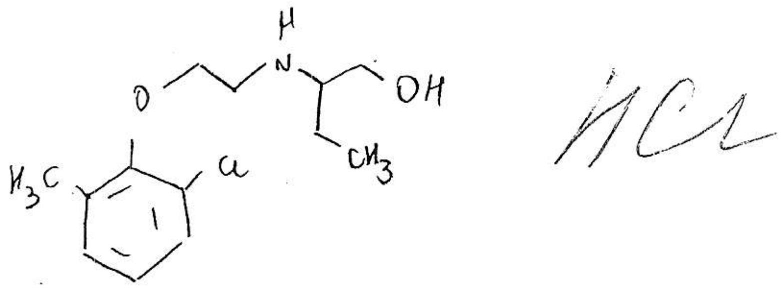

R

KM-526

Anticonvulsant Screening Program
Test 4 Results - Mice I.P. Quantification

Add ID: 400031 U Screen ID: 1

Solvent Code: MC Solvent Prep: TT,SB

Animal Weight: - g

Date Started: 04-Aug-2008 Date Completed: 06-Aug-2008

Reference: 431: 65-70

ED50 Value

Test	Time(Hrs)	ED50	95% Confidence Interval	Slope	STD Err	PI Value
MES	0.25	19.78	16.16 - 22.33	8.63	2.66	
SCMET	0.25	> 70.00	0.00 - 0.00			
TOX	0.25	54.01	35.70 - 64.23	10.48	4.30	

ED50 Biological Response

Test	Dose (mg/kg)	Dths	N / F	C
MES	13		1 / 8	
MES	19		2 / 8	
MES	22		5 / 8	
MES	25		14 / 16	
SCMET	70		0 / 8	3
TOX	25		0 / 8	
TOX	50		3 / 8	14
TOX	70		7 / 8	*
TOX	100		8 / 8	*

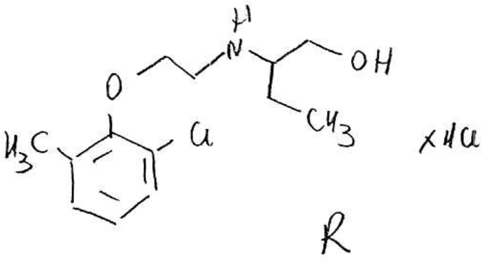

ED50 Biological Response Comments

Test	Dose (mg/kg)	Time	Code	Comment
SCMET	70	0.25	3	Death following continuous seizure
TOX	50	0.25	14	Unable to grasp rotorod
TOX	70	0.25	14	Unable to grasp rotorod
TOX	70	0.25	34	Muscle spasms
TOX	100	0.25	14	Unable to grasp rotorod
TOX	100	0.25	34	Muscle spasms

T[illegible] to Peak Effect

KM526

Anticonvulsant Screening Program

Test 4 Results - Mice I.P. Quantification

Add ID: 400031 U Screen ID: 1

Time (Hours)				0.25		0.5		1.0		2.0		4.0		6.0		8.0		24		3.0	
Test	Dose	Form	Dths	N / F	C	N / F	C	N / F	C	N / F	C	N / F	C	N / F	C	N / F	C	N / F	C	N / F	C
MES	25			4 / 4		3 / 4		0 / 4		0 / 4		0 / 4		/		/		/		/	
TOX	50			3 / 8	14	0 / 8		0 / 8		0 / 8		0 / 8		/		/		/		/	

Note: N/F = number of animals active or toxic over the number tested.

C= Comment code

Response Comments

Test	Dose(mg/kg)	Time	Code	Comments
TOX	50	0.25	14	Unable to grasp rotorod

Comments to Supplier:

KM-526

Anticonvulsant Screening Program

Test 8 Results - Anticonvulsant Identification (Rats I.P.)

Add ID: 400031 A **Screen ID:** 1

**Solvent Code:**	MC	**Solvent Prep:**	M&P,SB
**Animal Weight:**	115.0 - 150.0 g		
**Date Started:**	26-Nov-2008	**Date Completed:**	26-Nov-2008
**Reference:**	433:71		

Response

Time (Hours)				0.25		0.5		1.0		2.0		4.0		6.0		8.0		24		3.0	
Test	Dose	Form	Dths	N / F	C	N / F	C	N / F	C	N / F	C	N / F	C	N / F	C	N / F	C	N / F	C	N / F	C
MI[illegible]	30			4 / 4		3 / 4		3 / 4		1 / 4		0 / 4		/		/		/		/	
TOX	30			0 / 4		0 / 4		0 / 4		0 / 4		0 / 4		/		/		/		/	

Note: N/F = number of animals active or toxic over the number tested.

C= Comment code

Comments to Supplier:

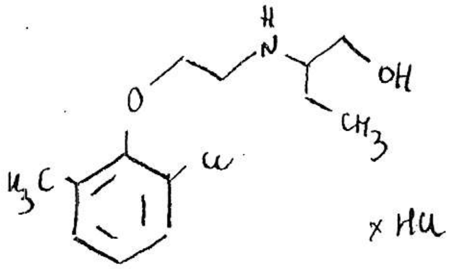

R

KN 526

Anticonvulsant Screening Program

Test 10 Results - Anticonvulsant Quantification (Rats I.P.)

Add ID: 400031 A Screen ID: 1

Solvent Code: MC Solvent Prep: TT Route Code: IP

Animal Weight: - g

Date Started: 27-Jan-2009 Date Completed: 09-Feb-2009

Reference: 424:243-255

ED50 Value

Test	Time(Hrs)	ED50	95% Confidence Interval	Slope	STD Err	PI Value
MES	0.25	3.9	2.1 - 6.2	2.8	0.7	
TOX	0.25	39.6	36.2 - 78.3	17	7.8	

ED50 Biological Response

Test	Dose (mg/kg)	Dths	N / F	C
MES	1		1 / 8	
MES	3		2 / 8	
MES	7.5		6 / 8	
MES	15		8 / 8	
MES	30		8 / 8	
TOX	30		0 / 8	
TOX	35		2 / 8	
TOX	40		4 / 8	
TOX	80	4	8 / 8	1

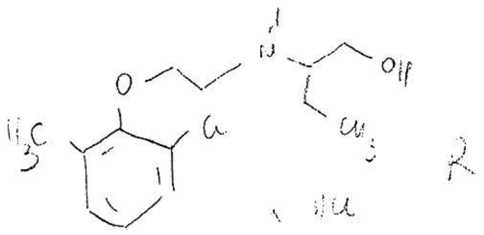

ED50 Biological Response Comments

Test	Dose (mg/kg)	Time	Code	Comment
TOX	80	0.25	1	Death

Time to Peak Effect

Time (Hours)			0.25		0.5		1.0		2.0		4.0		6.0		8.0		24		3.0	
Test	Dose	Dths	N / F	C	N / F	C	N / F	C	N / F	C	N / F	C	N / F	C	N / F	C	N / F	C	N / F	C
TOX	80	4	8 / 8	1	1 / 4		0 / 4		0 / 4		0 / 4		0 / 4		0 / 4		0 / 4		/	
TOX	125	1	2 / 2	1	1 / 1		0 / 1		0 / 1		0 / 1		0 / 1		0 / 1		0 / 1		/	
TOX	250	2	2 / 2	1	/		/		/		/		/		/		/		/	

KM-526

Anticonvulsant Screening Program

Test 10 Results - Anticonvulsant Quantification (Rats I.P.)

Add ID: 400031 A Screen ID: 1

TOX	500	2	2 / 2	1	/	/	/	/	/	/	/	/

Note: N/F = number of animals active or toxic over the number tested.

C= Comment code

Response Comments

Test	Dose (mg/kg)	Time	Code	Comments
TOX	80	0.25	1	Death
TOX	125	0.25	1	Death
TOX	250	0.25	1	Death
TOX	500	0.25	1	Death

Comments to Supplier:

KM-526

Anticonvulsant Screening Program
Test 26 Results - Corneal Kindled Mouse

Add ID: 400031 U Screen ID: 1

Solvent Code: MC **Solvent Prep:** TT **Route Code:** IP

Date Started: 04-Mar-2009 **Date Completed:** 04-Mar-2009

Reference: 438:221

Time Course

Dose (mg/kg)	Time (hrs)	N / F C	Individual Seizure Scores	Average Seizure Score
25	0.25	0 / 4	4,5,4,4	4.3

Comments to Supplier:

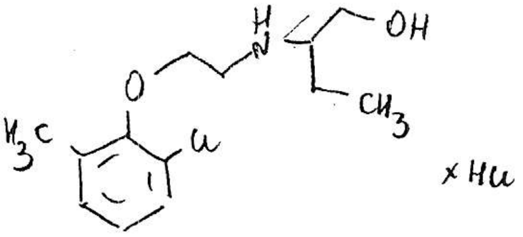

R

KM-526

Anticonvulsant Screening Program

Test 76 Results - In-vitro Hippocampal Slice Culture Neuroprotection Assay (NP)

Add ID: 400031 B Screen ID: 2

Solvent Code:	DMSO	Solvent Prep:	
Date Started:	11-Nov-2009	Date Completed:	20-Nov-2009
Reference:	439:251,258		

Summary of NP Assay: Kainic acid

- **Test Result:** No Neuroprotection

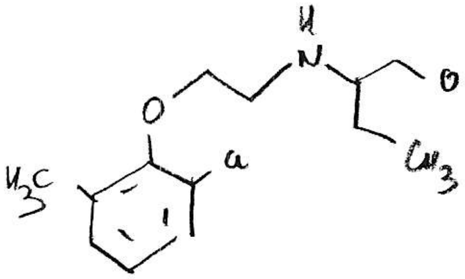

R

XHCl

Comments to Supplier:

TEST 76: *in vitro* HIPPOCAMPAL SLICE CULTURE NEUROPROTECTION ASSAY

Compound 1 : **ADD Number:** 400031 **Batch:** B **Date Started:** 11-Nov-2009

Compound 2 : **ADD Number:** **Batch:** **Date Completed:** 20-Nov-2009

References: 439: 251, 258

Excitotoxin: Kainic Acid **Insult Duration:** 4 Hours **Solvent:** DMSO

Primary Screen Results: No neuroprotection observed

EXPERIMENT IMAGES & WELL DESCRIPTION

A1	A2	A3
KA 20µM	KA 20µM + 400031 10µM	KA 20µM + 400031 10µM

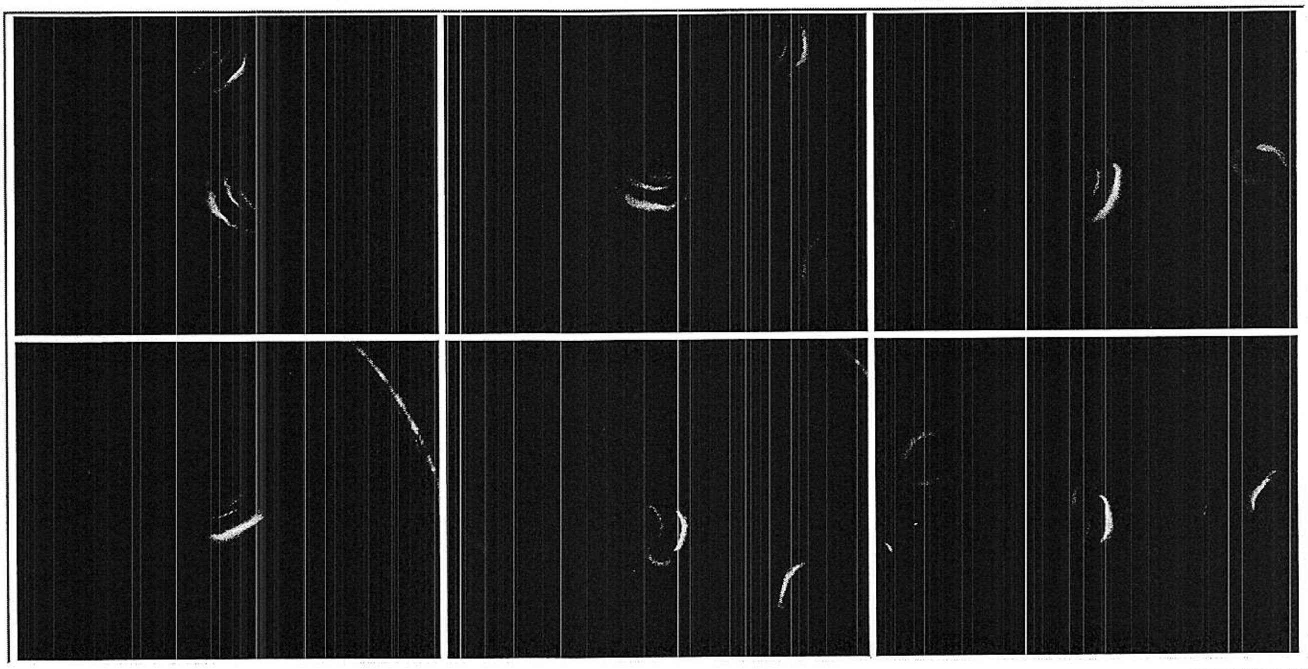

B1	B2	B3
KA 20µM	KA 20µM + 400031 100µM	KA 20µM + 400031 100µM

PRIMARY SCREEN EXPERIMENT DESCRIPTION

The "Primary Screen Experiment" is a qualitative assessment of the ability of a compound to prevent excitotoxic cell death. Organotypic hippocampal slice cultures are treated with N-methyl-D-aspartate (NMDA) or kainic acid (KA) to induce neuronal cell death. Propidium iodide (PI), a membrane-impermeant compound, is included in all wells of the culture plate. Dying cells have compromised cell membranes, thus PI may diffuse into the cell, intercalate with DNA and fluoresce. Thus, the intensity of the PI fluorescence is proportional to the amount of cell death in the individual slices. Hippocampal slice cultures are treated with the excitotoxin alone, or where indicated above, with the excitotoxin and either **one or two** investigational compounds at the concentrations indicated. If neuroprotection occurs as a consequence of the added compound, slice cultures will have a visibly reduced fluorescent intensity when compared to the slice cultures that have been treated with the excitotoxin alone.

Anticonvulsant Screening Program

Test 76 Results - In-vitro Hippocampal Slice Culture Neuroprotection Assay (NP)

Add ID: 400031 B Screen ID: 1

Solvent Code:	DMSO	Solvent Prep:	
Date Started:	11-Nov-2009	Date Completed:	20-Nov-2009
Reference:	439:251,258		

Summary of NP Assay: NMDA

- Test Result: **No Neuroprotection**

Comments to Supplier:

TEST 76: *in vitro* HIPPOCAMPAL SLICE CULTURE NEUROPROTECTION ASSAY

Compound 1 : **ADD Number:** 400031 **Batch:** B **Date Started:** 11-Nov-2009

Compound 2 : **ADD Number:** **Batch:** **Date Completed:** 20-Nov-2009

References: 439: 251, 258

Excitotoxin: **NMDA** **Insult Duration:** 4 Hours **Solvent:** DMSO

Primary Screen Results: **No neuroprotection observed**

EXPERIMENT IMAGES & WELL DESCRIPTION

**A1** NMDA 10µM	**A2** NMDA 10µM + 400031 10µM	**A3** NMDA 10µM + 400031 10µM

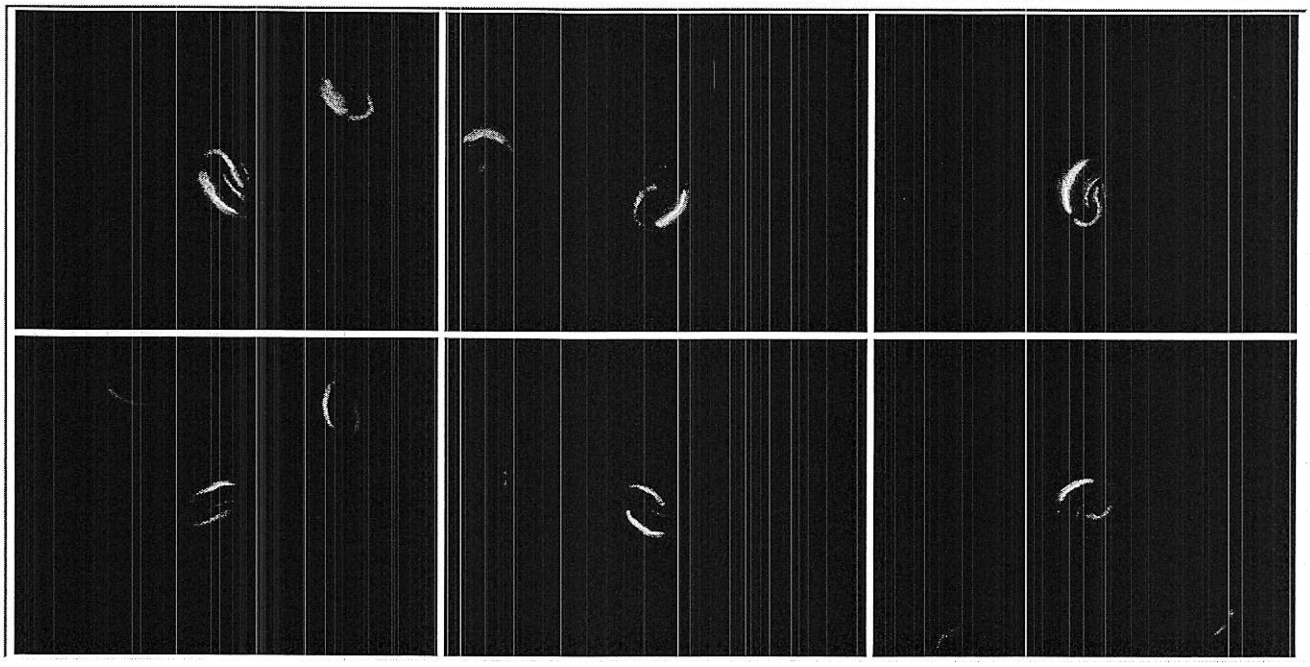

**B1** NMDA 10µM	**B2** NMDA 10µM + 400031 100µM	**B3** NMDA 10µM + 400031 100µM

PRIMARY SCREEN EXPERIMENT DESCRIPTION

The "Primary Screen Experiment" is a qualitative assessment of the ability of a compound to prevent excitotoxic cell death. Organotypic hippocampal slice cultures are treated with N-methyl-D-aspartate (NMDA) or kainic acid (KA) to induce neuronal cell death. Propidium iodide (PI), a membrane-impermeant compound, is included in all wells of the culture plate. Dying cells have compromised cell membranes, thus PI may diffuse into the cell, intercalate with DNA and fluoresce. Thus, the intensity of the PI fluorescence is proportional to the amount of cell death in the individual slices. Hippocampal slice cultures are treated with the excitotoxin alone, or where indicated above, with the excitotoxin and either **one or two** investigational compounds at the concentrations indicated. If neuroprotection occurs as a consequence of the added compound, slice cultures will have a visibly reduced fluorescent intensity when compared to the slice cultures that have been treated with the excitotoxin alone.

KM-526

Anticonvulsant Screening Program

Test 11 Results - Preliminary Hippocampal Kindling Screen - Rats

Add ID: 400031 B **Screen ID:** 1

Solvent Code: MC **Solvent Prep:** TT **Route Code:** IP

Animal Weight: - g

Date Started: 20-Jul-2011 **Date Completed:** 20-Jul-2011

Reference: 464: 280-283

Dose: 40 mg/kg Time of Maximum Effect: 15 to min

		Seizure Score		Afterdischarge Duration(secs)	
Rat #	Comment Code	Pre-Drug	Drug	Pre-Drug	Drug
1		4 - 5	4 -	11 - 19	63 -
2		5 -	0 -	19 - 26	14 -

Comments to Supplier:

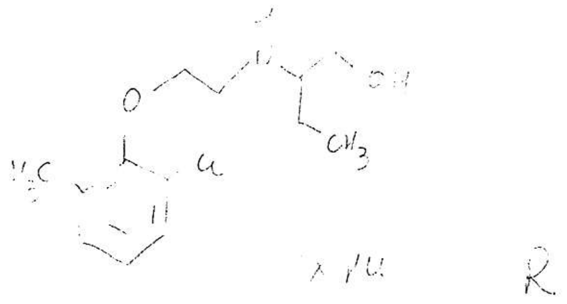

KH-526

Anticonvulsant Screening Program

Test 22 Results - Formalin Test (Mice I.P.)

Add ID: 400031 B Screen ID: 1

Solvent Code: MC **Solvent Prep:** TT,SB **Route Code:** IP

Time of Test: 0.25 (hrs)

Date Started: 20-Oct-2011 **Date Completed:** 21-Oct-2011

Reference: F06:55

Analysis

		Area Under the Curve				
Dose (mg/kg)	Test	Control	Drug Treated	% of Control	S.E.M	p Value
20.0	Acute	242.31	185.28	76.46	18.06	> 0.05
20.0	Inflammatory	805.09	757.15	94.04	10.5	> 0.05

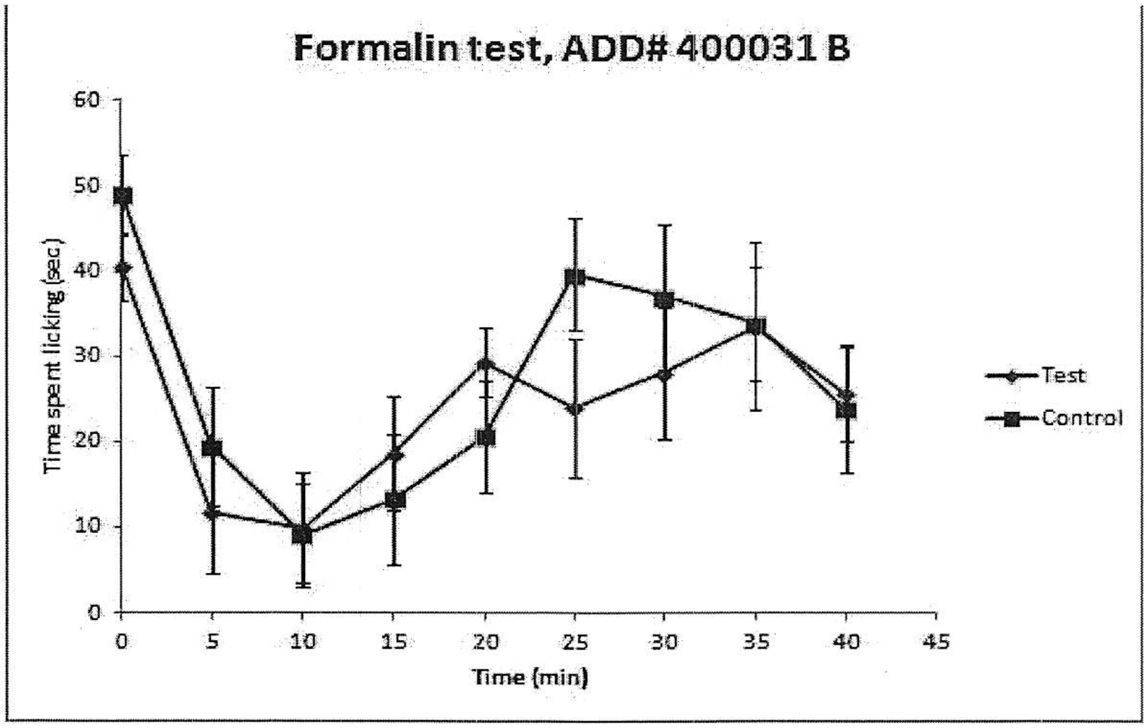

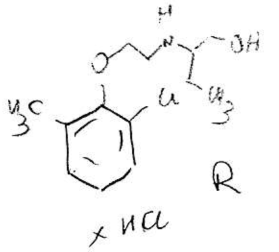

Response

Trial 1

		Duration of Licking (sec)											
Dose (mg/kg)	Animal #	0 min	5 min	10 min	15 min	20 min	25 min	30 min	35 min	40 min	45 min	50 min	55 min
**0.0**	01	48.16	8.72	0.00	0.00	1.48	22.30	35.57	39.25	4.69			

Anticonvulsant Screening Program

Test 22 Results - Formalin Test (Mice I.P.)

Add ID: 400031 B Screen ID: 1

**0.0**	02	63.31	50.94	0.00	61.66	57.25	61.88	43.58	9.30	12.49			
**0.0**	03	33.49	1.24	0.00	0.00	2.30	36.24	12.24	29.18	6.85			
**0.0**	04	51.10	18.54	0.00	18.39	4.49	64.79	49.40	59.93	57.46			
**0.0**	05	24.51	16.75	0.00	0.00	32.45	51.49	3.91	4.04	9.78			
**0.0**	06	59.28	0.00	0.00	0.00	22.31	17.52	76.01	34.55	35.07			
**0.0**	07	55.37	47.79	30.07	3.57	21.34	41.18	54.82	46.64	49.28			
**0.0**	08	56.42	11.78	42.19	22.60	23.75	22.46	20.87	48.07	14.79			

Trial 1

		Duration of Licking (sec)											
Dose (mg/kg)	Animal #	0 min	5 min	10 min	15 min	20 min	25 min	30 min	35 min	40 min	45 min	50 min	55 min
**20.0**	01	38.54	6.88	7.06	18.90	21.21	51.39	16.10	18.63	32.48			
**20.0**	02	32.33	1.67	3.46	5.46	22.84	0.00	0.00	42.75	47.27			
**20.0**	03	56.72	59.78	0.00	25.69	50.35	0.00	44.54	14.30	8.73			
**20.0**	04	55.64	3.43	52.37	18.76	16.93	33.84	61.36	87.30	0.00			
**20.0**	05	35.04	15.57	16.61	16.45	23.07	22.13	28.87	8.91	19.90			
**20.0**	06	27.69	6.85	0.00	59.79	34.64	26.10	40.48	5.51	22.93			
**20.0**	07	46.09	0.00	0.00	4.52	25.48	58.46	33.55	35.87	41.04			
**20.0**	08	33.01	0.00	0.00	0.00	40.21	0.00	0.00	54.76	32.76			

Comments to Supplier:

KM-526

Anticonvulsant Screening Program

Test 23 Results - Sciatic Ligation Model In Rats

Add ID: 400031 B Screen ID: 1

**Solvent Code:**	MC	**Solvent Prep:**	M&P,SB	**Route Code:**	IP
**Date Started:**	28-Mar-2012	**Date Completed:**	28-Mar-2012		
**Reference:**	SL5:03				

Analysis

Dose (mg/kg)	Time (hrs)	Mean values +/- S.E.M		
		Threshold +/- S.E.M	% Pre-Drug +/- S.E.M	
4.0	0.0	2.74 +/- 0.3	100 +/- 11	☐
4.0	0.5	4.57 +/- 0.77	203 +/- 47	☐
4.0	1.0	8.61 +/- 2.38	317 +/- 79	☑
4.0	2.0	6.62 +/- 1.59	233 +/- 46	☐
4.0	4.0	2.91 +/- 0.3	124 +/- 18	☐
4.0	6.0	2.57 +/- 0.49	107 +/- 20	☐

* % threshold of ligated leg prior to drug administration.

Note: Box checked if data is significantly different from control.

Results of Analysis

Dose (mg/kg)	Time of Peak Effect (hrs)	* % Pre-Drug	
		Threshold +/- SEM	
4.0	1.0	317 +/- 79	☑

Note: Box checked if data is significantly different from pre-drug.

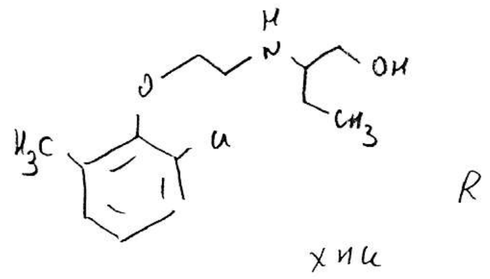

VH-526

Anticonvulsant Screening Program

Test 23 Results - Sciatic Ligation Model In Rats

Add ID: 400031 B Screen ID: 1

400031 B Manual von Frey Test

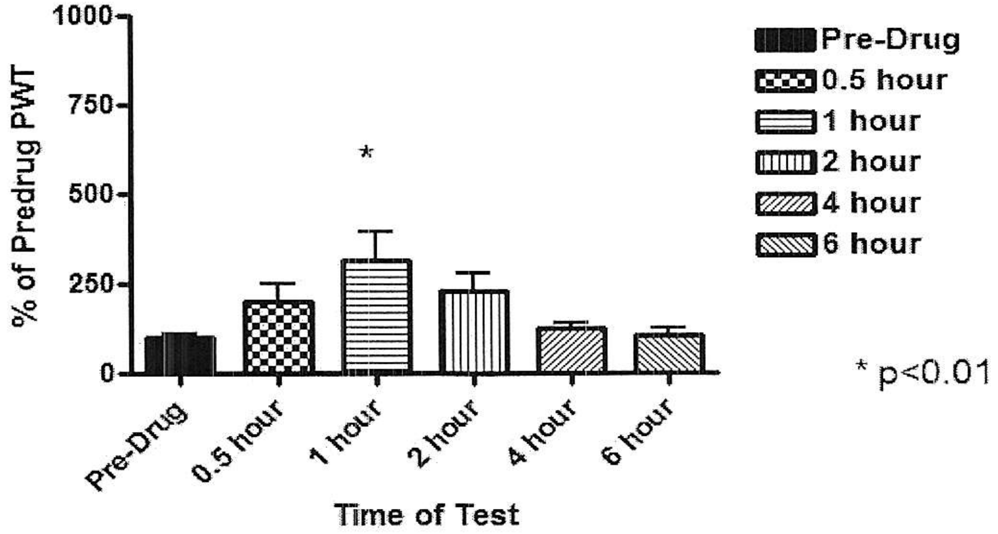

Response per Animal

		Threshold for Foot Withdrawal (grams)									
Dose (mg/kg)	Animal #	Pre-Drug	1hr	2hr	4hr	6hr	24hr	0.5hr			
4.0	1	2.87	4.34	2.87	1.61	1.61		2.87			
4.0	2	1.61	11.96	4.34	2.87	2.87		6.73			
4.0	3	4.34	22.8	11.96	9.29	11.96		22.8			
4.0	4	2.87	6.73	9.29	2.87	4.34		4.34			
4.0	5	2.87	9.29	9.29	2.87	1.61		8.0			
4.0	6	2.87	9.29	11.96	4.34	4.34		2.87			
4.0	7	2.87	1.61	1.61	2.87	1.61		2.87			
4.0	8	1.61	2.87	1.61	2.97	1.61		4.34			

Comment for Response: Animal 03 at 0.5, 4 and 6 hrs is a significant outlier as defined by Grubb's test (p<0.05) on Graphpad website(http://www.graphpad.com/quickcalcs/grubbs1.cfm) No toxicity observed.

Comments to Supplier:

KM-526

Anticonvulsant Screening Program

Test 72 Results - Pilocarpine-induced Status, Rats - Time 30 Min

Add ID: 400031 B Screen ID: 1

Solvent Code: MC Solvent Prep: M&P,SB Route Code: IP

Date Started: 06-Jun-2012 Date Completed: 08-Jun-2012

Reference: CM4:417;418

Response Data

Dose (mg/kg)	Time (hrs) [a]	N / F [c]	Dths	Avg. Weight Change(g) +/- S.E.M [b]			
				Protected Rats		Non-Protected Rats	
40.00	0.5	0 / 7	4			-	25.0 +/-

a Post first Stage III seizure

b Weight change 24 hours Post first Stage III seizure

Comments to Supplier:

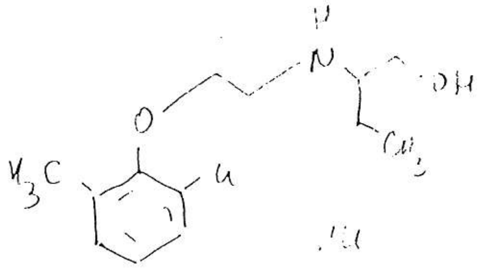

Supplement: Supplementary file 5 — Supplementary file5 (PDF 4814 KB) [file 43440_2022_431_MOESM5_ESM.pdf]
