# Supplementary material for: KM-408, a novel phenoxyalkyl derivative as a potential anticonvulsant and analgesic compound for the treatment of neuropathic pain
Source: Pharmacol Rep. 2022 Nov 19;75(1):128–65. doi: 10.1007/s43440-022-00431-7 (PMC9889419; doi:10.1007/s43440-022-00431-7)
Supplement: Supplementary file 6 — Supplementary file6 (PDF 5916 KB) [file 43440_2022_431_MOESM6_ESM.pdf]

KM-332

Anticonvulsant Screening Project

Test 1 Results - Mice I.P. Identification

Add ID: 354010 U **Screen ID:** 1

Solvent Code: MC **Solvent Prep:** M&P,SB

Animal Weight: 20.5 to 25.5 g

Date Started: 24-Jun-2004 **Date Completed** 24-Jun-2004

Reference: 376:247

Response

Time (Hours)				0.5		4.0		0.25		1.0		2.0		6.0		3.0		8.0		24	
Test	Dose	Form	Dth	N / F	C	N / F	C	N / F	C	N / F	C	N / F	C	N / F	C	N / F	C	N / F	C	N / F	C
MES	30	SUS		1 / 1		0 / 1		/		/		/		/		/		/		/	
.S	100	SUS		1 / 1		0 / 0		/		/		/		/		/		/		/	
SCMET	30	SUS		0 / 1		0 / 1		/		/		/		/		/		/		/	
SCMET	100	SUS		0 / 0		0 / 1		/		/		/		/		/		/		/	
TOX	30	SUS		0 / 4		0 / 2		/		/		/		/		/		/		/	
TOX	100	SUS	6	8 / 8	*	0 / 1		/		/		/		/		/		/		/	
TOX	300	SUS	4	4 / 4	1	/		/		/		/		/		/		/		/	

Response Comments

TEST	DOSE (mg/kg	TIME	CODE	COMMENT
TOX	100	0.5	14	Unable to grasp rotorod
TOX	100	0.5	1	Death
TOX	300	0.5	1	Death

Comments to Supplier:

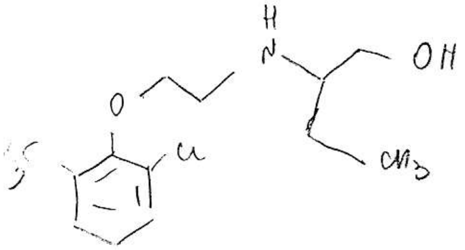

(S)

Anticonvulsant Screening Project

Test 8 Results -Anticonvulsant Identification (Rats I.P.)

Add ID: 354010 U **Screen ID:** 1

Solvent Code: MC Solvent Prep: M&P,SB

Animal Weight: 100 to 135 g

Date Started: 10-Nov-2006 Date Completed: 13-Nov-2006 KM 332

Reference: 397:263

Time to Peak Effect

Test	Dose (mg/kg)	# Dths	0.25		0.5		1.0		2.0		4.0		6.0		8.0		24		3.0	
			N / F	C	N / F	C	N / F	C	N / F	C	N / F	C	N / F	C	N / F	C	N / F	C	N / F	C
[illegible]ES	30		4 / 4		4 / 4		4 / 4		1 / 4		0 / 4		/		/		/		/	
TOX	30		0 / 4		0 / 4		0 / 4		0 / 4		0 / 4		/		/		/		/	

Comments to Supplier:

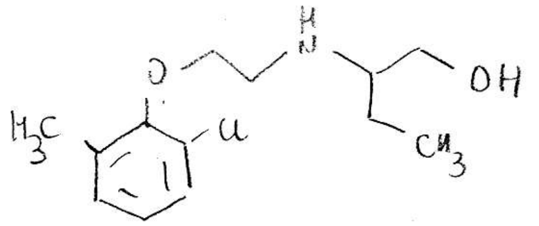

S (+)

KM 332

Anticonvulsant Screening Program

Test 10 Results - Anticonvulsant Quantification (Rats I.P.)

Add ID: 354010 A Screen ID: 1

Solvent Code: MC Solvent Prep: M&P,SB Route Code: IP

Animal Weight: - g

Date Started: 22-Mar-2007 Date Completed: 04-Apr-2007

Reference: 411:15-31

ED50 Value

Test	Time(Hrs)	ED50	95% Confidence Interval	Slope	STD Err	PI Value
MES	0.50	10.58	6.69 - 15.38	4.06	1.21	
SCMET	0.50	> 60.0	0.0 - 0.0			
TOX	0.25	44.29	40.72 - 47.52	23.78	7.92	

ED50 Biological Response

Test	Time (hr)	Dose (mg/kg)	Dths	N / F	C
MES	0.50	3.0		0 / 8	
MES	0.50	7.5		3 / 8	
MES	0.50	15.0		5 / 8	
MES	0.50	30.0		8 / 8	
SCMET	0.50	60.0		0 / 6	*
TOX	0.25	30.0		0 / 8	
TOX	0.25	40.0		2 / 8	
TOX	0.25	45.0		2 / 7	
TOX	0.25	50.0		8 / 8	
TOX	0.25	60.0		8 / 8	

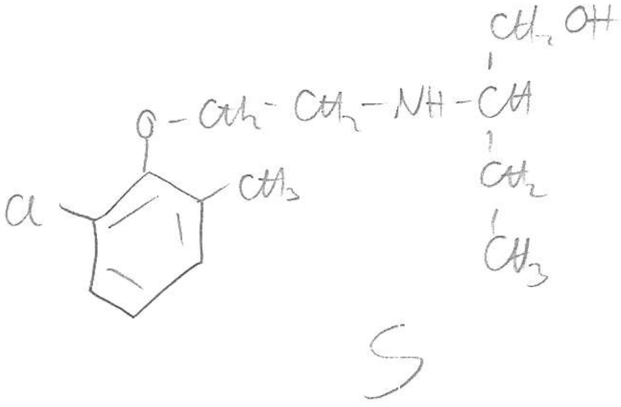

ED50 Biological Response Comments

Test	Dose (mg/kg)	Time (Hrs)	Code	Comment
SCMET	60	0.50	22	Continuous seizure activity
SCMET	60	0.50	3	Death following continuous seizure

Time to Peak Effect

Time (Hours)			0.25		0.5		1.0		2.0		4.0		6.0		8.0		24		3.0	
Test	Dose	Dths	N / F	C	N / F	C	N / F	C	N / F	C	N / F	C	N / F	C	N / F	C	N / F	C	N / F	C

Anticonvulsant Screening Program

Test 10 Results - Anticonvulsant Quantification (Rats I.P.)

Add ID: 354010 A Screen ID: 1

MES	7.5		3 / 4		4 / 4	3 / 4	/	/	/	/	/	/
MES	15.0		3 / 4		3 / 4	3 / 4	/	/	/	/	/	/
TOX	40.0		2 / 8		0 / 8	0 / 8	0 / 8	/	/	/	/	/
TOX	60.0		6 / 6		6 / 6	/	/	/	/	/	/	/
TOX	125.0	2	2 / 2	1	/	/	/	/	/	/	/	/
TOX	250.0	2	2 / 2	1	/	/	/	/	/	/	/	/
TOX	500.0	2	2 / 2	1	/	/	/	/	/	/	/	/

Note: N/F = number of animals active or toxic over the number tested.
C= Comment code

Response Comments

Test	Dose (mg/kg)	Time	Code	Comments
TOX	125	0.25	1	Death
TOX	250	0.25	1	Death
TOX	500	0.25	1	Death

Comments to Supplier:

KM 332

ANTICONVULSANT SCREENING PROJECT TEST RESULTS
COUNTERMEASURES

Test 71: Prevention of Pilocarpine-induced Status, Rats

ADD Number: 354010 A
Solvent: MC Prep: M&P,SB
Reference: 411:115,117-120

Date Started: 06-JUN-2007
Date Completed: 07-JUN-2007
Route: i.p.

Toxicity Test

Dose (mg/kg)	Time in Hours				
	.25	.5	1	2	4
300					
100					
30					

PiSE TPE Determination

Dose (mg/kg)	Time of Test (hrs)*	Prot./Tested	Comment	# Died	Ave. wght loss ± S.E.M.
45	0	3/8		4	+3.8 ± 5.7
					±
					±
					±

*post first Stage III seizure

Dose Response Data

PiSE Time of Test: hrs

mg/kg					
Pro./Test.					

Toxicity Time of Test: hrs

mg/kg					
Tox./Test.					

ED50 Values

TPE (hrs)		mg/kg	95% C.I.	Slope	± S.E.M.
	**TD50**				
	**ED50**				
	**ED97**				

Comment for NIH: No further testing recommended at this point.

KM 332

Anticonvulsant Screening Project

Test 11 Results - Preliminary Hippocampal Kindling Screen - Rats IP

Add ID: 354010 A **Screen ID:** 1

Solvent Code: MC | Solvent Prep: M&P,SB | Route Code: IP

Animal Weight: to g

Date Started: 30-Jul-2007 | Date Completed: 30-Jul-2007

Reference: 418:43-47

Dose: 45 mg/kg | Time of Maximum Effect: 15 to min

Rat #	Comment Code	Seizure Score Pre-Drug Low	Seizure Score Pre-Drug High	Seizure Score Drug Low	Seizure Score Drug High	Afterdischarge Duration (secs) Pre-Drug Low	Afterdischarge Duration (secs) Pre-Drug High	Afterdischarge Duration (secs) Drug Low	Afterdischarge Duration (secs) Drug High
1	15	5 -		0 -		29 -	42	33 -	
2	15	5 -		3 -		22 -	50	43 -	

Response Comments

RAT #	DOSE (mg/kg)	CODE	COMMENT
1	45	15	Minimal motor impairment
2	45	15	Minimal motor impairment

Comments to Supplier:

KM-332

Anticonvulsant Screening Program

Test 26 Results - Corneal Kindled Mouse

Add ID: 354010 A **Screen ID:** 1

Solvent Code: MC **Solvent Prep:** M&P,SB **Route Code:** IP

Date Started: 31-Aug-2007 **Date Completed:** 17-Oct-2007

Reference:

ED50 Value

Test	Time(Hrs)	ED50	95% Confidence Interval	Slope	STD Err	PI Value
CKM	0.25	10.50	3.67 - 22.09	1.44	0.48	

ED50 Biological Response

Test	Dose (mg/kg)	Dths	N / F	C
CKM	2		1 / 8	
CKM	5		4 / 8	
CKM	15		3 / 8	
CKM	25		3 / 4	
CKM	30		6 / 8	
CKM	50		4 / 4	Z

ED50 Biological Response Comments

Test	Dose (mg/kg)	Time	Code	Comment
CKM	50	0.25	Z	4/4 toxic; 3/4 had seizure activity before test

Time to Peak Effect

Time (Hours)				0.25		0.5		1.0		2.0		4.0		6.0		8.0		24		3.0	
Test	Dose	Form	Dths	N / F	C	N / F	C	N / F	C	N / F	C	N / F	C	N / F	C	N / F	C	N / F	C	N / F	C
CKM	25			3 / 4		2 / 4		0 / 4		/		/		/		/		/		/	

Note: N/F = number of animals active or toxic over the number tested.

C= Comment code

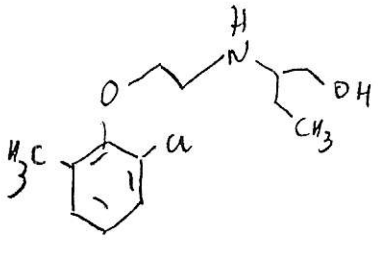

(S)

Comments to Supplier:

KM-332

Anticonvulsant Screening Project

Test 13 Results - Hippocampal Kindled Rats

Add ID: 354010 A Screen ID: 1

Solvent Code: MC Solvent Prep: M&P,SB Route Code: IP

Date Started: 07-Sep-2007 Date Completed: 25-Sep-2007

Reference: 418:110-113,139-186

ED50 Values

Test	Time (Hrs)	ED50	95% Confidence Interval LOW	HIGH	SLOPE	STD. ERR.	PI VALUE
KIN	0.25	15.81	8.65	30.38	2.15	0.64	

T[illegible]e Course

Dose (mg/kg)	Time (min.)	Seizure Score ± SEM		Duration (sec) ± SEM	
2	0	4.8 ± 0.2	☐	30 ± 4	☐
2	15	5.0 ± 0.0	☐	66 ± 14	☑
2	45	4.9 ± 1.0	☐	78 ± 11	☑
2	75	4.9 ± 0.1	☐	79 ± 8	☑
2	105	4.8 ± 0.2	☐	77 ± 14	☑
2	135	4.9 ± 0.1	☐	72 ± 10	☑

Note: Box checked if data is significantly different from control.

6	0	5.0 ± 0.0	☐	48 ± 5	☐
6	15	4.9 ± 0.1	☐	62 ± 4	☑
6	45	5.0 ± 0.0	☐	76 ± 12	☐
6	75	4.1 ± 0.6	☐	60 ± 7	☐
6	105	4.7 ± 0.2	☐	79 ± 13	☑
6	135	4.3 ± 0.3	☑	63 ± 7	☐

Note: Box checked if data is significantly different from control.

11	0	5.0 ± 0.0	☐	44 ± 7	☐
11	15	2.6 ± 0.8	☑	59 ± 13	☐
11	45	4.3 ± 0.6	☐	63 ± 9	☐
11	75	4.5 ± 0.3	☐	67 ± 11	☐
11	105	4.6 ± 0.3	☐	65 ± 12	☐
11	135	4.5 ± 0.3	☐	61 ± 6	☐

Note: Box checked if data is significantly different from control.

22	0	4.6 ± 0.2	☐	23 ± 3	☐
22	15	1.7 ± 0.7	☑	54 ± 11	☑
22	45	2.7 ± 0.7	☑	65 ± 10	☑

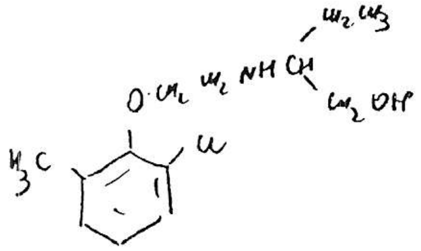

S

KM-332

Anticonvulsant Screening Project

Test 13 Results - Hippocampal Kindled Rats

Add ID: 354010A   Screen ID: 1[illegible]

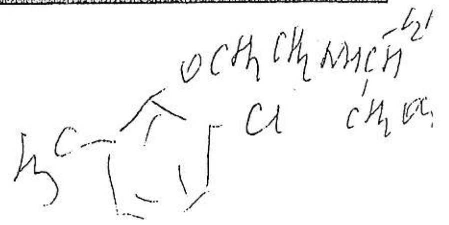

5

22	75	4.0 ± 0.6	☐	72 ± 8	☑
22	105	4.4 ± 0.2	☐	77 ± 14	☑
22	135	4.3 ± 0.7	☐	60 ± 17	☐

Note: Box checked if data is significantly different from control.

45	0	4.9 ± 0.1	☐	29.4 ± 4	☐
45	15	2.5 ± 0.7	☑	39 ± 11	☐
45	45	2.5 ± 0.7	☑	44 ± 11	☐
45	75	2.9 ± 0.7	☑	58 ± 15	☐
45	105	3.0 ± 0.7	☑	59 ± 4	☑
45	135	4.1 ± 0.5	☐	76 ± 15	☑

Note: Box checked if data is significantly different from control.

Dose Responses

Dose (mg)	Time (minutes)	Comment Code	N Toxic/ F Tested	N Protected / F Tested
2	15		0/ 8	0/ 8
6	75		0/ 7	1/ 7
11	15		0/ 8	4/ 8
22	15		2/ 7	5/ 7
45	15		4/ 8	6/ 8

Comments to Supplier:

KM-332

Anticonvulsant Screening Program

Test 76 Results - In-vitro Hippocampal Slice Culture Neuroprotection Assay

Add ID: 354010 A Screen ID: 1

Solvent Code: DMSO **Solvent Prep:**

Date Started: 13-Nov-2007 **Date Completed:** 13-Nov-2007

Reference: 423:50

Response

Add Compound Conc.(uM)	# of slices	% of Total Propidium Iodide Uptake (mean +/- S.E.M)
0.00	6.00	18.4 +/- 5.0 ☐
10.00	8.00	14.9 +/- 2.0 ☐
100.00	8.00	33.1 +/- 7.0 ☐

Note: box is checked if data is significantly different from kainic acid treatment alone, $p<0.05$.

IC50(uM) +/- S.E.M

Comments to Supplier:

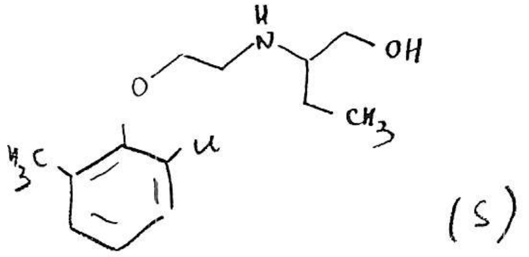

KM-332

Anticonvulsant Screening Program

Test 72 Results - Pilocarpine-induced Status, Rats - Time 30 Min

Add ID: 354010 A Screen ID: 1

Solvent Code: MC | Solvent Prep: M&P,SB | Route Code: IP

Date Started: 10-Dec-2007 | Date Completed: 12-Dec-2007

Reference: CM1:103-104

Response Data

Dose (mg/kg)	Time (hrs) [a]	N / F	C	Dths	Avg. Weight Change(g) +/- S.E.M [b] Protected Rats		Non-Protected Rats	
90.00	0.5	0 / 8		7			-	30.0 +/- 0.0

a Post first Stage III seizure
b Weight change 24 hours Post first Stage III seizure

Comments to Supplier:

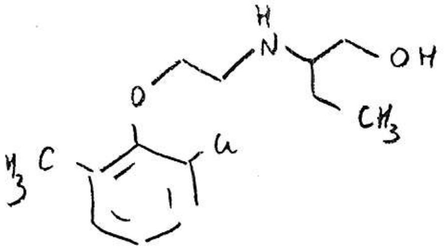

(S)

KM-332

Anticonvulsant Screening Program

Test 4 Results - Mice I.P. Quantification

Add ID: 354010 A Screen ID: 1

Solvent Code: MC Solvent Prep: M&P,SB

Animal Weight: - g

Date Started: 13-Feb-2008 Date Completed: 13-Feb-2008

Reference: 427:60-63

ED50 Value

Test	Time(Hrs)	ED50	95% Confidence Interval	Slope	STD Err	PI Value
MES	0.25	20.8	13.8 - 29.4	5.4	1.8	
TOX	0.25	47.7	32.6 - 68.3	4.6	1.5	

ED50 Biological Response

Test	Dose (mg/kg)	Dths	N / F	C
MES	12		1 / 8	
MES	25		5 / 8	
MES	50		8 / 8	
TOX	12		0 / 8	
TOX	25		1 / 8	
TOX	50		4 / 8	14
TOX	80	2	7 / 8	1

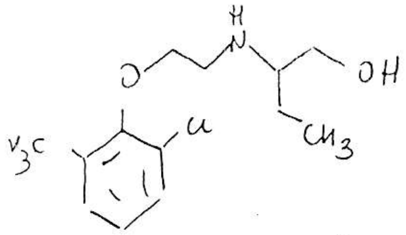

(S)

ED50 Biological Response Comments

Test	Dose (mg/kg)	Time	Code	Comment
TOX	50	0.25	14	Unable to grasp rotorod
TOX	80	0.25	1	Death

Time to Peak Effect

Time (Hours)			0.25		0.5		1.0		2.0		4.0		6.0		8.0		24		3.0	
Test	Dose	Dths	N / F	C	N / F	C	N / F	C	N / F	C	N / F	C	N / F	C	N / F	C	N / F	C	N / F	C
MES	25		1 / 4		1 / 4		0 / 4		0 / 4		/		/		/		/		/	
MES	50		8 / 8		6 / 8		/		/		/		/		/		/		/	
TOX	50		4 / 8	14	0 / 8		/		/		/		/		/		/		/	

Note: N/F = number of animals active or toxic over the number tested.

8/10/2009 10:48:21 AM

Anticonvulsant Screening Program

UN-332

Test 4 Results - Mice I.P. Quantification

Add ID: 354010 A Screen ID: 1

C= Comment code

Response Comments

Test	Dose (mg/kg)	Time	Code	Comments
TOX	50	0.25	14	Unable to grasp rotorod

Comments to Supplier:

KM-332

Anticonvulsant Screening Program

Test 22 Results - Formalin Test (Mice I.P.)

Add ID: 354010 A **Screen ID:** 1

Solvent Code: MC Solvent Prep: M&P,SB

Time of Test: 0.25 (hrs) Route Code: IP

ED50: 20.75 (mg/kg) TD50: 47.71 (mg/kg)

Date Started: 26-Feb-2008 Date Completed: 28-Feb-2008

Reference: F3: 34

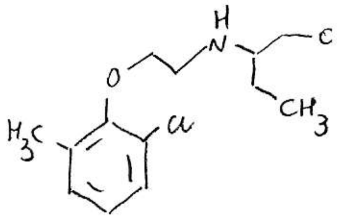

(S)

Analysis

Dose (mg/kg)	Test	Area Under the Curve: Control	Drug Treated	% of control	S.E.M	p Value
20	Acute	195.0	135.2	69.3	7.1	> 0.05
20	Inflammatory	677.0	387.0	57.2	6.1	< 0.01

Reponse

Trial 1 — Duration of Licking (sec)

Dose (mg/kg)	Animal #	0 min	5 min	10 min	15 min	20 min	25 min	30 min	35 min	40 min	45 min	50 min	55 min
0	01	49.11	0.00	0.00	8.98	33.73	32.54	15.27	23.73	0.00			
0	02	40.02	0.00	17.53	0.00	15.94	69.12	51.78	14.14	16.96			
0	03	53.89	8.72	0.00	25.93	9.16	59.82	10.49	0.00	0.00			
0	04	60.27	0.00	0.00	0.00	0.00	75.85	0.00	2.98	0.00			
0	05	40.95	0.00	0.00	0.00	46.63	21.46	41.44	48.62	43.31			
0	06	65.82	40.00	0.00	21.49	62.62	54.97	9.92	19.31	0.00			
0	07	48.47	0.00	15.04	32.36	25.85	38.08	19.35	39.25	73.72			
0	08	43.08	45.21	1.87	27.75	42.16	14.61	33.43	25.62	0.00			

Trial 1 — Duration of Licking (sec)

Dose (mg/kg)	Animal #	0 min	5 min	10 min	15 min	20 min	25 min	30 min	35 min	40 min	45 min	50 min	55 min
20	01	49.40	0.00	0.00	0.00	37.37	34.94	0.00	2.79	0.00			
20	02	33.40	0.00	0.00	0.00	0.00	20.35	0.00	28.36	18.78			
20	03	34.93	0.00	0.00	0.00	7.54	0.00	4.35	22.88	71.21	0.00	0.00	0.00
20	04	59.58	9.73	0.00	43.07	12.52	22.42	0.00	12.90	4.15			
20	05	51.53	0.00	0.00	16.59	16.09	12.99	38.27	0.00	21.41			
20	06	47.61	2.72	15.57	1.07	0.00	21.67	1.47	15.65	8.80			
20	07	59.52	0.00	0.00	9.67	0.00	22.43	61.78	14.54	36.68			
20	08	52.29	2.15	0.00	5.64	19.41	38.87	3.60	27.46	0.00			

V

Anticonvulsant Screening Program

Test 22 Results - Formalin Test (Mice I.P.)

Add ID: 354010 A **Screen ID:** 1

Comments to Supplier:

KM-332

Anticonvulsant Screening Program
Test 15 Results - IV Metrazol

Add ID: 354010 B Screen ID: 1

Solvent Code:	MC	Solvent Prep:	M&P,SB	Route Code:	IP
Time of Test:	0.25 (hrs)	Infusion Rate:	0.34 (ml/min)		
MES ED50:	21.00 (mg/kg)	TD50:	48.00 (mg/kg)		
Date Started:	24-Aug-2009	Date Completed:	24-Aug-2009		
Reference:	428: 234-235				

Anlaysis

Dose (mg/kg)		Weight (grams)	Time to Twitch	Twitch (mg/kg)	Time to Clonus	Clonus (mg/kg)
0	Mean	30.75	32.8	30.4	36.3	33.7
	Std. Err	0.84	0.91	1.18	0.99	1.41
	P-value					
21	Mean	29.95	29.0	27.6	35.8	33.9
	Std. Err	0.53	1.42	1.68	2.04	2.04
	P-value	0.216	0.019	0.094	0.406	0.465
48	Mean	31.45	27.0	24.3	32.9	29.6
	Std. Err	0.25	1.33	1.23	1.20	1.09
	P-value	0.221	0.001	0.001	0.020	0.016

Response

Dose (mg/kg)	Animal #	Weight (grams)	Time to Twitch	Twitch (mg/kg)	Time to Clonus	Clonus (mg/kg)
0	01	35.0	36.00	29.14	39.00	31.57
0	02	29.0	31.50	30.78	33.50	32.73
0	03	33.0	31.00	26.62	34.00	29.19
0	04	32.5	27.00	23.54	29.50	25.72
0	05	30.0	35.00	33.06	37.00	34.94
0	06	33.0	35.50	30.48	38.00	32.63
0	07	28.5	30.00	29.82	38.00	37.78
0	08	26.5	35.00	37.42	39.00	41.70
0	09	28.5	33.00	32.81	36.00	35.79
0	10	31.5	33.50	30.13	39.00	35.08
0	11					
0	12					
0	13					
0	14					

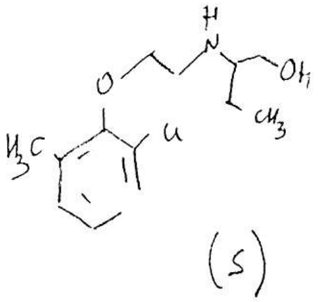

9/3/2009 9:20:02 AM

KM-332

Anticonvulsant Screening Program

Test 15 Results - IV Metrazol

Add ID: 354010 B Screen ID: 1

0	15					
0	16					
0	17					
0	18					
0	19					
0	20					
21	01	30.0	31.50	29.75	35.00	33.06
21	02	31.0	24.50	22.39	27.50	25.13
21	03	29.0	26.00	25.40	34.00	33.22
21	04	29.5	25.50	24.49	29.00	27.85
21	05	31.5	27.00	24.29	37.00	33.28
21	06	26.5	36.50	39.03	42.50	45.44
21	07	31.0	22.50	20.56	30.00	27.42
21	08	29.5	32.00	30.73	36.50	35.06
21	09	29.0	30.50	29.80	37.00	36.15
21	10	32.5	33.50	29.21	49.00	42.72
21	11					
21	12					
21	13					
21	14					
21	15					
21	16					
21	17					
21	18					
21	19					
21	20					
48	01	30.5	22.50	20.90	26.00	24.15
48	02	30.5	27.00	25.08	32.00	29.73
48	03	30.5	33.50	31.12	39.00	36.23
48	04	32.0	35.00	30.99	38.00	33.65
48	05	32.0	27.00	23.91	34.00	30.10
48	06	31.5	28.00	25.19	34.00	30.58
48	07	32.5	24.50	21.36	33.00	28.77
48	08	31.5	23.00	20.69	29.50	26.53
48	09	31.0	25.00	22.85	31.50	28.79
48	10	32.5	24.50	21.36	31.50	27.46

KM-332

Anticonvulsant Screening Program

Test 15 Results - IV Metrazol

Add ID: 354010 B **Screen ID: 1**

48	11					
48	12					
48	13					
48	14					
48	15					
48	16					
48	17					
48	18					
48	19					
48	20					

Comments to Supplier:

Anticonvulsant Screening Program

Test 24 Results - In vitro Slice Electrophysiology Studies

Add ID: 354010 C Screen ID: 1

Solvent Code: DMSO **Solvent Prep:**

Date Started: 01-Mar-2010 **Date Completed:** 01-Jun-2010

Reference: EP8 211-213, 222-223

Response

Test	Add Compound Conc.(uM)	# Slices	% Control Burst Rate	+/-	S.E.M		% Control Burst Dur.	+/-	S.E.M	
SPONTBURST	30	5	67	+/-	7	☐	49	+/-	11	☑

Note: Box checked if data is significantly different from control.

Comments to Supplier:

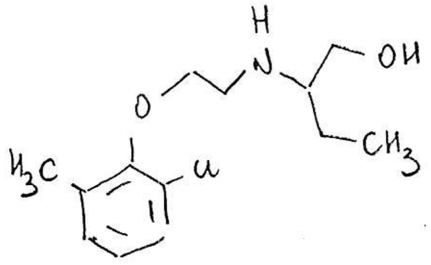

S-(+)

VM 332

Anticonvulsant Screening Program

Test 7 Results - Anticonvulsant Evaluation (6Hz, Mice)

Add ID: 354010 B **Screen ID:** 1

Solvent Code: MC Solvent Prep: M&P,SB Route Code: IP

Animal Weight: - g Current(mA): 32

Date Started: 02-Aug-2010 Date Completed: 02-Aug-2010

Reference: 456:136

Time to Peak Effect

Time (Hours)			0.25		0.5		1.0		2.0		4.0		6.0		8.0		24		3.0	
Test	Dose	Dths	N / F	C	N / F	C	N / F	C	N / F	C	N / F	C	N / F	C	N / F	C	N / F	C	N / F	C
6HZ	30		3 / 4		2 / 4		1 / 4		1 / 4		2 / 4		/		/		/		/	
TOX	30		0 / 4		0 / 4		0 / 4		0 / 4		0 / 4		/		/		/		/	

Note: N/F = number of animals active or toxic over the number tested.

C= Comment code

Comments to Supplier:

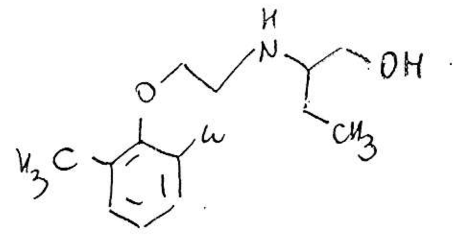

S-(+)

8/11/2010 12:10:55 PM

Anticonvulsant Screening Program

Test 7 Results - Anticonvulsant Evaluation (6Hz, Mice)

Add ID: 354010 U Screen ID: 2

Solvent Code: MC
Solvent Prep: M&P,SB
Route Code: IP
Animal Weight: - g
Current(mA): 32
Date Started: 17-Aug-2010
Date Completed: 19-Aug-2010
Reference: 456:162-165

ED50 Value

Test	Time(Hrs)	ED50	95% Confidence Interval	Slope	STD Err	PI Value
6HZ	0.25	20.4	14.9 - 25.4	5.7	1.7	
TOX	0.25	35.8	33.2 - 40.8	16.8	5.2	

ED50 Biological Response

Test	Dose (mg/kg)	Dths	N / F	C
6HZ	7		0 / 8	
6HZ	15		2 / 8	
6HZ	22		5 / 8	
6HZ	30		6 / 8	
6HZ	45		8 / 8	
TOX	30		0 / 8	
TOX	32		1 / 8	
TOX	35		6 / 8	14
TOX	45		7 / 8	*
TOX	75	1	8 / 8	*

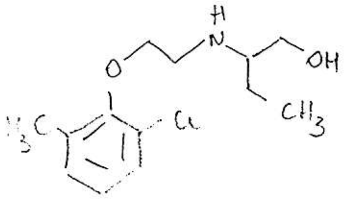

S

ED50 Biological Response Comments

Test	Dose (mg/kg)	Time (Hrs)	Code	Comment
TOX	35	0.25	14	Unable to grasp rotorod
TOX	45	0.25	14	Unable to grasp rotorod
TOX	45	0.25	34	Muscle spasms
TOX	75	0.25	14	Unable to grasp rotorod
TOX	75	0.25	34	Muscle spasms

Time to Peak Effect

'KM-332

Anticonvulsant Screening Program

Test 7 Results - Anticonvulsant Evaluation (6Hz, Mice)

Add ID: 354010 U Screen ID: 2

Time (Hours)			0.25		0.5		1.0		2.0		4.0		6.0		8.0		24		3.0	
Test	Dose	Dths	N / F	C	N / F	C	N / F	C	N / F	C	N / F	C	N / F	C	N / F	C	N / F	C	N / F	C
TOX	35		6 / 8	14	4 / 8	14	0 / 8		0 / 8		0 / 8		/		/		/		/	

Note: N/F = number of animals active or toxic over the number tested.

C= Comment code

Response Comments

Test	Dose (mg/kg)	Time	Code	Comments
TOX	35	0.25	14	Unable to grasp rotorod
TOX	35	0.50	14	Unable to grasp rotorod

Comments to Supplier:

VH-332

Anticonvulsant Screening Program

Test 76 Results - In-vitro Hippocampal Slice Culture Neuroprotection Assay (NP)

Add ID: 354010 C Screen ID: 2

Solvent Code:	DMSO	Solvent Prep:	
Date Started:	19-Oct-2010	Date Completed:	21-Oct-2010
Reference:	458:71		

Summary of NP Assay: NMDA

◎ Test Result: No Neuroprotection

Comments to Supplier:

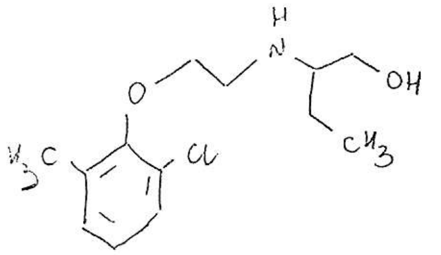

(S)

TEST 76: *in vitro* HIPPOCAMPAL SLICE CULTURE NEUROPROTECTION ASSAY

Compound 1 : ADD Number: 354010 Batch: C Date Started: 19-Oct-2010

Compound 2 : ADD Number: Batch: Date Completed: 21-Oct-2010

References: 458: 71

Excitotoxin: NMDA Insult Duration: 4 Hours Solvent: DMSO

Primary Screen Results: No neuroprotection observed

EXPERIMENT IMAGES & WELL DESCRIPTION

A1 NMDA 10μM

A2 NMDA 10μM + 354010 10μM

A3 NMDA 10μM + 354010 10μM

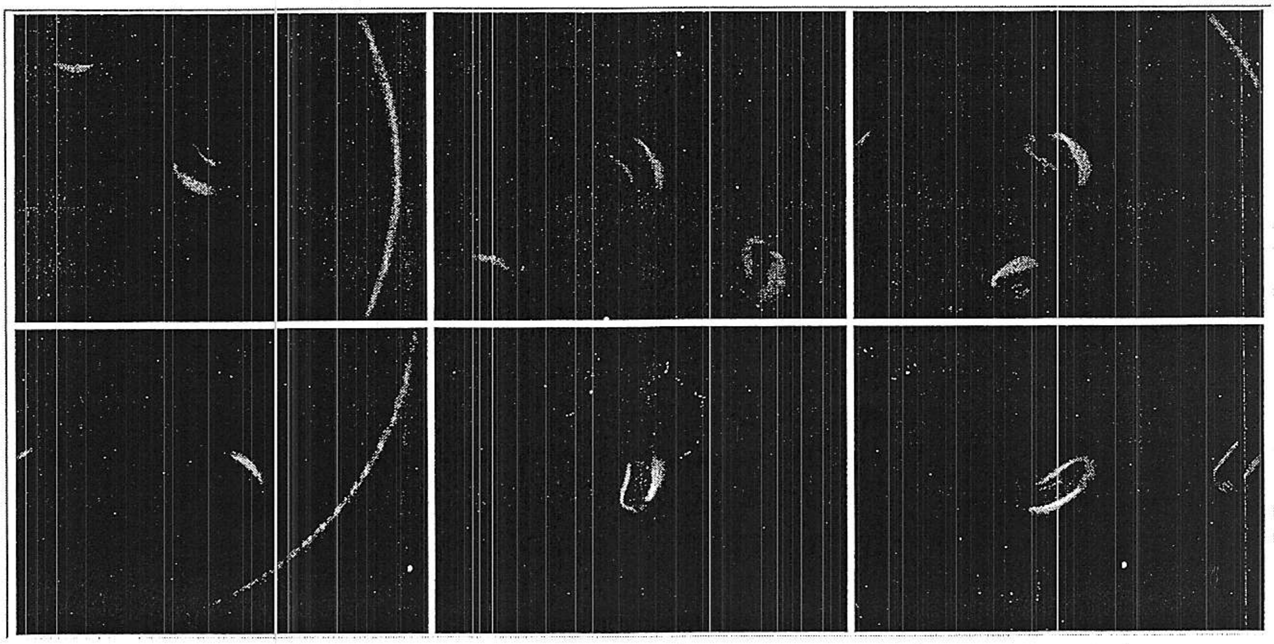

B1 NMDA 10μM

B2 NMDA 10μM + 354010 100μM

B3 NMDA 10μM + 354010 100μM

PRIMARY SCREEN EXPERIMENT DESCRIPTION

The "Primary Screen Experiment" is a qualitative assessment of the ability of a compound to prevent excitotoxic cell death. Organotypic hippocampal slice cultures are treated with N-methyl-D-aspartate (NMDA) or kainic acid (KA) to induce neuronal cell death. Propidium iodide (PI), a membrane-impermeant compound, is included in all wells of the culture plate. Dying cells have compromised cell membranes, thus PI may diffuse into the cell, intercalate with DNA and fluoresce. Thus, the intensity of the PI fluorescence is proportional to the amount of cell death in the individual slices. Hippocampal slice cultures are treated with the excitotoxin alone, or where indicated above, with the excitotoxin and either <u>one or two</u> investigational compounds at the concentrations indicated. If neuroprotection occurs as a consequence of the added compound, slice cultures will have a visibly reduced fluorescent intensity when compared to the slice cultures that have been treated with the excitotoxin alone.
