# Supplementary material for: KM-408, a novel phenoxyalkyl derivative as a potential anticonvulsant and analgesic compound for the treatment of neuropathic pain
Source: Pharmacol Rep. 2022 Nov 19;75(1):128–65. doi: 10.1007/s43440-022-00431-7 (PMC9889419; doi:10.1007/s43440-022-00431-7)
Supplement: Supplementary file 7 — Supplementary file7 (PDF 534 KB) [file 43440_2022_431_MOESM7_ESM.pdf]

KM-527

# Anticonvulsant Screening Program

## Test 1 Results - Mice I.P. Identification

**Add ID:** 400032 U **Screen ID:** 1

| | | | |
|---|---|---|---|
| Solvent Code: | MC | Solvent Prep: | M&P,SB |
| Animal Weight: | 21.0 - 25.0 g | | |
| Date Started: | 07-May-2008 | Date Completed: | 07-May-2008 |
| Reference: | 427:206 | | |

### Response

| Time (Hours) | | | | 0.5 | | 4.0 | | 0.25 | | 1.0 | | 2.0 | | 6.0 | | 3.0 | | 8.0 | | 24 | |
|---|---|---|---|---|---|---|---|---|---|---|---|---|---|---|---|---|---|---|---|---|---|
| Test | Dose | Form | Dths | N/F | C | N/F | C | N/F | C | N/F | C | N/F | C | N/F | C | N/F | C | N/F | C | N/F | C |
| MES | 30 | SOL | | 0/1 | | 0/1 | | / | | / | | / | | / | | / | | / | | / | |
| MES | 100 | SOL | | 1/1 | | 0/3 | | / | | / | | / | | / | | / | | / | | / | |
| SCMET | 30 | SOL | | 0/1 | | 0/1 | 22 | / | | / | | / | | / | | / | | / | | / | |
| SCMET | 100 | SOL | | 0/0 | | 0/1 | 22 | / | | / | | / | | / | | / | | / | | / | |
| SCMET | 300 | SOL | | 0/1 | 22 | 0/0 | | / | | / | | / | | / | | / | | / | | / | |
| TOX | 30 | SOL | | 0/4 | | 0/2 | | / | | / | | / | | / | | / | | / | | / | |
| TOX | 100 | SOL | 3 | 5/8 | * | 0/4 | | / | | / | | / | | / | | / | | / | | / | |
| TOX | 300 | SOL | 3 | 4/4 | * | / | | / | | / | | / | | / | | / | | / | | / | |

Note: N/F = number of animals active or toxic over the number tested.

C= Comment code

### Response Comments

| Test | Dose(mg/kg) | Time | Code | Comments |
|---|---|---|---|---|
| SCMET | 30 | 4 | 22 | Continuous seizure activity |
| SCMET | 100 | 4 | 22 | Continuous seizure activity |
| SCMET | 300 | 0.5 | 22 | Continuous seizure activity |
| TOX | 100 | 0.5 | 1 | Death |
| TOX | 100 | 0.5 | 14 | Unable to grasp rotorod |

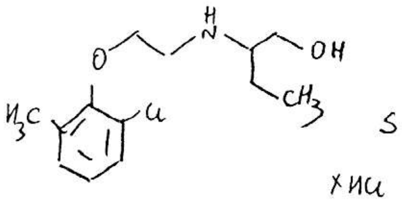

# Anticonvulsant Screening Program

KM-527

## Test 1 Results - Mice I.P. Identification

**Add ID: 400032 U Screen ID: 1**

**Response Comments**

| Test | Dose(mg/kg) | Time | Code | Comments |
|---|---|---|---|---|
| TOX | 300 | 0.5 | 1 | Death |
| TOX | 300 | 0.5 | 14 | Unable to grasp rotorod |

**mments to Supplier:**
