# Supplementary material for: KM-408, a novel phenoxyalkyl derivative as a potential anticonvulsant and analgesic compound for the treatment of neuropathic pain
Source: Pharmacol Rep. 2022 Nov 19;75(1):128–65. doi: 10.1007/s43440-022-00431-7 (PMC9889419; doi:10.1007/s43440-022-00431-7)
Supplement: Supplementary file 8 — Supplementary file8 (DOCX 2424 KB) [file 43440_2022_431_MOESM8_ESM.docx]

Supplementary material

# I. Tables

## Table S1. Radioligand binding assay results for compounds 4 and KM408 (% of control specific binding).

| **Assay** | **Ligand** | **Compd** | **Conc.**  **[μM]** | **Mean % of control specific binding** |
| --- | --- | --- | --- | --- |
| **A_1_ (antagonist radioligand)**  human recombinant (CHO cells) | [^3^H]DPCPX  (1 nM) | **4** | 100 | 124.5 |
| **A_2A_ (agonist radioligand)**  human recombinant (CHO cells) | [^3^H]CGS21680  (6 nM) | **4** | 100 | 71.7 |
| **A_3_ (agonist radioligand)**  human recombinant (CHO cells) | [^125^I]AB-MECA  (0.15 nM) | **4** | 100 | 115.1 |
| **α_1_ (non-selective) (antagonist radioligand)**  rat cerebral cortex | [^3^H]prazosin  (0.25 nM) | **4**  **KM408** | 100  0.01  0.1  1  10  100 | 15.9  102.4  97.3  90.0  55.9  12.8 |
| **α_2_ (non-selective) (antagonist radioligand)**  rat cerebral cortex | [^3^H]RX 821002  (0.5 nM) | **4**  **KM408** | 100  0.01  0.1  1  10  100 | 27.7  101.3  111.6  101.2  73.4  23.5 |
| **β_1_ (agonist radioligand)**  human recombinant (HEK-293 cells) | [^3^H](-)CGP 12177  (0.15 nM) | **4** | 100 | 64.4 |
| **β_2_ (agonist radioligand)**  human recombinant (CHO cells) | [^3^H](-)CGP 12177  (0.3 nM) | **4** | 100 | 85.1 |
| **AT_1_ (antagonist radioligand)**  human recombinant (HEK-293  cells) | [^125^I][Sar1,Ile8]-AT-II | **4** | 100 | 131.2 |
| **AT_2_ (agonist radioligand)**  human recombinant (HEK-293  cells) | [^125^I]CGP 42112A | **4** | 100 | 108.2 |
| **BZD (central) (agonist radioligand)**  rat cerebral cortex | [^3^H]flunitrazepam  (0.4 nM) | **4** | 100 | 110.8 |
| **BZD (peripheral) (antagonist radioligand)**  rat heart | [^3^H]PK 11195  (0.2 nM) | **4** | 100 | 75.1 |
| **BB (non-selective) (agonist radioligand)**  rat cerebral cortex | [^125^I][Tyr4]bombesin  (0.01) | **4** | 100 | 104.6 |
| **B_2_ (agonist radioligand)**  human recombinant (CHO cells) | [^3^H]bradykinin  (0.3 nM) | **4** | 100 | 110.6 |
| **CGRP (agonist radioligand)**  human recombinant (CHO cells) | [^125^I]hCGRPα  (0.03 nM) | **4** | 100 | 119.0 |
| **CB_1_ (agonist radioligand)**  human recombinant (CHO cells) | [^3^H]CP 55940  (0.5 nM) | **4** | 100 | 126.0 |
| **CCK_1_ (CCK_A_) (agonist radioligand)**  human recombinant (CHO cells) | [^125^I]CCK-8s  (0.08 nM) | **4** | 100 | 99.8 |
| **CCK_2_ (CCK_B_) (agonist radioligand)**  human recombinant (CHO cells) | [^125^I]CCK-8s  (0.08 nM) | **4** | 100 | 107.4 |
| **D_1_ (antagonist radioligand)**  human recombinant (CHO cells) | [^3^H]SCH 23390  (0.3 nM) | **4** | 100 | 58.6 |
| **D_2_S (antagonist radioligand)**  human recombinant (HEK-293 cells) | [^3^H]methy lspiperone  (0.3 nM) | **4**  **KM408** | 100  0.1  1  3  10  100 | 33.3  96.2  96.5  92.8  86.0  49.1 |
| **D_3_ (antagonist radioligand)**  human recombinant (CHO cells) | [^3^H]methylspiperone  (0.3 nM) | **4**  **KM408** | 100  0.003  0.03  0.3  3  30 | 11.9  104.3  103.2  101.7  95.3  42.5 |
| **D_4.4_ (antagonist radioligand)**  human recombinant (CHO cells) | [^3^H]methylspiperone  (0.3 nM) | **4**  **KM408** | 100  0.01  0.1  1  10  100 | 26.6  100.4  101.1  97.6  87.9  39.4 |
| **D_5_ (antagonist radioligand)**  human recombinant (GH4 cells) | [^3^H]SCH 23390  (0.3 nM) | **4** | 100 | 54.4 |
| **ET_A_ (agonist radioligand)**  human recombinant (CHO cells) | [^125^I]endothelin-1  (0.03 nM) | **4** | 100 | 130.9 |
| **ET_B_ (agonist radioligand)**  human recombinant (CHO cells) | [^125^I]endothelin-1  (0.03 nM) | **4** | 100 | 134.4 |
| **GABA (non-selective) (agonist radioligand)**  rat cerebral cortex | [^3^H]GABA  (10 nM) | **4** | 100 | 109.3 |
| **GAL_1_ (agonist radioligand)**  human recombinant (HEK-293  cells) | [^125^I]galanin  (0.1 nM) | **4** | 100 | 123.5 |
| **GAL_2_ (agonist radioligand)**  human recombinant (CHO cells) | [^125^I]galanin  (0.05 nM) | **4** | 100 | 111.9 |
| **PDGF (agonist radioligand)**  Balb/c 3T3 cells | [^125^I]PDGF BB  (0.03 nM) | **4** | 100 | 127.9 |
| **CXCR2 (IL-8B) (agonist radioligand)**  human recombinant (HEK-293 cells) | [^125^I]IL-8  (0.025 nM) | **4** | 100 | 112.2 |
| **CCR1 (agonist radioligand)**  human recombinant (HEK-293  cells) | [^125^I]MIP-1α  (0.01 nM) | **4** | 100 | 100.7 |
| **TNF-α (agonist radioligand)**  U-937 cells | [^125^I]TNF-α   - 1. nM) | **4** | 100 | 96.5 |
| **H_1_ (antagonist radioligand)**  human recombinant (HEK-293  cells) | [^3^H]pyrilamine  (1 nM) | **4** | 100 | 58.9 |
| **H_2_ (antagonist radioligand)**  human recombinant (CHO cells) | [^125^I]APT  (0.075 nM) | **4** | 100 | 90.3 |
| **MC_4_ (agonist radioligand)**  human recombinant (CHO cells) | [^125^I]NDP-α-MSH  (0.05 nM) | **4** | 100 | 92.0 |
| **MT_1_ (ML_1A_) (agonist radioligand)**  human recombinant (CHO cells) | [^125^I]2-Iodomelatonin  (0.01 nM) | **4** | 100 | 55.1 |
| **M_1_ (antagonist radioligand)**  human recombinant (CHO cells) | [^3^H]pirenzepine  (2 nM) | **4**  **KM408** | 100  0.1  1  3  10  100 | 46.7  113.9  107.8  123.4  99.7  43.1 |
| **M_2_ (antagonist radioligand)**  human recombinant (CHO cells) | [^3^H]AF-DX 384  (2 nM) | **4** | 100 | 58.4 |
| **M_3_ (antagonist radioligand)**  human recombinant (CHO cells) | [^3^H]4-DAMP  (0.2 nM) | **4** | 100 | 63.5 |
| **M_4_ (antagonist radioligand)**  human recombinant (CHO cells) | [^3^H]4-DAMP  (0.2 nM) | **4**  **KM408** | 100  0.1  1  3  10  100 | 30.5  98.0  92.0  82.7  67.0  23.8 |
| **M_5_ (antagonist radioligand)**  human recombinant (CHO cells) | [^3^H]4-DAMP  (0.3 nM) | **4**  **KM408** | 100  0.01  0.1  1  10  100 | 21.5  104.1  92.6  96.8  76.6  26.0 |
| **NK_1_ (agonist radioligand)**  U-373MG cells (endogenous) | [^125^I]BH-SP  (0.15 nM) | **4** | 100 | 82.1 |
| **NK_2_ (agonist radioligand)**  human recombinant (CHO cells) | [^125^I]NKA  (0.1 nM) | **4** | 100 | 88.7 |
| **NK_3_ (antagonist radioligand)**  human recombinant (CHO cells) | [^3^H]SR 142801  (0.4 nM) | **4** | 100 | 91.8 |
| **Y_1_ (agonist radioligand)**  SK-N-MC cells (endogenous) | [^125^I]peptide YY  (0.025 nM) | **4** | 100 | 122.3 |
| **Y_2_ (agonist radioligand)**  KAN-TS cells | [^125^I]peptide YY  (0.015 nM) | **4** | 100 | 126.5 |
| **NTS_1_ (NT_1_) (agonist radioligand)**  human recombinant (CHO cells) | [^125^I]Tyr3-Neurotensin  (0.05 nM) | **4** | 100 | 128.1 |
| **δ (DOP) (agonist radioligand)**  human recombinant (CHO cells) | [^3^H]DADLE  (0.5 nM) | **4** | 100 | 97.0 |
| **κ (KOP) (agonist radioligand)**  rat recombinant (CHO cells) | [^3^H]U 69593  (1 nM) | **4**  **KM408** | 100  0.01  0.1  1  10  100 | 29.0  104.2  98.3  97.3  72.1  21.1 |
| **μ (MOP) (agonist radioligand)**  human recombinant (HEK-293  cells) | [^3^H]DAMGO  (0.5 nM) | **4** | 100 | 50.1 |
| **NOP (ORL1) (agonist radioligand)**  human recombinant (HEK-293  cells) | [^3^H]nociceptin  (0.2 nM) | **4** | 100 | 95.5 |
| **PAC_1_ (PACAP) (agonist radioligand)**  human recombinant (CHO cells) | [^125^I]PACAP1-27  (0.015 nM) | **4** | 100 | 107.7 |
| **PPARγ (agonist radioligand)**  human recombinant (*E. coli*) | [^3^H]rosiglitazone  (5 nM) | **4** | 100 | 92.3 |
| **PCP (antagonist radioligand)**  rat cerebral cortex | [^3^H]TCP  (10 nM) | **4** | 100 | 72.6 |
| **EP_2_ (agonist radioligand)**  human recombinant (HEK-293  cells) | [^3^H]PGE2  (3 nM) | **4** | 100 | 75.4 |
| **IP (PGI_2_) (agonist radioligand)**  human recombinant (HEK-293  cells) | [^3^H]iloprost  (6 nM) | **4** | 100 | 97.2 |
| **P2X (agonist radioligand)**  rat urinary bladder | [^3^H]α,β-MeATP  (3 nM) | **4** | 100 | 100.6 |
| **P2Y (agonist radioligand)**  rat cerebral cortex | [^35^S]dATPαS  (10 nM) | **4** | 100 | 102.2 |
| **5-HT_1A_ (agonist radioligand)**  human recombinant (HEK-293  cells) | [^3^H]8-OH-DPAT  (0.3 nM) | **4**  **KM408** | 100  0.001  0.01  0.1  1  10 | 2.1  100.3  95.9  88.6  55.6  12.5 |
| **5-HT_1B_ (antagonist radioligand)**  rat cerebral cortex | [^125^I]CYP  (+30 μM isoproterenol)  (0.1 nM) | **4** | 100 | 79.7 |
| **5-HT_2A_ (antagonist radioligand)**  human recombinant (HEK-293  cells) | [^3^H]ketanserin  (0.5 nM) | **4**  **KM408** | 100  0.1  1  3  10  100 | 37.1  103.9  95.0  94.1  85.1  36.9 |
| **5-HT_2B_ (agonist radioligand)**  human recombinant (CHO cells) | [^125^I](±)DOI  (0.2 nM) | **4**  **KM408** | 100  0.001  0.01  0.1  1  10 | 2.3  129.6  120.7  103.3  88.0  28.3 |
| **5-HT_2C_ (antagonist radioligand)**  human recombinant (HEK-293  cells) | [^3^H]mesulergine  (1 nM) | **4**  **KM408** | 100  0.1  1  3  10  100 | 44.6  95.6  87.1  95.2  82.4  40.6 |
| **5-HT_3_ (antagonist radioligand)**  human recombinant (CHO cells) | [^3^H]BRL 43694  (0.5 nM) | **4** | 100 | 76.2 |
| **5-HT_5a_ (agonist radioligand)**  human recombinant (HEK-293  cells) | [^3^H]LSD  (1.5 nM) | **4** | 100 | 52.8 |
| **5-HT_6_ (agonist radioligand)**  human recombinant (CHO cells) | [^3^H]LSD  (2 nM) | **4** | 100 | 101.6 |
| **5-HT_7_ (agonist radioligand)**  human recombinant (CHO cells) | [^3^H]LSD  (4 nM) | **4**  **KM408** | 100  0.01  0.1  1  10  100 | 26.4  106.9  105.6  104.6  66.1  20.2 |
| **Sigma (non-selective) (agonist radioligand)**  rat cerebral cortex | [^3^H]DTG  (8 nM) | **4**  **KM408** | 100  0.001  0.01  0.1  1  10 | -2.7  104.0  97.1  50.0  13.1  1.4 |
| **Sigma 1 (agonist radioligand)**  human endogenous (Jurkat cells) | [^3^H] (+) pentazocine  (15 nM) | **KM408** | 10 | 95.0 |
| **Sigma 2 (agonist radioligand)**  human endogenous (Jurkat cells) | [^3^H] DTG (+1μM (+)Pentazocine)  (25 nM) | **KM408** | 10 | 94.8 |
| **sst (non-selective) (agonist radioligand)**  AtT-20 cells | [^125^I]Tyr11-somatostatin-14  (0.05 nM) | **4** | 100 | 75.5 |
| **GR (agonist radioligand)**  IM-9 cells (cytosol) | [^3^H]dexamethasone  (1.5 nM) | **4** | 100 | 111.7 |
| **VPAC_1_ (VIP_1_) (agonist radioligand)**  human recombinant (CHO cells) | [^125^I]VIP  (0.04 nM) | **4** | 100 | 117.9 |
| **V_1a_ (agonist radioligand)**  human recombinant (CHO cells) | [^3^H]AVP  (0.3 nM) | **4** | 100 | 99.8 |
| **Ca^2+^ channel (L, verapamil site) (phenylalkylamine) (antagonist radioligand)**  rat cerebral cortex | [^3^H]D888  (3 nM) | **4**  **KM408** | 100  0.1  1  3  10  100 | 38.5  101.9  104.4  93.6  80.9  34.8 |
| **K_V_ channel (antagonist radioligand)**  rat cerebral cortex | [^125^I]α-dendrotoxin | **4** | 100 | 104.2 |
| **SK_Ca_ channel (antagonist radioligand)**  rat cerebral cortex | [^125^I]apamin  (0.007 nM) | **4** | 100 | 90.5 |
| **Na^+^ channel (site 2) (antagonist radioligand)**  rat cerebral cortex | [^3^H]batrachotoxinin  (10 nM) | **4**  **KM408** | 100  0.003  0.03  0.3  3  30 | 11.6  117.9  111.0  101.1  92.0  40.3 |
| **Cl^-^ channel (GABA-gated) (antagonist radioligand)**  rat cerebral cortex | [^35^S]TBPS  (3 nM) | **4** | 100 | 99.6 |
| **norepinephrine transporter (antagonist radioligand)**  human recombinant (CHO cells) | [^3^H]nisoxetine  (1 nM) | **4** | 100 | 55.0 |
| **dopamine transporter (antagonist radioligand)**  human recombinant (CHO cells) | [^3^H]BTCP  (4 nM) | **4**  **KM408** | 100  0.1  1  3  10  100 | 29.6  93.9  87.8  94.4  72.6  29.6 |
| **5-HT transporter (antagonist radioligand)**  human recombinant (CHO cells) | [^3^H]imipramine  (2 nM) | **4**  **KM408** | 100  0.003  0.03  0.3  3  30 | 5.1  105.1  112.1  92.8  61.0  11.5 |
| **hERG**  human recombinant (HEK-293  cells) | [^3^H]astemizole  (2 nM) | **KM408**  **5a**  **6a** | 100  100  100 | 26.1  23.9  20.9 |

## Table S2. Patch-clamp results for 4 on NIE-115 cells.

| **Concentration**  **[µM]** | **No. of cells** | **% of control +/- SEM** | **Holding potential**  **[mV]** |
| --- | --- | --- | --- |
| 100 | 7 | 55 +/- 5 | -90 |
| 100 | 7 | 26 +/- 3 | -60 |

## Table S3. Affinities of compound KM408 towards Na_v_1.1-1.8 channels.

|  | **Na_V_ channel subtype**  **IC_50_ [µM]** | | | | | | | |
| --- | --- | --- | --- | --- | --- | --- | --- | --- |
|  | 1.1 | 1.2 | 1.3 | 1.4 | 1.5 | 1.6 | 1.7 | 1.8 |
| **Tonic block** | 64.2 | 48.7 | 49.2 | 68.0 | 47.9 | 60.1 | 52.0 | 44.1 |
| **10 Hz** | 17.5 | 15.6 | 13.1 | 8.17 | 4.85 | 20.5 | 15.4 | 15.2 |
| **Inactivated state** | <10 | <10 | <10 | <10 | <10 | <10 | <10 | <10 |

## Table S4. Comprehensive pharmacological data for compound 18.

|  | | **Time [h]** | |
| --- | --- | --- | --- |
|  |  | **0.5** | **2.0** |
| **Test** | **Dose [mg/kg]** | **Number of animals active or toxic over the number tested** | **Number of animals active or toxic over the number tested** |
| **6 Hz** | 30 | 0/4 | 0/4 |
|  | 100 | 4/4 | 0/4 |
|  | 300 | 4/4 | 4/4 |
| **MES** | 30 | 3/4 | 0/4 |
|  | 100 | 4/4 | 0/4 |
|  | 300 | 4/4 | 4/4 |
| **Rotarod** | 30 | 0/8 | 0/8 |
|  | 100 | 2/8 | 0/8 |
|  | 300 | 8/8 (sedated) | 0/8 (unable to grasp rotarod) |

## Table S5. Analgesic activity of compound KM408 and 5a (SNL model, rats, *i.p.*).

| **Time**  **[h]** | **KM408**  **(6 mg/kg b.w.)** | | **5a**  **(4 mg/kg b.w.)** | |
| --- | --- | --- | --- | --- |
|  | **Mean value ± S.E.M.** | | | |
|  | **Threshold ± S.E.M.**  **[g]** | **% Pre-Drug ± S.E.M.** | **Threshold ± S.E.M.**  **[g]** | **% Pre-Drug ± S.E.M.** |
| **0.0** | 2.88 ± 0.59 | 100 ± 21 | 2.74 ± 0.3 | 100 ± 11 |
| **0.5** | 3.07 ± 0.51 | 131 ± 15 | 4.57 ± 0.77 | 203 ± 47 |
| **1.0** | 5.94 ± 1.19 | 202 ± 19 | 8.61 ± 2.38 | 317 ± 79 * |
| **2.0** | 8.23 ± 1.56 | 301 ± 45 * | 6.62 ± 1.59 | 233 ± 46 |
| **4.0** | 7.09 ± 1.31 | 260 ± 39 * | 2.91 ± 0.3 | 124 ± 18 |
| **6.0** | 5.34 ± 1.32 | 186 ± 31 | 2.57 ± 0.49 | 107 ± 20 |

* Significantly different from control, p<0.01; data presented as mean of 8 animals. Threshold for foot withdrawal of the ligated leg is presented as mean mass in grams.

# II. Figures


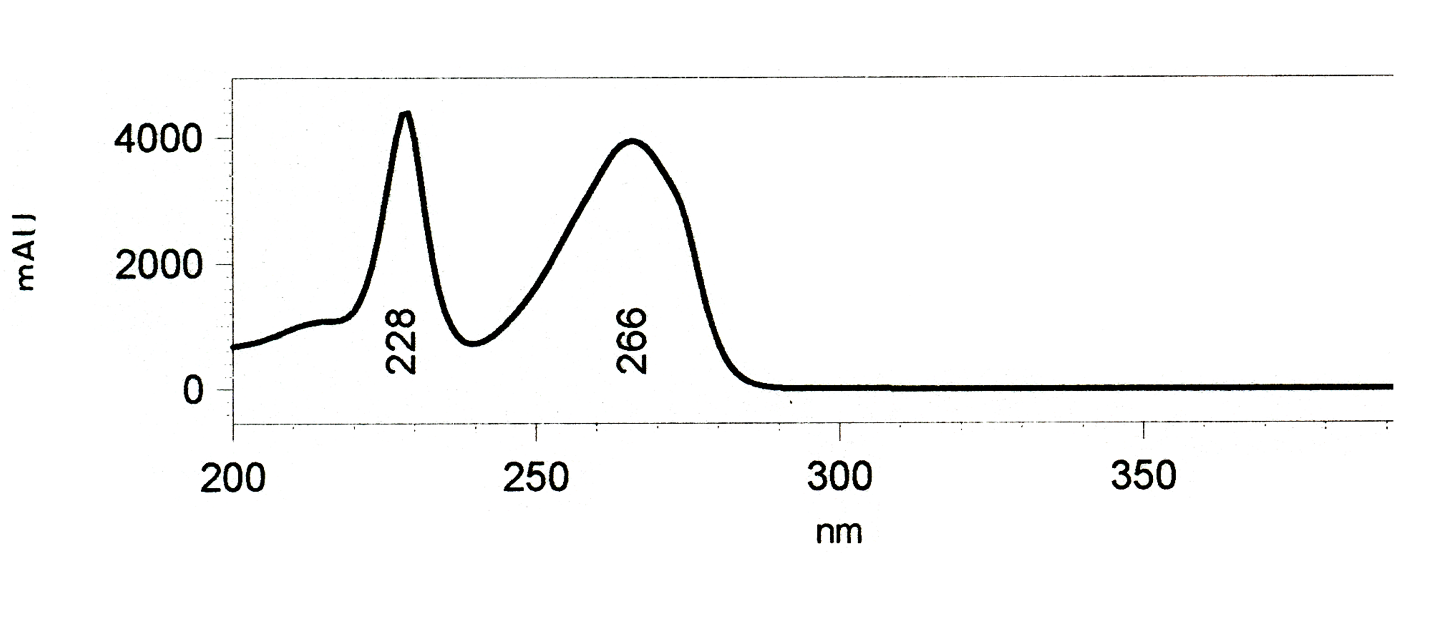


**Figure S1.** Absorption spectrum of **KM-408**.

**
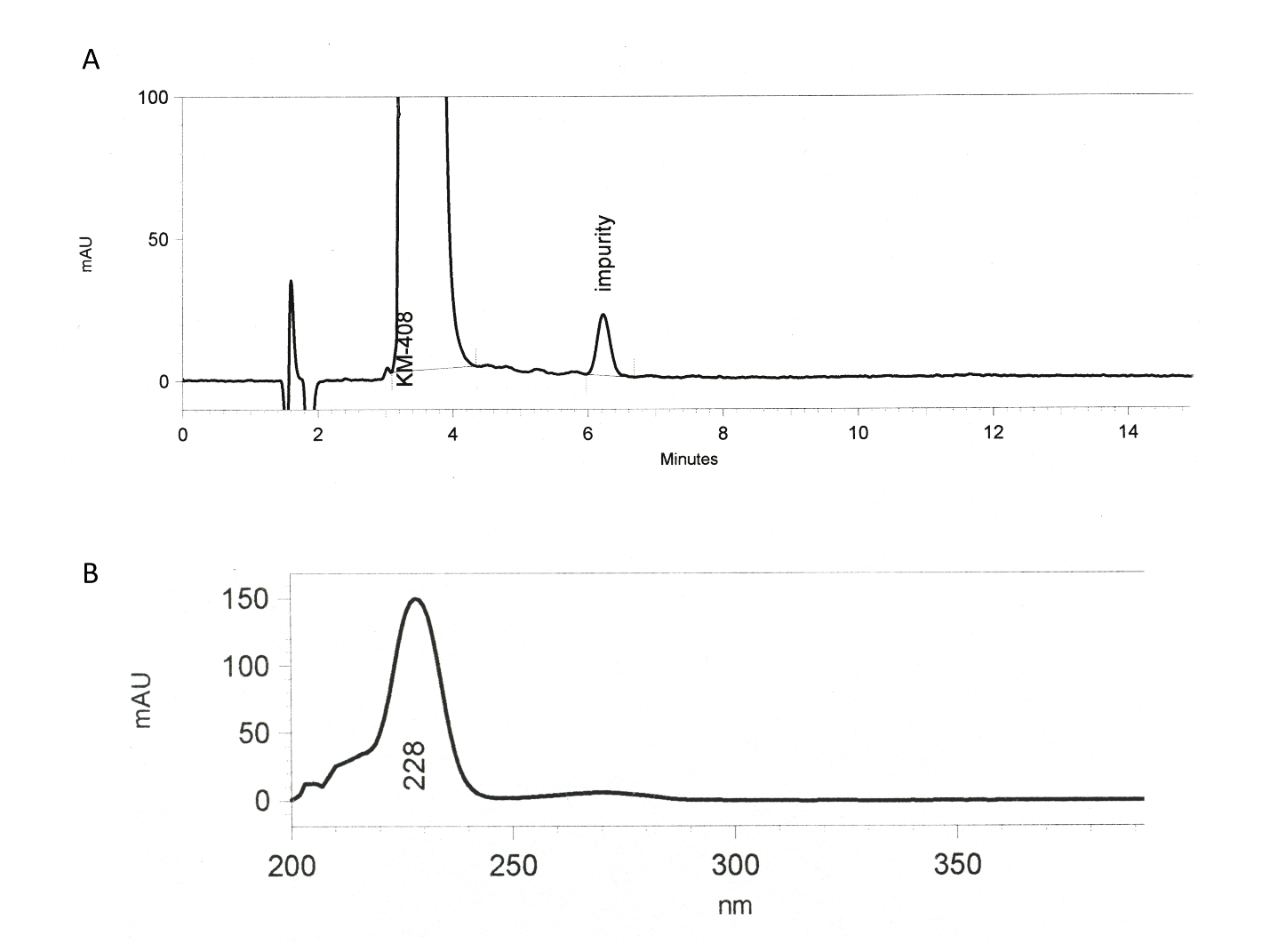
**

**Figure S2.** (A) HPLC chromatogram of **KM-408**; (B) Absorption spectrum of **KM-408** impurity.


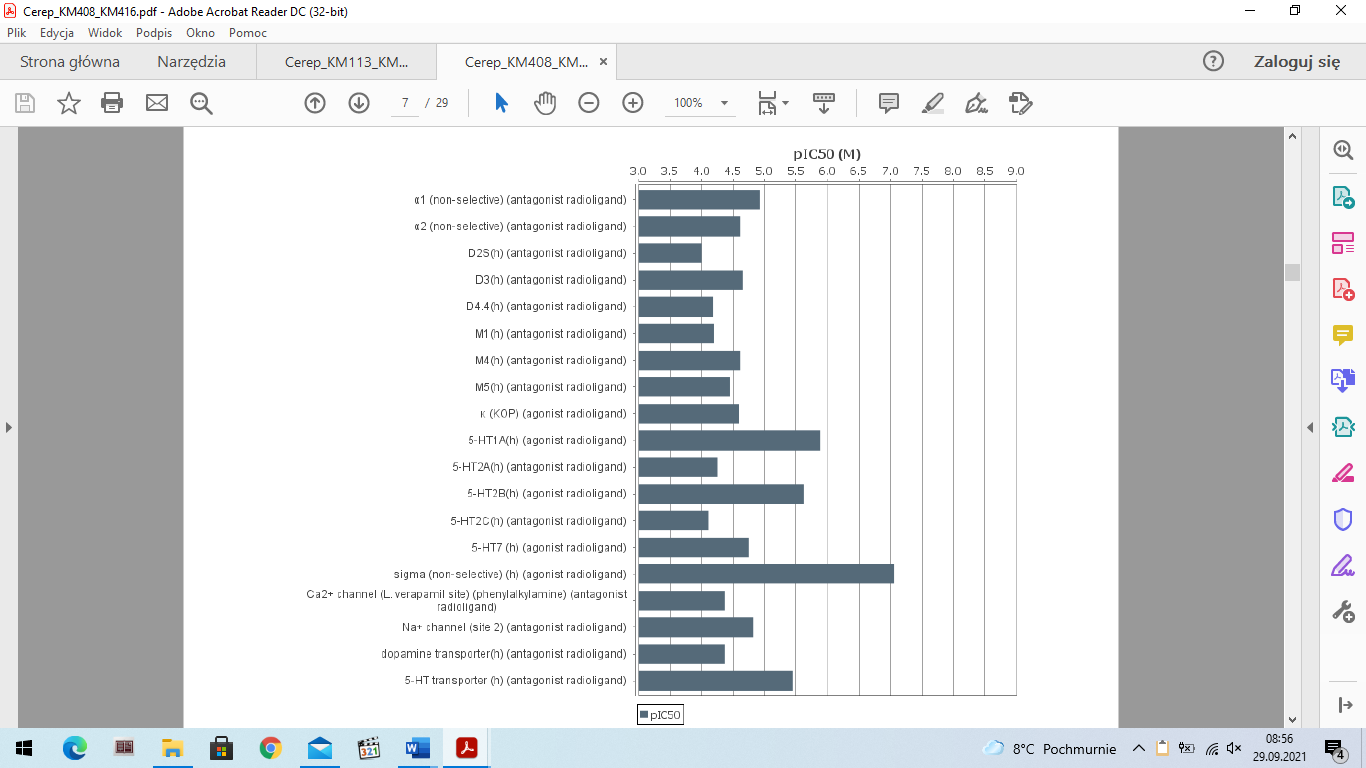


## Figure S3. Results of IC_50_ determination for KM408.

**
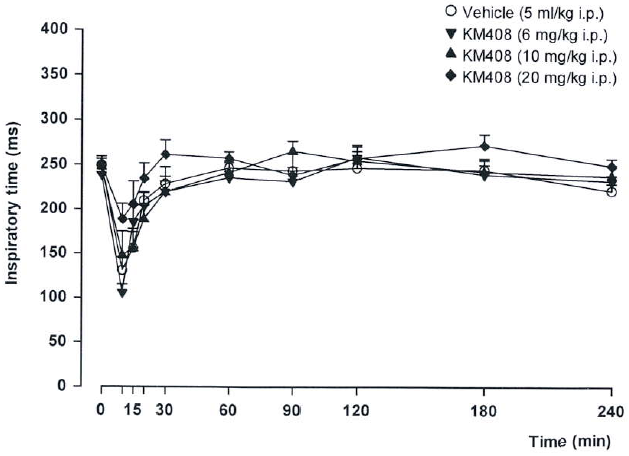
**

## Figure S4. Effects of KM408 (6, 10 and 20 mg/kg) on inspiratory time following i.p. administration of single doses in the conscious male Wistar rat. Mean +/- SEM (n=6). Inter-group comparison (versus vehicle): no indication = not significant: * = p<0.05; ** = p<0.01; *** = p<0.001.


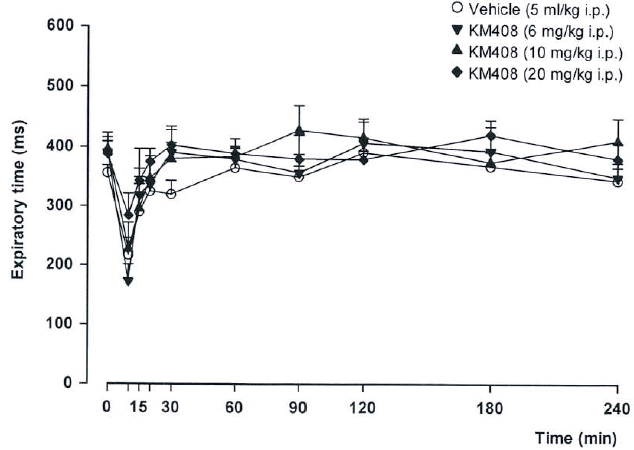


## Figure S5. Effects of KM408 (6, 10 and 20 mg/kg) on expiratory time following i.p. administration of single doses in the conscious male Wistar rat. Mean +/- SEM (n=6). Inter-group comparison (versus vehicle): no indication = not significant: * = p<0.05; ** = p<0.01; *** = p<0.001.


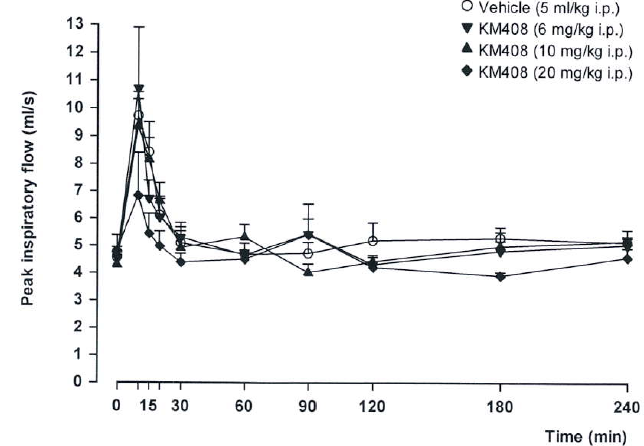


## Figure S6. Effects of KM408 (6, 10 and 20 mg/kg) on peak inspiratory flow following i.p. administration of single doses in the conscious male Wistar rat. Mean +/- SEM (n=6). Inter-group comparison (versus vehicle): no indication = not significant: * = p<0.05; ** = p<0.01; *** = p<0.001.


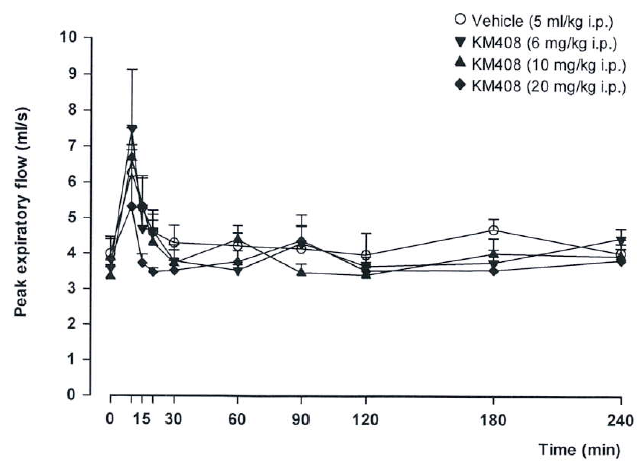


## Figure S7. Effects of KM408 (6, 10 and 20 mg/kg) on peak expiratory flow following i.p. administration of single doses in the conscious male Wistar rat. Mean +/- SEM (n=6). Inter-group comparison (versus vehicle): no indication = not significant: * = p<0.05; ** = p<0.01; *** = p<0.001.

##
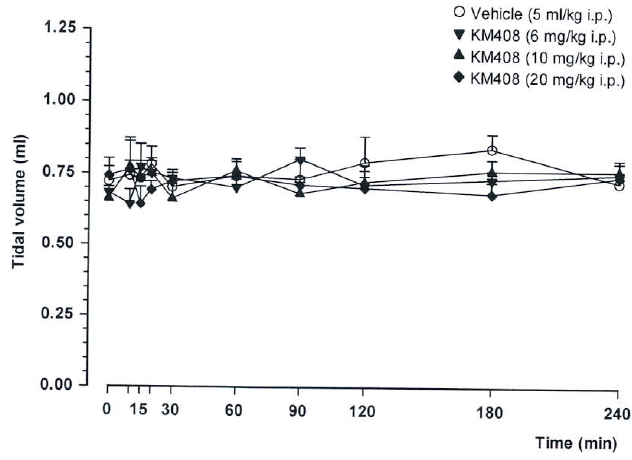
 Figure S8. Effects of KM408 (6, 10 and 20 mg/kg) on tidal volume following i.p. administration of single doses in the conscious male Wistar rat. Mean +/- SEM (n=6). Inter-group comparison (versus vehicle): no indication = not significant: * = p<0.05; ** = p<0.01; *** = p<0.001.


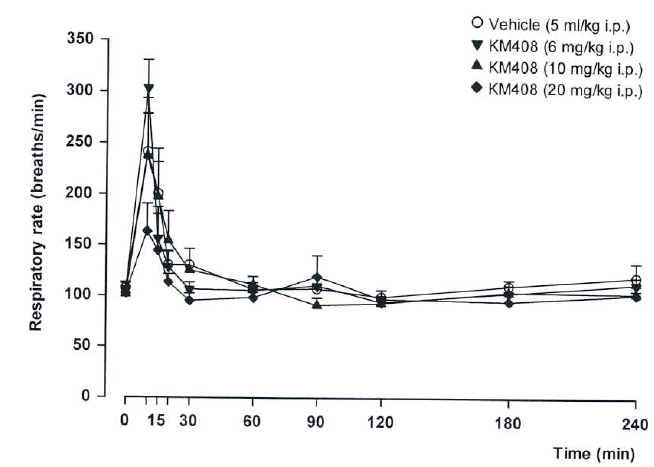


## Figure S9. Effects of KM408 (6, 10 and 20 mg/kg) on respiratory rate following i.p. administration of single doses in the conscious male Wistar rat. Mean +/- SEM (n=6). Inter-group comparison (versus vehicle): no indication = not significant: * = p<0.05; ** = p<0.01; *** = p<0.001.


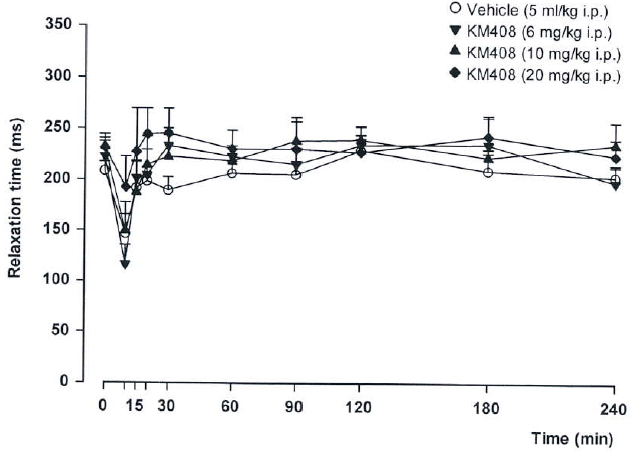


## Figure S10. Effects of KM408 (6, 10 and 20 mg/kg) on relaxation time following i.p. administration of single doses in the conscious male Wistar rat. Mean +/- SEM (n=6). Inter-group comparison (versus vehicle): no indication = not significant: * = p<0.05; ** = p<0.01; *** = p<0.001.


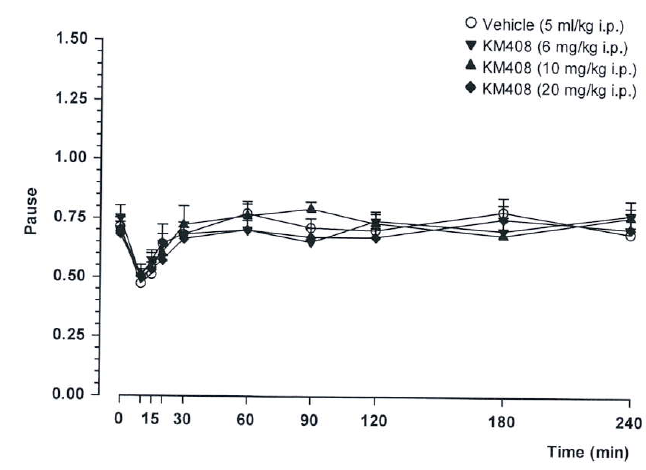


## Figure S11. Effects of KM408 (6, 10 and 20 mg/kg) on pause following i.p. administration of single doses in the conscious male Wistar rat. Mean +/- SEM (n=6). Inter-group comparison (versus vehicle): no indication = not significant: * = p<0.05; ** = p<0.01; *** = p<0.001.


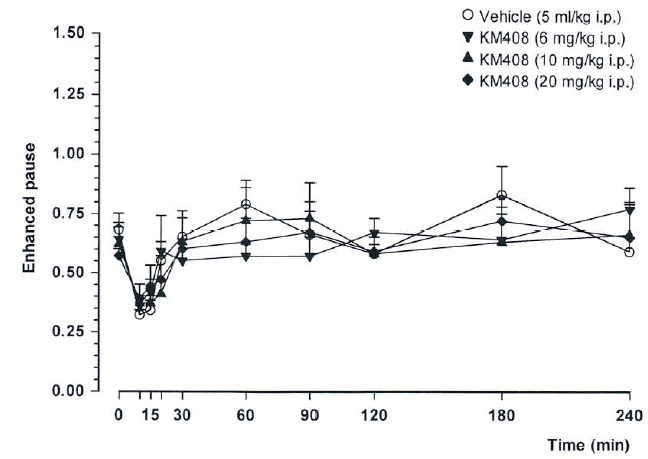


## Figure S12. Effects of KM408 (6, 10 and 20 mg/kg) on enhanced pause following i.p. administration of single doses in the conscious male Wistar rat. Mean +/- SEM (n=6). Inter-group comparison (versus vehicle): no indication = not significant: * = p<0.05; ** = p<0.01; *** = p<0.001.


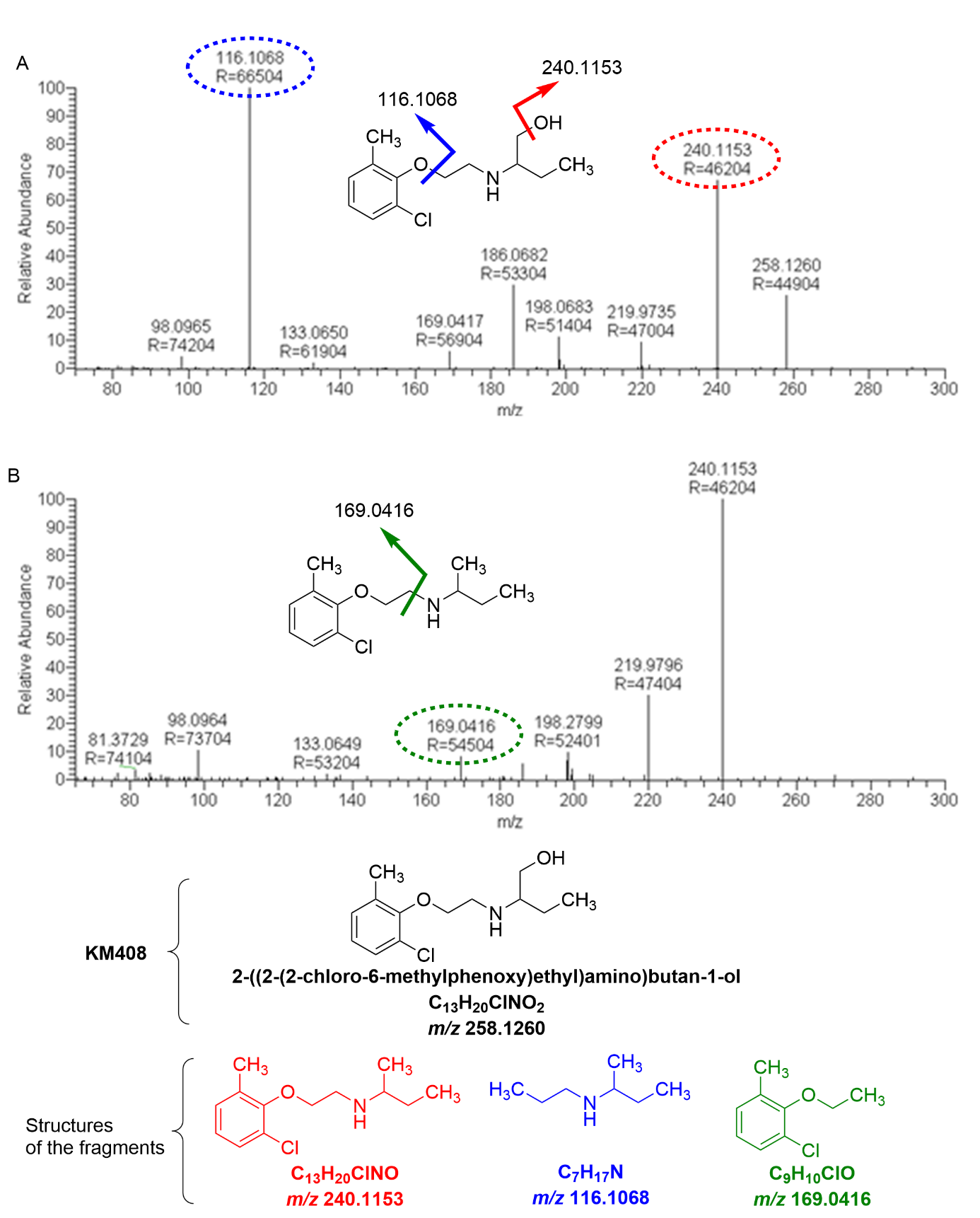


**Figure S13.** Fragmentation mass spectrum MS2 of **KM-408** (m/z 258.1260) (A) and mass spectrum MS3 of the ion m/z 240.1153 (B) using He as a collision gas together with likely fragmentation pathways and the structures of the fragments.
